# Tuning the selectivity of P_4_ reduction at alkaline-earth metal centres†

**DOI:** 10.1039/d4sc08502g

**Published:** 2025-02-05

**Authors:** Stefan Thum, Oliver P. E. Townrow, Jens Langer, Sjoerd Harder

**Affiliations:** a Inorganic and Organometallic Chemistry, Friedrich-Alexander-Universität Erlangen-Nürnberg Egerlandstraße 1 91058 Erlangen Germany sjoerd.harder@fau.de

## Abstract

Reduction of P_4_ with β-diketiminate Mg^I^ complexes, (BDI)MgMg(BDI), depends strongly on the bulk of the ligand. Whereas superbulky BDI ligands gave selective reduction to P_4_^2−^ in a butterfly conformation, reduction with a less bulky ligand gave various products among which P_8_^4−^ had a realgar-type structure. The selectivity of P_4_ reduction can also be controlled by metal choice. Reduction of P_4_ with Ca^I^ synthons of general type (BDI*)Ca–X–Ca(BDI*) in which BDI* is a superbulky ligand and X is a bridging dianion (C_6_H_6_^2−^ < *p*-xylene^2−^ < N_2_^2−^) led to reduction of P_4_ to the very common, stable Zintl anion P_7_^3−^. Monitoring this process with ^31^P NMR shows that *cyclo*-P_4_^2−^ is an intermediate en route to P_7_^3−^. Conversion rates increase with increasing reducing power: X = C_6_H_6_^2−^ < *p*-xylene^2−^ < N_2_^2−^. A complex with the weakly reducing DBA^2−^ dianion led to selective P_4_ reduction to *cyclo*-P_4_^2−^ (DBA = 9,10-dimethyl-diboraanthracene). DBA inhibits P_4_-to-P_7_ conversion, most likely by capturing the electron needed for further P_4_ reduction by radical processes. Experimental investigations are supported by crystal structure determinations and a computational DFT study which also shows that the nature of metal–P_4_ bonding (covalent or ionic) determines the preference for formation of butterfly-shaped P_4_^2−^ or planar 6π-electron aromatic *cyclo*-P_4_^2−^.

## Abstract 2

The selectivity of P_4_ reduction with Mg^I^ complexes or Ca^I^ synthons can be tuned with ligand bulk or metal choice. Also the addition of inhibitors influences product selectivity.

## Graphical abstract

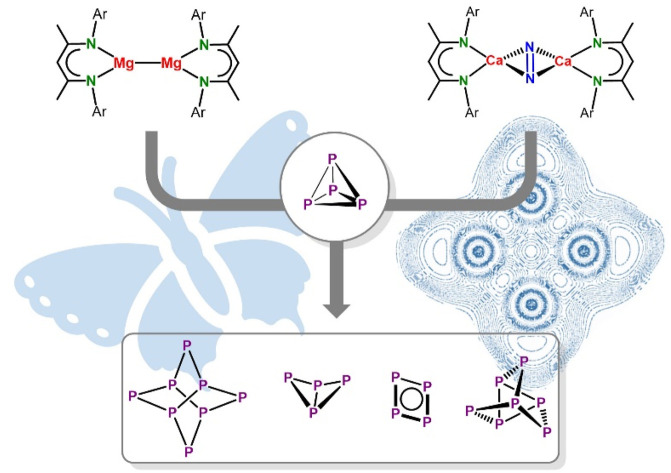

## Introduction

White phosphorus (P_4_) is a commodity reagent for the production of industrially relevant P-containing products. While traditional bulk processes convert P_4_ with highly corrosive Cl_2_ to PCl_3_ for further functionalization with polar organo-metallic reagents, current research initiatives aim for catalytic protocols to directly convert P_4_ to organophosphorus compounds.^1^ In this light, the activation and chemical breakdown of P_4_ is an important research field. Being a highly strained molecule, P_4_ can be easily oxidized or reduced and shows diverse reactivity. Reacting either as an electrophile, nucleophile, or as an e-donor/acceptor, it could be seen as a chameleon in P-chemistry.^2^

**Scheme 1:**
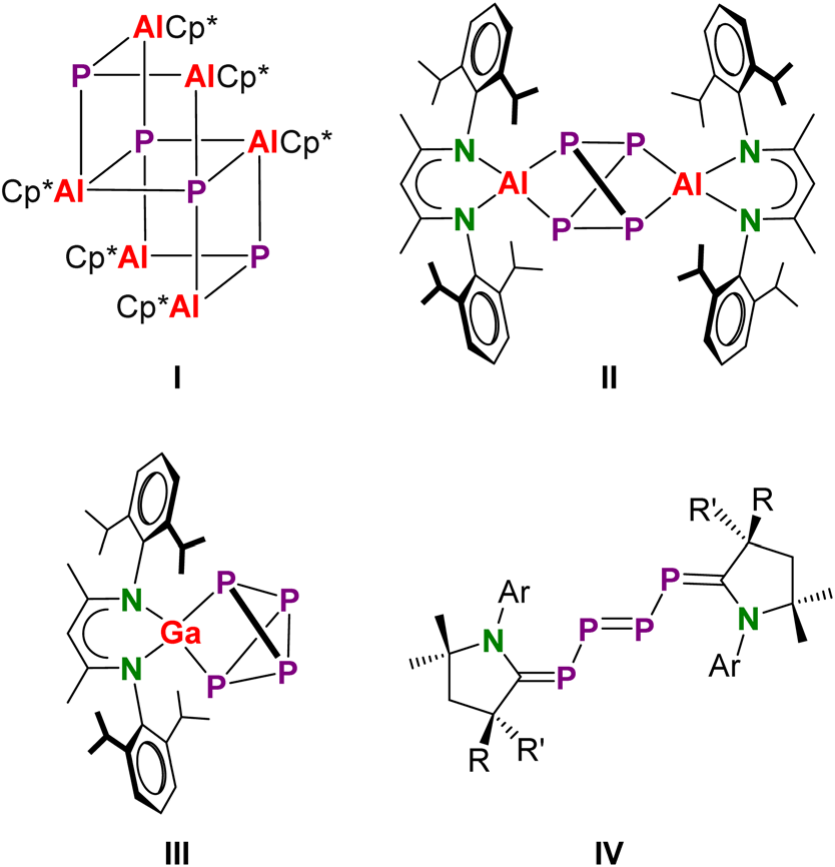
Selected products of P_4_ activation by low-valent main group compounds.

Although P_4_ is inherently highly reactive, reaction pathways often remain unclear and selective conversions are difficult to achieve. Numerous groups have reported on P_4_ activation using the rich redox reactivity of the transition metals.^3–6^ Recent developments in low-valent p-block chemistry stimulated P_4_ activation with reagents that, due to small HOMO–LUMO gaps, show transition metal-like reactivity.^7–9^ Earlier highlights of this work include the insertion of (Me_3_Si)_3_CGa in three P–P bonds of P_4_ by Uhl and coworkers^10^ or the complete reduction of P_4_ by Cp*Al to give a P^3−^ containing cluster (Cp*Al)_6_(P)_4_ (I, Scheme 1) by the Schnöckel group.^11^ In contrast, the bulkier β-diketiminate complex (BDI)Al reacted to give a complex of the P_4_^4−^ anion (II), formed by 4e-reduction and cleavage of two edges in the P_4_ tetrahedron; BDI is herein defined as HC[C(Me)-N(DIPP)]_2_ (DIPP = 2,6-diisopropylphenyl). Reaction with the softer reducing agent (BDI)Ga led only to 2e-reduction and cleavage of one P–P edge (III).^12^ The valence isoelectronic cyclic alkyl amino carbenes (CAACs) have also been shown to activate P_4_ (IV),^13^ whereas silylenes show either 4e-reduction giving P_4_^4−^, like in II, or 2e-reduction resulting in P_4_^2−^, like in III.^14^

Given the plethora of important breakthroughs in low-oxidation state s-block metal chemistry,^15–17^ it is remarkable that there is a complete lack of research on P_4_ reduction with these highly reactive early main group metal complexes. The reduction chemistry of one of the first Mg^I^ complexes, (BDI)MgMg(BDI),^18^ has been extensively investigated^15^ but we are unaware of reactivity studies with P_4_. However, this mild reducing agent has been reported to reduce the P_5_^−^ ring in Cp*Fe(P_5_).^19^ The far majority of P_4_ activation studies with s-block metal reagents exploit their superb nucleophilicity. Classical examples include P–P bond cleavage in P_4_ by nucleophilic addition of RLi or Grignard reagents.^20^ An interesting case of nucleophilic activation of P_4_ is its reaction with nucleo-philic hydride reagents like [(BDI)Ca(μ_2_-H)]_2_ which after subsequent H_2_ release gave the reduction-like product [(BDI)Ca]_3_(P_7_), containing the Zintl P_7_^3−^ ion.^19^ In this example, the calcium hydride complex reacts as a synthon for the hitherto unknown Ca^I^ complex (BDI)CaCa(BDI). Such reductive reactivity of group 2 metal hydride complexes is well-established.^21^

We herein report a systematic study on the reduction of P_4_ with Mg^I^ complexes or Ca^I^ synthons, *i.e.* Ca^II^ complexes containing electron-rich ligands that can react like the corresponding Ca^I^ species.^22−24^ We demonstrate that selectivity is largely dependent on metal choice, ligand bulk or the nature of the electron-rich ligand delivering the electrons for P_4_ reduction.

## Results and discussion

### P_4_ activation with Mg^I^ complexes

**Scheme 2:**
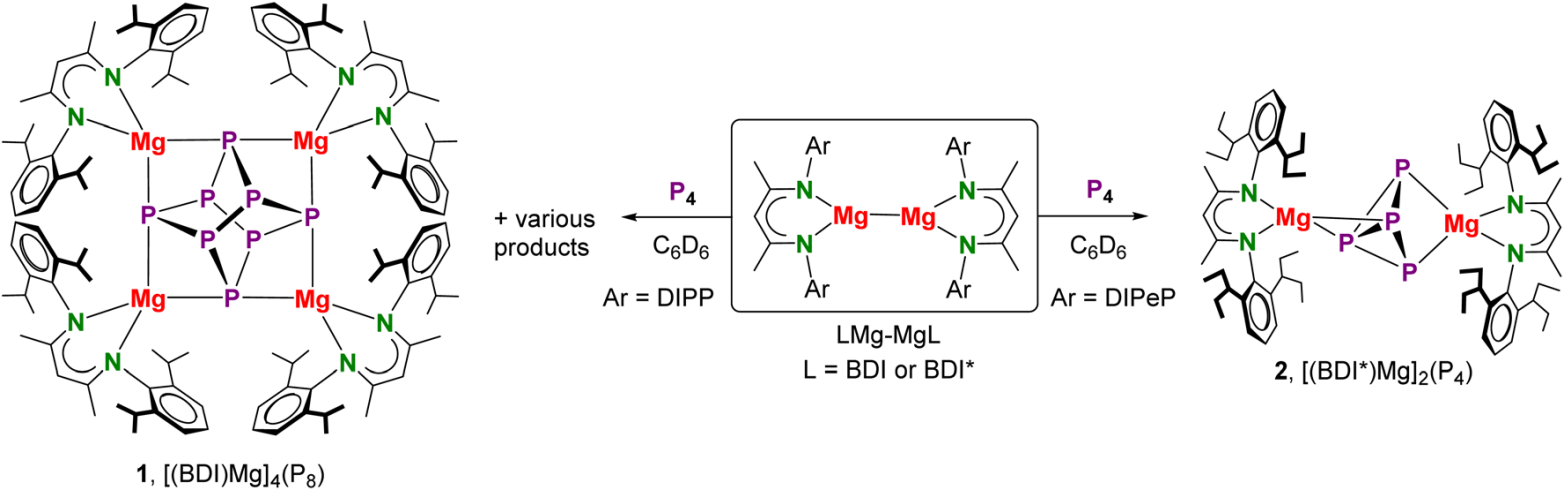
Activation of P_4_ by β-diketiminate Mg^I^ complexes.

**Fig. 1:**
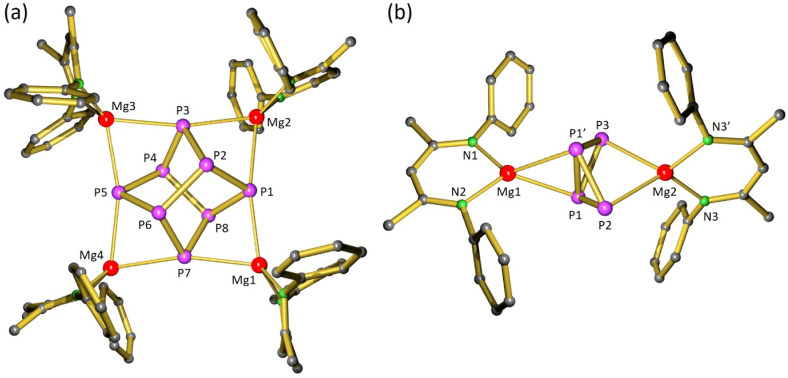
(a) Crystal structure of [(BDI)Mg]_4_(P_8_) (1) in which iPr-groups and H atoms are omitted for clarity and a view of the Mg_4_P_8_ core. (b) Crystal structure of [(BDI*)Mg]_2_(P_4_) (2); the Et_2_CH-groups and H atoms are omitted for clarity. A crystallographic mirror plane runs through the atoms Mg1, Mg2, P2, P3.

The direct reduction of P_4_ with β-diketiminate Mg^I^ complexes of type (BDI)MgMg(BDI) has so far not been described in the literature. This could be because ^1^H and ^31^P{^1^H} NMR monitoring of an equimolar mixture of [(BDI)Mg]_2_ and P_4_ in C_6_D_6_ at room temperature showed a highly unselective conversion (Scheme 2 and Fig. S37/S38†). Various side-reactions may originate from the poor solubility of the reactants and the heterogeneity of the reaction mixture. However, we found that gently heating the mixture for three days at 60 °C leads to further conversion and selective crystallization of a most insoluble reaction product [(BDI)Mg]_4_(P_8_) (1) in 10% yield (Fig. 1a). This minor product shows ^31^P NMR resonances at +68.3 and +145.0 ppm which were not observed in the crude product of the room temperature [(BDI)Mg]_2_/P_4_ conversion. This means that 1 was formed after thermal treatment. The P_8_^4−^ unit is isostructural and valence isoelectronic to α-P_4_S_4_, which is of the realgar-type,^25^ and flanked by four [(BDI)Mg]^+^ fragments at the corners with each Mg atom bound to two P atoms. Despite the high symmetry of the P_8_^4−^ anion, the complex shows no crystallographic symmetry. Although this structure is unprecedented in s-block metal chemistry, a few examples for P_8_^4−^ formation are known from transition metal,^26–28^ lanthanide,^29^ and gallium mediated P_4_ activation.^30^ Similar realgar-type polystibides Sb_8_ have been isolated as the corresponding [(BDI)Mg]_4_(Sb_8_) complexes with different BDI ligands.^31^ The Mg–P distances in 1 are in the narrow range of 2.599(1)–2.692(1) Å with P–Mg–P bite angles varying from 73.92(3)° to 74.79(3)°. In contrast to [(BDI)Mg]_4_(Sb)_8_ which exhibits an almost linear Mg–Sb–Mg arrangement, the Mg–P–Mg angles in 1 deviate slightly from linearity: 164.26(3)–168.05(3)°. Within the P_8_^4−^ anion in 1 there are two types of P–P bonds. The P–P bonds between three-coordinate P atoms, P2–P6 (2.2868(8) Å) and P4–P8 (2.2751(8) Å), are slightly elongated compared to the remaining P–P bonds ranging from 2.1999(7) to 2.2188(8) Å. The P–P–P angles range from 92.60(3)° to 102.67(3)°. These structural features are in agreement with those reported for the Sm^III^ cluster (Cp*_2_Sm)_4_(P_8_).^29^

In contrast, reaction of P_4_ with the more sterically hindered Mg^I^ complex [(BDI*)Mg]_2_,^32^ featuring a considerably elongated Mg–Mg interatomic distance,^33^ gave at room temperature overnight highly selective conversion; BDI* is defined as HC[C(Me)–N(DIPeP)]_2_ (DIPeP = 2,6-(Et_2_CH)-phenyl). In contrast to the unselective [(BDI)Mg]_2_/P_4_ conversion (Fig. S37/S38†), the crude product of the [(BDI*)Mg]_2_/P_4_ conversion showed a ^31^P{^1^H} NMR spectrum with only two triplet resonances in a 1 : 1 ratio (Fig. S41†). Despite the high selectivity of this reaction, the very good solubility induced by the Et_2_CH-substituents allowed for isolation of crystalline [(BDI*)Mg]_2_(P_4_) (2) in only 34% yield.

In agreement with two triplet signals in ^31^P{^1^H} NMR, inspection of the crystal structure revealed a P_4_^2−^ dianion in a butterfly conformation which is bridging two (BDI*)Mg^+^ units in an unusual η^2^,η^2^-fashion. Butterfly-shaped P_4_^2−^ anions usually bridge metals in η^1^,η^1^-fashion.^34^ Recently, Aldridge and co-workers isolated an odd example of P_4_^2−^ bridging between Al and K in η^2^,η^1^-fashion^35^ while Hill and co-workers reported bridging in η^2^,η^3^-fashion.^36^ Complex 2 could also be considered to consist of a magnesate anion (BDI*)Mg(P_4_)^−^ with two polar Mg–P bonds to the two-coordinate P atoms (P2, P3) of 2.573(4)–2.694(4) Å, charge-balanced by a (BDI*)Mg^+^ cation that interacts with Mg–P interactions of circa 2.606(4) Å with both three-coordinate P-atoms (P1 and P1′). Due to disorder of the bridging P_4_^2−^ anion (Fig. S62†) a more accurate discussion of the crystal structure is not possible.

### P_4_ activation with Ca^I^ synthons

Although β-diketiminate stabilized Ca^I^ complexes are currently unknown, we reported a range of (BDI*)Ca–X–Ca(BDI*) complexes with various bridging X^2−^ anions that react like a low-valent Ca^I^ complex (X = N_2_, benzene, *p*-xylene).^22,24^ Their reducing ability is directly related to the 2e-oxidation of the bridging anion, X^2−^ → X + 2e^−^, and therefore to the reduction potential of X.

**Scheme 3:**
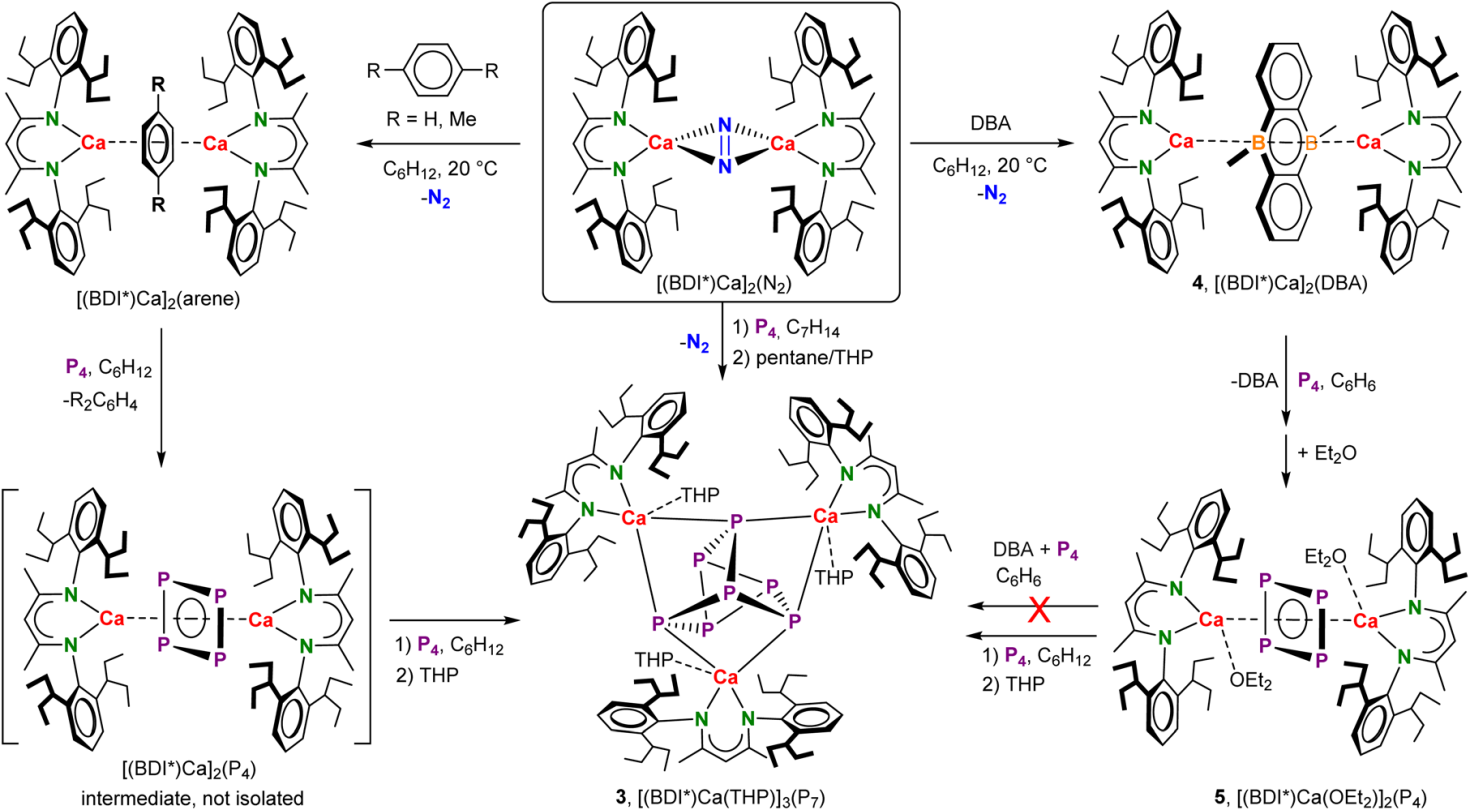
Activation of P_4_ by Ca^I^ synthons.

Since the most reducing Ca^I^ synthon [(BDI*)Ca]_2_(N_2_) is unstable in aromatic solvents,^22^ the reaction with P_4_ was carried out in methylcyclohexane (Scheme 3). Reaction of the Ca^I^ synthon with a methylcyclohexane solution of P_4_ at room temperature led to immediate N_2_ evolution and a colour change from dark brown to dark orange. Analysis of the crude reaction mixture by ^31^P{^1^H} NMR spectroscopy revealed after 30 minutes a sharp low-field singlet at 458.4 ppm and a rather broad high-field singlet at −85.3 ppm in a ratio of 0.09 : 0.91, respectively (Fig. S42†). Continued stirring for another 4 hours showed the selective formation of one species corresponding to the broad high-field signal at −85.3 ppm (Fig. S43†). Layering a saturated methylcyclohexane/pentane solution with a few drops of tetrahydropyran (THP) resulted in the isolation of yellow crystals of composition [(BDI*)Ca(THP)]_3_(P_7_) (3) in a 28% yield.

**Fig. 2:**
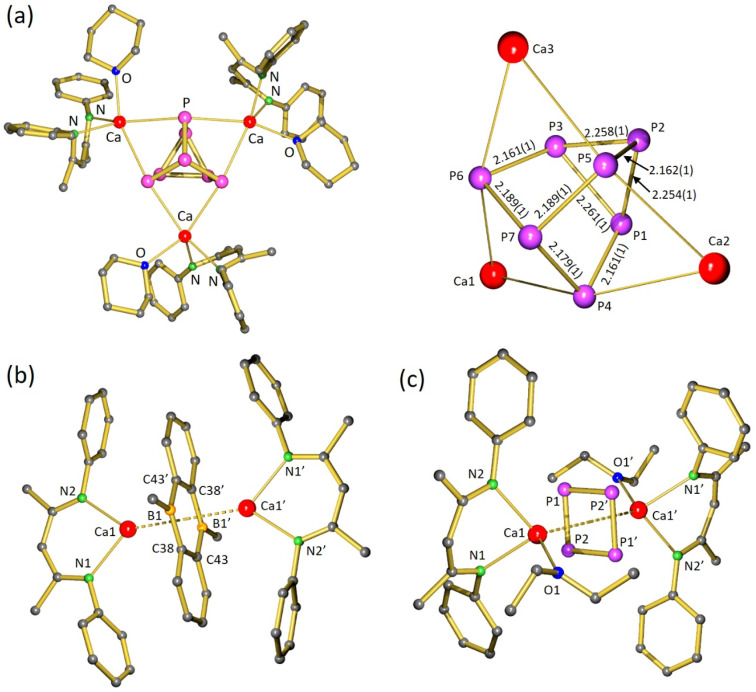
(a) Crystal structure of [(BDI*)Ca(THP)]_3_(P_7_) (3) and a view of the Ca_3_P_7_ core. (b) Centrosymmetric crystal structure of [(BDI*)Ca]_2_(DBA) (4). (c) Centrosymmetric crystal structure of [(BDI*)Ca(OEt_2_)]_2_(*cyclo*-P_4_) (5). In all figures the H atoms and Et_2_CH-groups have been omitted for clarity.

The crystal structure of 3 (Fig. 2a) revealed that P_4_ had been reduced to the polyphosphide P_7_^3−^ Zintl ion, encapsulated by three [(BDI*)Ca(THP)]^+^ fragments. A similar product, [(BDI)Ca]_3_(P_7_), has been obtained by Roesky and co-workers by reduction of P_4_ with [(BDI)Ca(μ_2_-H)]_2_.^19^ However, in contrast to this previous report which describes a major side-product with a ^31^P-resonance at −241.3 ppm, the reduction of P_4_ with the Ca^I^ synthon is highly selective.

Although the structure of 3 is close to being *C*_3_-symmetric, the complex does not show crystallographic symmetry. The Ca metal centres have shortest interactions to the three two-coordinate P atoms (P4–P6) in P_7_^3−^ which formally carry a negative charge (Ca–P: 3.0557(7)–3.1163(6) Å). These bonds are considerably longer than the corresponding Ca–P bonds in [(BDI)Ca]_3_(P_7_) which vary from 2.8667(9) to 2.9346(9) Å.^19^ The long Ca–P contacts in 3 are mainly due to the bulkier BDI* ligand and the additional THP coordination. While in 3 there are no Ca–P contacts to the apical P atom P7, the formally neutral three-coordinate P atoms in the P_3_-triangle (P1–P3) show long contacts (Ca–P: 3.3253(7)–3.4611(5) Å). The P–P bond lengths in the P_7_-cage (2.1601(6)–2.2610(4) Å) are in the range of previous reported Zintl ions.^37^ Reports of group 2 metal based Zintl ions are particularly rare and especially their selective formation from P_4_ remains difficult. Hill and co-workers isolated a Mg flanked P_7_ Zintl cluster that is structurally very similar to [(BDI)Ca]_3_(P_7_). However, this product could only be obtained in poor yields as a minor side product by fractional crystallization of the raw product.^38^

Whilst the ^31^P{^1^H} NMR spectrum of [(BDI*)Ca(THP)]_3_(P_7_) at room temperature showed one very broad singlet at −85.28 ppm due to fast exchange between the three different P-positions in the P_7_^3−^ anion, at −90 °C this resonance was split into seven broad but distinct signals (Fig. S22†), partially with visible magnetic coupling (ppm: −147.6, −130.4, −111.6, −75.7, −69.2, −45.1, and −38.2). This behaviour differs from other reports on the dynamics of P_7_^3−^ which generally describe splitting in only three signals in a ratio of 1 : 3 : 3 upon cooling.^19,37^ These can be assigned to the apical position, the P_3_ basal triangle and the three connecting P atoms. Our observed splitting into seven signals at low temperature can only be rationalized by a loss of trigonal symmetry by different coordination geometries at the Ca centres, making each P atom magnetically inequivalent.

The central 2e-donor X in (BDI*)Ca–X–Ca(BDI*) influences the synthon's stability and reactivity.^22−24^ In order to evaluate the effect of the central X^2−^ anion on P_4_ reduction, the most reducing Ca^I^ synthon, [(BDI*)Ca]_2_(N_2_) was converted to the corresponding arene complexes by reaction with benzene or *p*-xylene which led to N_2_ release. These binuclear arene complexes were further reacted with equimolar quantities of P_4_ in cyclohexane-*d*_12_ and conversion was monitored with ^31^P{^1^H} NMR. Similarly to P_4_ reduction with N_2_^2−^, two signals were observed: a sharp low-field resonance at 458.4 ppm and a rather broad high-field singlet at −85.3 ppm, corresponding to the P_7_^3−^ Zintl anion. Over time, the sharp low-field resonance at 458.4 ppm disappeared and clean formation of the P_7_ complex was observed. This suggests that the species with a ^31^P resonance at 458.4 ppm is an intermediate on the way to formation of the P_7_ complex. The only difference is the rate at which this happens which seems to be related to the reducing power of the central X^2−^ anion: N_2_^2−^ > *p*-xylene^2−^ > benzene^2−^. While in the case of the N_2_ complex, the intermediate species was nearly fully converted to the P_7_ complex after an hour, the less reducing benzene complex required stirring overnight (Fig. S45†). As we were not able to isolate the intermediate, we sought a weaker Ca^I^ synthon to slow the reaction further.

Reaction of P_4_ with (BDI*)Ca–(anthracene)–Ca(BDI*)^23^ gave a myriad of products of which one could be recognized as the P_7_ complex (3) by ^31^P NMR analysis (Fig. S48†). This prompted us to look further for a suitable bridging ligand. Due to aromaticity in its dianionic state, the boron-doped 9,10-dimethyl-diboraanthracene (DBA) dianion is less reducing and has markedly different electronic properties.^39–41^ Recently, first lanthanide triple-decker complexes featuring DBA^2−^ ligands were reported.^42^ Reduction of DBA with the Ca^I^ synthon [(BDI*)Ca](N_2_) in cyclohexane resulted in gas evolution and immediate precipitation of a microcrystalline orange solid (Scheme 3). ^1^H NMR showed selective formation of the target complex [(BDI*)Ca]_2_(DBA) (4) which could be isolated in 88% yield (Fig. S49†). Unlike (BDI*)Ca–(C_6_H_6_)–Ca(BDI*), which in benzene shows reductive benzene coupling (C_6_H_6_^2−^ + C_6_H_6_ → biphenyl^2−^),^43^ [(BDI*)Ca]_2_(DBA) is even at 60 °C remarkably stable in aromatic solvents. Its ^11^B NMR spectrum did not show a clear signal, even when quartz NMR tubes were used. Bright orange crystals suitable for single crystal X-ray diffraction were obtained by recrystallization from a cyclohexane/*n*-pentane mixture at room temperature.

The complex crystallized in the *P*1̄ space group with two independent [(BDI*)Ca]_2_(DBA) (4) inverse sandwich complexes in the asymmetric unit (Fig. 2b). The structure shows a planar (μ^2^-η^6^:η^6^-DBA)^2−^ ligand of which the central B_2_C_4_-ring is sandwiched between the two (BDI*)Ca^+^ fragments with Ca–ring_centre_ distances ranging from 2.3552(4) to 2.3786(4) Å. Preference for metal coordination to the central B_2_C_4_-ring was also observed in alkali metal complexes: M(THF)_*n*_–(DBA)–M(THF)_*n*_ complexes (M = Li, Na, K).^40,44^ Like in these alkali metal inverse sandwiches, the planar DBA^2−^ anion in 4 is isoelectronic to anthracene and shows in its central ring C–C bond distances of 1.466(3)–1.468(2) Å and B–C bond distances of 1.530(3)–1.541(3) Å, specifying the extended aromatic nature of this dianion. Computational investigation of 4 shows that Ca ligand bonding is highly ionic. NPA charges: (BDI*) −0.88, Ca +1.79, DBA −1.87 (Fig. S67†).

A C_6_D_6_ solution of [(BDI*)Ca]_2_(DBA) (4) and one equivalent of P_4_ was stirred overnight at room temperature resulting in a colour change from orange to bright yellow. The ^31^P{^1^H} NMR spectrum showed a sharp singlet at 453.9 ppm indicative for exclusive formation of the hitherto unidentified intermediate (Fig. S51†) whereas the high field signal at −85 ppm for P_7_^3−^ formation is missing. In addition, ^1^H, ^11^B, and ^13^C NMR confirmed the release of neutral DBA (Fig. S52–S54†). The sharp low-field singlet of this intermediate could be assigned to the four chemically equivalent phosphorus atoms of the *cyclo*-P_4_ dianion, in the form of an inverse sandwich complex. Comparing to few reports in literature, the ^31^P NMR chemical shift of the *cyclo*-P_4_^2−^ ring is sensitive to its environment: [(^DIPP^Form)_2_Sm]_2_(*cyclo*-P_4_) with *δ* = +453 ppm^45^ (^DIPP^Form = HC(N-DIPP)_2_), Cs_2_P_4_·2NH_3_ with *δ* = +348 ppm,^46^ [(NON)Sm(THF)_2_]_2_(*cyclo*-P_4_) with *δ* = +480 ppm (NON = 4,5-bis(2,6-diisopropylphenyl-anilido)-2,7-di-*tert*-butyl-9,9-dimethyl-xanthene),^47^ [(NON)Yb(THF)_2_]_2_(*cyclo*-P_4_) with *δ* = +382 ppm,^47^ and a U complex with η^2^,η^2^-bridging *cyclo*-P_4_^2−^ with *δ* = +718 ppm.^48^ Despite this considerable range in chemical shifts, all values are considerably downfield shifted, substantiating the 6π-electron aromatic character of this dianion.

Crystallization from a saturated pentane solution, layered with drops of Et_2_O, allowed for isolation and structural characterization of [(BDI*)Ca(OEt_2_)]_2_(*cyclo*-P_4_) (5) by X-ray diffraction. The crystal structure of 5 confirms the formation of an inverse sandwich complex with a bridging *cyclo*-P_4_ dianion (Fig. 2c). The 6π-electron aromatic *cyclo*-P_4_^2−^ ring bridges in η^4^,η^4^-fashion between two cationic (BDI*)Ca^+^ fragments with Ca–P_4_(centroid) bond lengths of 2.6460(5) Å and Ca–P bond lengths between 3.0446(9) and 3.0682(8) Å. The P_4_ ring is disordered over two positions which are rotated in respect to each other around the Ca⋯Ca′ axis by circa 45°. The P–P bond lengths range from 2.156(1) Å to 2.158(1) Å and internal P–P–P angles are close to 90° (P2–P1–P2′ 89.40(4)°, P1′–P2–P1 90.60(4)°). Hence, the aromatic P_4_^2−^ ring is almost perfectly square planar. The P–P bond lengths of the phosphorus ring are in the range of reported examples and seems independent of the metal atoms which sandwiches this ring (Sm: 2.144(1)–2.162(1) Å,^45^ U: 2.149(2)–2.152(2) Å,^48^ Cs: 2.146(1)–2.148(1) Å).^46^ It is of interest to note that the geometry of P_4_^2−^ depends strongly on the metals that sandwich this dianion. The ring structure of 6π-electron aromatic *cyclo*-P_4_^2−^ in the Ca inverse sandwich 5 contrasts strongly with the butterfly structure of P_4_^2−^ in the Mg inverse sandwich 2. A detailed discussion on these differences follows below (*vide infra*).

It is noteworthy, that the reduction of P_4_ with [(BDI*)Ca]_2_(DBA) (4) exclusively led to formation of the aromatic (*cyclo*-P_4_)^2−^ dianion. Even with a large excess of P_4_ and using forcing reaction conditions (60 °C), only the P_4_ complex 5 was formed. This represents the first example of a quantitative reduction of P_4_ to (*cyclo*-P_4_)^2−^ mediated by s-block metals. Treatment of P_4_ with Cs in THF followed by solvation in liquid ammonia led to the formation of Cs_3_P_7_·3NH_3_ as major product and the desired cyclotetraphosphide Cs_2_P_4_·2NH_3_ was only a by-product.^46^

In order to evaluate its role as an intermediate in polyphosphide formation, crystalline [(BDI*)Ca(OEt_2_)]_2_(*cyclo*-P_4_) (5) and one equivalent of P_4_ were suspended in cyclohexane-*d*_12_ (Scheme 3). Monitoring the reaction with ^31^P{^1^H} NMR showed after 2 hours at room temperature slow conversion of the (*cyclo*-P_4_)^2−^ complex into the (P_7_)^3−^ complex (P_4_ : P_7_ = 0.63 : 0.37). Complete, selective conversion to the P_7_ product was achieved after two days at room temperature (Fig. S55†).

Two observations need further attention. (1) The DBA complex 4 cannot be converted to a P_7_ complex and reacts with excess white phosphorous only to the P_4_ product. (2) Once crystallized in presence of ether and isolated, 5 selectively reacts with P_4_ to the P_7_ complex. Although at first sight contradicting, these combined observations can only lead to one conclusion. The neutral DBA that is released in reaction of 4 with P_4_ must be an inhibitor for the P_4_-to-P_7_ conversion.

Indeed, whereas the reaction of crystalline [(BDI*)Ca(OEt_2_)]_2_(*cyclo*-P_4_) (5) with P_4_ in cyclohexane-*d*_12_ showed slow but selective conversion to the P_7_ complex, addition of DBA to 5 inhibited P_4_-to-P_7_ conversion. With catalytic quantities of DBA as low as 5 mol% no conversion was observed, even after one day at room temperature. However, after 30 hours at 60 °C a small amount of the P_7_ complex was observed (Fig. S58†). The mechanism of this inhibitor effect are still unclear but leave room for speculation (*vide infra*).

### P_4_^2−^: butterfly or aromatic ring?

The geometry of P_4_^2−^ depends strongly on the metals that sandwich this dianion. Captured between two (BDI*)Mg^+^ cations it takes the form of a butterfly with η^2^,η^2^-bridging (2) but between (BDI*)Ca^+^ a η^4^,η^4^-bridging 6π-electron aromatic *cyclo*-P_4_^2−^ dianion (5) is favoured. These intriguing differences in geometry and coordination modes may be understood by Density Functional Theory (DFT) calculations.

The structures of 2 and ether-free 5 were optimized at the PBE0/def2-SVP level of theory. The calculated structure of 2 fits reasonably well with that from the crystal structure (Fig. S66†), indicating a sufficient level of theory. The aromaticity of 5 has been investigated before, and differs from that in classical aromatic hydrocarbons like in benzene.^49^ It can be noticed that the P–P bonds in *cyclo*-P_4_^2−^ in 5 (calculated: 2.173–2.184 Å) are not much different from the single P–P bonds in P_4_ (calculated: 2.201 Å). Electron Localization Function (ELF) analyses showed that the P–P bonds in *cyclo*-P_4_^2−^ have P–P single bond character and show no sign of the high electron delocalization as found in benzene.^49^ In contrast to the high population in C–C bonds of benzene, ELF analysis does not show a high population in the P–P bonding orbitals. Instead, a high population was found in the P lone-pairs in *cyclo*-P_4_^2−^. Hence, the concept of “lone-pair aromaticity” was defined. In contrast to benzene, for which a circle within the C_6_-ring depicts aromaticity, for *cyclo*-P_4_^2−^ a circle around the P_4_-ring has been proposed.^49^

**Fig. 3:**
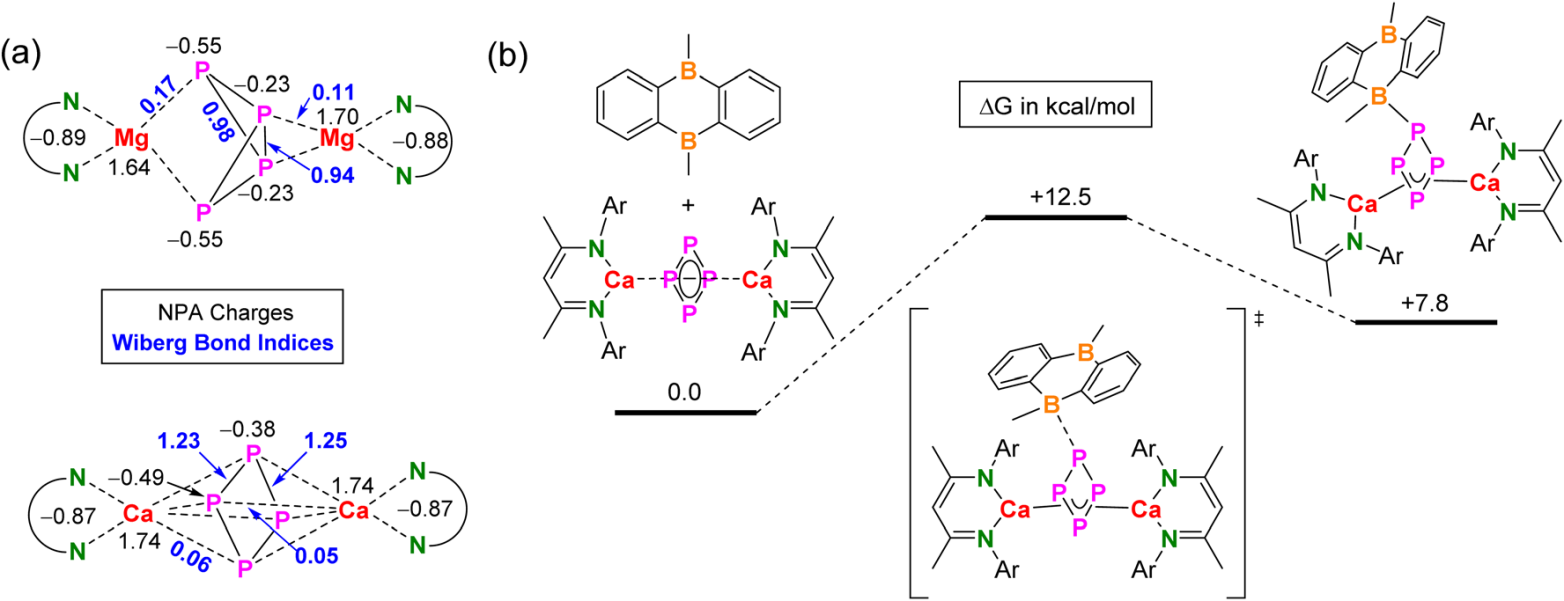
Computational studies at the PBE0-D3BJ(PCM = cyclohexane)/def2-TZVP level of theory. (a) NPA charges and WBI's for [(BDI*)Mg]_2_(P_4_) and [(BDI*)Ca]_2_(*cyclo*-P_4_). (b) Energy profile for the reaction of [(BDI*)Ca]_2_(*cyclo*-P_4_) with DBA.

The question remains why the P_4_^2−^ dianion can have two different appearances, butterfly or cyclic structure, depending on the metal that sandwich this entity. The answer may lie in the character of metal–P_4_ bonding. Based on electronegativity differences (Mg: 1.31, Ca: 1.00, P: 2.19),^50^ Mg–P_4_ bonding in 2 should be slightly more covalent than Ca–P_4_ bonding in 5. The rather low Wiberg Bond Indices (WBI's) for Mg–P_4_ bonding (0.11/0.17) and Ca–P_4_ bonding (0.05/0.06) show that both bonds have ionic character but the Mg–P_4_ bonds are somewhat more covalent (Fig. 3a). Natural Population Analysis (NPA) indeed shows a significantly higher negative charge on the *cyclo*-P_4_ unit in Ca complex 5 (−1.74) than that on butterfly P_4_ unit in Mg complex 2 (−1.56); see Fig. 3a. This corresponds to higher positive charges on the Ca cations (+1.74) compared to the Mg cations (average: +1.67). The butterfly form of P_4_^2−^ shows two different P atoms. The two-coordinate P atoms that carry most of the negative charge (−0.55) shows strong bonding to Mg and feature WBI's of 0.17. The three-coordinate P atoms with charges of −0.23 have weaker bonds to Mg (WBI: 0.11). This confirms the view that 2 can be seen as a magnesate anion (BDI*)Mg(P_4_)^−^ with a total NPA charge of −0.82 that interacts with a (BDI*)Mg^+^ cation carrying a charge of +0.82. The WBI's for the P–P bonds (0.94/0.98) are close to those for a covalent single bond.

In comparison, the NPA charges on the P atoms in *cyclo*-P_4_ are more similar (−0.38/−0.49) and intermediate to those in butterfly P_4_^2−^ (−0.23/−0.55). Charge differences are likely dictated by their different environments. Atoms-In-Molecules (AIM) analyses show that the P_4_^2−^ dianions in 2 and 5 are also involved in weak P⋯H–C bonding with organic fragments of the BDI* ligands (Fig. S72 and S74†). In agreement with more ionic character of the Ca complex, the WBI's for Ca–P_4_ bonding (0.05/0.06) are smaller than for Mg–P_4_ bonding (0.11/0.17). However, the WBI's for the P–P bonds in *cyclo*-P_4_ (1.23/1.25) are slightly larger than that expected for a single bond. This is in agreement with some extent of aromaticity (*vide supra*).

**Scheme 4:**
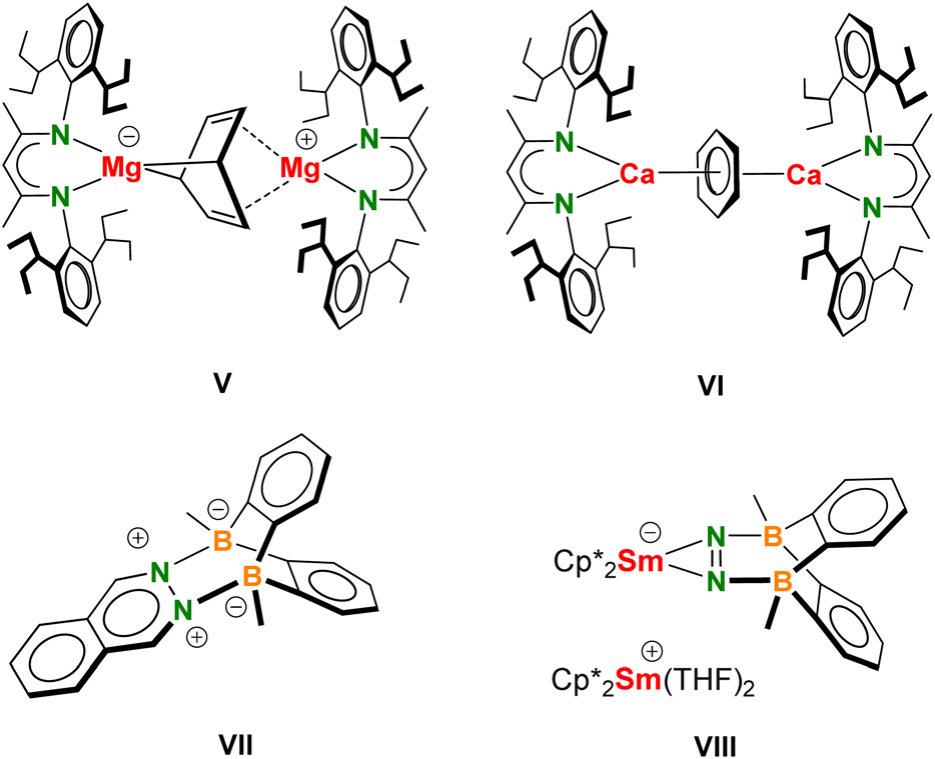
Formulas V–VIII.

The differences observed in bonding of the P_4_^2−^ dianion in Mg and Ca complexes are comparable to differences observed in bonding of the C_6_H_6_^2−^ dianion (V and VI, Scheme 4). Bonding in the Mg complex (BDI*)Mg–(C_6_H_6_)–Mg(BDI*) is more covalent than that in the corresponding Ca complex. The C_6_H_6_^2−^ in the Mg complex shows the typical boat form with strong localized Mg–C bonding to bow and stern and much weaker Mg–C interactions to the C=C bonds.^33^ Similarly as for 2, the complex can be seen as a magnesate anion (BDI*)Mg(C_6_H_6_)^−^ interacting with a (BDI*)Mg^+^ cation through unusual Mg–alkene coordination for which we recently found ample proof.^51–53^ In contrast, (BDI*)Ca–(C_6_H_6_)–Ca(BDI*) shows a nearly flat C_6_H_6_^2−^ dianion and η^6^,η^6^-bridging between the Ca^2+^ ions.^22^ These parallels between C_6_H_6_^2−^ and P_4_^2−^ bonding in Mg or Ca sandwich complexes find their origins in the more covalent nature of the Mg–ligand bond but could also be related to the considerably larger size of the Ca^2+^ cation compared to the Mg^2+^ cation. This explanation is in agreement with the occurrence of P_4_^2−^ butterfly structures in Al, Ga, Si or Ni complexes which are even more covalent in character than the Mg complex.^12,14,34–36^

### How does diboraanthracene (DBA) inhibit P_4_ to P_7_ conversion?

Whilst [(BDI*)Ca(OEt_2_)]_2_(*cyclo*-P_4_) (5) reacts selectively with P_4_ to the P_7_ complex, addition of DBA inhibited this conversion. Initially, we sought an explanation in possible interaction of DBA with the P_4_ reactant. It is known that the electrophilic diborane DBA can interact with bidentate electron-rich ligands like 1,2-diazines (*e.g.* in VII)^54^ and even assists in N_2_ fixation with Cp*_2_Sm (VIII) (Cp* = 1,2,3,4,5-penta-methylcyclopentadienyl).^55^ However, ^1^H and ^31^P NMR spectra of a mixture of DBA and P_4_ did not show any changes in the chemical shifts when compared to the pure species. This excludes significant DBA⋯P_4_ interaction. Attempts to optimize the structure of potential DBA⋯P_4_ complexes by DFT calculation also only led to separation of these molecules.

Alternatively, DBA can interact with the P_4_ complex [(BDI*)Ca]_2_(*cyclo*-P_4_), thus inhibiting further reactivity with P_4_. DFT calculations indeed show that this complex is able to interact with DBA by formation of a P–B bond (Fig. 3b; for structures see Fig. S78†). Although the activation free energy for this process is only Δ*G*^‡^_298_ = 12.5 kcal mol^−1^, the adduct is Δ*G*_298_ = 7.8 kcal mol^−1^ higher in energy than the unbound molecules. This implies a fast equilibrium that lies mainly on the side of free P_4_ and [(BDI*)Ca]_2_(*cyclo*-P_4_). This means that, although complexation of DBA with *cyclo*-P_4_^2−^ is possible, this cannot be a reason to inhibit further reactivity towards the formation of P_7_ complex. Especially, when one considers that also small, catalytic quantities of DBA already work as inhibitor.

Reaction mechanisms that give rise to higher nuclearity clusters are in general poorly understood.^37^ However, it seems reasonable to assume that reduction of P_4_ with s-block metal reducing agents starts with electron-transfer. As we could show that the P_4_^2−^ dianion is a likely intermediate en route to P_7_^3−^, the first step of this transformation could be a single electron transfer (SET) process. As DBA is a molecule with a low-lying LUMO and can be easily reduced, its mode of inhibiting the P_4_-to-P_7_ conversion may simply be a reversible electron-capture process. The high affinity of DBA for electrons inhibits SET to P_4_. Using DFT, we found that reduction of DBA is indeed much more facile than reduction of P_4_. The reaction, DBA^−^ + P_4_ → P_4_^−^ + DBA, was calculated to be highly endothermic (in cyclohexane: Δ*H* = +23.5 kcal mol^−1^). This is also in agreement with the observation that catalytic quantities of DBA function as a stabilizer for the P_4_^2−^ dianion and would explain the highly selective formation of the P_4_ complex 5.

## Conclusion

We achieved first P_4_ reductions with low-valent β-diketiminate Mg^I^ complexes and found the selectivity to be greatly dependent on the bulk of the ligand. Using a superbulky BDI ligand with DIPeP substituents (BDI*) led to highly selective formation of a butterfly-shaped P_4_^2−^ dianion bridging two (BDI*)Mg^+^ fragments in a unique η^2^,η^2^-fashion. The same complex with Ca instead of Mg features a *cyclo*-P_4_^2−^ dianion bridging (BDI*)Ca^+^ fragments in an η^4^,η^4^ mode. This difference finds its origin in the more covalent nature of the Mg–(P_4_) bond but could also be related to the considerably larger size of the Ca^2+^ cation compared to the Mg^2+^ cation, favouring a delocalized *cyclo*-P_4_^2−^ dianion.

Reaction of low-valent Ca^I^ synthons selectively gave products with the P_7_^3−^ Zintl anion: [(BDI*)Ca]_3_(P_7_). Monitoring these reactions by NMR shows first unambiguous proof that P_7_^3−^ formation proceeds through a *cyclo*-P_4_^2−^ intermediate. The kinetics of the P_4_ → *cyclo*-P_4_^2−^ → P_7_^3−^ conversion depends strongly on the reducing power of the Ca^I^ synthon: [(BDI*)Ca]_2_(X) in which X is dianionic N_2_^2−^ or arene^2−^. Conversion is faster along the row X = benzene^2−^ < *p*-xylene^2−^ < N_2_^2−^.

In case of Ca^I^ synthons, using 9,10-dimethyl-diboraanthracene (DBA) as a bridging dianion led to exclusive formation of the *cyclo*-P_4_^2−^ product. Even with excess P_4_ and forcing reaction conditions no further P_4_ → P_7_ conversion was observed in the time frame conducted. As addition of trace quantities of DBA already inhibited and considerably retarded further reactivity of the *cyclo*-P_4_^2−^ dianion, it is proposed that DBA prevents radical reactivity by functioning as a reversible electron trap.

These first investigations on P_4_ reduction with low-valent Ae metal complexes show that selectivities depend on the bulk of the BDI ligand, the metal and the presence of inhibitors for radical reactivity. We continue our research with investigations on P_4_ reduction with heavier low-valent Ae metal synthons.

## Data availability

Crystallographic data has been deposited with the Cambridge Structural Database.

## Author contributions

S. Thum: conceptualization, investigation, validation, formal analysis, writing – original draft, visualization. O. P. E. Townrow: investigation, validation, formal Analysis. J. Langer: formal analysis, validation. Sjoerd Harder: conceptualization, writing – original draft – review and editing, visualization, validation, supervision, project administration.

## Conflicts of interest

There are no conflicts to declare.

## Supplementary Material

SC-OLF-D4SC08502G-s001

SC-OLF-D4SC08502G-s002
