# Supplementary material for: Tuning the selectivity of P4 reduction at alkaline-earth metal centres
Source: Chem Sci. 2025 Feb 5;16(10):4528–36. doi: 10.1039/d4sc08502g (PMC11808274; doi:10.1039/d4sc08502g)
Supplement: SC-016-D4SC08502G-s001 [file SC-016-D4SC08502G-s001.pdf]

Supplementary Information (SI) for Chemical Science.
This journal is © The Royal Society of Chemistry 2025

Supporting Information

Table of Contents

1. Materials and Methods S2
2. Synthetic Procedures....S3
3. Spectroscopic Characterization S7
4. Selected NMR Spectra S26
5. Crystal Structure Determinations....S39
6. Computational Details....S48
7. References S94

1. Materials and Methods

All experiments were conducted in dry glassware under an inert nitrogen or argon atmosphere by applying standard Schlenk techniques or gloveboxes (MBraun) using freshly dried and degassed solvents. Benzene, cyclohexane, diethylether, hexanes, methylcyclohexane, and pentanes were degassed with nitrogen, dried over a column with activated aluminum oxide (Innovative Technology, Pure Solv 400-4-MD, Solvent Purification System) and then stored under inert atmosphere over molecular sieves (3 Å). Tetrahydropyran (THP) and *p*-xylene were dried over freshly grounded CaH_2, distilled and stored over molecular sieves (3 Å) under inert atmosphere. Deuterated benzene (C_6D_6), cyclohexane (C_6D_{12}), methylcyclohexane (C_7D_{14}), and toluene (C_7D_8) were purchased either from Deutero GmbH or Sigma Aldrich, degassed and dried over molecular sieves (3 Å) and stored under an inert atmosphere. The following compounds were prepared according to literature procedures: 9,10-dimethyl-diboraanthracene (DBA);[S1] Na/NaCl;[S2] P_4;[S3] $[(BDI^*)Ca]_2(N_2)$;[S4] $[(BDI^*)Ca]_2$(benzene);[S4] $[(BDI^*)Ca]_2$(*p*-xylene);[S4] $[(BDI^*)Ca]_2$(anthracene);[S5] $[(BDI)Mg]_2$;[S2] (BDI*)MgI;[S6] (BDI = HC[C(Me)-N(DIPP)]$_2$, BDI* = HC[C(Me)-N(DIPeP)]$_2$, DIPeP = 2,6-(Et_2CH)-phenyl, DIPP = 2,6-di*iso*propylophenyl).

NMR spectra were measured on Bruker Avance III H 400 MHz and Bruker Avance III HD 600 MHz NMR spectrometers. Chemical shifts (δ) are denoted in ppm (parts per million) and coupling constants in Hz (Hertz). ^1H and ^{13}C NMR spectra were referenced to the solvent residual signal ($SiMe_4$ = 0 ppm). Signal multiplicities are described using common abbreviations: s (singlet), d (doublet), t (triplet), q (quartet), quint (quintet), m (multiplet) and br (broad). Elemental analysis was performed with a Hekatech Eurovector EA3000 analyzer. All crystal structures have been measured on a SuperNova (Agilent) diffractometer with dual Cu and Mo microfocus sources and an Atlas S2 detector.

2. Synthetic Procedures

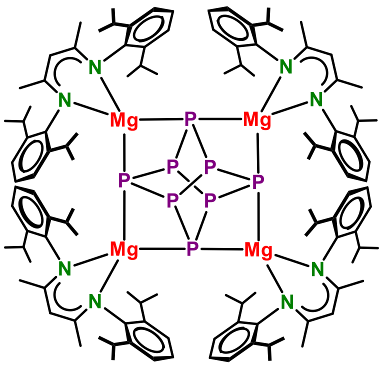

Synthesis of [(BDI)Mg]$_4$(P$_8$) (1) [(^{DIPP}BDI)Mg]$_2$ (79.9 mg, 90.4 µmol, 1.00 eq.) and P_4 (11.2 mg, 90.4 µmol, 1.00 eq.) were suspended in C_6D_6 (700 µL) and heated at 60 °C for 3 days. Single crystals suitable for X-ray diffraction analysis were grown at this temperature. The crystals were decanted off and washed with pentane (2 x 1 mL) and dried *in vacuo*. The mother liquor was concentrated and heated at 60 °C for a second crop of crystals (overall yield: 18 mg, 8.93 µmol, 10%).

^{1}H NMR (600.13 MHz, C_6D_6, 298K): δ = 1.12 (d, J = 6.7 Hz, 12H, C**H$_3$**), 1.19 (d, J = 6.7 Hz, 12H, C**H$_3$**), 1.21–1.22 (m, 12H, C**H$_3$**), 1.46 (s, 12H, C**H$_3$**-backbone), 1.48–1.49 (m, 12H, C**H$_3$**), 1.52 (s, 12H, C**H$_3$**-backbone), 3.08–3.12 (m, 16H, C**H**), 4.69 (s, 4H, C**H**-backbone), 7.19–7.21 (m, 10H, C**H**-arom.), 7.24–7.25 (m, 8H, C**H**-arom.), 7.27–7.29 (m, 6H, C**H**-arom.) ppm.

^{13}C NMR (150.92 MHz, C_6D_6, 298K): δ = 24.8 (**C**H$_3$-backbone), 25.0 (**C**H$_3$), 25.2 (**C**H$_3$-backbone), 25.7 (**C**H$_3$), 27.3 (**C**H$_3$), 28.8 (**C**H), 28.9 (**C**H), 96.1 (**C**H-backbone), 124.2 (**C**-arom.), 124.4 (**C**-arom.), 125.9 (**C**-arom.), 126.2 (**C**-arom.), 142.9 (**C**-arom.), 143.5 (**C**-arom.), 145.7 (**C**-arom.), 146.3 (**C**-arom.), 169.7 (**C**N-backbone), 170.8 (**C**N-backbone) ppm.

^{31}P{^{1}H} NMR (242.92 MHz, C_6D_6, 298K): δ = 68.33 (br.), 144.98 (br.) ppm.

Elemental analysis Calculated for $C_{116}H_{164}Mg_4N_8P_8$ (MW = 2015.65 g/mol): C 69.12, H 8.20, N 5.56; Found: C 69.09, H 7.96, N 5.98.

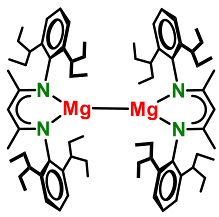

Improved synthesis of [(BDI*)Mg]$_2$ (BDI*)MgI (224 mg, 329 µmol, 1.00 eq.) and Na/NaCl (5w/w%, 755 mg, 1.64 mmol, 5.00 eq.) were suspended in benzene (7 mL) and the reaction mixture was vigorously stirred at room temperature for 15 h. The reaction mixture was filtered and evaporated to dryness. The residue was stripped with hexanes (2 mL) and dried *in vacuo* yielding [(BDI*)Mg]$_2$ as yellow powder in almost quantitative yield (176 mg, 317 µmol, 96%). The spectroscopic data are in accordance with those reported in literature.[S6]

Synthesis of [(BDI*)Mg]$_2$(P$_4$) (2) [(BDI*)Mg]$_2$ (48.0 mg, 43.3 µmol, 1.00 eq.) and P$_4$ (5.2 mg, 42.4 µmol, 0.98 eq.) were dissolved in in C_6D_6 (600 µL) and stirred at room temperature overnight. The reaction mixture was evaporated to dryness and stripped with hexanes (1 mL). The yellow powder was dissolved in pentane (1 mL), filtered, layered with a few drops of Et_2O. Single crystals suitable for X-ray diffraction analysis were obtained by storing the solution at –28 °C. The crystals were decanted off, washed with cold pentane (–28 °C, 2 x 1 mL) and dried *in vacuo* yielding [(BDI*)Mg]$_2$(P$_4$) (18.1 mg, 14.7 µmol, 34%).

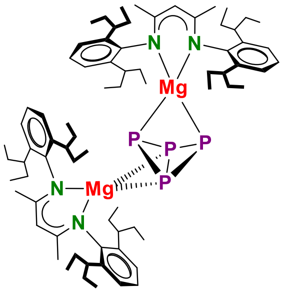

^{1}H NMR (600.13 MHz, C_6D_6, 298K): δ = 0.82 (t, J = 7.3 Hz, 12H, C**H$_3$**), 0.88 (t, J = 7.4 Hz, 12H, C**H$_3$**), 1.01 (t, J = 7.3 Hz, 12H, C**H$_3$**), 1.05 (t, J = 7.3 Hz, 12H, C**H$_3$**), 1.56–1.66 (m, 10H, C**H$_2$**), 1.67 (s, 6H, C**H$_3$**-backbone), 1.68–1.73 (m, 12H, C**H$_2$**), 1.75 (s, 6H, C**H$_3$**-backbone), 1.81–1.85 (m, 10H, C**H$_2$**), 2.76–2.80 (m, 4H, C**H**), 3.12–3.16 (m, 4H, C**H**), 4.86 (s, 1H, C**H**-backbone), 4.86 (s, 1H, C**H**-backbone), 6.97–6.98 (m, 4H, C**H**-arom.), 7.03–7.05 (m, 4H, C**H**-arom.), 7.17–7.20 (m, 4H, C**H**-arom.) ppm.

^{13}C NMR (150.92 MHz, C_6D_6, 298K): δ = 11.0 (**C**H$_3$), 11.3 (**C**H$_3$), 12.6 (**C**H$_3$), 12.9 (**C**H$_3$), 24.2 (**C**H$_3$-backbone), 24.4 (**C**H$_3$-backbone), 25.1 (**C**H$_2$), 25.5 (**C**H$_2$), 27.7 (**C**H$_2$), 28.2 (**C**H$_2$), 41.8 (**C**H), 42.3 (**C**H), 94.2 (**C**H-backbone), 95.1 (**C**H-backbone), 124.2 (**C**-arom.), 124.9 (**C**-arom.), 125.7 (**C**-arom.), 139.3 (**C**-arom.), 140.3 (**C**-arom.), 145.4 (**C**-arom.), 146.4 (**C**-arom.), 168.2 (**C**N-backbone), 170.4 (**C**N-backbone) ppm.

^{31}P{^{1}H} NMR (242.92 MHz, C_6D_6, 298K): δ = –331.54 (t, J = 198.5 Hz), 260.22 (t, J = 199.9 Hz) ppm.

Elemental analysis Calculated for $C_{74}H_{114}MgN_2P_4$ (MW = 1232.26 g/mol): C 72.13, H 9.33, N 4.55; Found C 72.26, H 9.42, N 4.75.

Synthesis of [(BDI*)Ca(THP)]$_3$(P$_7$) (3) P$_4$ (7.6 mg, 61.3 µmol, 1.00 eq.) was suspended in C_7D_{14} (600 µL) and stirred for 6 h at room temperature. [(BDI*)Ca]$_2$(N$_2$) (71.6 mg, 61.3 µmol, 1.00 eq.) was then added and the reaction mixture was stirred for another 4 h at room temperature. The orange solution was concentrated to approximately 150 µL, filtered, layered with pentane (400 µL) and drops of THP and cooled to –28 °C. The obtained crystals were decanted off, washed with cold pentane (–28 °C, 2 x 1 mL) and dried *in vacuo*. The mother liquor was further concentrated to obtain a second crop of crystals (overall yield: 38 mg, 17.4 µmol, 28%).

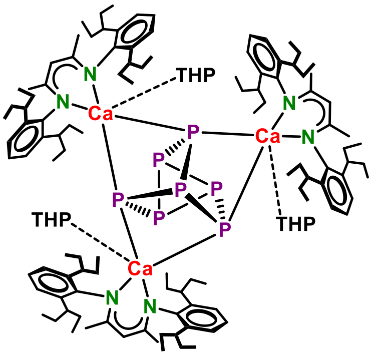

^{1}H NMR (600.13 MHz, C_6D_6, 298K): δ = 0.94–0.95 (br. m, 36H, C**H$_3$**), 1.17–1.24 (br. m, 36H, C**H$_3$**), 1.24–1.28 (m, 18H, THP β-,γ-C**H$_2$**), 1.68 (s, 18H, C**H$_3$**-backbone), 1.68–1.81 (m, 48H, C**H$_2$**), 2.59 (m, 12H, C**H**), 3.52 (m, 12H, THP, α-C**H$_2$**), 4.75 (s, 3H, C**H**-backbone), 7.16 (s, 18H, C**H**-arom.) ppm.

^{13}C NMR (150.92 MHz, C_6D_6, 298K): δ = 11.2 (**C**H$_3$), 14.2 (**C**H$_3$), 23.7 (THP γ-**C**H$_2$), 25.2 (**C**H$_2$), 26.4 (**C**H$_3$-backbone), 26.7 (THP β-**C**H$_2$), 29.2 (**C**H$_2$), 42.2 (**C**H), 68.9 (THP α-**C**H$_2$), 94.8 (**C**H-backbone), 123.5 (**C**-arom.), 125.8 (**C**-arom.), 138.4 (**C**-arom.), 166.0 (**C**N-backbone) ppm.

^{31}P{^{1}H} NMR (242.92 MHz, C_6D_6, 298K): δ = –85.28 (br.) ppm.

^{31}P{^{1}H} NMR (161.97 MHz, C_7D_8, 183K): δ = –147.63 (br.), –130.44 (br.), –111.55 (br.), –75.42 (br.), –69.22 (br.), –45.07 (br.), –38.22 (br.) ppm.

Elemental analysis Calculated for $C_{126}H_{201}Ca_3N_6O_3P_7 \cdot C_5H_{12}$ (M = 2257.23 g/mol): C 69.71, H 9.51, N 3.72; Found: C 70.28, H 9.44, N 4.23.

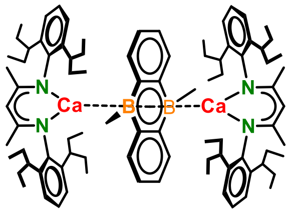

Synthesis of [(BDI*)Ca]$_2$(DBA) (4) [(BDI*)Ca]$_2$(N$_2$) (82.9 mg, 71.0 µmol, 2.00 eq.) and DBA (7.2 mg, 35.5 µmol, 1.00 eq.) were dissolved in C_6H_{12} (800 µL). Upon stirring for 5 min at room temperature, [(BDI*)Ca]$_2$(DBA) started to precipitate. The reaction mixture was stored at room temperature overnight. The microcrystalline solid was decanted off and washed with cold pentane (–28 °C, 3 x 1 mL) and dried *in vacuo*. Single crystals suitable for X-ray diffraction analysis were obtained by layering the mother liquor with the washing solution at room temperature (overall yield: 42 mg, 31.2 µmol, 88%).

^{1}H NMR (600.13 MHz, C_6D_{12}, 298K): δ = 0.53 (s, 6H, B-C**H$_3$**), 0.59 (t, J = 7.3 Hz, 24H, C**H$_3$**), 0.96 (t, J = 7.3 Hz, 24H, C**H$_3$**), 1.13 (s, 12H, C**H$_3$**-backbone), 1.23–1.33 (m, 16H, C**H$_2$**), 1.41–1.52 (m, 16H, C**H$_2$**), 1.96 (m, 8H, C**H**), 4.25 (s, 2H, C**H**-backbone), 6.03–6.06 (m, 4H, DBA-**H**), 6.91–6.95 (m, 12H, C**H**-arom.), 7.54–7.57 (m, 4H, DBA-**H**) ppm.

^{13}C NMR (150.92 MHz, C_6D_{12}, 298K): δ = 10.9 (**C**H$_3$), 12.7 (**C**H$_3$), 23.8 (**C**H$_2$), 25.0 (DBA-**C**H$_3$), 26.9 (**C**H$_3$-backbone) 28.3 (**C**H$_2$), 45.5 (**C**H), 93.9 (**C**H-backbone), 121.8 (DBA-**C**H), 123.7 (**C**-arom.), 126.6 (**C**-arom.), 135.2 (DBA-**C**H), 136.2 (DBA-**C**H), 139.3 (**C**-arom.), 148.4 (**C**-arom.), 166.7 (**C**N-backbone) ppm.

^{11}B NMR (192.55 MHz, C_6D_{12}, 298K): No signal observed.

Elemental analysis Calculated for $C_{88}H_{128}B_2Ca_2N_4 \cdot C_6H_{12}$ (M = 1427.96 g/mol): C 79.07, H 9.88, N 3.92; Found: C 79.39, H 9.45, N 4.43.

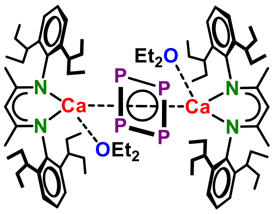

Synthesis of [(BDI*)Ca(OEt$_2$)]$_2$(*cyclo*-P$_4$) (5) [(BDI*)Ca]$_2$(DBA) (36.7 mg, 27.3 µmol, 1.00 eq.) and P_4 (3.4 mg, 27.3 µmol, 1.00 eq.) were suspended in a J. Young tube in C_6D_6 (510 µL) and stirred overnight at room temperature. The dark yellow solution was evaporated to dryness. Single crystals suitable for X-ray diffraction analysis were obtained by dissolving the crude solid in pentane (1 mL), filtering, layering the yellow solution with a few drops of Et_2O and cooled to –28 °C. The crystals were decanted off, washed with cold pentane (–28 °C, 2 x 1 mL) and dried *in vacuo* yielding [(BDI*)Ca(OEt$_2$)]$_2$(*cyclo*-P$_4$) (12 mg, 8.5 µmol, 31%).

^{1}H NMR (600.13 MHz, C_6D_{12}, 298K): δ = 0.78 (t, J = 7.4 Hz, 24H, C**H$_3$**), 0.86 (t, J = 7.3 Hz, 24H, C**H$_3$**), 1.12 (t, J = 7.0 Hz, 12H, OCH$_2$C**H$_3$**), 1.55 (s, 12H, C**H$_3$**-backbone), 1.56–1.58 (m, 10H, C**H$_2$**), 1.61–1.67 (m, 22H, C**H$_2$**), 2.54–2.58 (m, 8H, C**H**), 3.36 (q, J = 7.0 Hz, OC**H$_2$**), 4.57 (s, 2H, C**H**-backbone), 6.92 (s, 12H, C**H**-arom.) ppm.

^{13}C NMR (150.92 MHz, C_6D_{12}, 298K): δ = 11.3 (**C**H$_3$), 12.2 (**C**H$_3$), 15.7 (OCH$_2$**C**H$_3$), 23.9 (**C**H$_3$-backbone), 25.5 (**C**H$_2$), 28.7 (**C**H$_2$), 42.6 (**C**H), 66.3 (O**C**H$_2$CH$_3$), 94.7 (**C**H-backbone), 124.1 (**C**-arom.), 126.2 (**C**-arom.), 138.9 (**C**-arom.), 147.0 (**C**-arom.), 165.9 (**C**N-backbone) ppm.

^{31}P{^{1}H} NMR (242.92 MHz, C_6D_{12}, 298K): δ = 457.73 (s) ppm.

Elemental analysis Calculated for $C_{82}H_{134}Ca_2N_4O_2P_4$ (M = 1412.05 g/mol): C 69.75, H 9.57, N 3.97; Found: C 70.33, H 9.00, N 4.41.

3. Spectroscopic Characterization

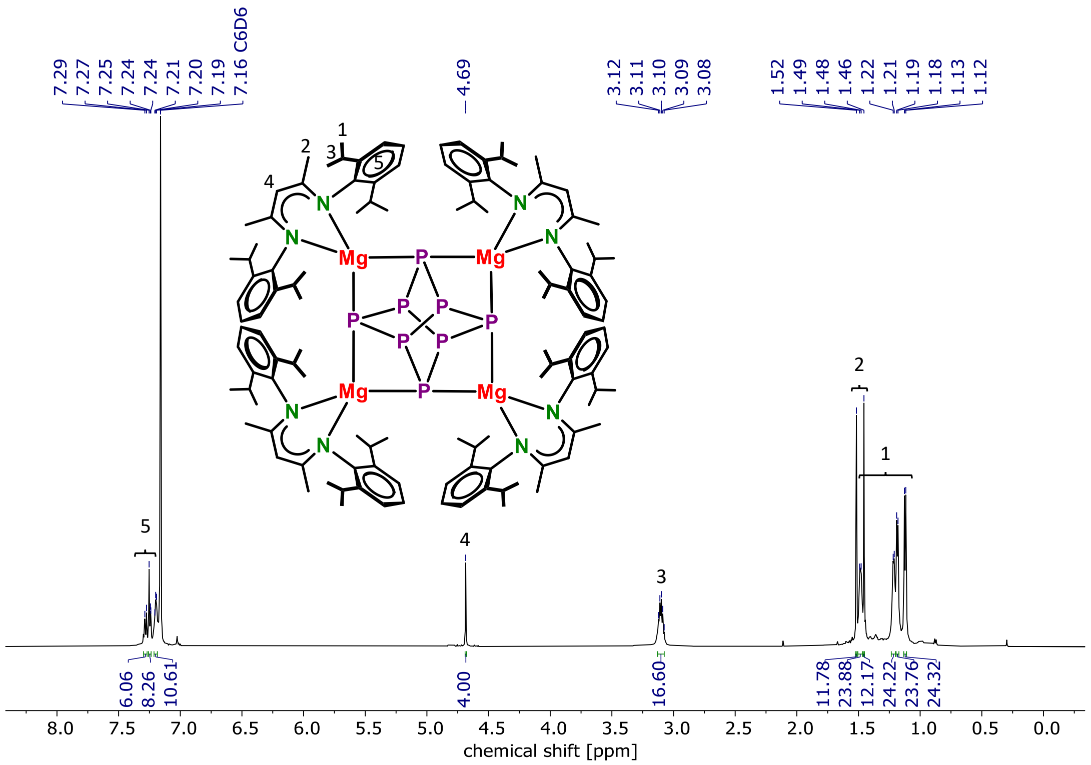

Figure S1. ^{1}H NMR (600.13 MHz, C_6D_6, 298K) of $[(BDI)Mg]_4(P_8)$ (**1**).

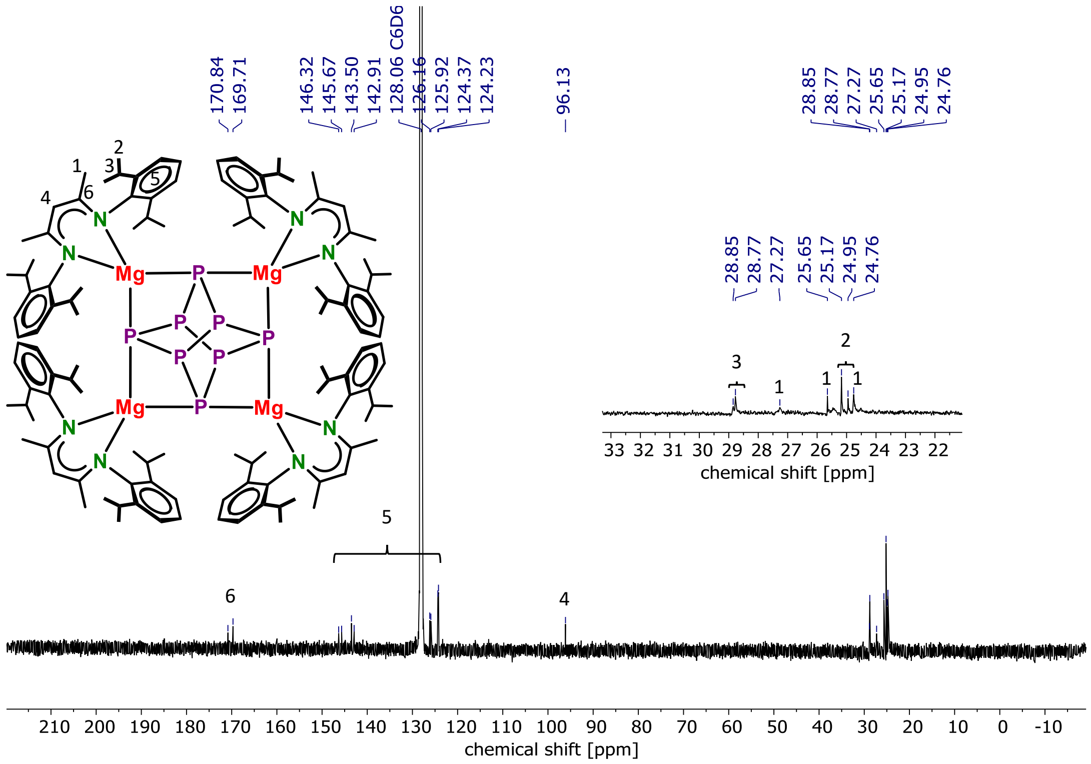

Figure S2. ^{13}C NMR (150.92 MHz, C_6D_6, 298K) of $[(BDI)Mg]_4(P_8)$ (**1**).

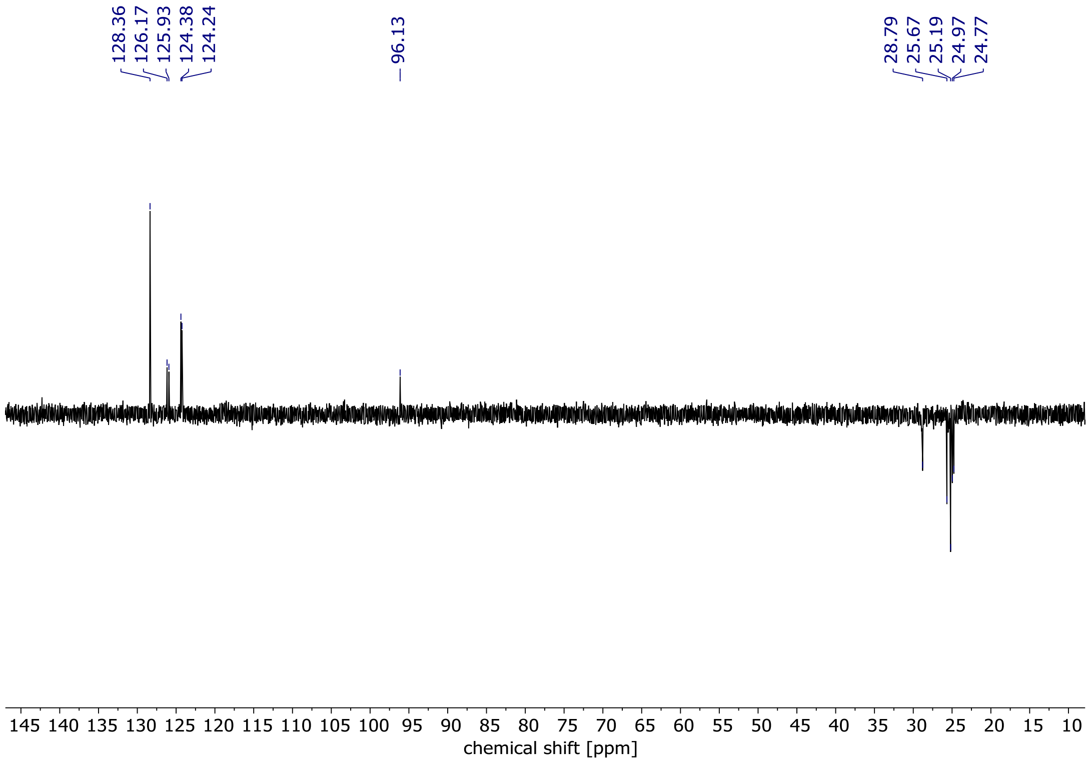

Figure S3. ^{13}C(DEPT 135) NMR (150.92 MHz, C_6D_6, 298K) of $[(BDI)Mg]_4(P_8)$ (**1**).

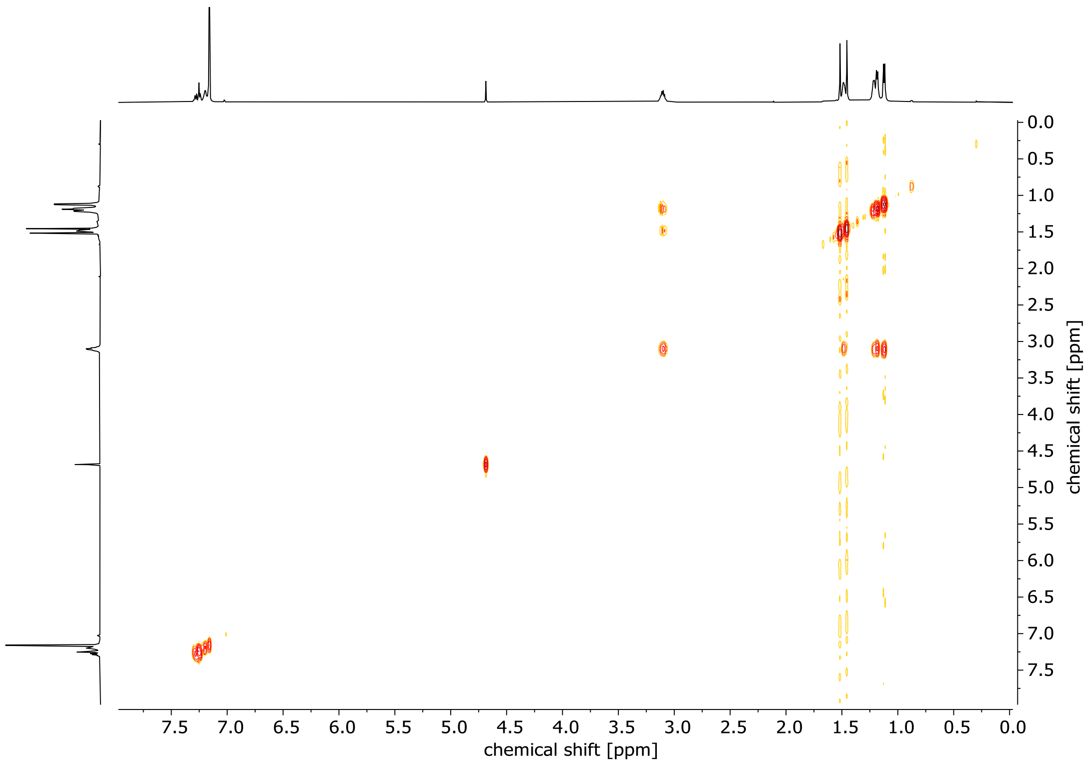

Figure S4. ^1H-^1H COSY NMR (600.13 MHz, C_6D_6, 298K) of $[(BDI)Mg]_4(P_8)$ (**1**).

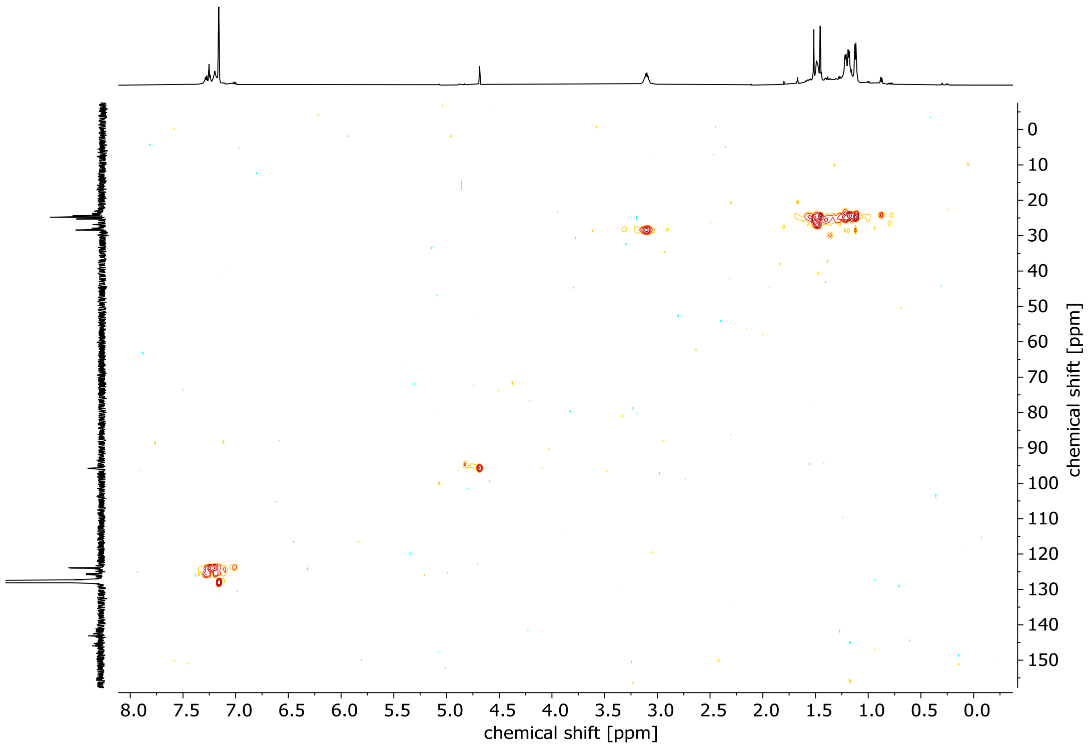

Figure S5. ^{1}H-^{13}C HSQC NMR (600.13/150.91 MHz, C_6D_6, 298K) of $[(BDI)Mg]_4(P_8)$ (**1**).

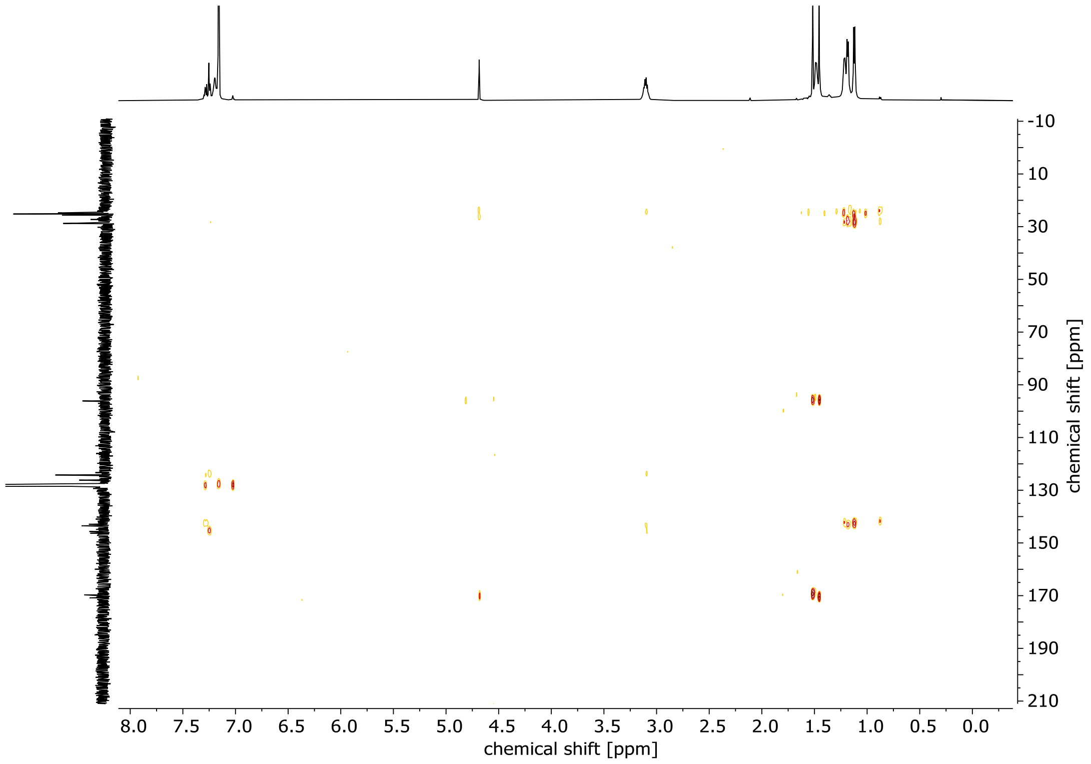

Figure S6. ^{1}H-^{13}C HMBC NMR (600.13/150.91 MHz, C_6D_6, 298K) of $[(BDI)Mg]_4(P_8)$ (**1**).

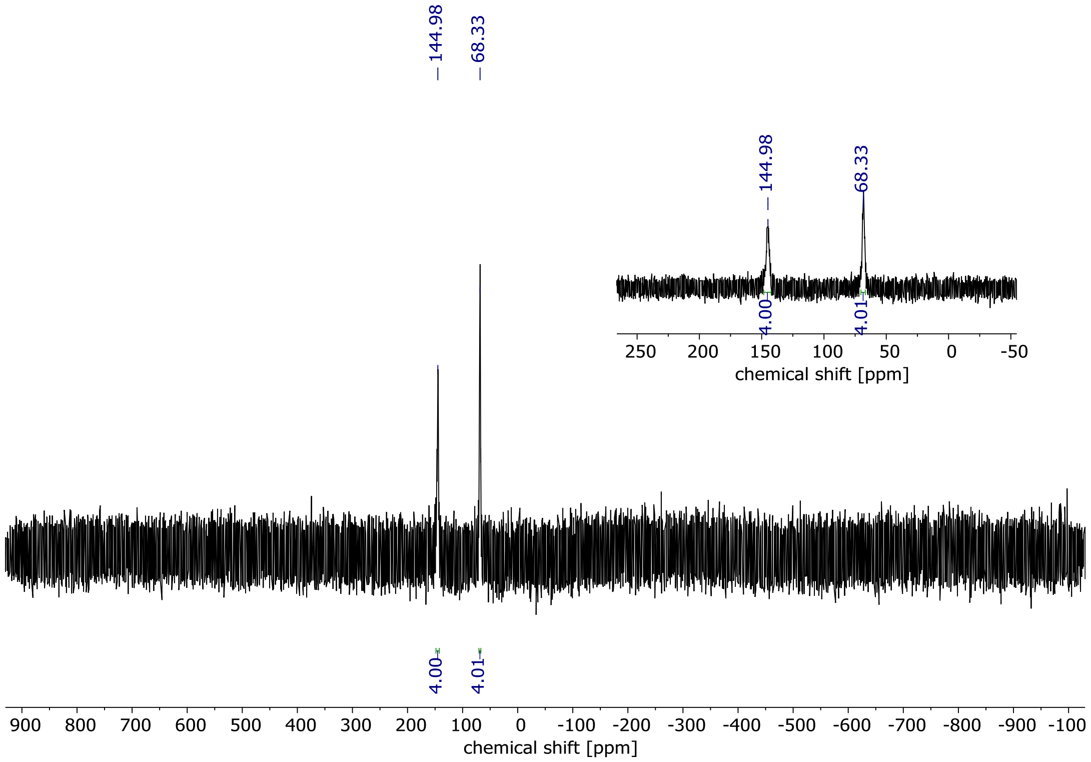

Figure S7. $^{31}P\{^{1}H\}$ NMR (242.92 MHz, C_6D_6, 298K) of $[(BDI)Mg]_4(P_8)$ (**1**).

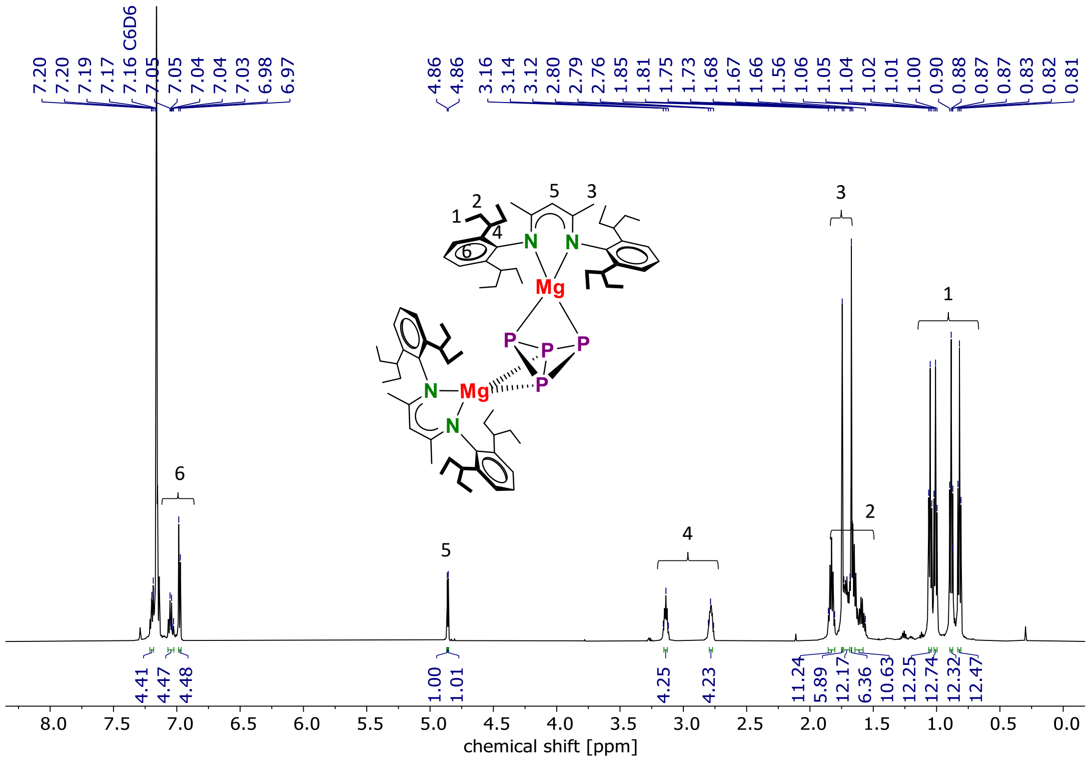

Figure S8. ^{1}H NMR (600.13 MHz, C_6D_6, 298K) of $[(BDI^*)Mg]_2(P_4)$ (**2**).

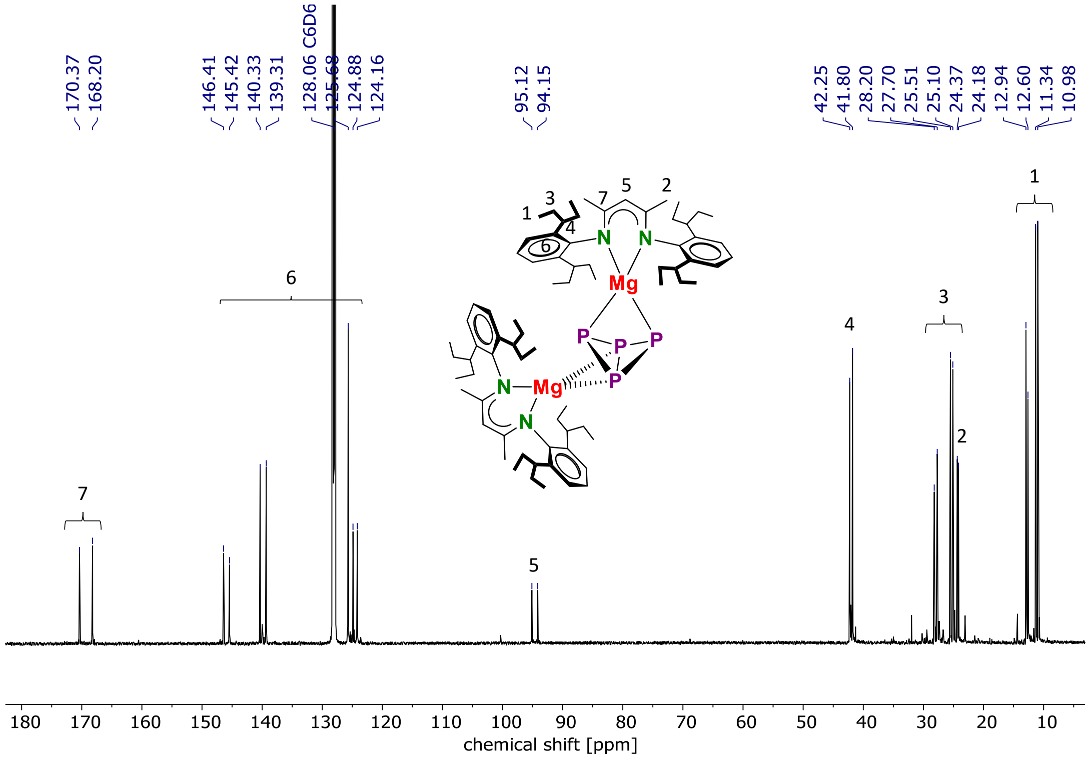

Figure S9. ^{13}C NMR (150.92 MHz, C_6D_6, 298K) of [(BDI*)Mg]$_2$(P_4) (**2**).

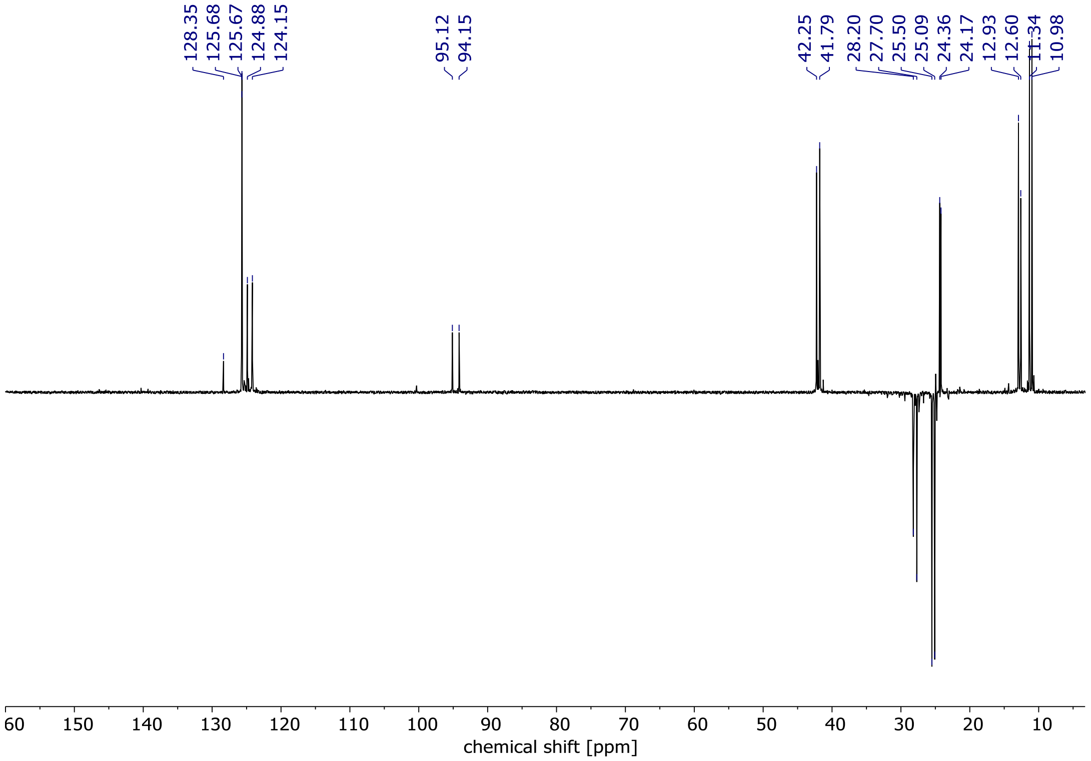

Figure S10. ^{13}C(DEPT 135) NMR (150.92 MHz, C_6D_6, 298K) of [(BDI*)Mg]$_2$(P_4) (**2**).

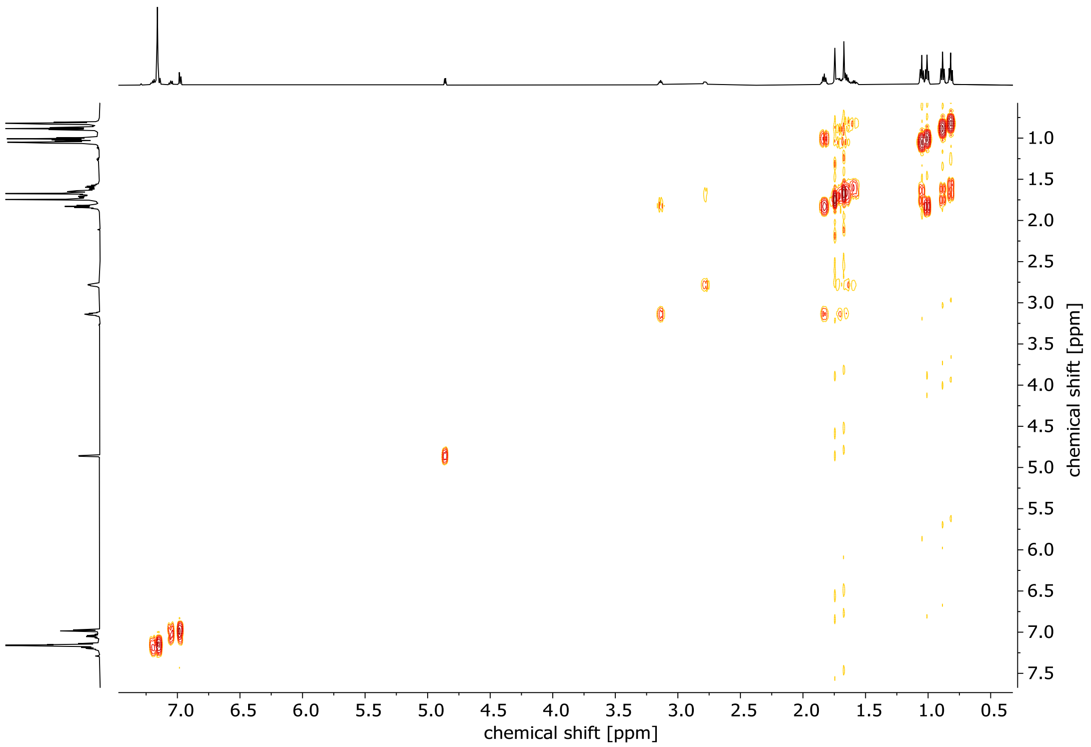

Figure S11. ^{1}H-^{1}H COSY NMR (600.13 MHz, C_6D_6, 298K) of [(BDI*)Mg]$_2$(P_4) (**2**).

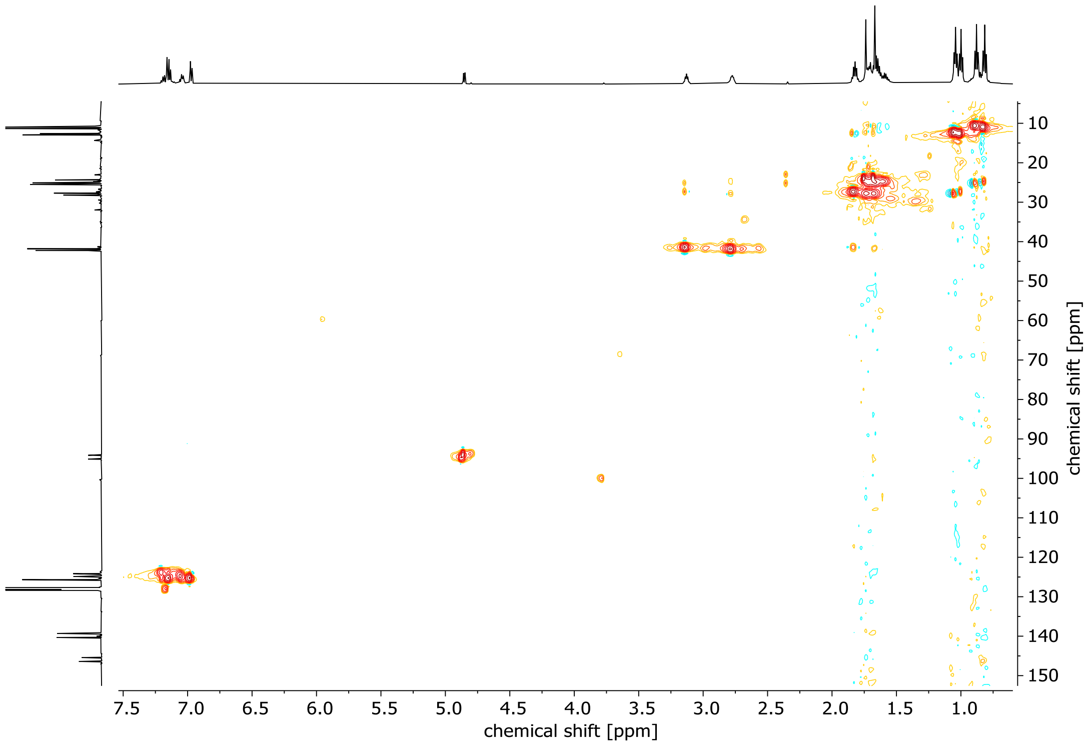

Figure S12. ^{1}H-^{13}C HSQC NMR (600.13/150.91 MHz, C_6D_6, 298K) of [(BDI*)Mg]$_2$(P_4) (**2**).

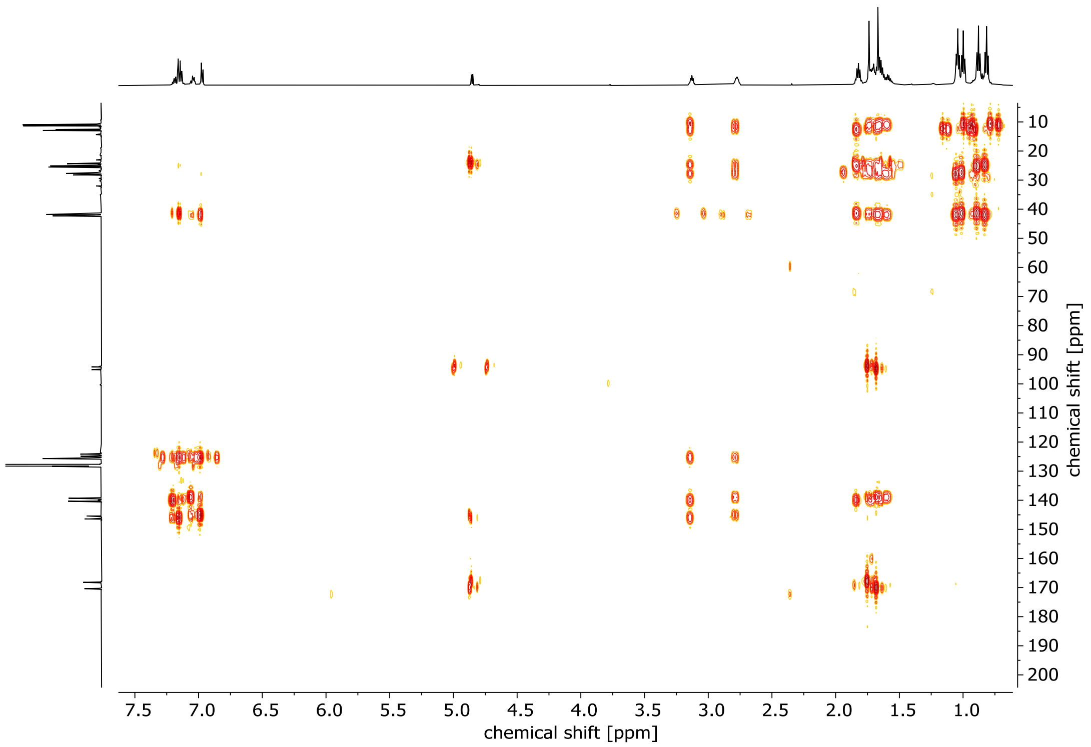

Figure S13. ^{1}H-^{13}C HMBC NMR (600.13/150.91 MHz, C_6D_6, 298K) of [(BDI*)Mg]$_2$(P_4) (**2**).

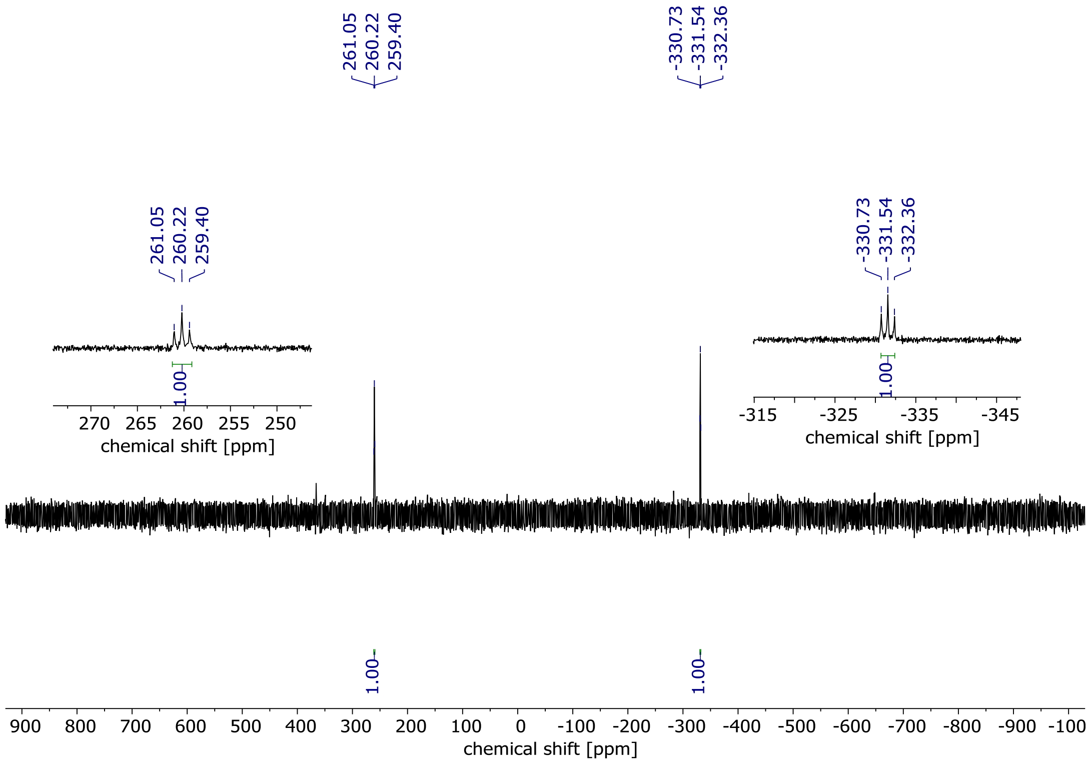

Figure S14. $^{31}P\{^{1}H\}$ NMR (242.92 MHz, C_6D_6, 298K) of [(BDI*)Mg]$_2$(P_4) (**2**).

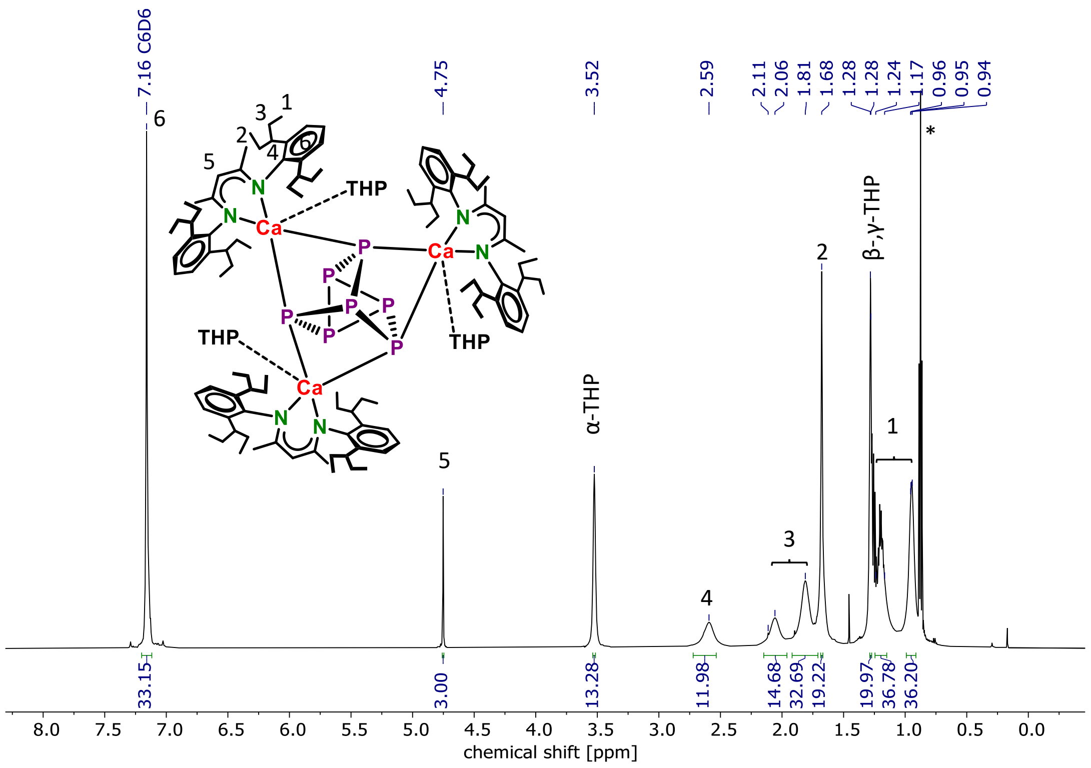

Figure S15. ^{1}H NMR (600.13 MHz, C_6D_6, 298K) of $[(BDI^*)Ca(THP)]_3(P_7)$ (**3**). Co-crystallized pentane is marked with asterisks.

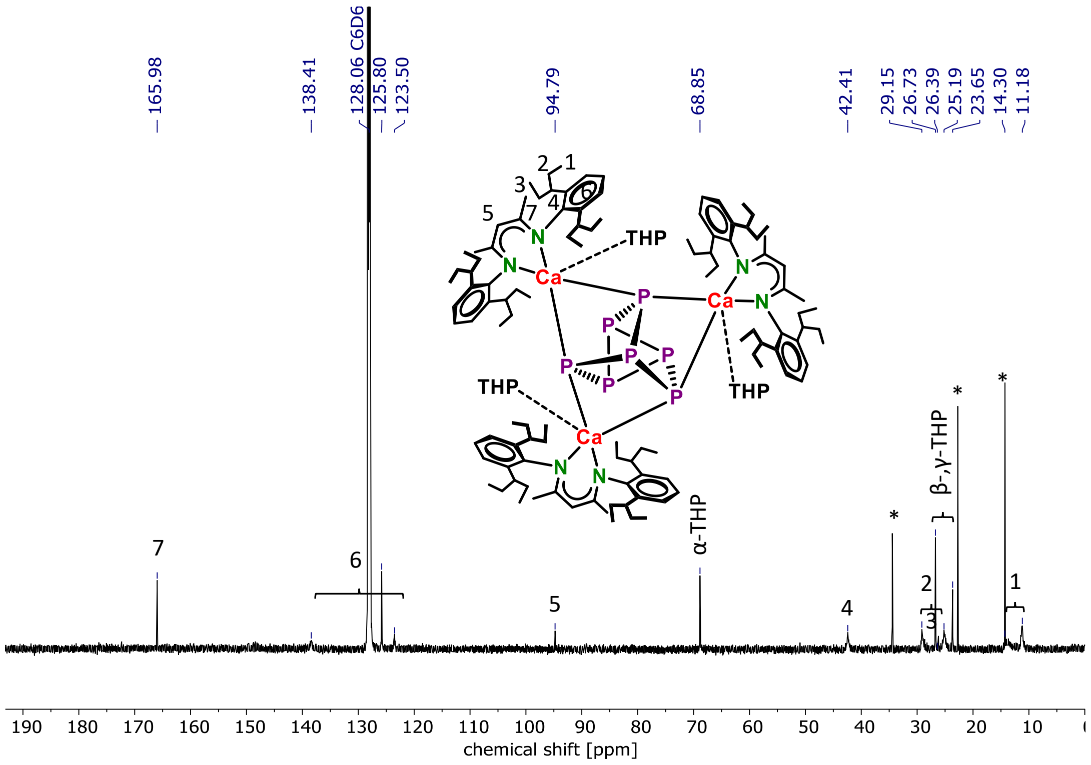

Figure S16. ^{13}C NMR (150.92 MHz, C_6D_6, 298K) of $[(BDI^*)Ca(THP)]_3(P_7)$ (**3**). Co-crystallized pentane is marked with asterisks.

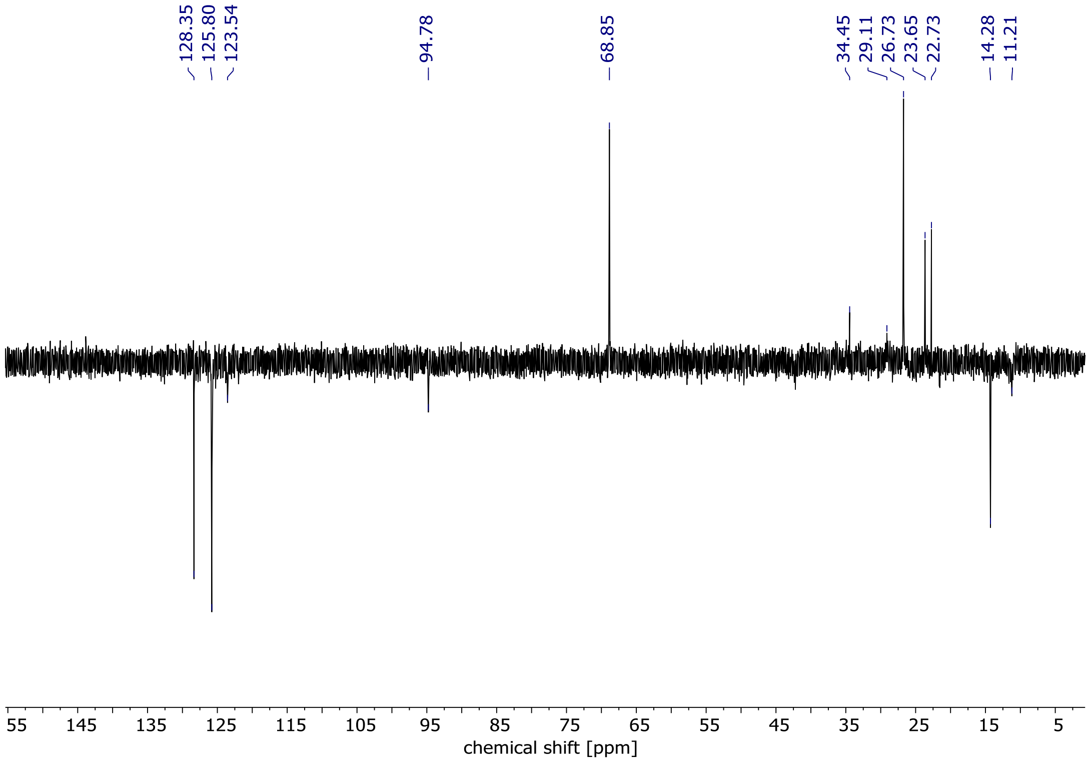

Figure S17. ^{13}C(DEPT 135) NMR (150.92 MHz, C_6D_6, 298K) of [(BDI*)Ca(THP)]$_3$(P_7) (**3**).

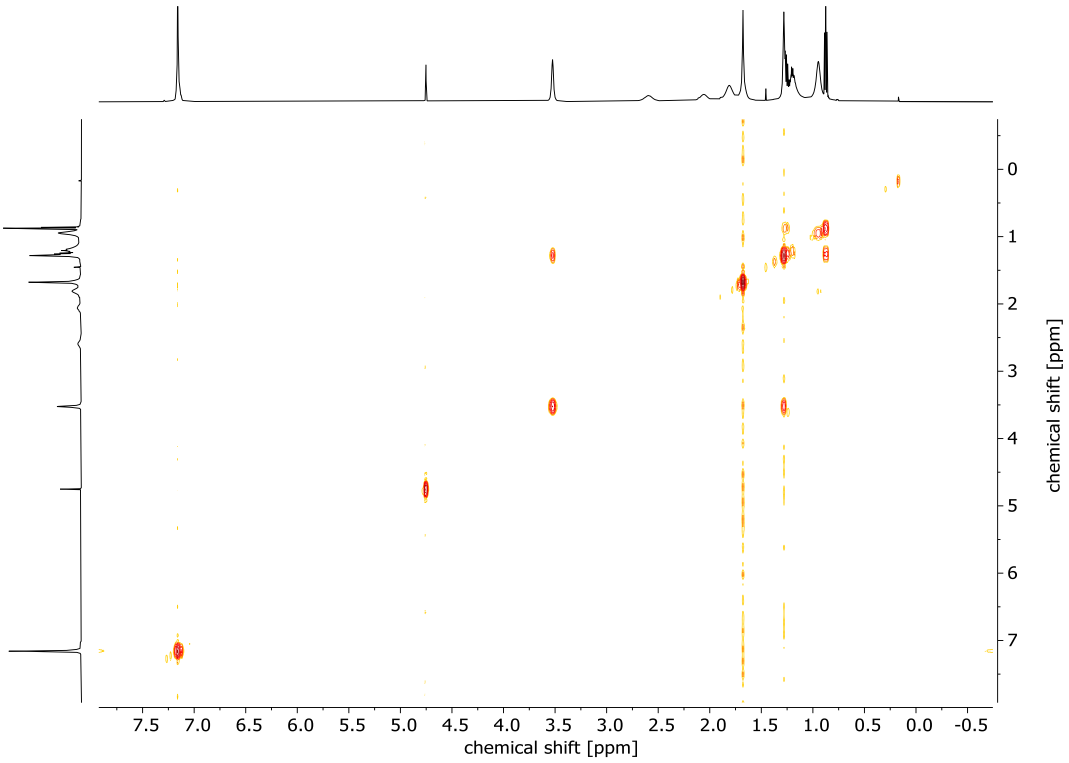

Figure S18. ^{1}H-^{1}H COSY NMR (600.13 MHz, C_6D_6, 298K) of [(BDI*)Ca(THP)]$_3$(P_7) (**3**).

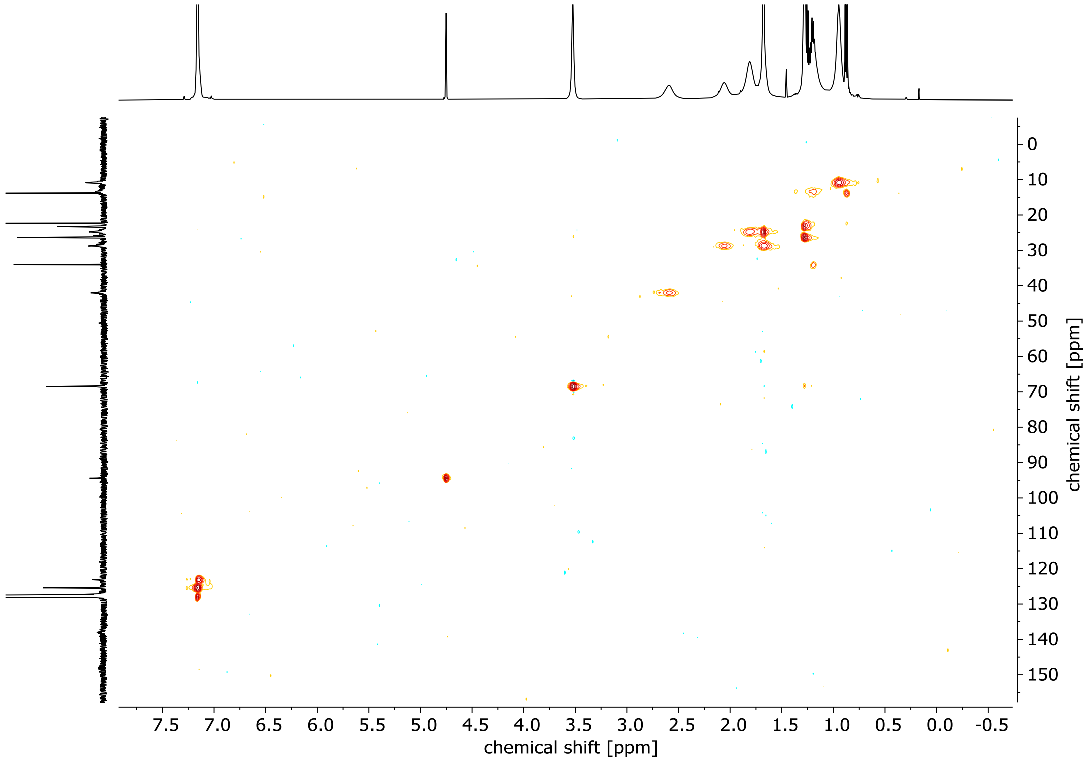

Figure S19. ^{1}H-^{13}C HSQC NMR (600.13/150.91 MHz, C_6D_6, 298K) of [(BDI*)Ca(THP)]$_3$(P_7) (**3**).

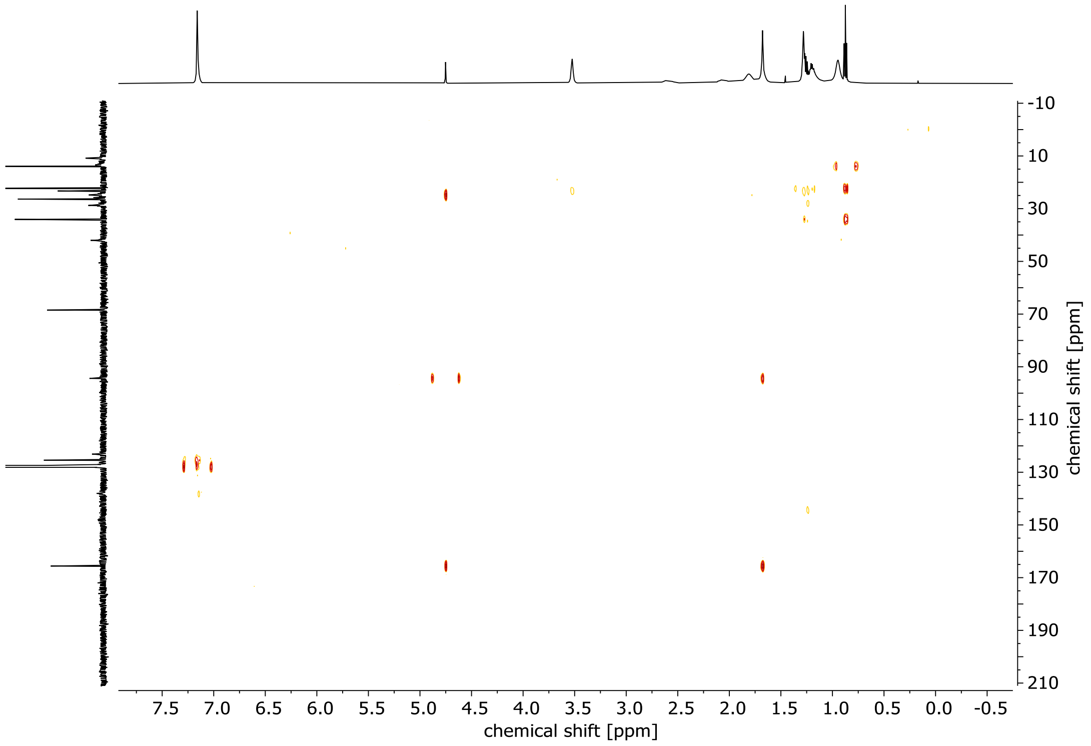

Figure S20. ^{1}H-^{13}C HMBC NMR (600.13/150.91 MHz, C_6D_6, 298K) of [(BDI*)Ca(THP)]$_3$(P_7) (**3**).

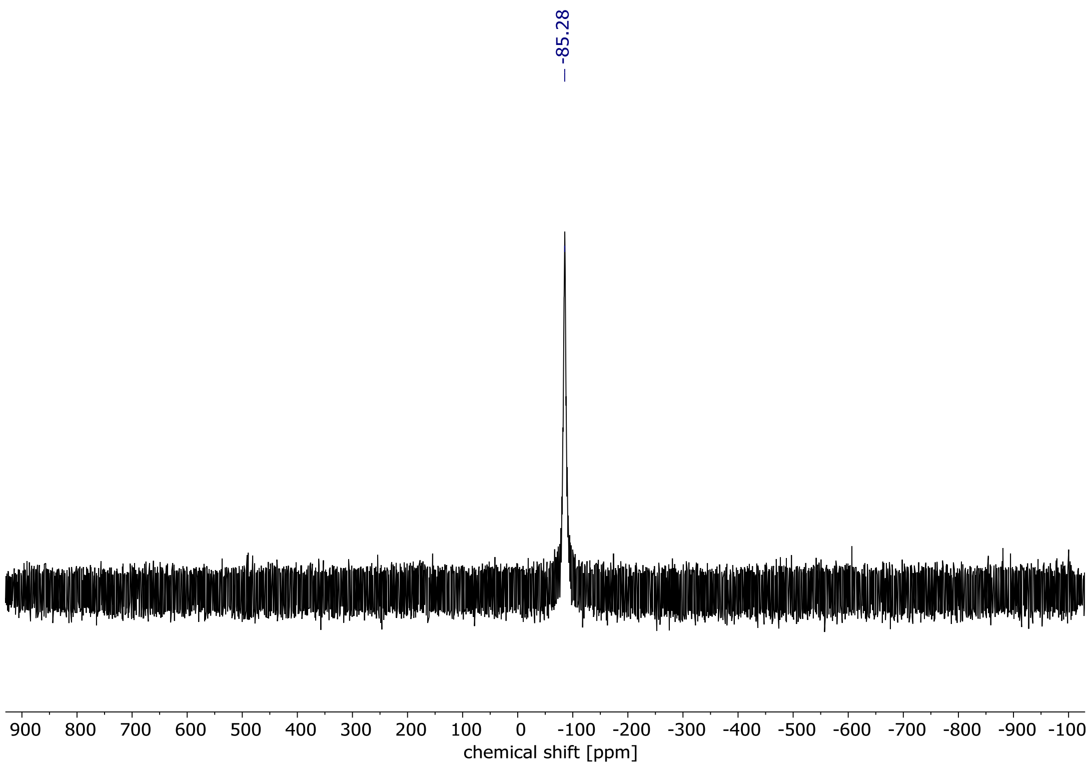

Figure S21. $^{31}P\{^1H\}$ NMR (242.92 MHz, C_6D_6, 298K) of [(BDI*)Ca(THP)]$_3$(P_7) (**3**).

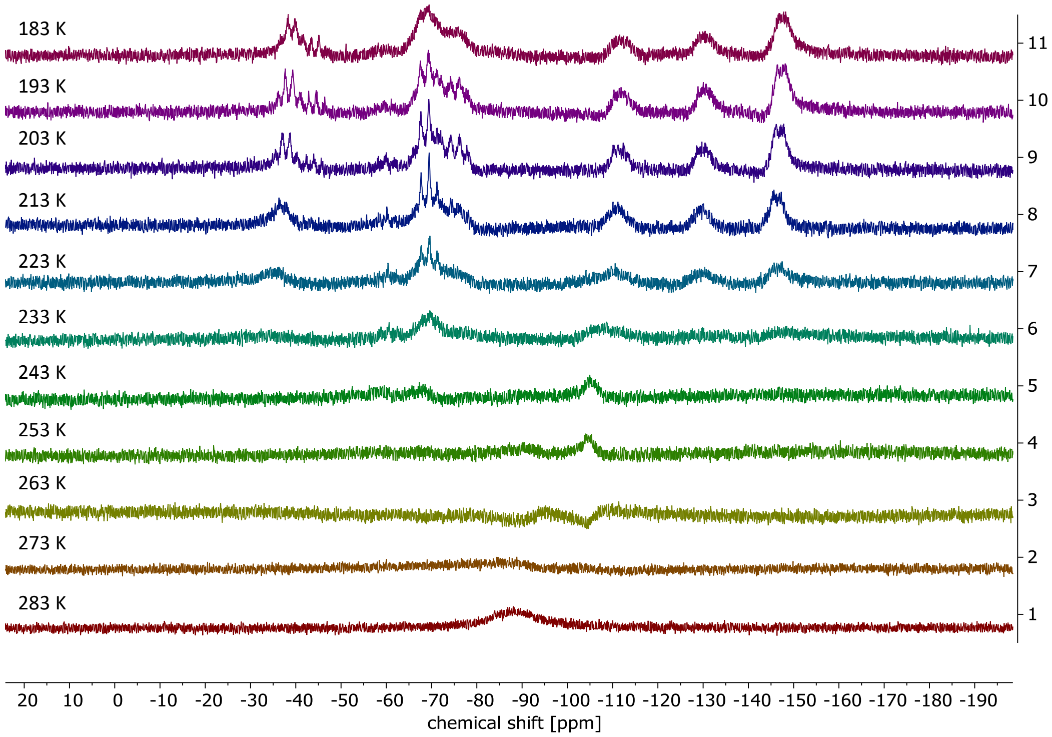

Figure S22. Variable temperature $^{31}P\{^1H\}$ NMR (161.97 MHz, C_7D_8) of [(BDI*)Ca(THP)]$_3$(P_7) (**3**).

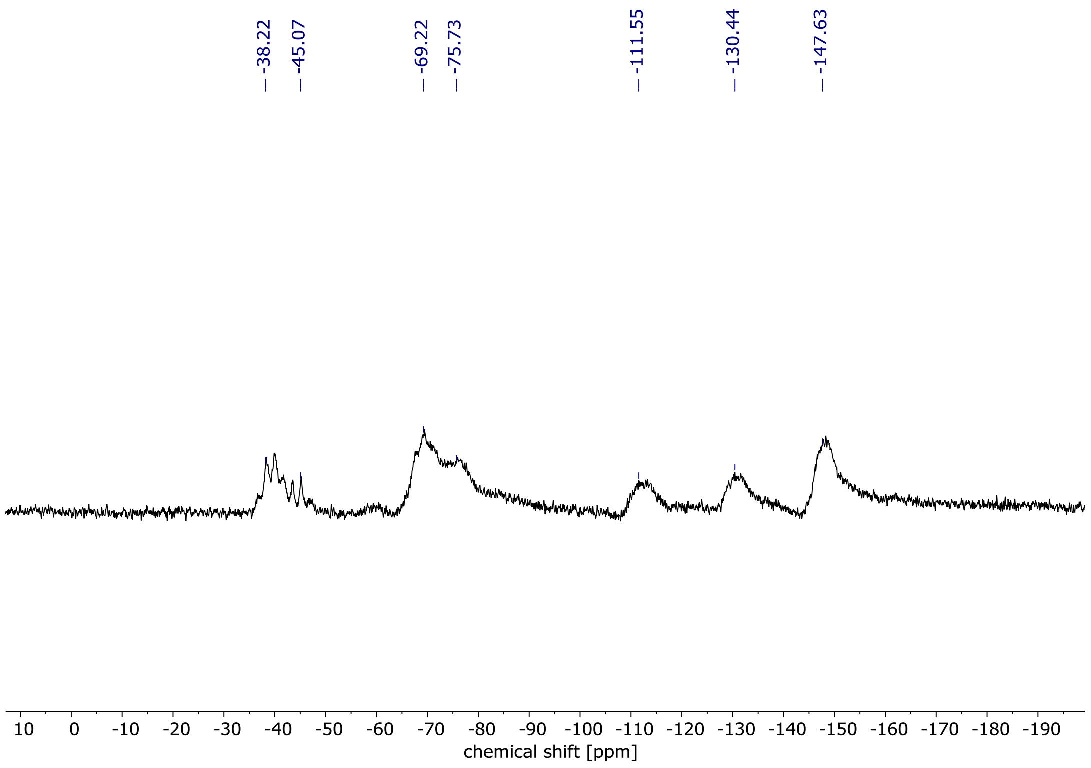

Figure S23. $^{31}P\{^{1}H\}$ NMR (161.97 MHz, C_7D_8, 183K) of $[(BDI^*)Ca(THP)]_3(P_7)$ (**3**).

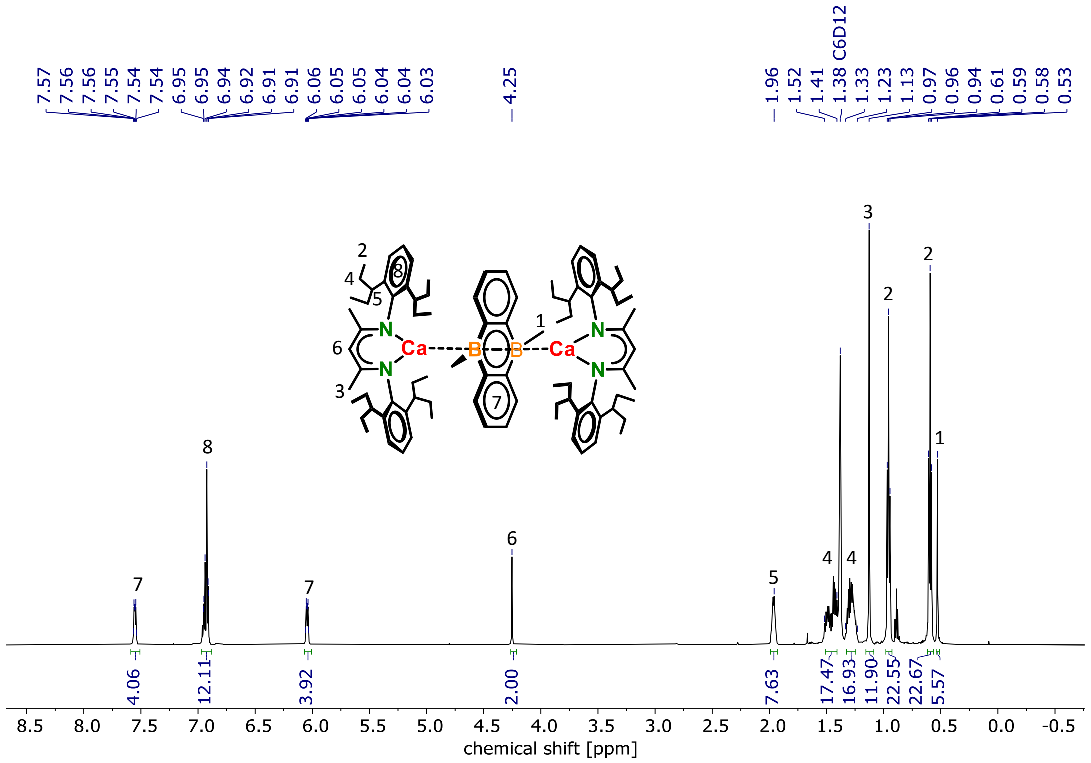

Figure S24. ^{1}H NMR (600.13 MHz, C_6D_{12}, 298K) of $[(BDI^*)Ca]_2(DBA)$ (**4**).

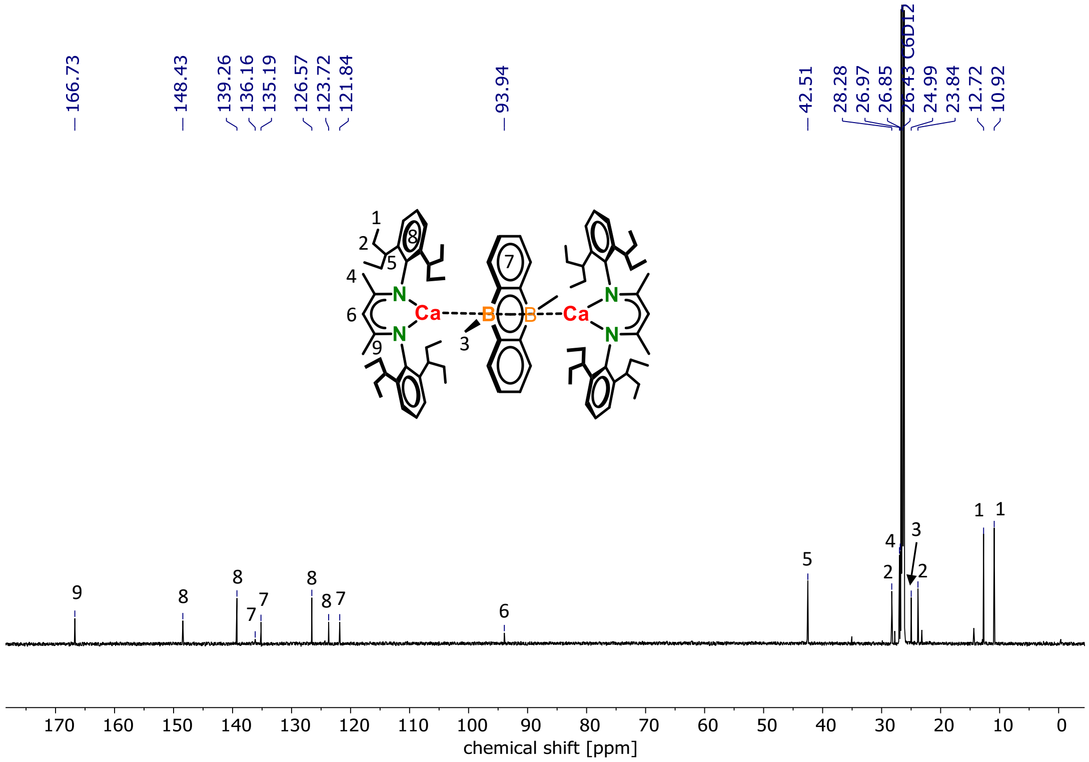

Figure S25. ^{13}C NMR (150.92 MHz, C_6D_{12}, 298K) of [(BDI*)Ca]$_2$(DBA) (**4**).

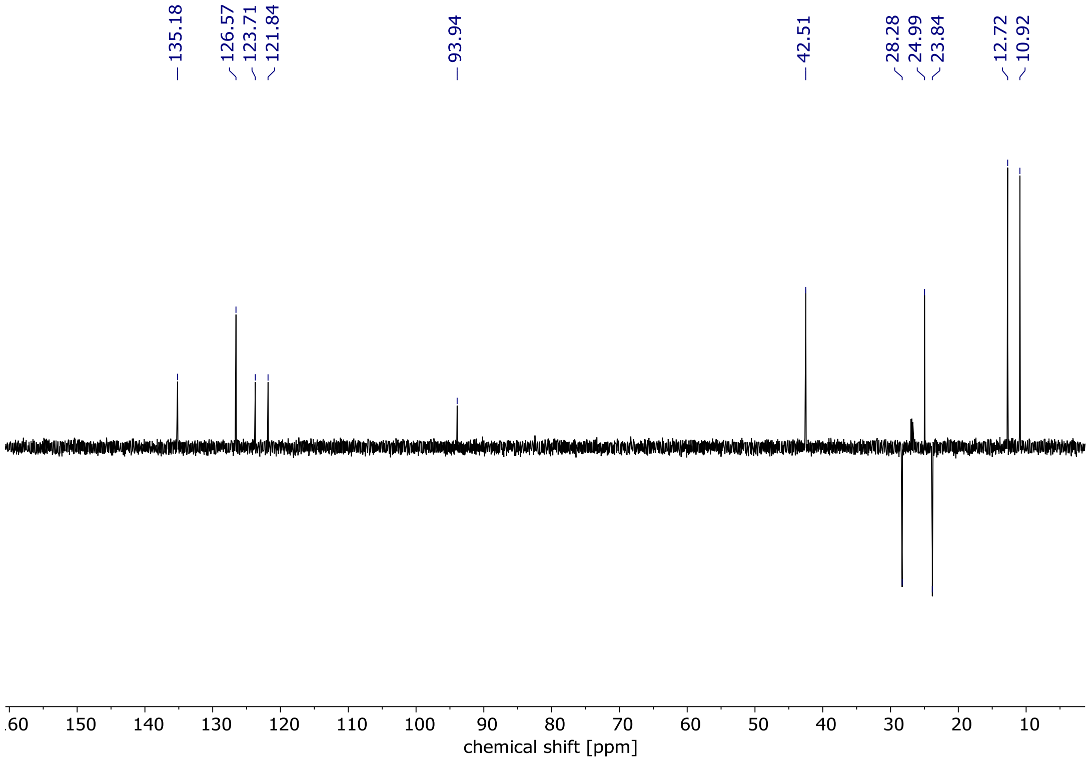

Figure S26. ^{13}C(DEPT 135) NMR (150.92 MHz, C_6D_{12}, 298K) of [(BDI*)Ca]$_2$(DBA) (**4**).

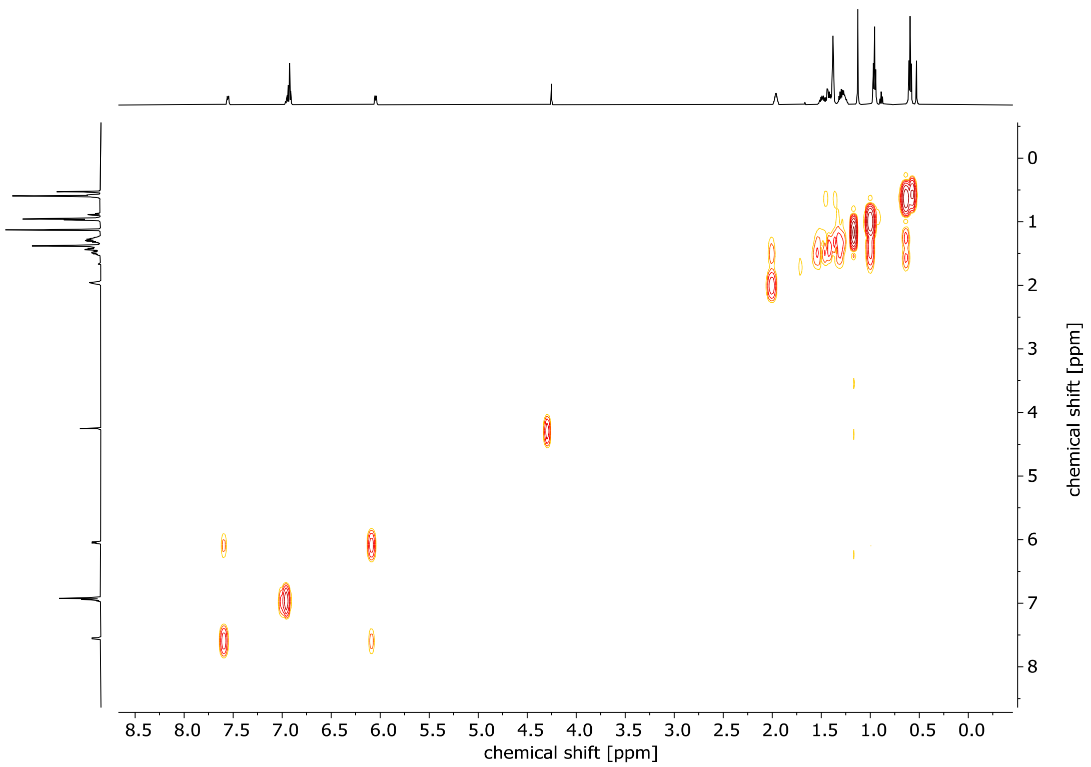

Figure S27. ^{1}H-^{1}H COSY NMR (600.13 MHz, C_6D_{12}, 298K) of [(BDI*)Ca]$_2$(DBA) (**4**).

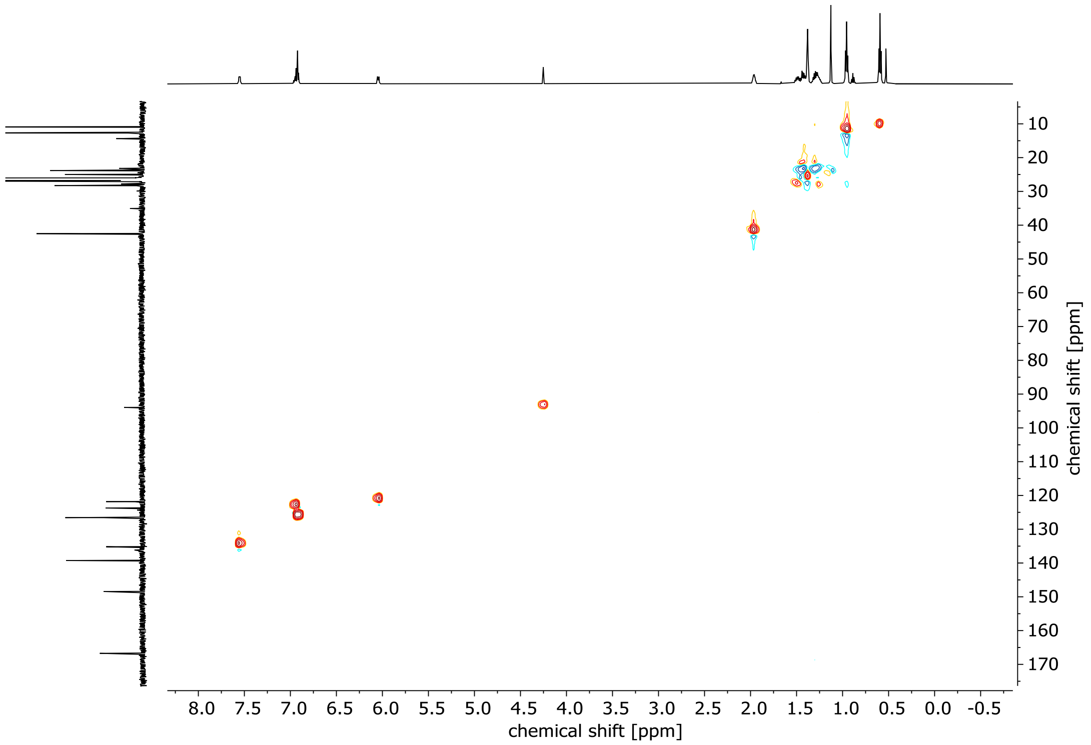

Figure S28. ^{1}H-^{13}C HSQC NMR (600.13/150.91 MHz, C_6D_{12}, 298K) of [(BDI*)Ca]$_2$(DBA) (**4**).

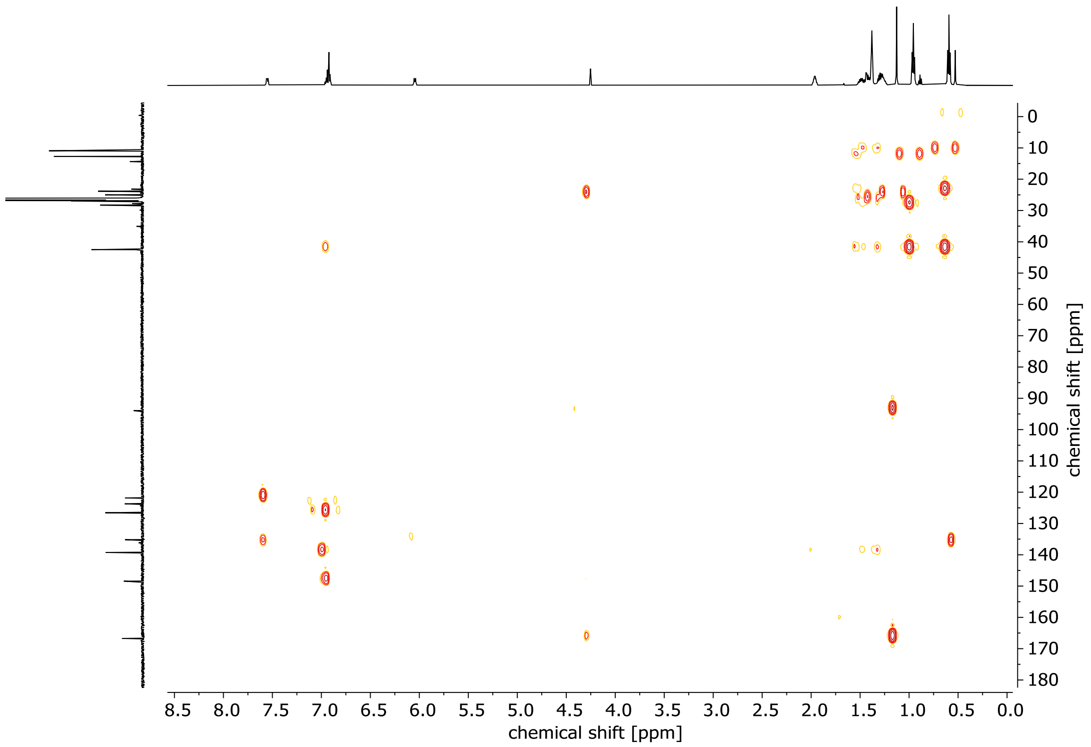

Figure S29. ^{1}H-^{13}C HMBC NMR (600.13/150.91 MHz, C_6D_{12}, 298K) of [(BDI*)Ca]$_2$(DBA) (**4**).

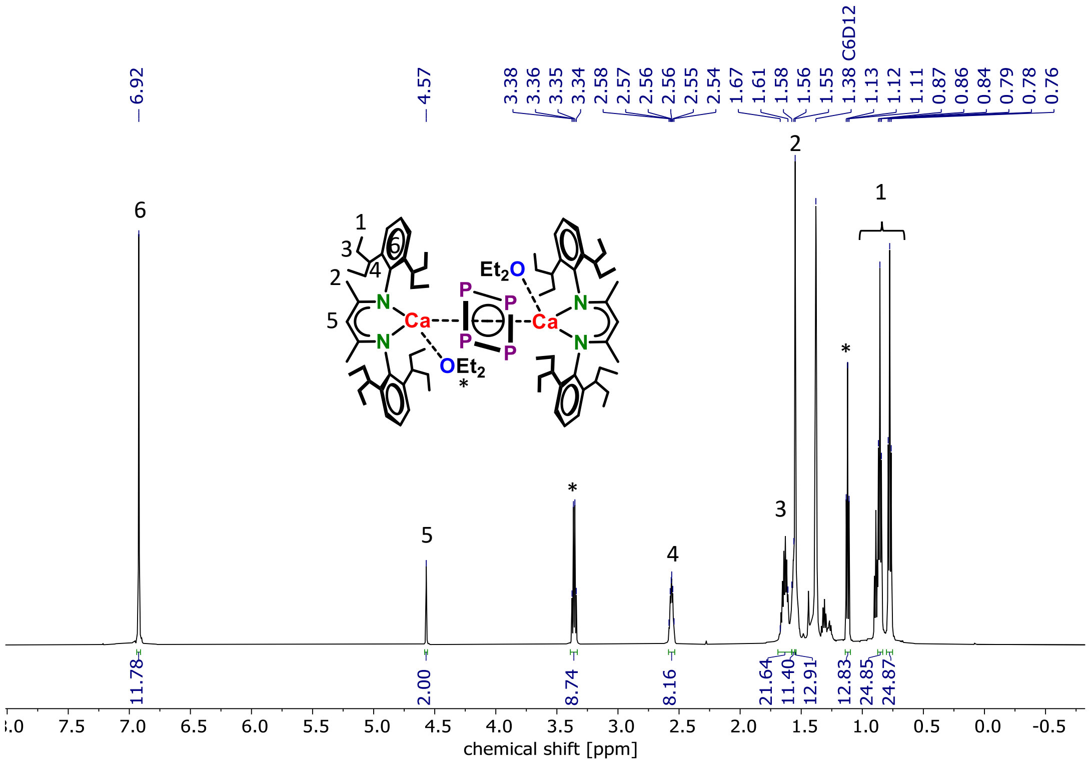

Figure S30. ^{1}H NMR (600.13 MHz, C_6D_{12}, 298K) of [(BDI*)Ca(OEt$_2$)]$_2$(*cyclo*-P_4) (**5**).

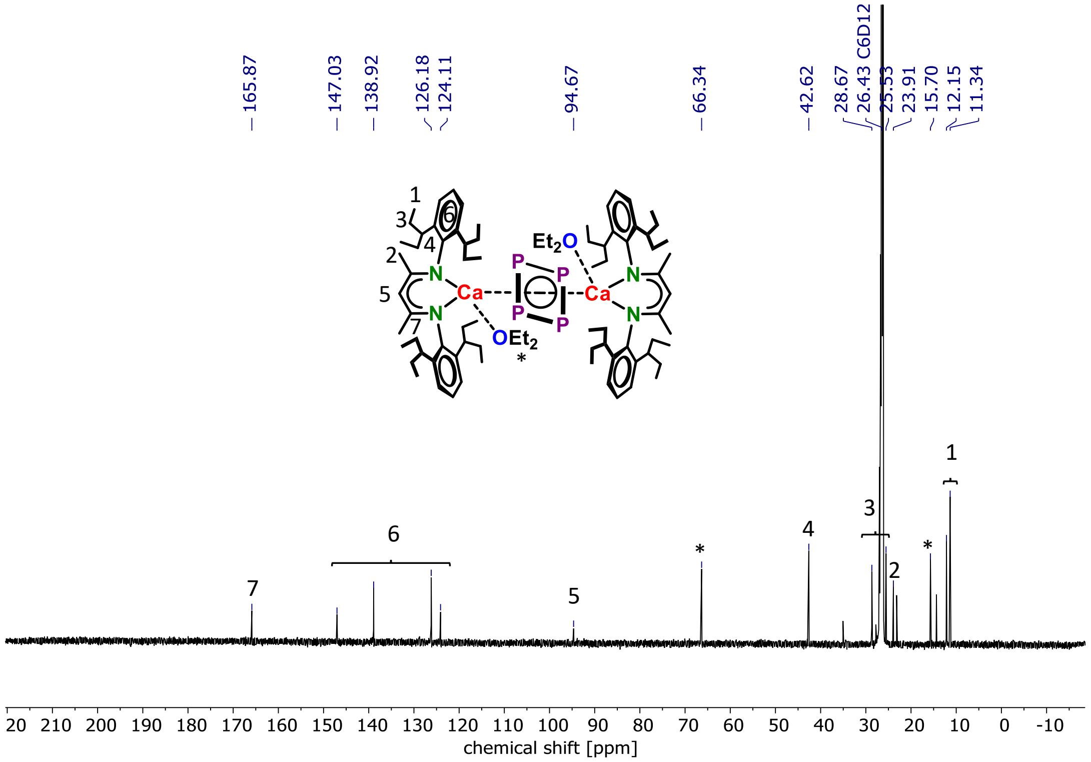

Figure S31. ^{13}C NMR (150.92 MHz, C_6D_{12}, 298K) of $[(BDI^*)Ca(OEt_2)]_2$(*cyclo*-P_4) (**5**).

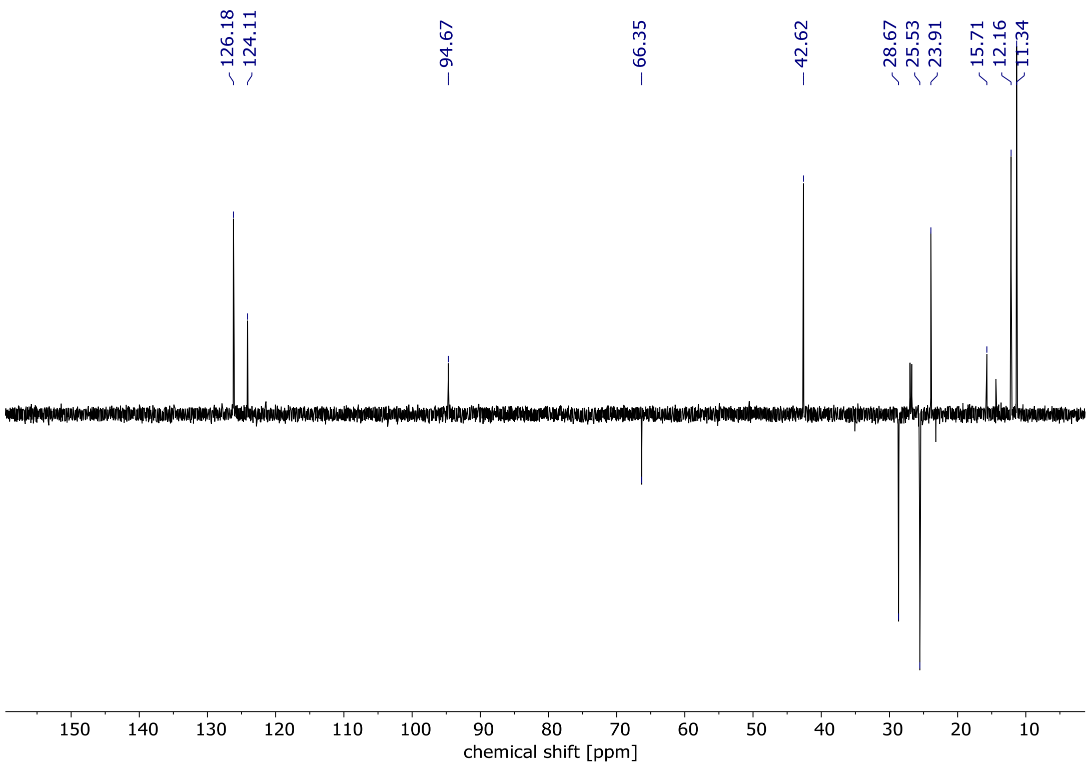

Figure S32. ^{13}C(DEPT 135) NMR (150.92 MHz, C_6D_{12}, 298K) of $[(BDI^*)Ca(OEt_2)]_2$(*cyclo*-P_4) (**5**).

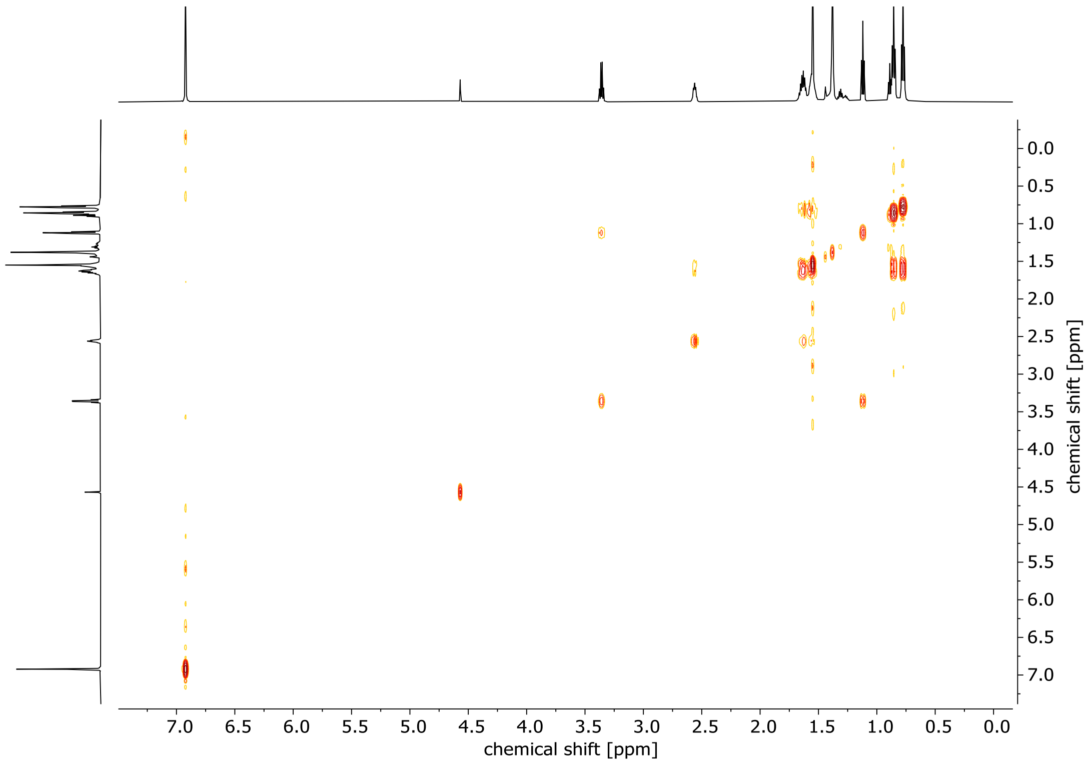

Figure S33. ^{1}H-^{1}H COSY NMR (600.13 MHz, C_6D_{12}, 298K) of $[(BDI^*)Ca(OEt_2)]_2$(*cyclo*-P_4) (**5**).

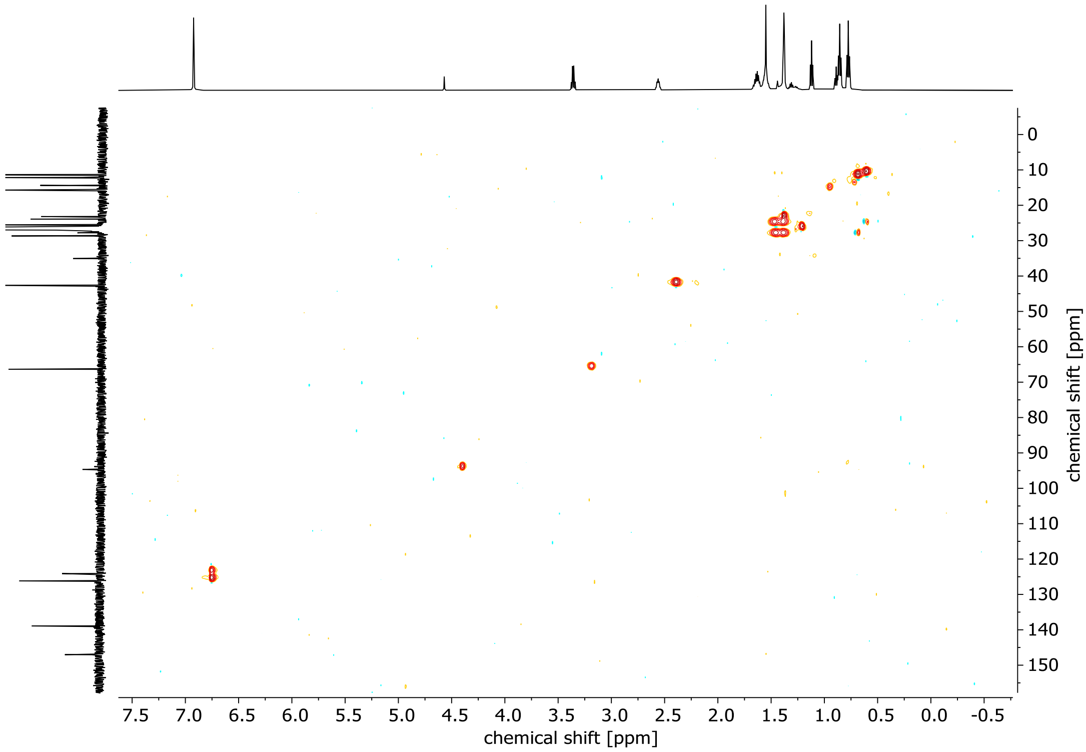

Figure S34. ^{1}H-^{13}C HSQC NMR (600.13/150.91 MHz, C_6D_{12}, 298K) of [(BDI*)Ca(OEt_2)]$_2$(*cyclo*-P_4) (**5**).

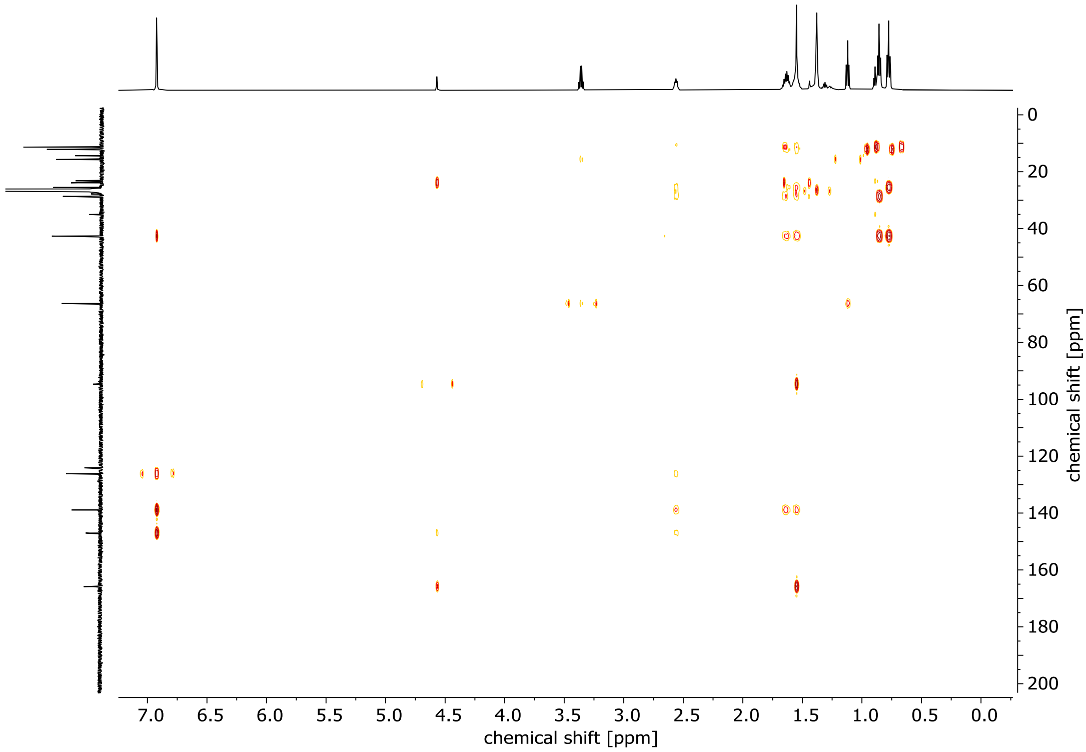

Figure S35. ^{1}H-^{13}C HMBC NMR (600.13/150.91 MHz, C_6D_{12}, 298K) of [(BDI*)Ca(OEt_2)]$_2$(*cyclo*-P_4) (**5**).

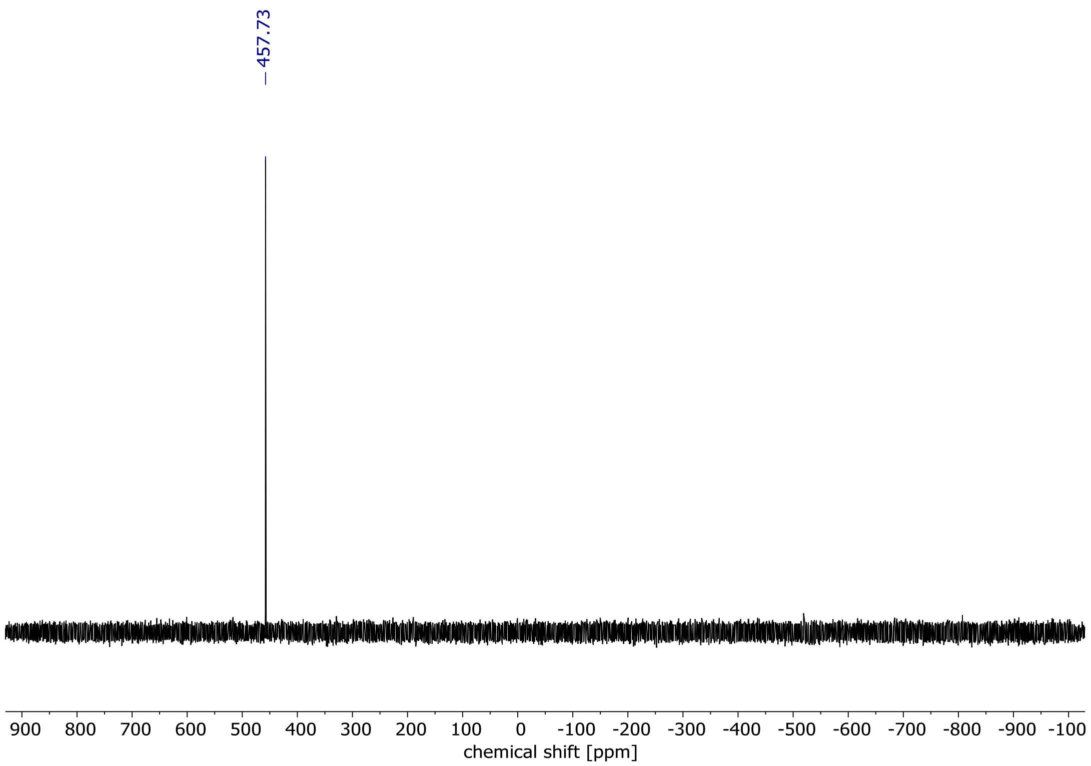

Figure S36. $^{31}P\{^{1}H\}$ NMR (242.92 MHz, C_6D_{12}, 298K) of $[(BDI^*)Ca(OEt_2)]_2$(*cyclo*-P_4) (**5**).

4. Selected NMR Spectra

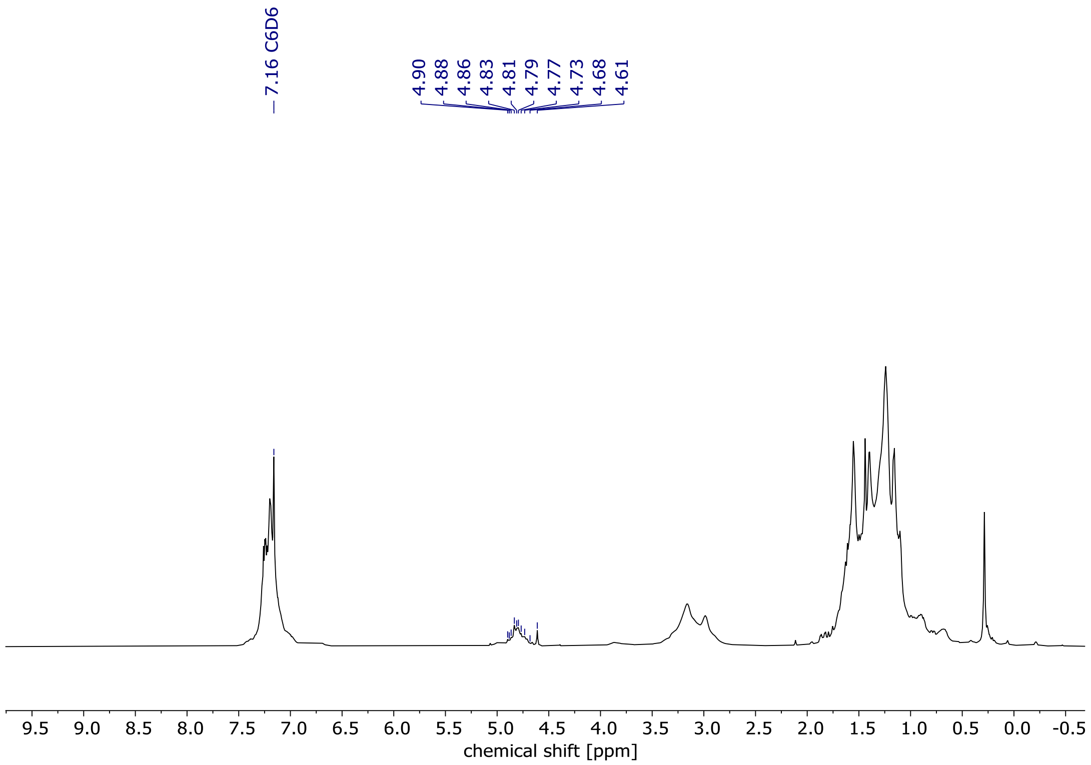

Figure S37. Reaction of $[(BDI)Mg]_2$ with P_4. ^1H NMR (600.13 MHz, C_6D_6, 298K) after 30 min shows very unselective conversion and signal broadening. This may be due to poor solubility of reagents and the heterogeneity of the reaction mixture.

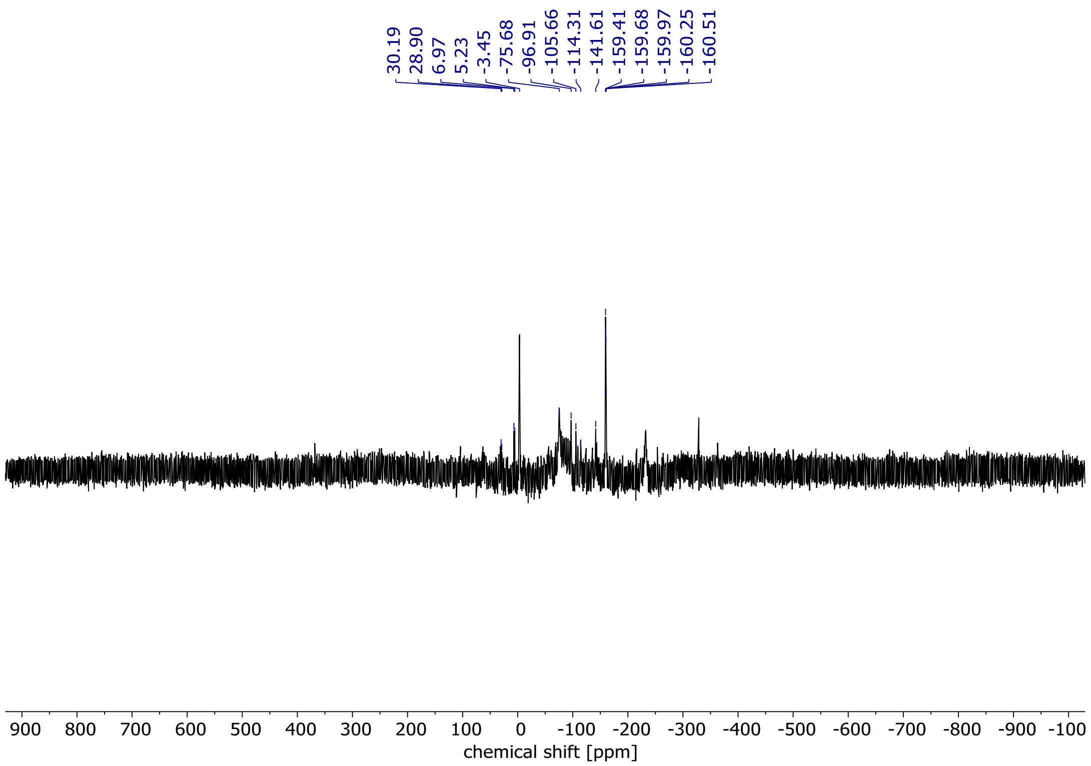

Figure S38. Reaction of $[(BDI)Mg]_2$ with P_4. $^{31}P\{^1H\}$ NMR (242.92 MHz, C_6D_6, 298K) of reaction mixture after 30 min shows very unselective conversion of P_4 to several different products.

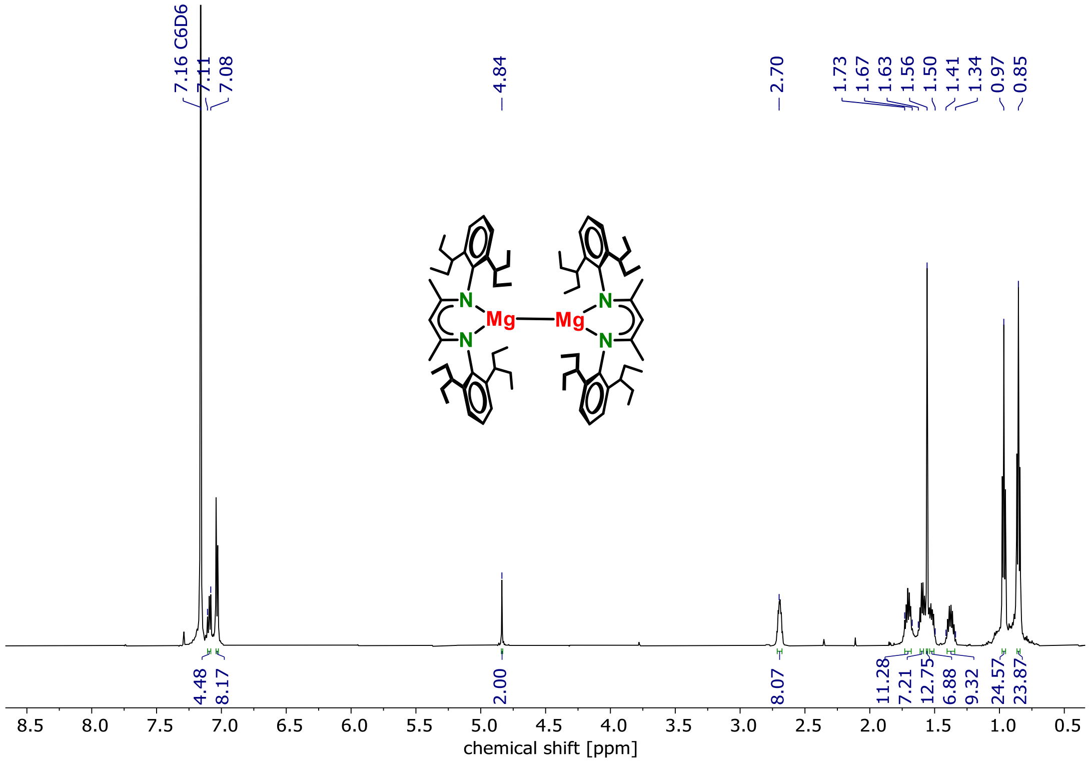

Figure S39. ^1H NMR (600.13 MHz, C_6D_6, 298 K) of crude $[(BDI^*)Mg]_2$.

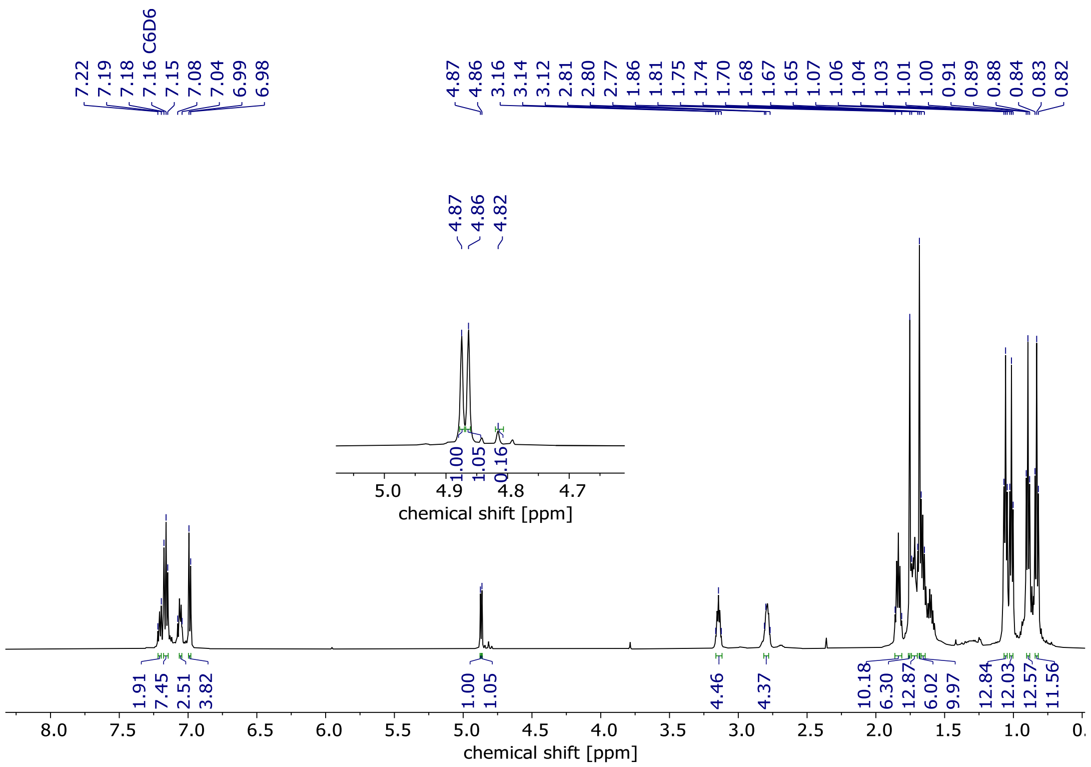

Figure S40. ^{1}H NMR (600.13 MHz, C_6D_6, 298 K) obtained from reaction of $[(BDI^*)Mg]_2$ with P_4 before work up. It shows selective formation of $[(BDI^*)Mg]_2(P_4)$ (**2**) with a minor side product.

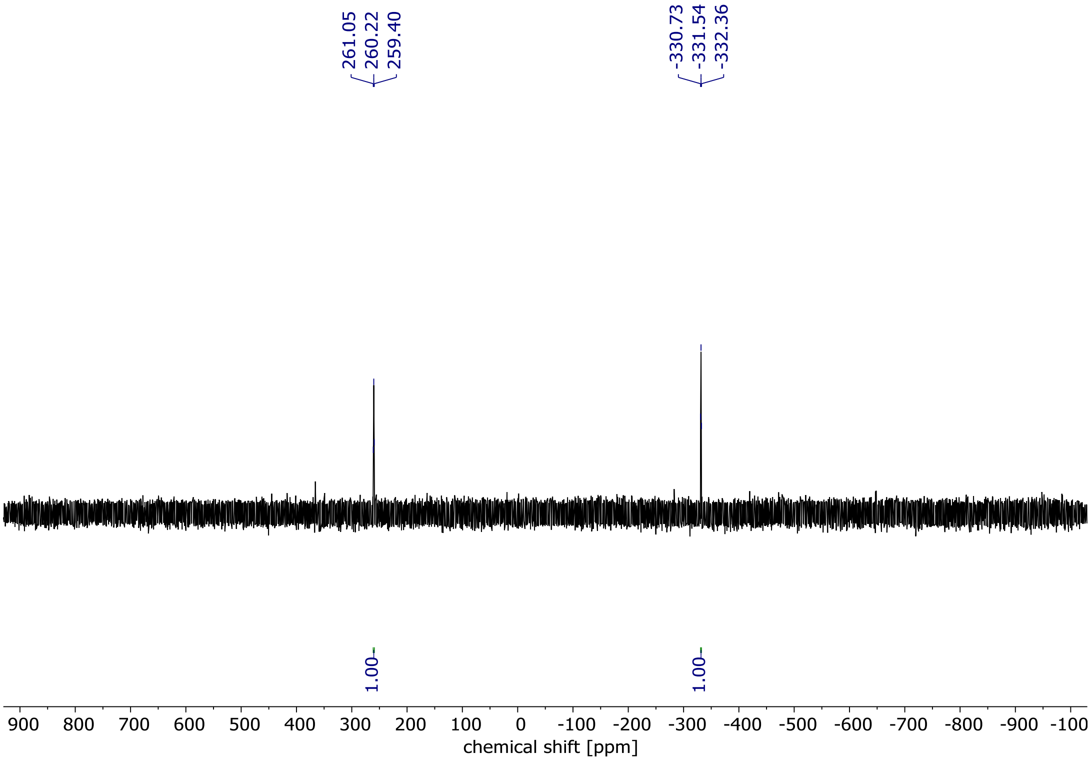

Figure S41. $^{31}P\{^{1}H\}$ NMR (242.92 MHz, C_6D_6, 298 K) obtained from reaction of $[(BDI^*)Mg]_2$ with P_4 before work up. It shows selective formation of $[(BDI^*)Mg]_2(P_4)$ (**2**).

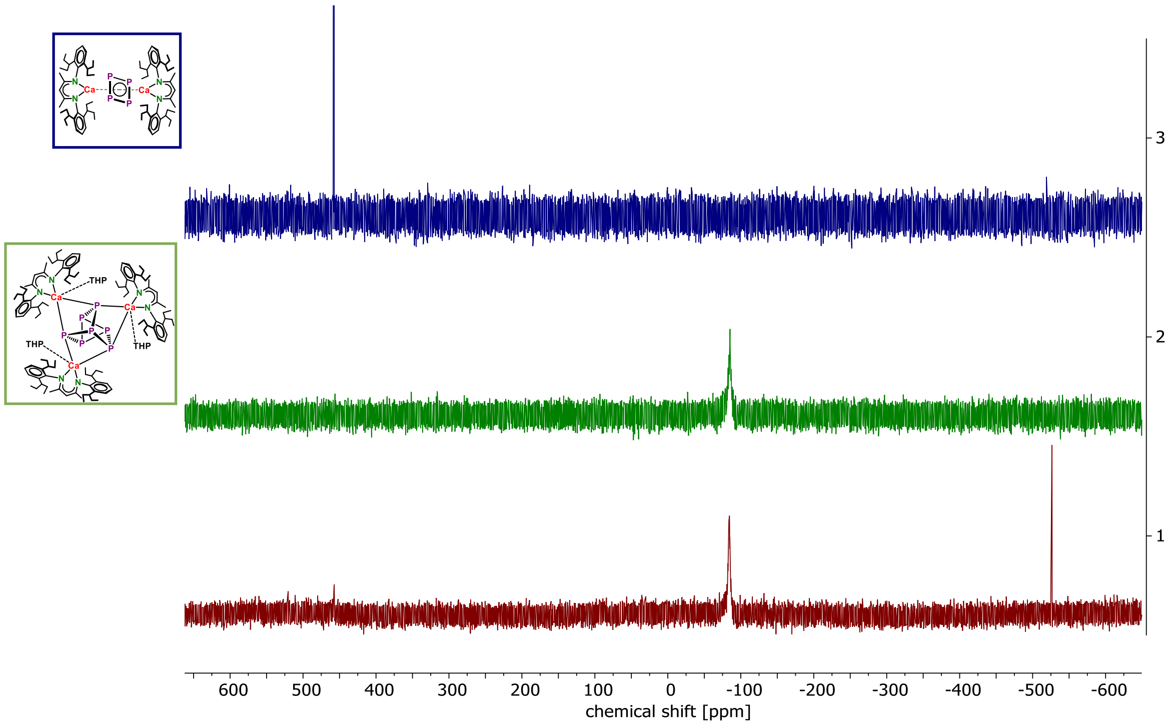

Figure S42. $^{31}P\{^{1}H\}$ NMR (242.92 MHz, C_7D_{14}, 298K) of reaction of [(BDI*)Ca]$_2$(N_2) with 1.00 eq. P_4 after 30 min stirring at room temperature (bottom) compared to $^{31}P\{^{1}H\}$ NMR spectrum of isolated [(BDI*)Ca(THP)]$_3$(P_7) (**3**) (middle) and [(BDI*)Ca]$_2$(*cyclo*-P_4) (**5**) (top). It shows formation of *cyclo*-P_4^{2-} and P_7^{3-} complex in a ratio of circa 0.09:0.91.

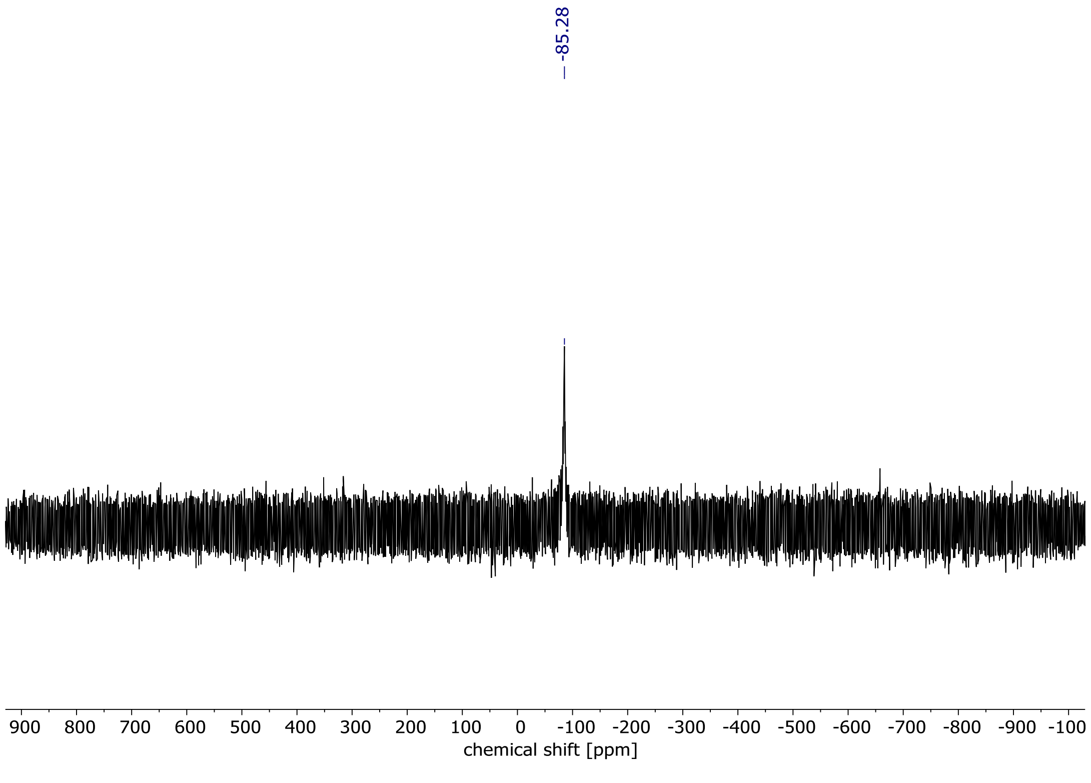

Figure S43. $^{31}P\{^{1}H\}$ NMR (242.92 MHz, C_7D_{14}, 298K) of reaction of [(BDI*)Ca]$_2$(N_2) with 1.00 eq. P_4 shows selective formation of P_7^{3-} complex (**3**) after for 4h.

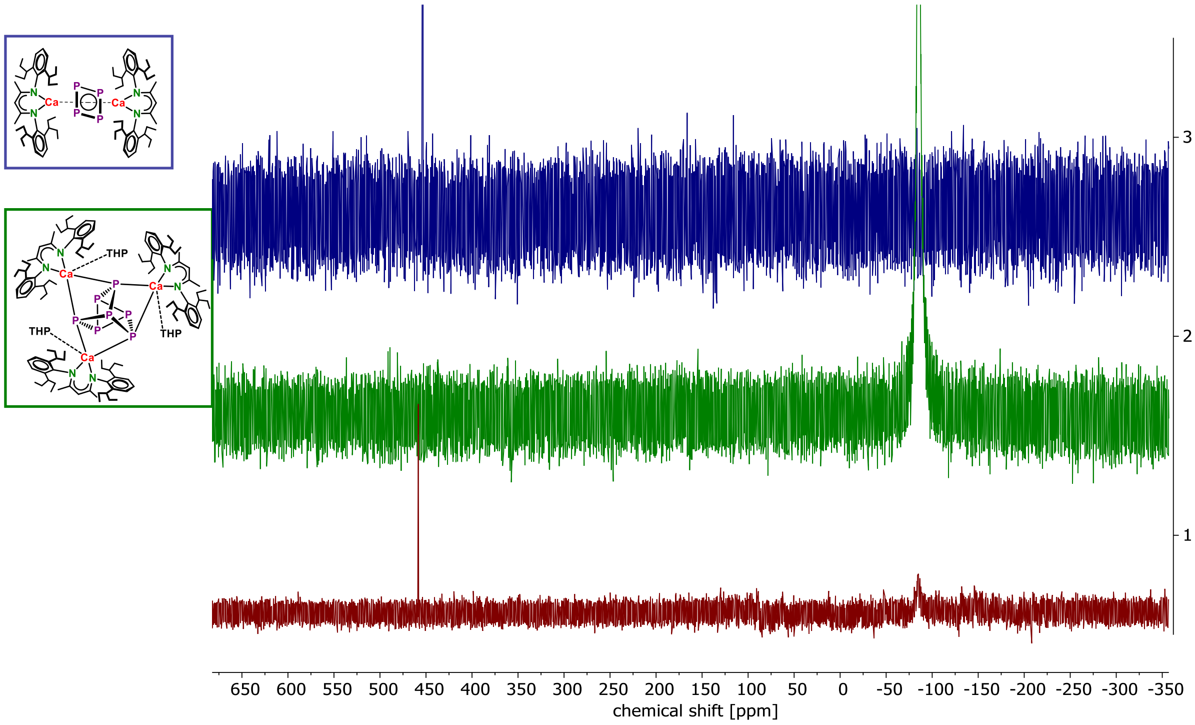

Figure S44. Reaction of [(BDI*)Ca]$_2$(benzene) in C_6D_{12} with 1.00 eq P_4. $^{31}P\{^1H\}$ NMR (242.92 MHz, C_6D_{12}, 298K) after stirring for 30 minutes at room temperature (bottom) compared to $^{31}P\{^1H\}$ NMR spectrum of isolated [(BDI*)Ca(THP)]$_3$(P_7) (**3**) (middle) and [(BDI*)Ca]$_2$(*cyclo*-P_4) (**5**) (top). It shows formation of *cyclo*-P_4^{2-} and P_7^{3-} complex in a ratio of circa 0.43:0.57.

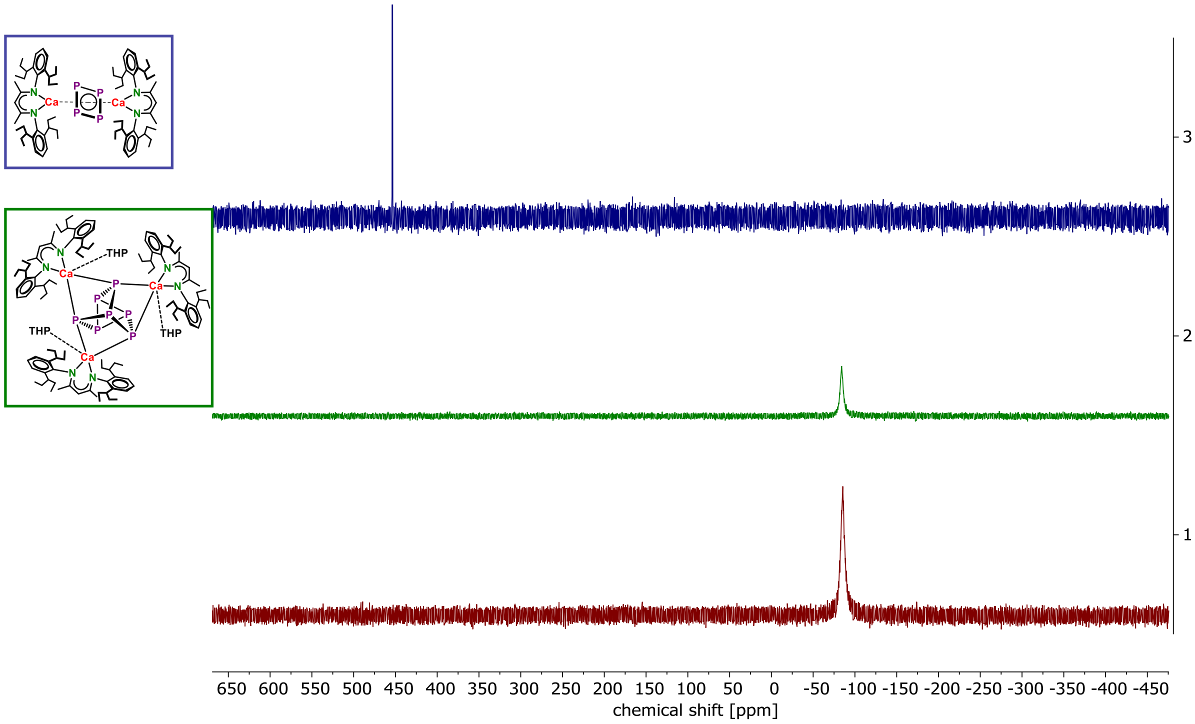

Figure S45. Reaction of [(BDI*)Ca]$_2$(benzene) in C_6D_{12} with 1.00 eq P_4. $^{31}P\{^1H\}$ NMR (242.92 MHz, C_6D_{12}, 298K) after stirring overnight (bottom) compared to $^{31}P\{^1H\}$ NMR spectrum of isolated [(BDI*)Ca(THP)]$_3$(P_7) (**3**) (middle) and [(BDI*)Ca]$_2$(*cyclo*-P_4) (**5**) (top). It shows full conversion of *cyclo*-P_4^{2-} into P_7^{3-} complex overnight.

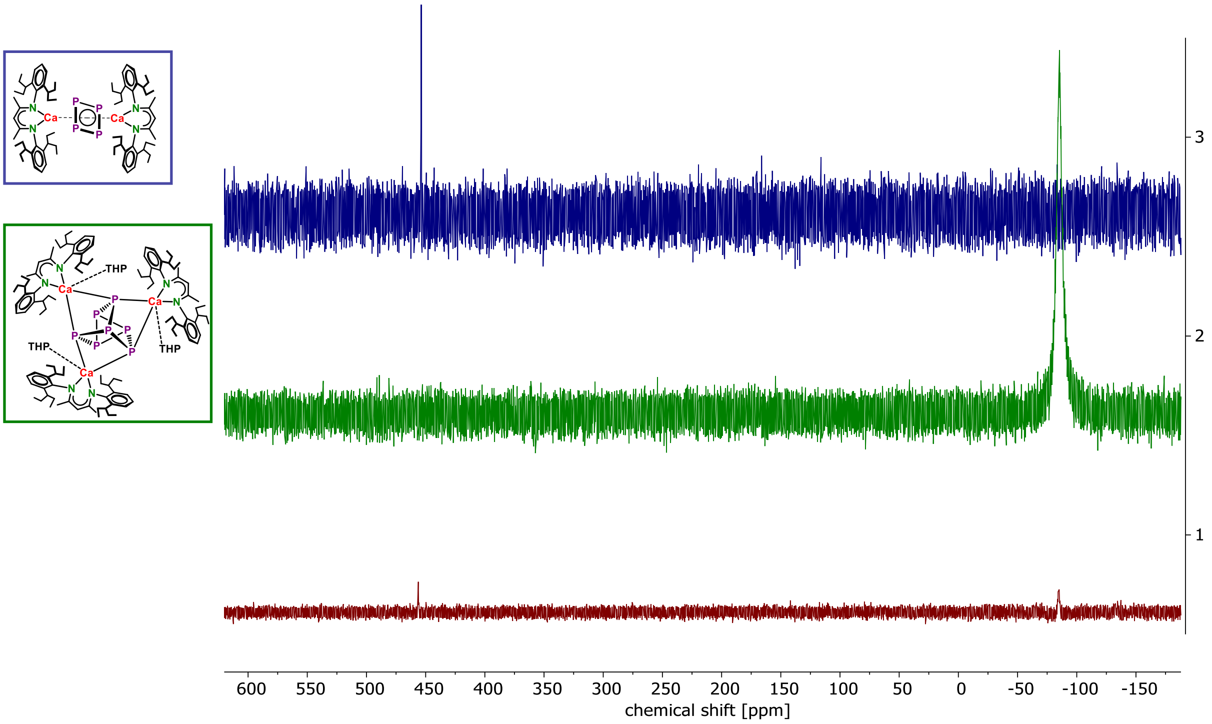

Figure S46. Reaction of $[(BDI^*)Ca]_2$(*p*-xylene) in C_6D_{12} with 1.00 eq P_4. $^{31}P\{^1H\}$ NMR (242.92 MHz, C_6D_{12}, 298K) after stirring for 30 min at room temperature (bottom) compared to $^{31}P\{^1H\}$ NMR spectrum of isolated $[(BDI^*)Ca(THP)]_3(P_7)$ (**3**) (middle) and $[(BDI^*)Ca]_2$(*cyclo*-P_4) (**5**) (top). It shows formation of *cyclo*-P_4^{2-} and P_7^{3-} complex in a ratio of circa 0.36:0.64.

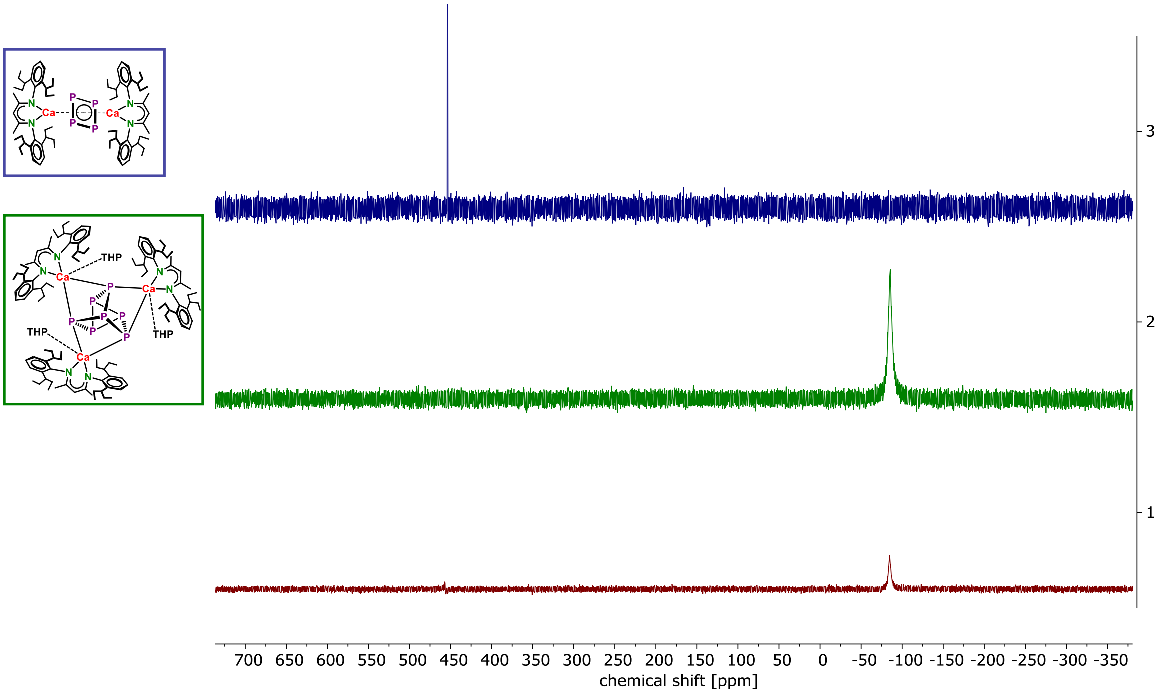

Figure S47. Reaction of $[(BDI^*)Ca]_2$(*p*-xylene) in C_6D_{12} with 1.00 eq P_4. $^{31}P\{^1H\}$ NMR (242.92 MHz, C_6D_6, 298K) after stirring overnight (bottom) compared to $^{31}P\{^1H\}$ NMR spectrum of isolated $[(BDI^*)Ca(THP)]_3(P_7)$ (**3**) (middle) and $[(BDI^*)Ca]_2$(*cyclo*-P_4) (**5**) (top). It shows full conversion of *cyclo*-P_4^{2-} into P_7^{3-} complex overnight.

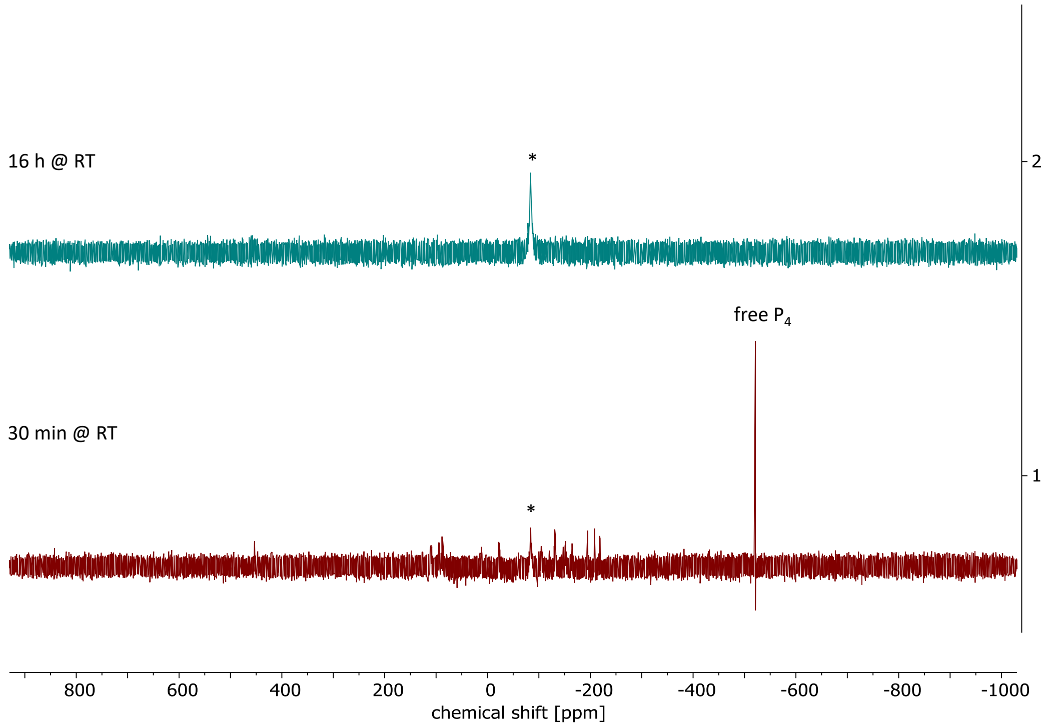

Figure S48. Reaction of [(BDI*)Ca]$_2$(anthracene) in C_6D_{12} with P_4. $^{31}P\{^1H\}$ NMR (242.92 MHz, C_6D_6, 298K) after stirring 30 min (bottom) compared to $^{31}P\{^1H\}$ NMR spectrum after 16 h at room temperature. After 30 min, a myriad of products was obtained, including the P_7 complex (**3**) (marked with asterisk).

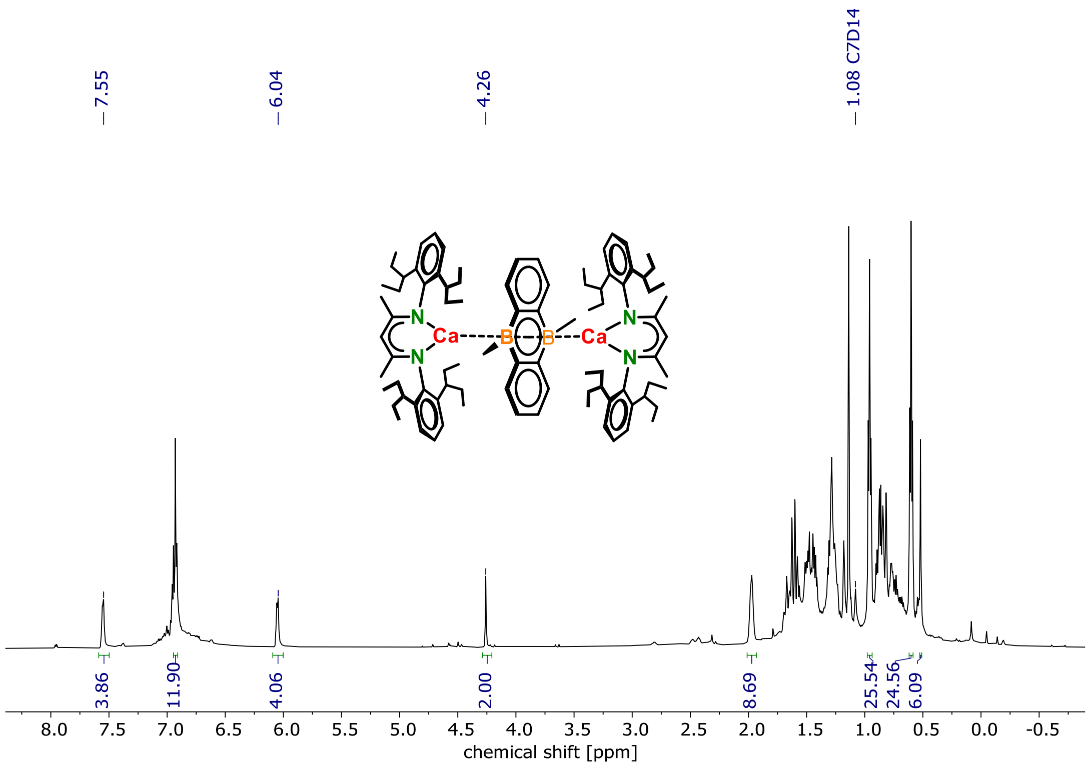

Figure S49. Crude ^{1}H NMR (600.13 MHz, C_7D_{14}, 298K) of [(BDI*)Ca]$_2$(N_2) and DBA after shaking the J.-Young NMR tube. It shows clean and selective formation of [(BDI*)Ca]$_2$(DBA) (**4**).

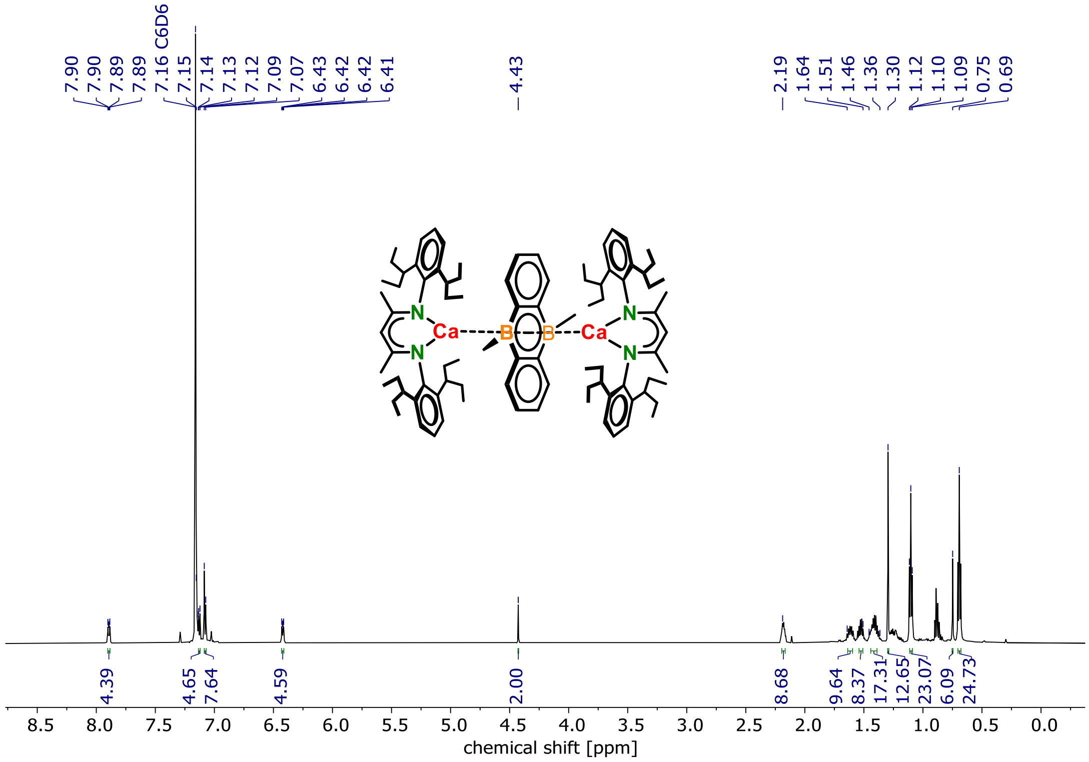

Figure S50. ^{1}H NMR (600.13 MHz, C_6D_6, 298K) of crystalline [(BDI*)Ca]$_2$(DBA) (**4**) recorded in C_6D_6.

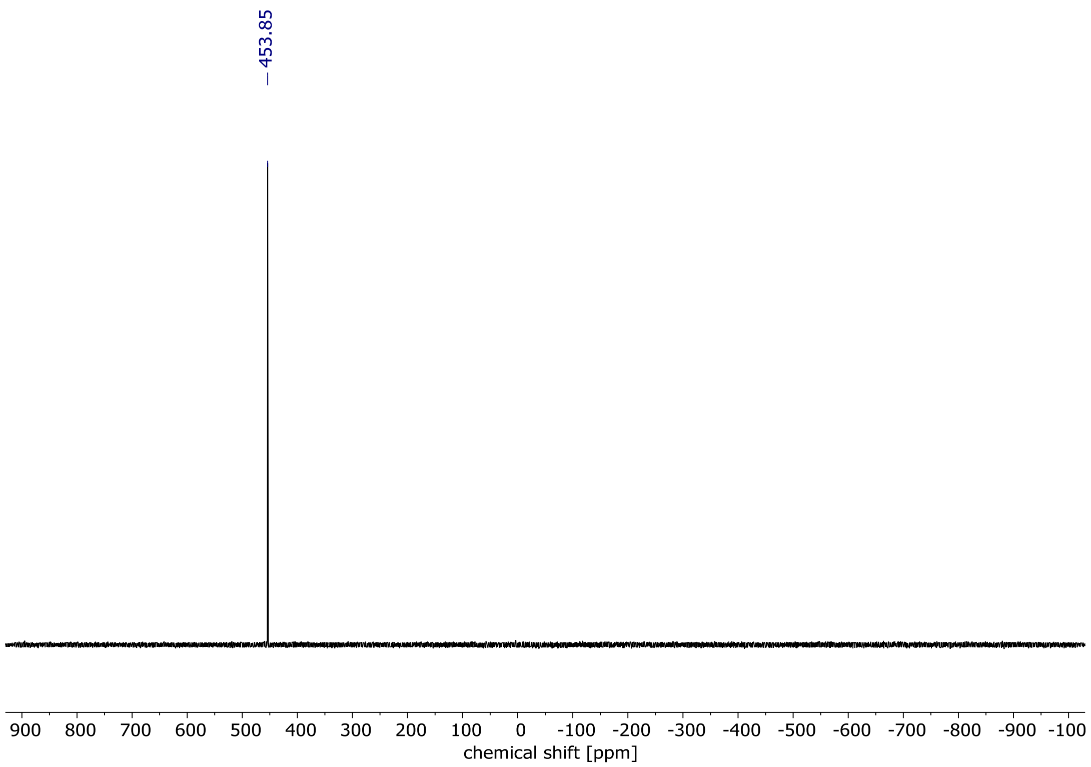

Figure S51. $^{31}P\{^1H\}$ NMR (242.92 MHz, C_6D_6, 298K) of reaction mixture of [(BDI*)Ca]$_2$(DBA) in C_6D_6 with 1.00 eq. P_4 before work up.

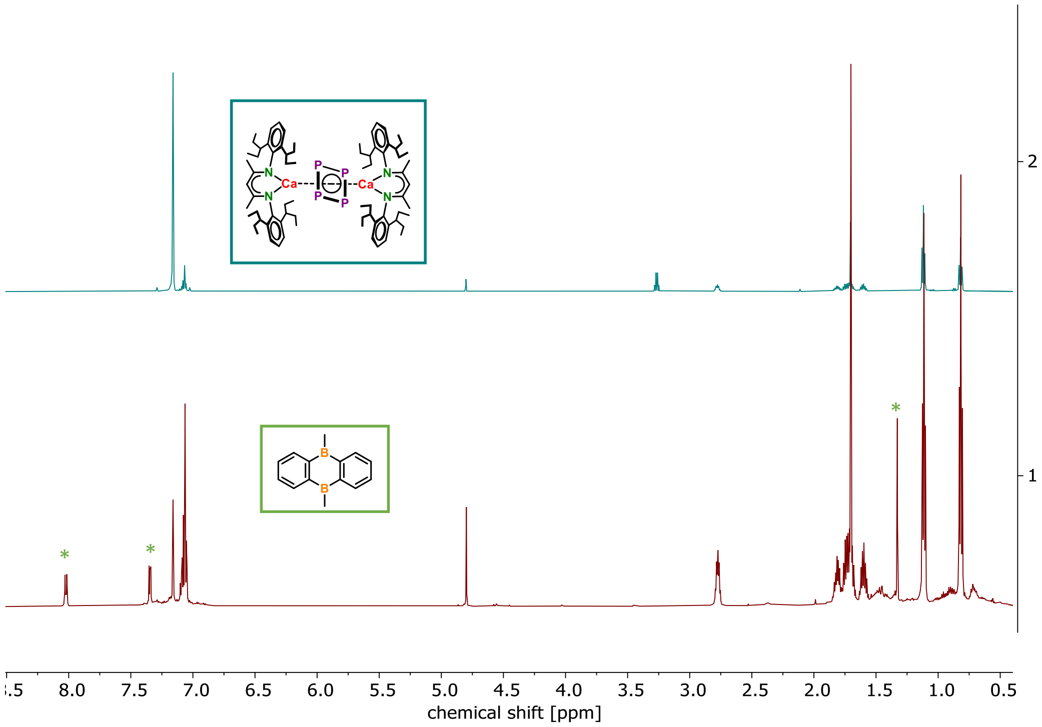

Figure S52. Reaction of [(BDI*)Ca]$_2$(DBA) (**4**) in C_6D_6 with 1.00 eq. P_4. ^{1}H NMR (600.13 MHz, C_6D_6, 298K) of reaction (bottom) compared to ^{1}H NMR of pure complex (top) after stirring overnight shows clean transformation to cyclo-P_4^{2-} complex (**5**) and free DBA (signals are marked with green asterisks).

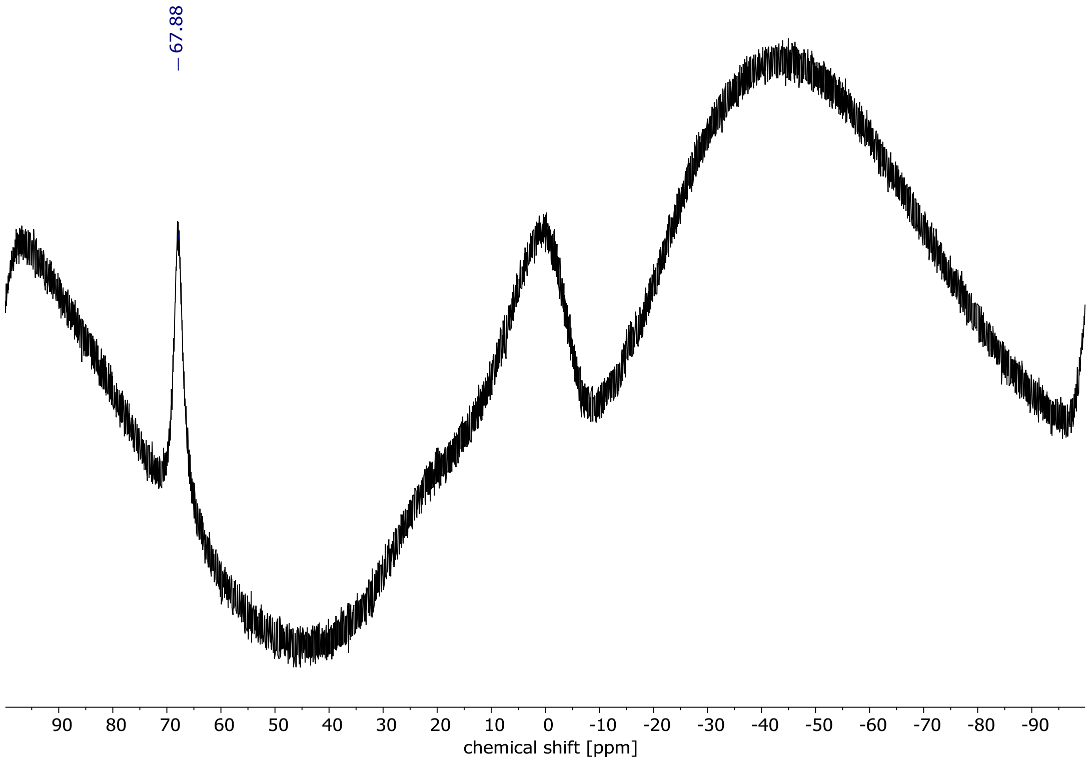

Figure S53. ^{11}B NMR (192.55 MHz, C_6D_6, 298K) of reaction mixture of [(BDI*)Ca]$_2$(DBA) (**4**) in C_6D_6 with 1.00 eq. P_4 shows one signal at 67.88 ppm for free DBA.

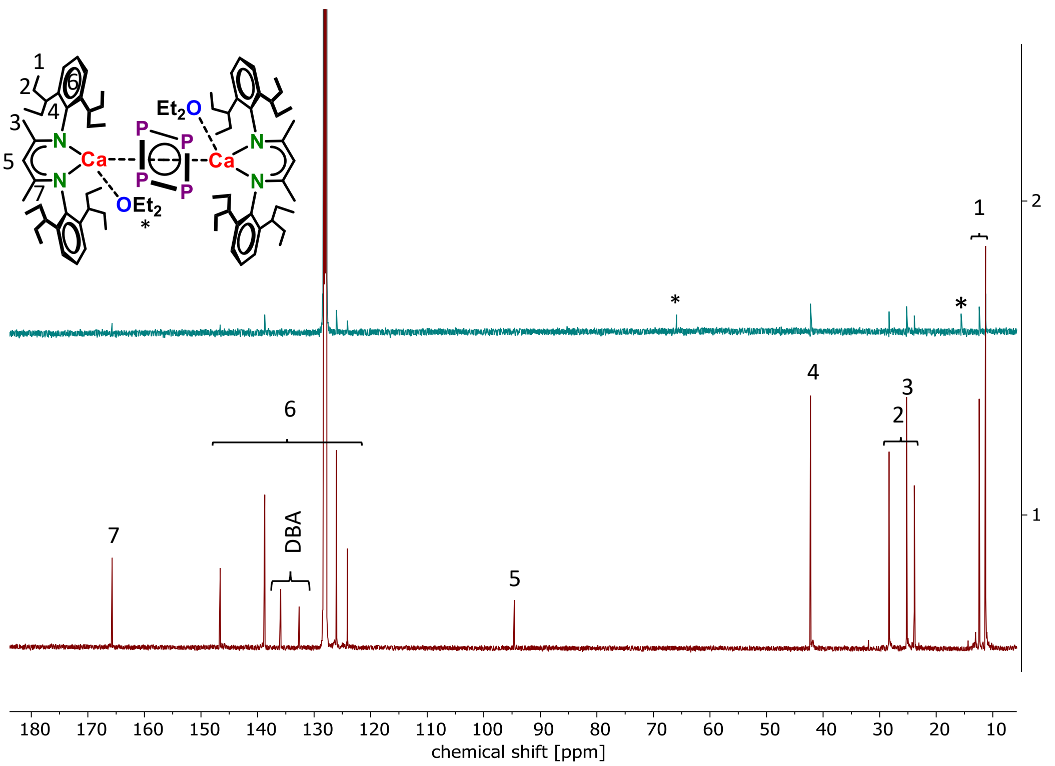

Figure S54. Reaction of [(BDI*)Ca]$_2$(DBA) in C_6D_6 with 1.00 eq. P_4. ^{13}C NMR (150.91 MHz, C_6D_6, 298K) of reaction (bottom) compared to ^{13}C NMR of pure crystalline complex (**5**) (top) after stirring overnight shows clean transformation to *cyclo*-P_4^{2-} complex and free DBA.

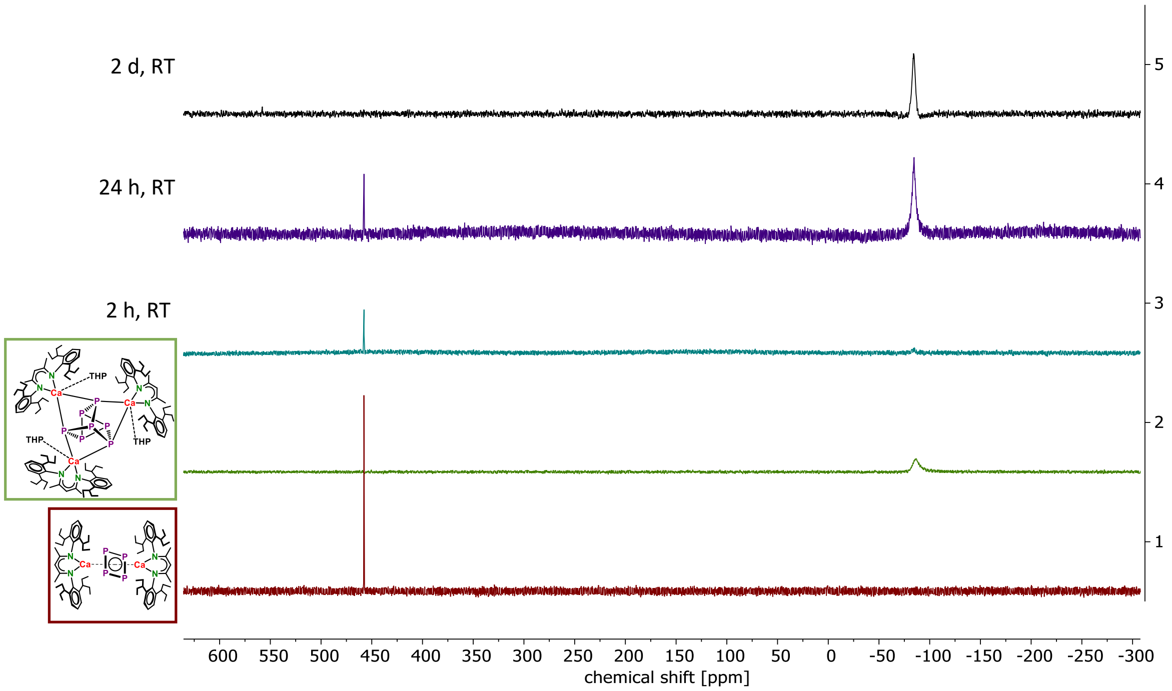

Figure S55. Stacked $^{31}P\{^1H\}$ NMR spectra (242.92 MHz, C_6D_{12}, 298K) of reaction of crystalline [(BDI*)Ca(OEt$_2$)]$_2$(*cyclo*-P_4) (**5**) with P_4 in C_6D_{12} compared to $^{31}P\{^1H\}$ NMR spectra of isolated P_7^{3-} (**3**) (green) and (*cyclo*-P_4)$^{2-}$ (**5**) (red) complexes. It shows full conversion of [(BDI*)Ca(OEt$_2$)]$_2$(*cyclo*-P_4) with P_4 in two days at room temperature (black).

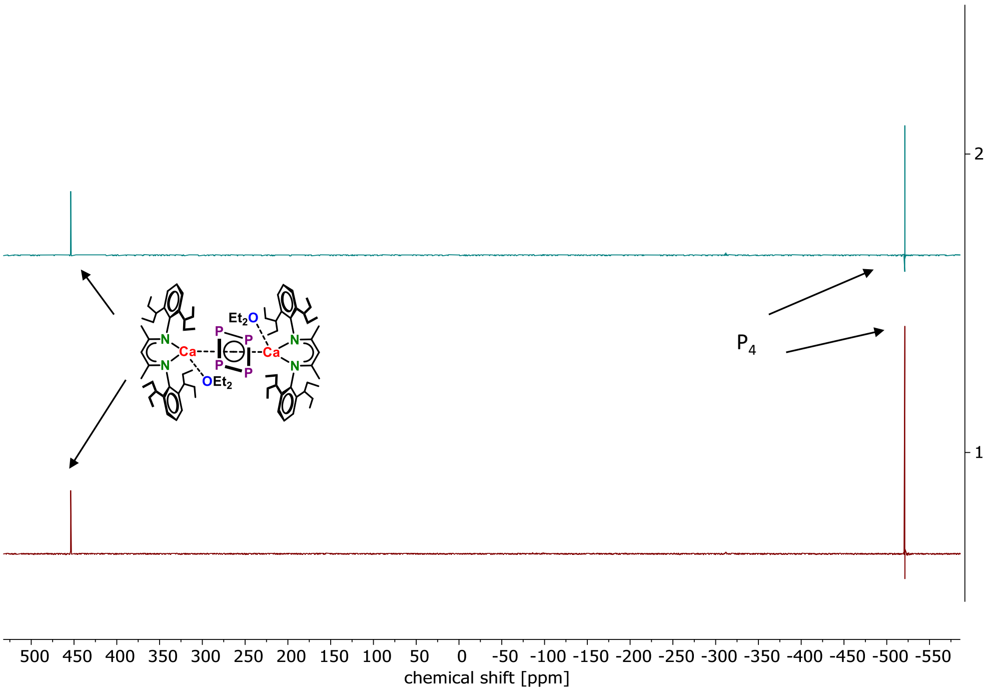

Figure S56. Comparison of $^{31}P\{^1H\}$ NMR spectra (242.92 MHz, C_6D_{12}, 298K) of reaction of crystalline $[(BDI^*)Ca(OEt_2)]_2$(*cyclo*-P_4) (**5**) with one equivalent of DBA and P_4 immediately after mixing (bottom) and after 3 days (top) shows no conversion of (**5**) to (**3**).

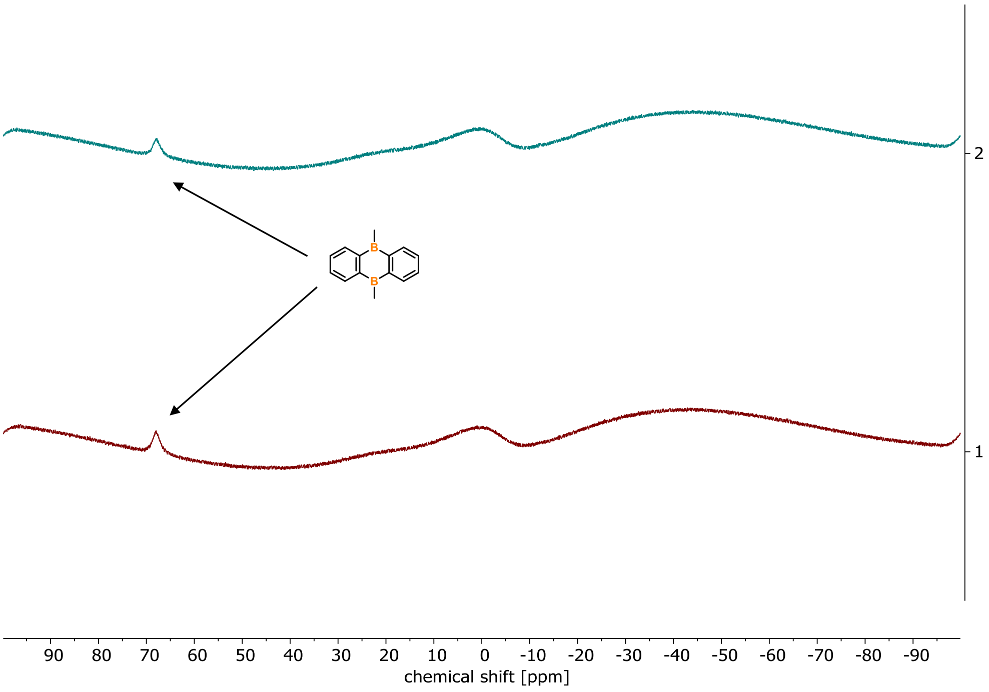

Figure S57. Comparison of $^{11}B\{^1H\}$ NMR spectra (192.55 MHz, C_6D_{12}, 298K) of reaction of crystalline $[(BDI^*)Ca(OEt_2)]_2$(*cyclo*-P_4) (**5**) with one equivalent of DBA and P_4 immediately after mixing (bottom) and after 3 days (top) shows no conversion of DBA.

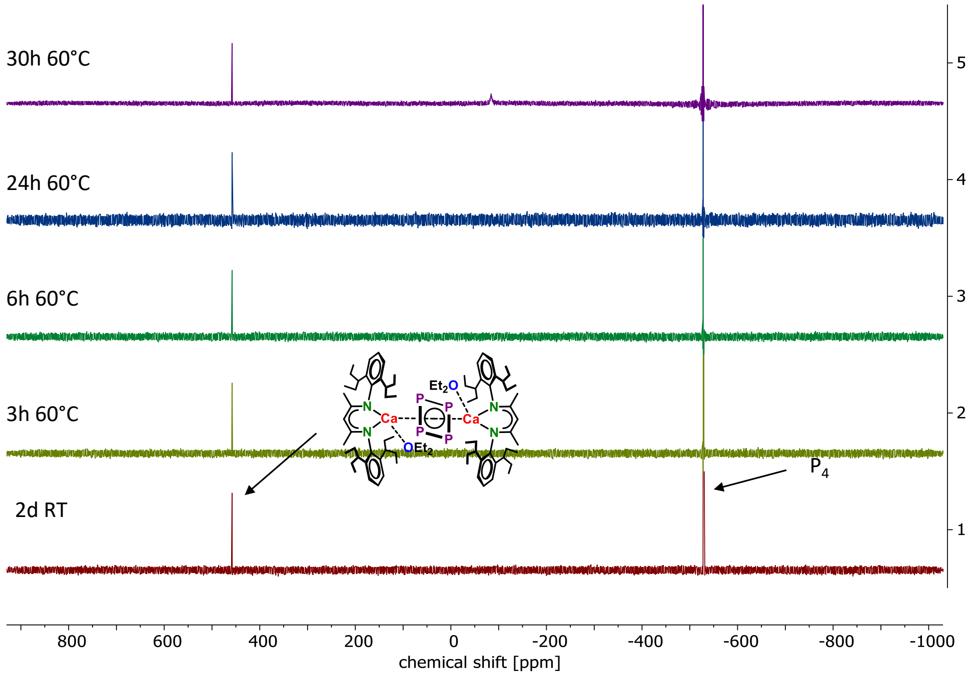

Figure S58. Stacked $^{31}P\{^1H\}$ NMR spectra (242.92 MHz, C_6D_{12}, 298K) of reaction of crystalline $[(BDI^*)Ca(OEt_2)]_2(cyclo\text{-}P_4)$ (**5**) with P_4 and catalytic amount of DBA (5 mol%) in C_6D_{12} shows conversion to the Zintl P_7^{3-} complex (**3**) under forcing conditions at 60 °C only after 30 h and thus, the conversion is retarded compared to the reaction of just crystalline $[(BDI^*)Ca(OEt_2)]_2(cyclo\text{-}P_4)$ (**5**) mixed with P_4 (see **Figure S56**). This underlines the inhibition role of DBA in the case of P-cluster growth by [(BDI*)Ca] based complexes.

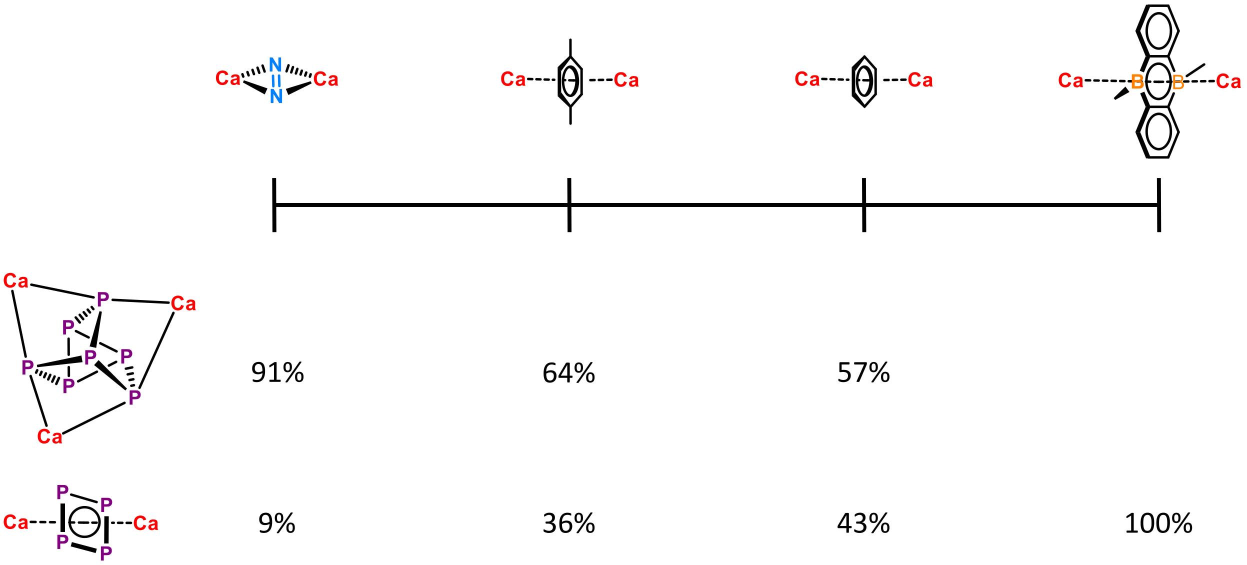

Figure S59. Comparison of product ratio based on integration of signals in $^{31}P\{^1H\}$ NMR of formed P_7^{3-} and *cyclo*-P_4^{2-} anions after 30 min stirring of reaction mixture at room temperature.

5. Crystal Structure Determinations

Suitable single crystals of compounds **1**-**5** were embedded in protective perfluoropolyalkyether oil (viscosity 1800 cSt; ABCR GmbH) on a microscope slide and a single specimen was selected and subsequently transferred to the cold nitrogen gas stream of the diffractometer.

The intensity data was collected at 100 K using CuK_α radiation (λ = 1.54184 Å) on an Agilent SuperNova dual radiation diffractometer with microfocus X-ray sources and mirror optics. The measured data were processed with the CrysAlisPro software package.[S7] Data were corrected for Lorentz and polarization effects, and an empirical absorption correction using spherical harmonics as well as a numerical absorption correction based on Gaussian integration over a multifaceted crystal model were applied. Using Olex2,[S8] the structures were solved by dual-space methods (SHELXT)[S9] and refined by full-matrix least-squares procedures on F^2 using SHELXL.[S10] All non-hydrogen atoms were refined with anisotropic displacement parameters. Most H-atoms were placed in geometrically calculated positions and refined by using a riding model where each H-atom was assigned a fixed isotropic displacement parameter with a value equal to 1.2U_{eq} (CH or CH_2) or 1.5U_{eq} (CH_3) of its parent C-atom.

Compound [(BDI)Mg]$_4$(P_8) (**1**) showed signs of disorder. Two DIPP moieties, two additional *i*Pr groups and the co-crystallized benzene were affected. This disorder was modeled with the help of similarity restraints (SADI, SIMU) and rigid bond restraints (RIGU).[S11] Additionally, an ISOR restraint was placed on C18 and C18A. The disordered benzene moiety was constrained as perfect hexagon with C-C bond length of 1.39 Å by applying AFIX 66. The relative occupancies of the alternative orientations of the different disordered parts of the compound were refined to 0.552(19)/0.448(19) (DIPP 1), 0.597(10)/0.403(10) (DIPP 2), 0.749(12)/0.251(12) (*i*Pr 1), 0.785(19)/0.215(19) (*i*Pr 2) and 0.765(14)/0.235(14) (benzene), respectively.

The asymmetric unit of [(BDI*)Mg]$_2$(P_4) (**2**) contained half of the molecule. Extensive disorder was also present in this case. Here, only the magnesium and nitrogen atoms as well as the ligand backbone were not affected. Similarity restraints (SADI, SIMU) were used during refinement and site occupancy factors of 0.505(9)/0.495(9) (P_4 sub-unit) and 0.545(3)/0.455(3) (DIPeP) were determined, respectively. The other DIPeP moiety was disordered over a mirror plane and therefore site occupancy factors were constrained to 0.5 in this part of the molecule. Due to the severe disorder and the restraints used, bond lengths and angles of this compound are somewhat unreliable and should not be discussed.

In case of compound [(BDI*)Ca(THP)]$_3$(P_7) (**3**), the hydrogen atoms at C3, C45 and C87 in the backbone of the BDI* ligands deviated noticeably from the positions calculated by the riding model. Therefore, these H atoms were placed in the positions indicated by a difference electron density map and their positions were refined together with an isotropic displacement parameter. Disorder was present as well and affected a phenyl moiety with one of the connected 3-pentyl groups, a separate 3-pentyl group and the co-crystallized solvent. The disorder was modeled with the help of similarity restraints (SADI, SIMU), geometric restraints (FLAT) and rigid bond restraints (RIGU).[S11] The relative occupancies of the alternative orientations of the different disordered parts of the compound were refined to 0.534(4)/0.466(4) (Ph + 3-pentyl), 0.762(4)/0.238(4) (3-pentyl) and 0.737(4)/0.263(4) (co-crystallized THP), respectively. Additional co-crystallized solvent moieties were also present, but in these cases it

was not possible to identify the heavily disordered solvents without doubt (a mixture of methylcyclohexane, THP and pentane was used for crystallization). Therefore, their contribution to the structure factors was secured by back-Fourier transformation using the solvent mask routine[S12] of the program Olex2.[S8] The solvent accessible voids treated this way had a size of 1714.4 $Å^3$ (22.4% of the unit cell) and contained 340.0 electrons/unit cell.

The asymmetric unit of crystals of compound $[(BDI^*)Ca]_2(DBA)$ (**4**) contained two crystallographically unique half-molecules of **4** and 1.5 cyclohexane. These solvent moieties as well as two 3-pentyl groups and a methyl group of the main compound were disordered. Similarity restraints (SADI, SIMU) and rigid bond restraints (RIGU)[S11] were used during refinement and site occupancy factors of 0.821(5)/0.179(5) (3-pentyl 1), 0.866(5)/0.134(5) (3-pentyl 2), 0.57(3)/0.43(3) (Me) and 0.487(3)/0.270(3)/0.243(2) (cyclohexane) were determined, respectively. The second cyclohexane moiety was disordered about an inversion center and was treated accordingly. Its site occupancy factor was constrained to 0.5.

Crystals of compound $[(BDI^*)Ca(OEt_2)]_2$(*cyclo*-P_4) (**5**) contained half of the molecule and a co-crystallized diethyl ether per asymmetric unit. The hydrogen atom at C3 in the backbone of the BDI* ligand deviated noticeably from the positions calculated by the riding model. Therefore, this H atom was placed in the position indicated by a difference electron density map and its positions was refined together with an isotropic displacement parameter. Significant disorder, which affected three out of four 3-pentyl groups, the central P4 unit as well as the diethyl ether moieties was noticed and modeled with the help of similarity restraints (SADI, SIMU) and rigid bond restraints (RIGU).[S11] The relative occupancies of the alternative orientations of the disordered parts were refined to 0.902(3)/0.098(3) (P_4 sub-unit), 0.511(8)/0.489(8) (3-pentyl 1), 0.764(6)/0.236(6) (3-pentyl 2), 0.840(6)/0.160(6) (3-pentyl 3), 0.867(5)/0.133(5) (Et_2O coordinated) and 0.515(10)/0.485(10) (Et_2O co-crystallized), respectively.

The crystal structure data has been deposited with the Cambridge Crystallographic Data Centre. CCDC 2407627-2407631 contain the supplementary crystallographic data for the complexes. This data can be obtained free of charge from The Cambridge Crystallographic Data Centre via www.ccdc.cam.ac.uk/data_request/cif.

Crystallographic and refinement data are summarized in Table **S1**.

Table S1. Crystal data and structure refinement for compounds **1-5**.

Compound	$[(BDI)Mg]_4(P_8)$ (**1**) ·Benzene	$[(BDI^*)Mg]_2(P_4)$ (**2**)	$[(BDI^*)Ca(THP)]_3(P_7)$ (**3**) ·THP [·solvent]
Identification code	hasj240610a	hasj240617a	hasj240506a
Empirical formula	$C_{122}H_{170}Mg_4N_8P_8$	$C_{74}H_{114}Mg_2N_4P_4$	$C_{131}H_{211}Ca_3N_6O_4P_7$ a)
Formula weight	2093.65	1232.19	2271.08 a)
Temperature/K	99.98(19)	100.0(2)	100.0(2)
Crystal system	monoclinic	orthorhombic	triclinic
Space group	$P2_1/c$	Pnma	P-1
a/Å	26.4049(5)	26.1211(5)	20.4699(6)
b/Å	18.7396(2)	21.8425(4)	20.4809(5)
c/Å	27.4993(5)	12.8482(3)	21.4594(5)
α/°	90	90	70.084(2)
β/°	118.570(3)	90	81.755(2)
γ/°	90	90	64.564(3)
Volume/$Å^3$	11950.2(5)	7330.5(3)	7638.7(4)
Z	4	4	2
$\rho_{calc}g/cm^3$	1.164	1.116	0.987 a)
μ/mm^{-1}	1.672	1.427	1.970 a)
F(000)	4504.0	2680.0	2472.0 a)
Crystal size/mm^3	0.311 × 0.077 × 0.063	0.343 × 0.216 × 0.126	0.297 × 0.189 × 0.145
Radiation	Cu Kα (λ = 1.54184)	Cu Kα (λ = 1.54184)	Cu Kα (λ = 1.54184)
2Θ range for data collection/°	7.32 to 145.166	7.668 to 140.114	7.634 to 145.446
Index ranges	-31 ≤ h ≤ 32, -22 ≤ k ≤ 15, -34 ≤ l ≤ 27	-31 ≤ h ≤ 30, -17 ≤ k ≤ 26, -15 ≤ l ≤ 15	-25 ≤ h ≤ 24, -25 ≤ k ≤ 21, -26 ≤ l ≤ 25
Reflections collected	48510	45281	85219
Independent reflections	23050 [R_{int} = 0.0293, R_{sigma} = 0.0400]	7152 [R_{int} = 0.0311, R_{sigma} = 0.0144]	29465 [R_{int} = 0.0293, R_{sigma} = 0.0296]
Data/restraints/parameters	23050/3246/1636	7152/2733/715	29465/623/1589
Goodness-of-fit on F^2	1.007	1.060	1.052
Final R indexes [I>=2σ (I)]	R_1 = 0.0441, wR_2 = 0.1072	R_1 = 0.0756, wR_2 = 0.2342	R_1 = 0.0369, wR_2 = 0.0955
Final R indexes [all data]	R_1 = 0.0580, wR_2 = 0.1163	R_1 = 0.0881, wR_2 = 0.2547	R_1 = 0.0424, wR_2 = 0.0991
Largest diff. peak/hole / e $Å^{-3}$	0.51/-0.30	0.40/-0.35	0.53/-0.43
CCDC number	2407627	2407628	2407629

a) Contribution of the masked disordered solvent neglected.

Table S1. Crystal data and structure refinement for compounds **1-5** (continued).

Compound	$[(BDI^*)Ca]_2(DBA)$ (**4**) ·1.5Cyclohexane	$[(BDI^*)Ca(OEt_2)]_2$(*cyclo*-P_4) (**5**) ·$2Et_2O$
Identification code	hasj240219a	hasj240524a
Empirical formula	$C_{97}H_{146}B_2Ca_2N_4$	$C_{90}H_{154}Ca_2N_4O_4P_4$
Formula weight	1469.95	1560.20
Temperature/K	99.97(16)	99.98(12)
Crystal system	triclinic	monoclinic
Space group	P-1	$P2_1/c$
a/Å	12.7375(2)	13.4588(2)
b/Å	16.5295(3)	23.0603(3)
c/Å	21.8122(3)	15.7858(2)
α/°	106.025(2)	90
β/°	90.6120(10)	111.8607(18)
γ/°	98.438(2)	90
Volume/Å^3	4359.87(13)	4547.02(13)
Z	2	2
ρ_{calc}g/cm^3	1.120	1.140
μ/mm^{-1}	1.477	2.121
F(000)	1612.0	1708.0
Crystal size/mm^3	0.325 × 0.18 × 0.118	0.272 × 0.226 × 0.194
Radiation	Cu Kα (λ = 1.54184)	Cu Kα (λ = 1.54184)
2Θ range for data collection/°	7.026 to 144.962	7.076 to 145.21
Index ranges	-12 ≤ h ≤ 15, -18 ≤ k ≤ 20, -26 ≤ l ≤ 19	-16 ≤ h ≤ 16, -28 ≤ k ≤ 28, -19 ≤ l ≤ 19
Reflections collected	29288	34888
Independent reflections	16686 [R_{int} = 0.0257, R_{sigma} = 0.0366]	8864 [R_{int} = 0.0638, R_{sigma} = 0.0445]
Data/restraints/parameters	16686/1859/1176	8864/1069/717
Goodness-of-fit on F^2	1.033	1.067
Final R indexes [I>=2σ (I)]	R_1 = 0.0468, wR_2 = 0.1191	R_1 = 0.0584, wR_2 = 0.1529
Final R indexes [all data]	R_1 = 0.0555, wR_2 = 0.1263	R_1 = 0.0622, wR_2 = 0.1569
Largest diff. peak/hole / e Å^{-3}	0.84/-0.42	0.77/-0.38
CCDC number	2407630	2407631

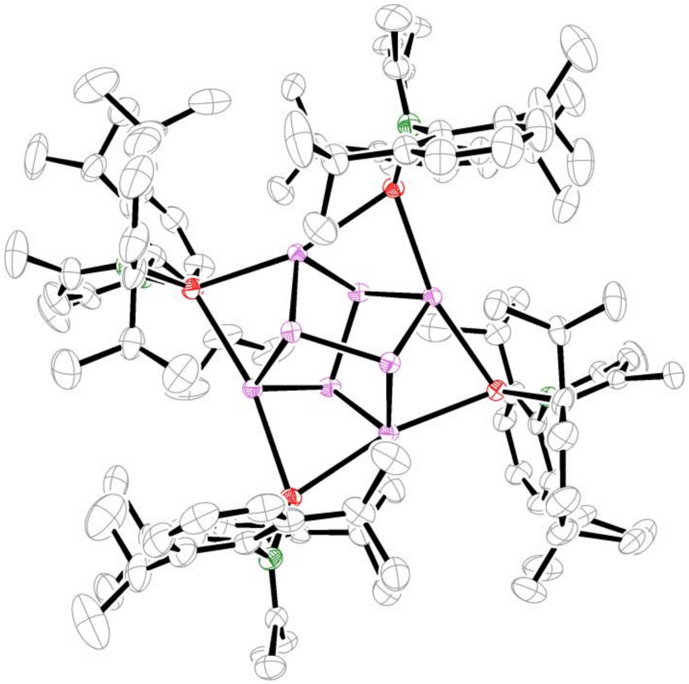

Figure S60. Solid state structure of $[(BDI)Mg]_4(P_8)$ (**1**). Ellipsoids represent 50% probability. Hydrogen atoms have been omitted for clarity.

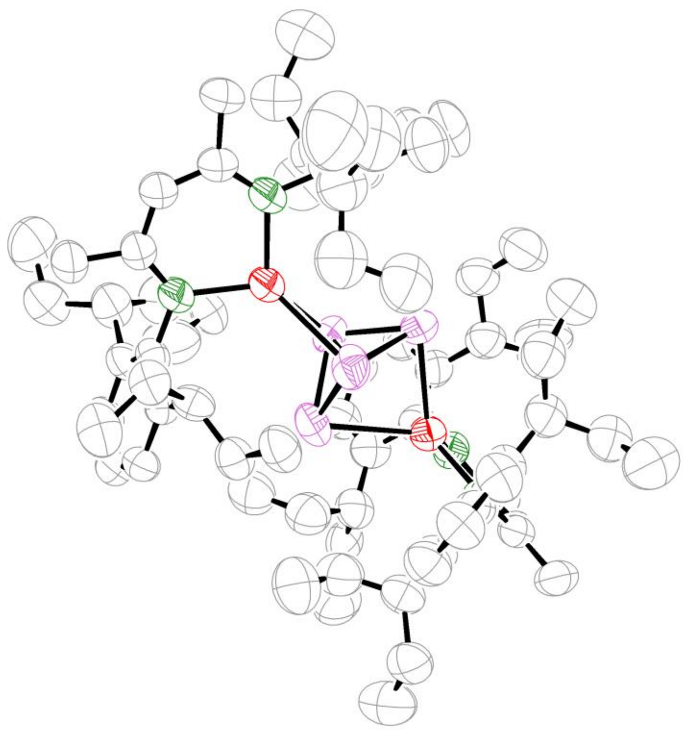

Figure S61. Solid state structure of $[(BDI^*)Mg]_2(P_4)$ (**2**). Ellipsoids represent 50% probability. Hydrogen atoms have been omitted for clarity. The structure is disordered. Only one disorder model is shown.

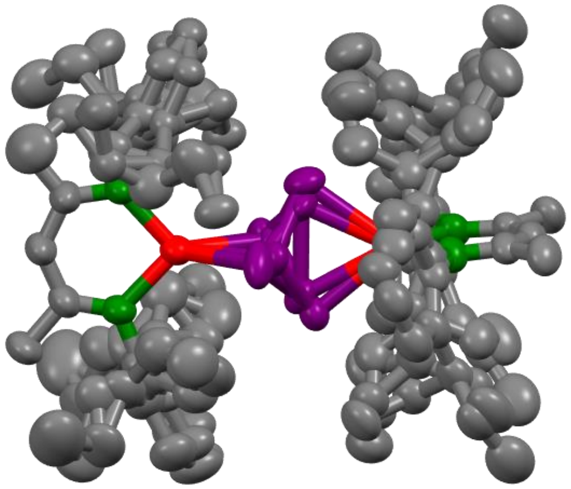

Figure S62. Solid state structure of $[(BDI^*)Mg]_2(P_4)$ (**2**) showing disorder in the butterfly-P_4^{2-} dianion.

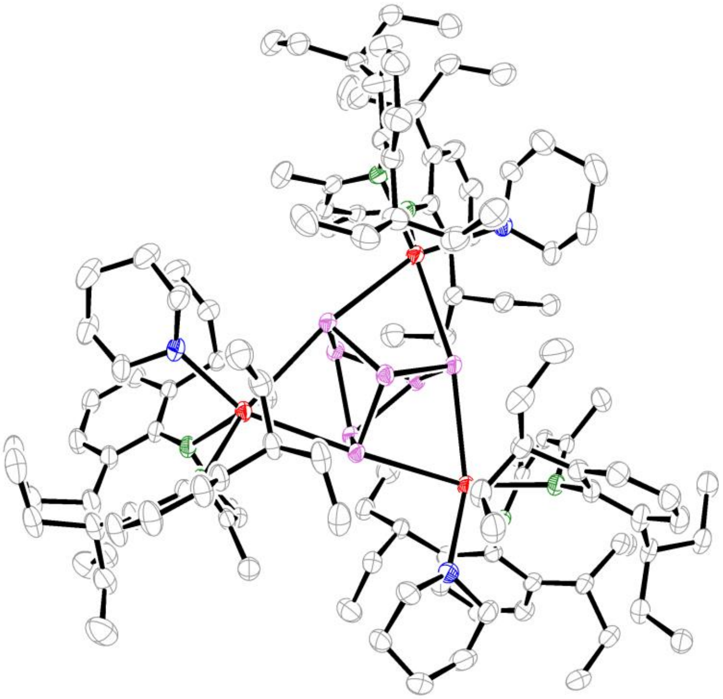

Figure S63. Solid state structure of [(BDI*)Ca(THP)]$_3$(P_7) (**3**). Ellipsoids represent 50% probability. Hydrogen atoms have been omitted for clarity.

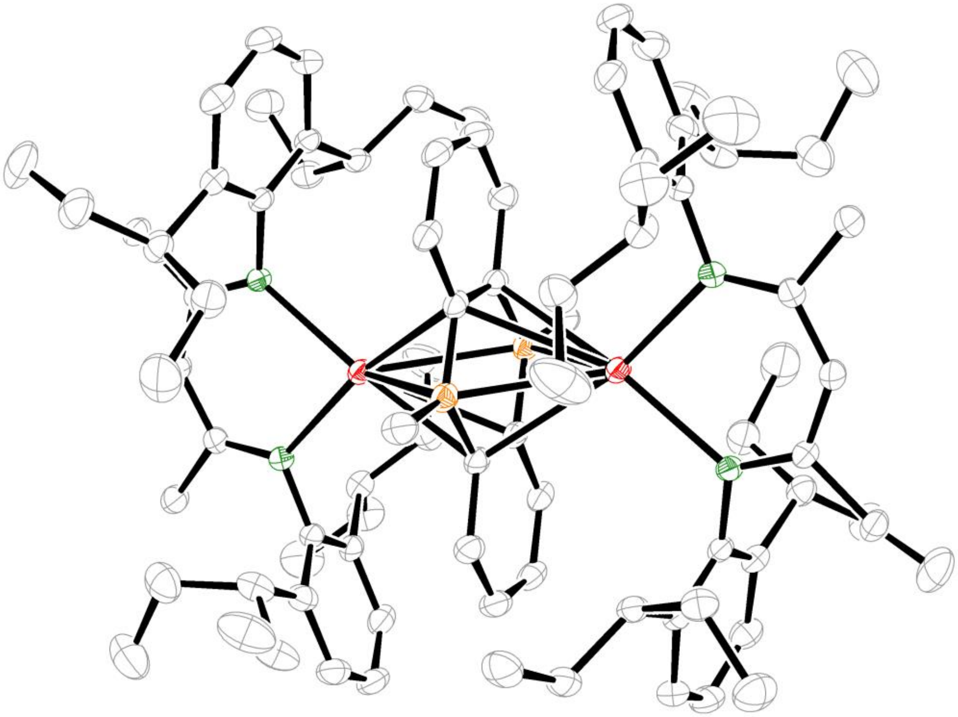

Figure S64. Solid state structure of [(BDI*)Ca]$_2$(DBA) (**4**) of one of the two independent molecules. Ellipsoids represent 50% probability. Hydrogen atoms have been omitted for clarity.

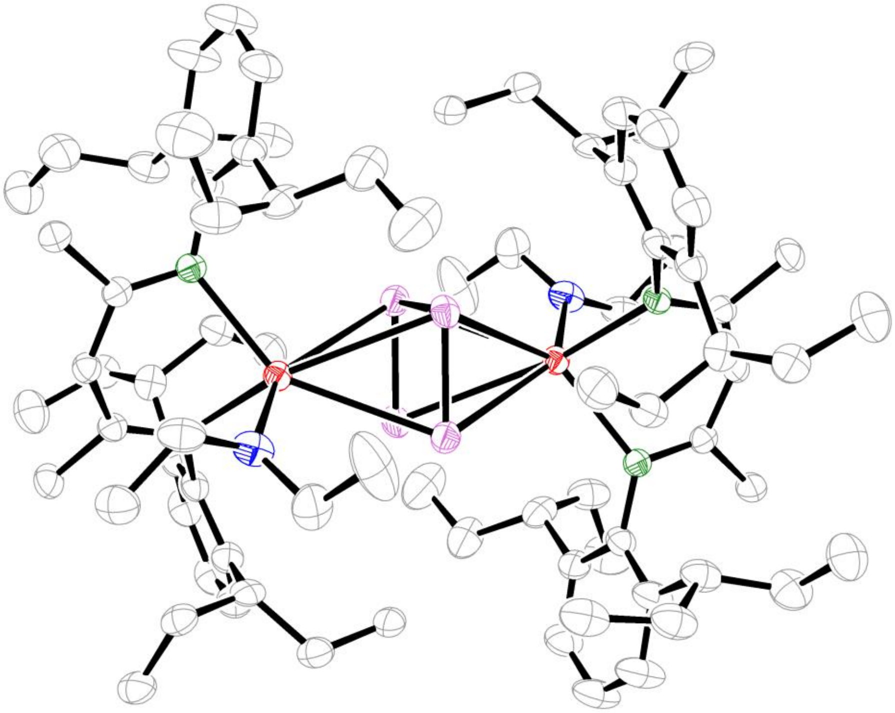

Figure S65. Solid state structure of [(BDI*)Ca(OEt$_2$)]$_2$(*cyclo*-P$_4$) (**5**). Ellipsoids represent 50% probability. Hydrogen atoms have been omitted for clarity.

6. Computational Details

Geometry optimizations, frequency calculations and PCM solvent corrections were run with Gaussian 16 Revision A.03[S13] using the PBE0[S14,S15] functional. For geometry optimizations, all atoms were described with def2-SVP basis sets of Ahlrichs and Weigand.[S16] Single point energy calculations were performed on the optimized geometries, at the PBE0/def2-TZVP level of theory. Stationary points were fully characterized using analytical frequency calculations as either minima (all positive eigenvalues) or transition states (one negative eigenvalue). IRC calculations and subsequent geometry optimizations were used to confirm the minima linked by the transition states. Energies reported in the text are based on the gas-phase free energies and incorporate a correction for dispersion effects using Grimme's D3 parameter set with Becke-Johnson dampening[S17,S18] (*i.e.* PBE0-D3BJ) as well as solvation (PCM approach) in benzene, toluene, or THF. Energies are given in atomic units (a.u.) unless otherwise stated. Natural Bond Orbital (NBO) and Natural Population Analysis (NPA) was performed using NBO-7 using the single point calculations performed at the PBE0/def2-TZVP level of theory.[S19] QTAIM analysis was calculated with a wavefunction produced at the PBE0/def2-TZVP level of theory using AIMAll-17.[S20,S21]

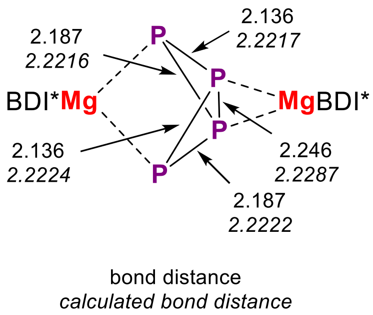

Figure S66. Comparison of P–P bond distances (crystal structure and calculated structure) in $[(BDI^*)Mg]_2(P_4)$ (**2**).

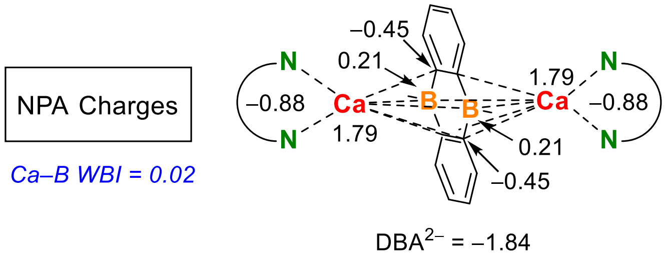

Figure S67. NPA charges of $[(BDI^*)Ca)]_2(DBA)$ (**4**).

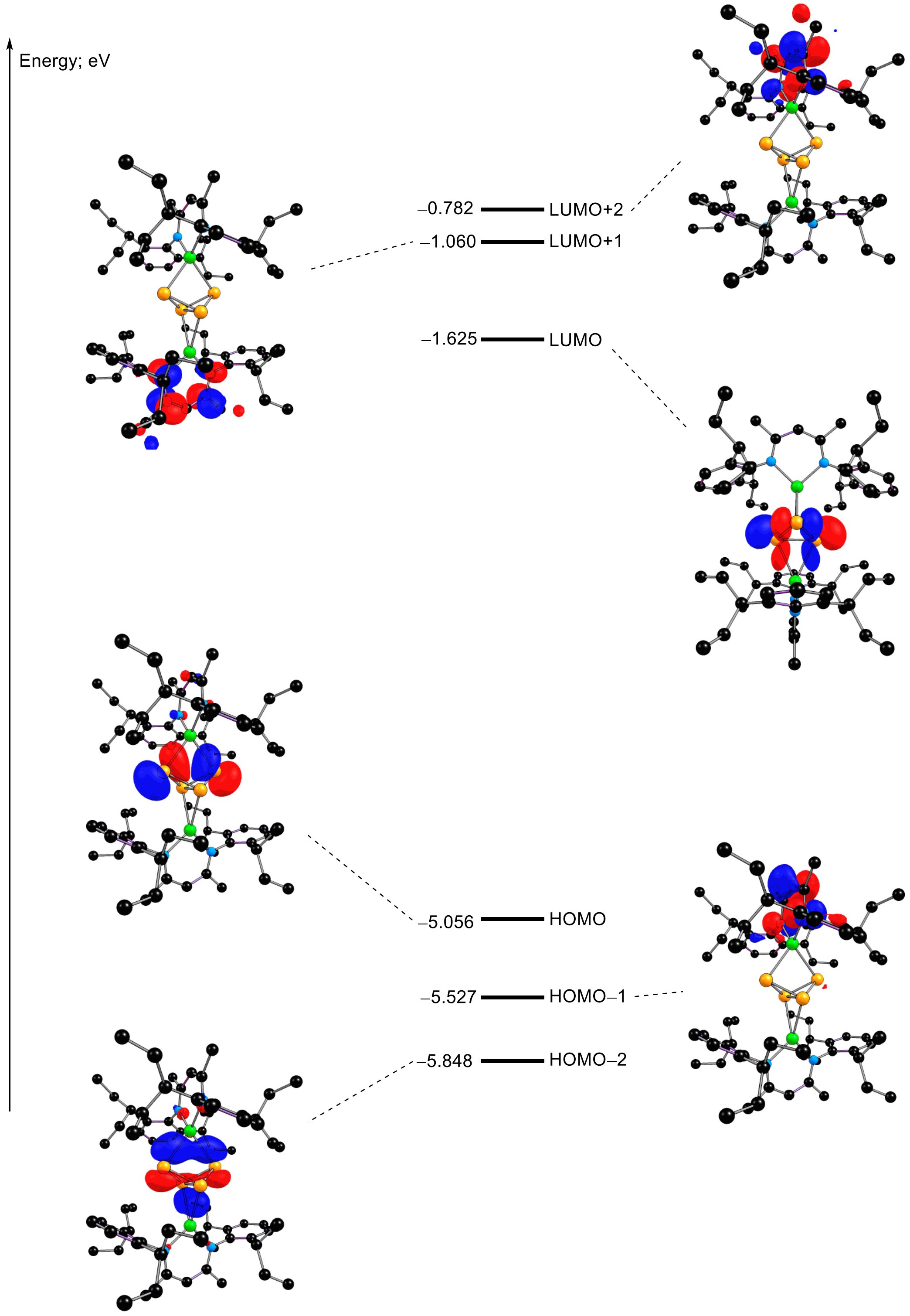

Figure S68. Kohn-Sham Molecular Orbital Diagram of $[(^{DIPeP}BDI)Mg]_2(P_4)$ (**2**) calculated at the PBE0-D3BJ/def2-TZVP level of theory.

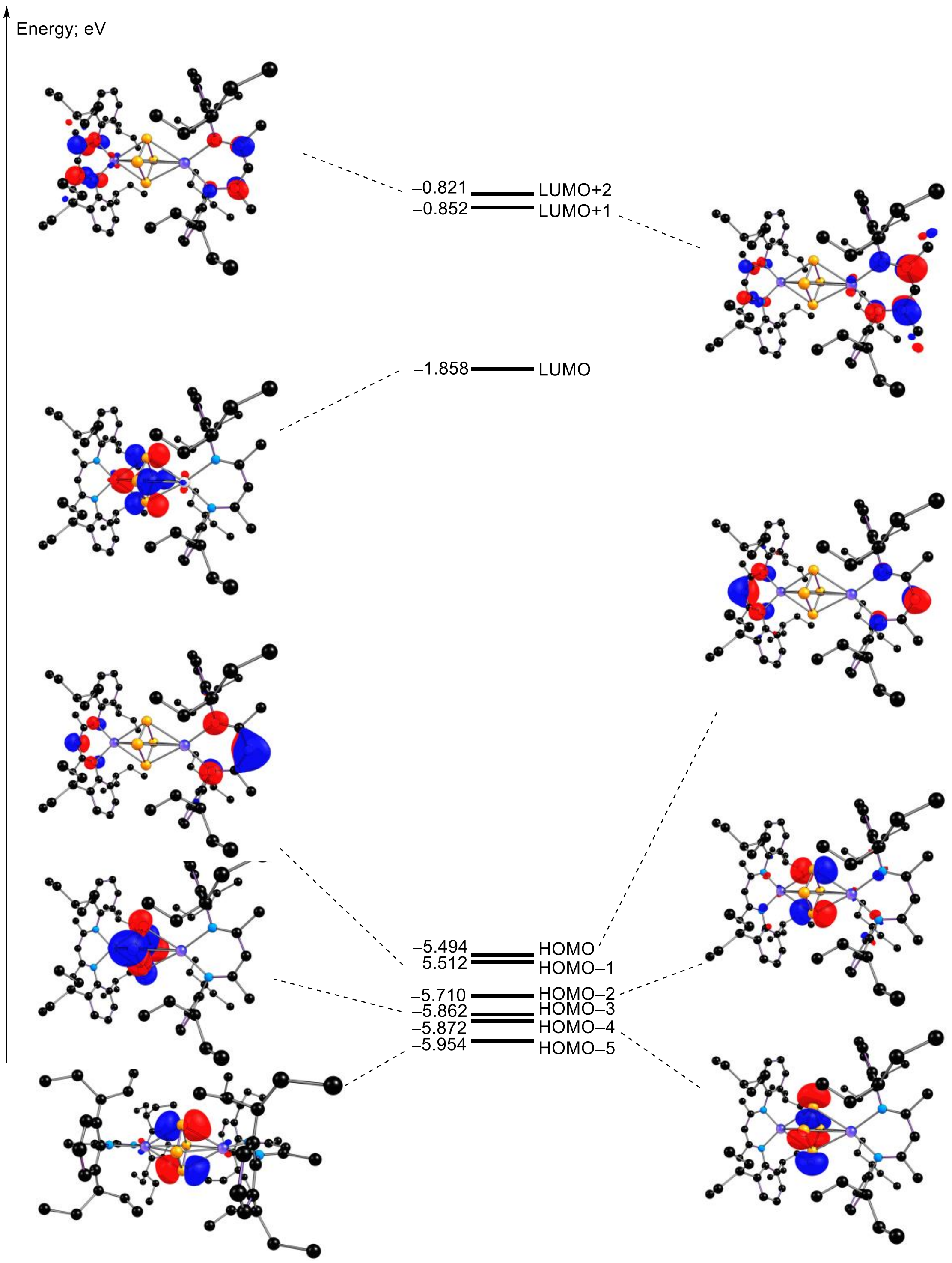

Figure S69. Kohn-Sham Molecular Orbital Diagram of $[(^{DIPeP}BDI)Ca]_2(\textit{cyclo}\text{-}P_4)$ (**5**) calculated at the PBE0-D3BJ/def2-TZVP level of theory.

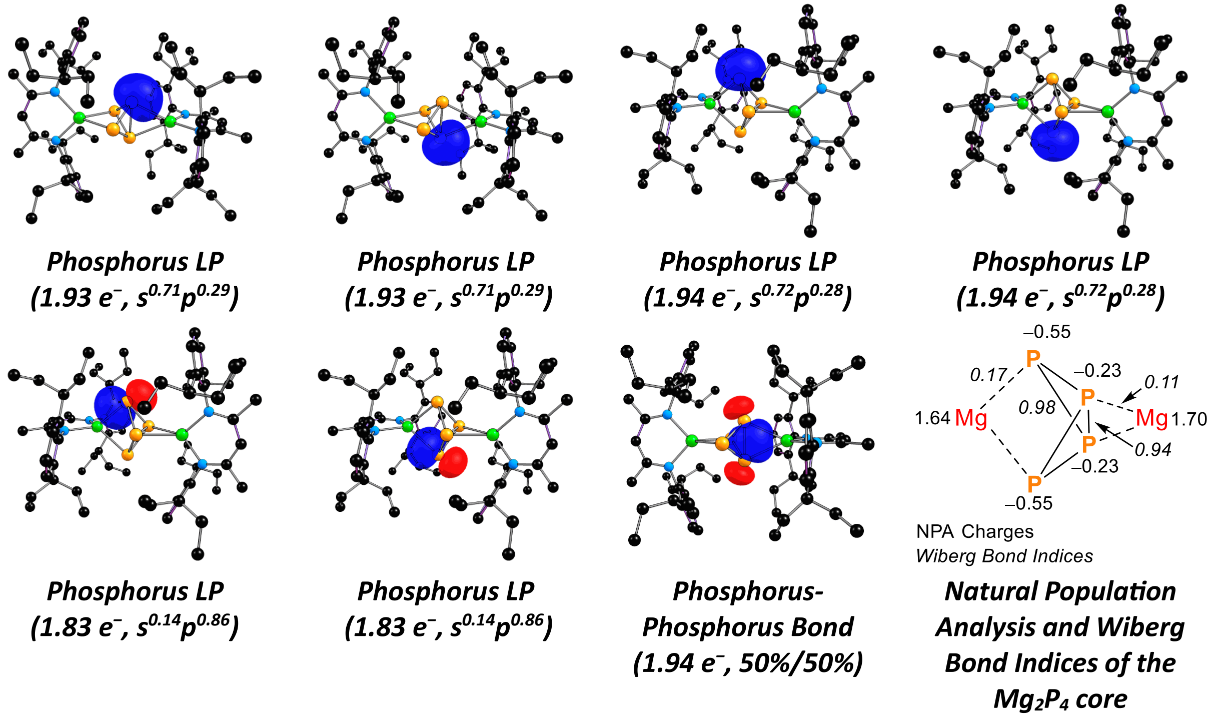

Figure S70. Natural Bond Orbital Analysis of [(^{DIPeP}BDI)Mg]$_2$(P_4) (**2**).

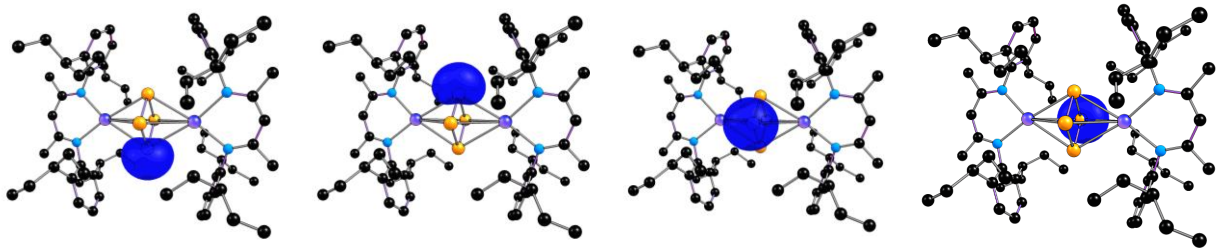

Phosphorus LP (1.95 e⁻, $s^{0.71}p^{0.29}$) | *Phosphorus LP (1.95 e⁻, $s^{0.71}p^{0.29}$)* | *Phosphorus LP (1.95 e⁻, $s^{0.71}p^{0.29}$)* | *Phosphorus LP (1.95 e⁻, $s^{0.71}p^{0.29}$)*

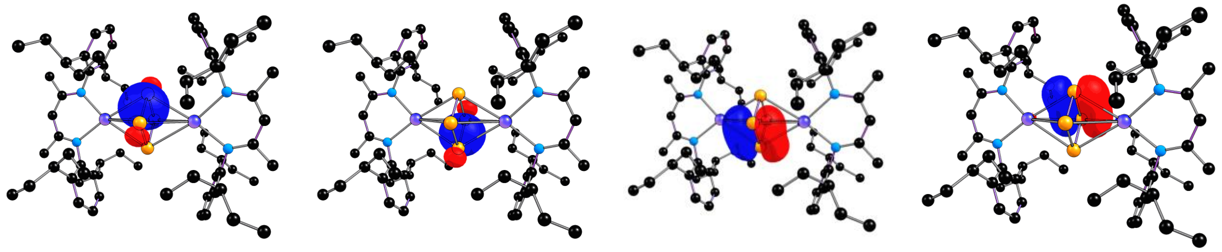

Phosphorus-Phosphorus Bond (1.98 e⁻, 49%/51%) | *Phosphorus-Phosphorus Bond (1.98 e⁻, 49%/51%)* | *Phosphorus-Phosphorus Bond (1.96 e⁻, 48%/52%)* | *Phosphorus-Phosphorus Bond (1.96 e⁻, 49%/51%)*

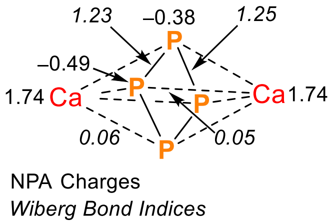

Natural Population Analysis and Wiberg Bond Indices of the Ca_2P_4 core

Figure S71. Natural Bond Orbital Analysis of [(^DIPeP^BDI)Ca]$_2$(*cyclo*-P$_4$) (**5**).

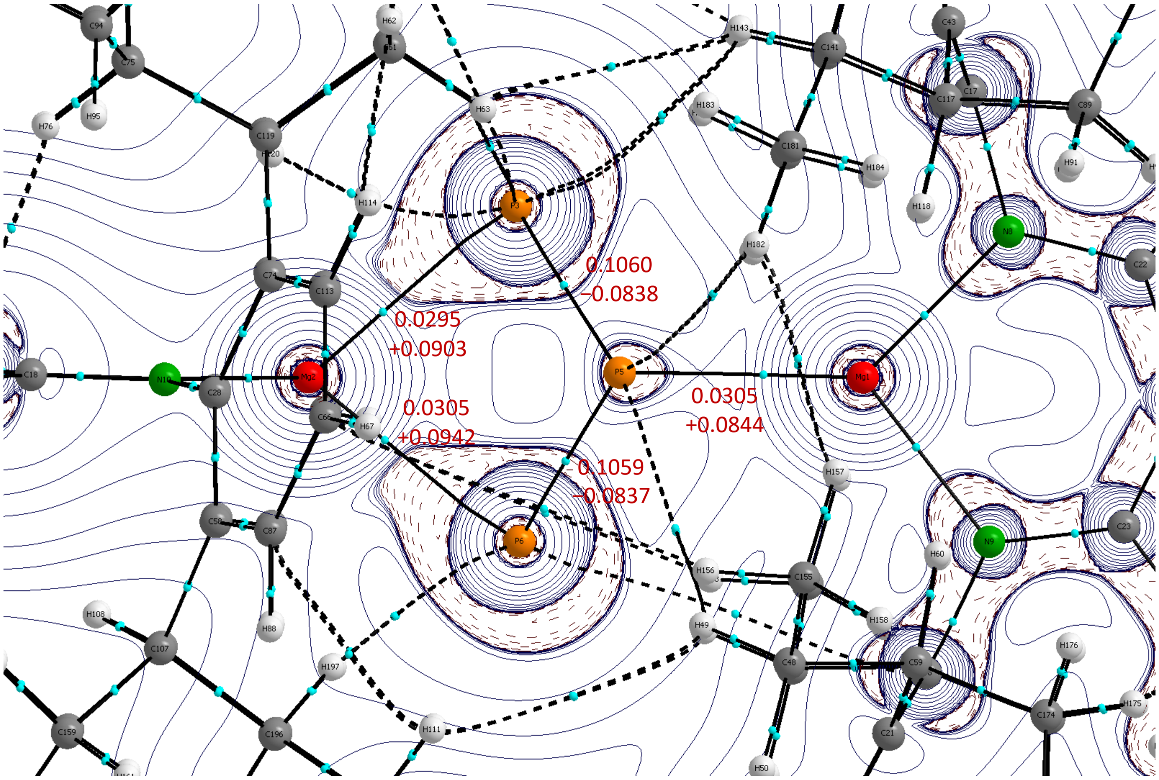

Figure S72. Laplacian of electron density contour plot of $[(^{DIPeP}BDI)Mg]_2(P_4)$ (**2**) in the Mg2, P3, Mg1 plane.

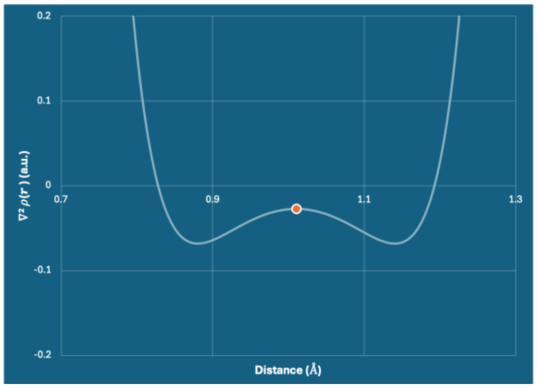

P4–P5

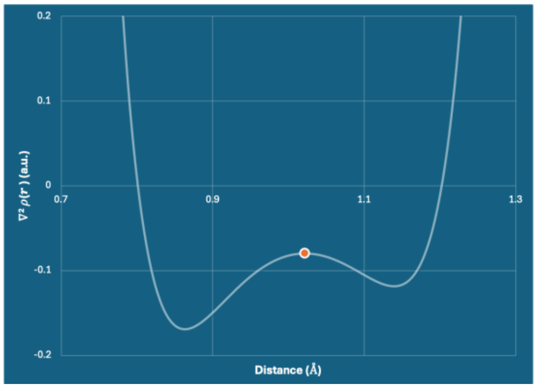

P4–P3

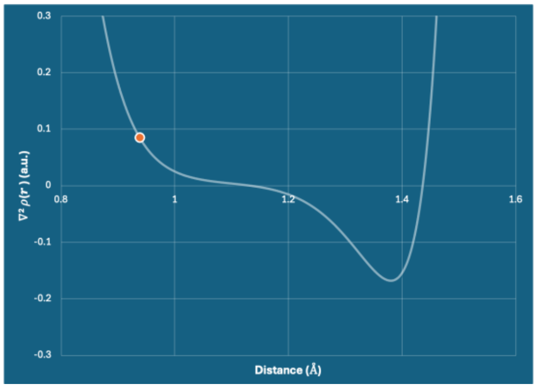

Mg1–P4

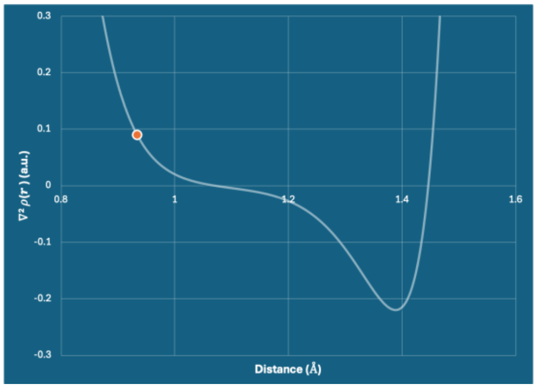

Mg2–P3

Figure S73. Selected graphical representations of the Laplacian for $[(^{DIPeP}BDI)Mg]_2(P_4)$ (**2**), orange dots denote the BCP.

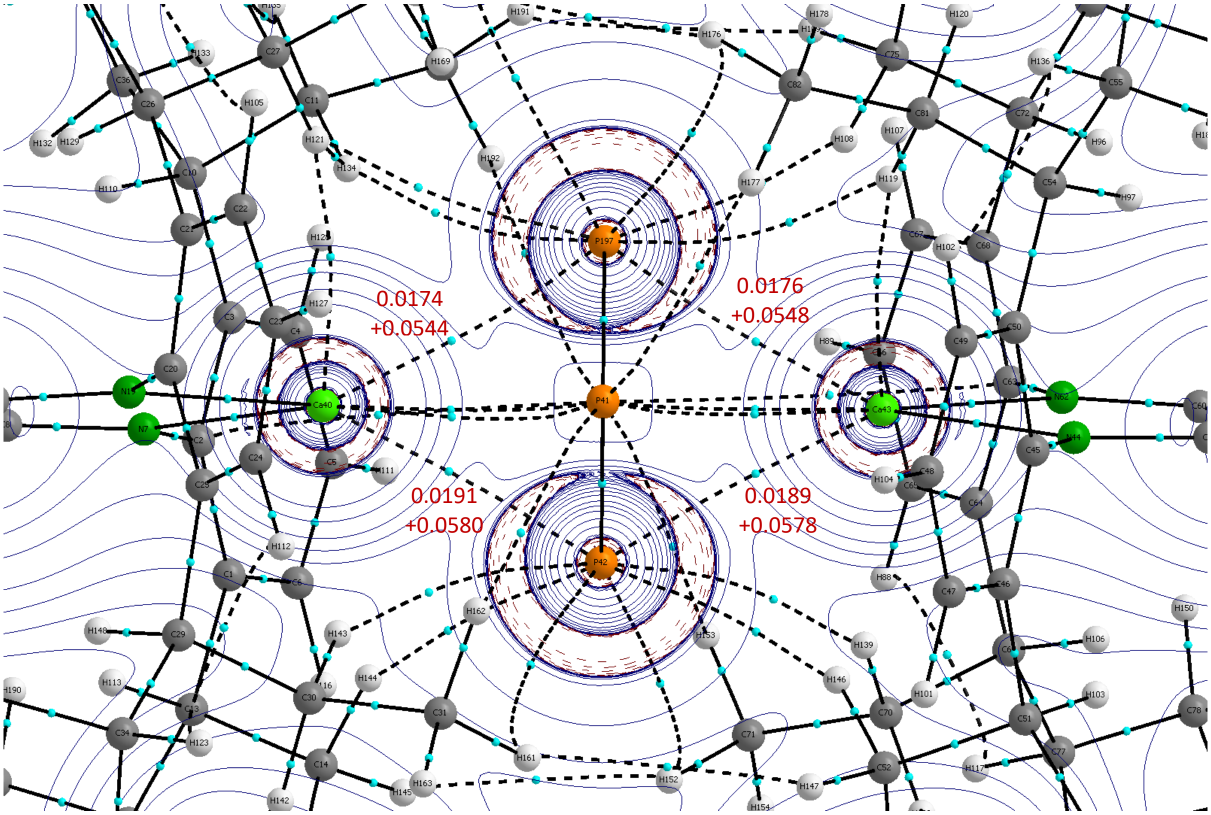

Figure S74. Laplacian of electron density contour plot of [(^{DIPeP}BDI)Ca]$_2$(*cyclo*-P_4) (**5**) in the Ca40, P42, Ca43 plane.

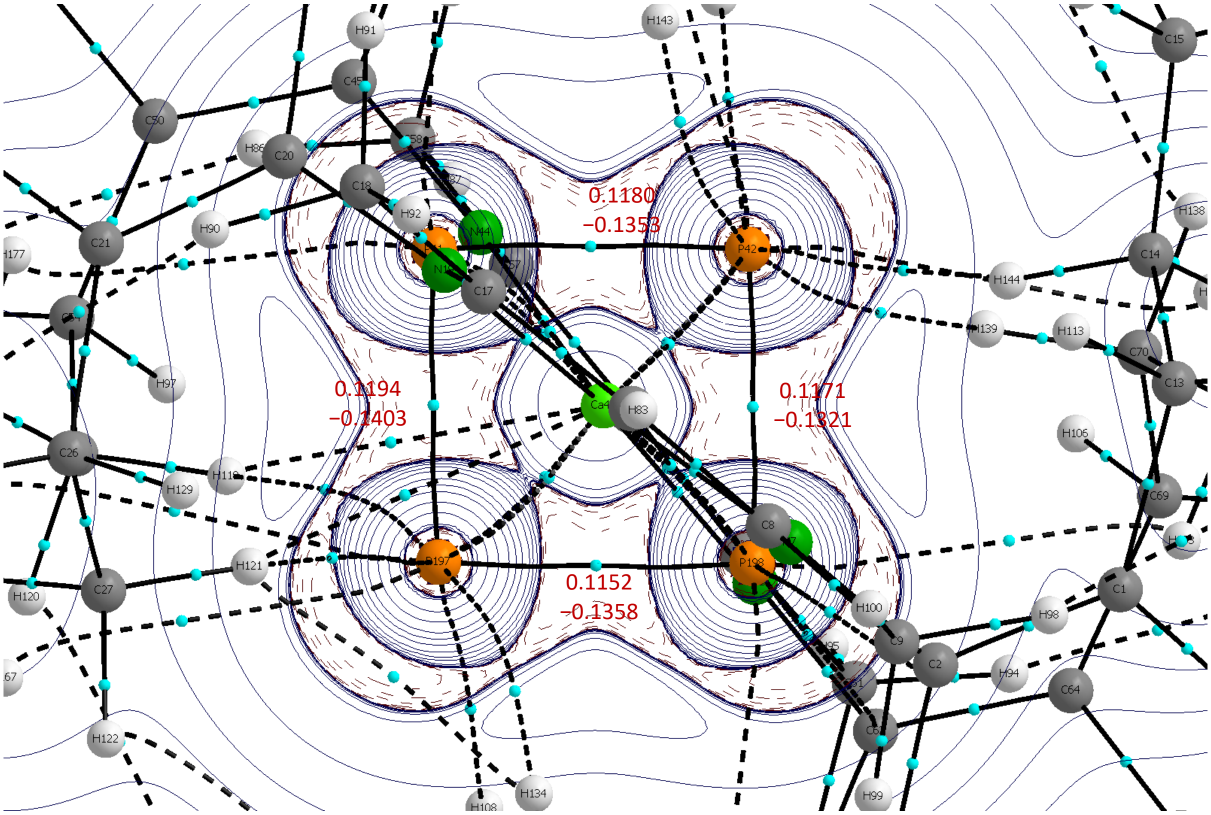

Figure S75. Laplacian of electron density contour plot of [(^{DIPeP}BDI)Ca]$_2$(*cyclo*-P$_4$) (**5**) in the P41, P42, P197 plane.

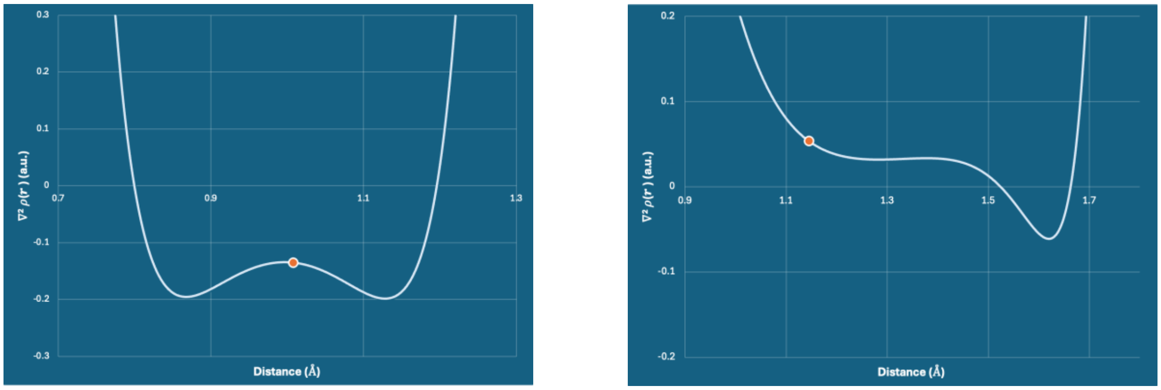

P41–P42 ***Ca40–P42***

Figure S76. Selected graphical representations of the Laplacian for [(^{DIPeP}BDI)Ca]$_2$(*cyclo*-P$_4$) (**5**), orange dots denote the BCP.

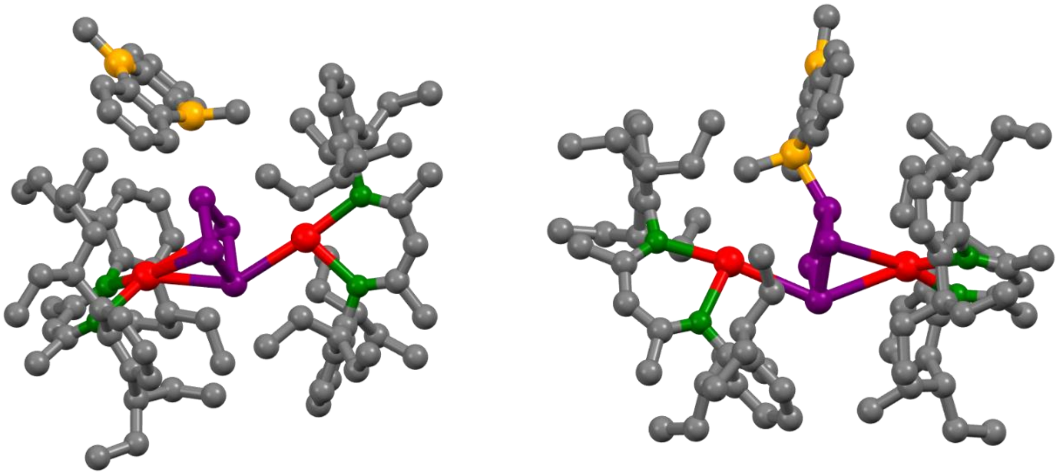

Figure S78. Calculated structures for interaction of DBA with complex **5:** TS (left) and adduct (right).

Table S2. Analysis of LCa(dianion)CaL systems by DFT at the PBE0-D3BJ/def2-TZVP (PCM = Cyclohexane) level of theory, triplet states were calculated adiabatically.

**Bridging Dianion**	**HOMO–LUMO Gap (eV)**	**Singlet–Triplet Gap (ΔH, kcal mol^{-1})**
Dinitrogen	2.60	7.57
Benzene	1.90	−2.00
Xylene	1.99	2.33
DBA	3.18	32.01

Computed Energies of Optimized Geometries

N2

SCF = -109.315115144

H(0 K) = -109.309353

H(298 K) = -109.306048

G(298 K) = -109.32779

SCF+D3 = -109.315466793

BS2 (def2-tzvp) = -109.315309944

Low Freq. = 2529.2366cm-1

Benzene

SCF = -231.802063851

H(0 K) = -231.700821

H(298 K) = -231.695510

G(298 K) = -231.72762

SCF+D3 = -231.811414477

BS2 (def2-tzvp) = -231.803071851

Low Freq. = 416.5064cm-1,416.6462cm-1

Xylene

SCF = -310.284453886

H(0 K) = -310.128724

H(298 K) = -310.119688

G(298 K) = -310.163127

SCF+D3 = -310.299440938

BS2 (def2-tzvp) = -310.285493805

Low Freq. = 18.5674cm-1,35.9219cm-1

DBA_Singlet

SCF = -590.533974346

H(0 K) = -590.289833

H(298 K) = -590.274706

G(298 K) = -590.332464

SCF+D3 = -590.568567324

BS2 (def2-tzvp) = -590.535487858

Low Freq. = 11.0532cm-1,62.5161cm-1

DBA(1-)_Doublet

SCF = -590.57656877

H(0 K) = -590.334926

H(298 K) = -590.319991

G(298 K) = -590.376216

SCF+D3 = -590.611077617

BS2 (def2-tzvp) = -590.611040821

Low Freq. = 41.9242cm-1,87.0279cm-1

P4_singlet

SCF = -1364.52612674

H(0 K) = -1364.519548

H(298 K) = -1364.514299

G(298 K) = -1364.548188

SCF+D3 = -1364.53354558

BS2 (def2-tzvp) = -1364.52622134

Low Freq. = 391.0122cm-1,391.2648cm-1

P4_triplet

SCF = -1364.45350278

H(0 K) = -1364.447953

H(298 K) = -1364.442273

G(298 K) = -1364.478168

SCF+D3 = -1364.46122507

BS2 (def2-tzvp) = -1364.45372429

Low Freq. = 244.3387cm-1,340.6686cm-1

P4(1-)_doublet

SCF = -1364.5239178

H(0 K) = -1364.518264

H(298 K) = -1364.512621

G(298 K) = -1364.54804

SCF+D3 = -1364.53141376

BS2 (def2-tzvp) = -1364.56503485

Low Freq. = 245.2526cm-1,329.5023cm-1

P4(2-)

SCF = -1364.36087647

H(0 K) = -1364.355751

H(298 K) = -1364.349858

G(298 K) = -1364.385211

SCF+D3 = -1364.3685811

BS2 (def2-tzvp) = -1364.52572422

Low Freq. = 246.17cm-1,255.5522cm-1

[(BDI*)Ca]2(P4) + DBA TS

SCF = -6411.63647742

H(0 K) = -6409.651519

H(298 K) = -6409.533994

G(298 K) = -6409.817043

SCF+D3 = -6412.02404241

BS2 (def2-tzvp) = -6411.64814487

Low Freq. = -52.0854cm-1,4.2235cm-1

[(BDI*)Ca]2(P4) + DBA adduct

SCF = -6411.64424575

H(0 K) = -6409.540761

H(298 K) = -6409.540761

G(298 K) = -6409.8204

SCF+D3 = -6412.03895762

BS2 (def2-tzvp) = -6411.65465819

Low Freq. = 6.1663cm-1,10.3594cm-1

[(BDI*)Mg]2(P4) (2)

SCF = -4866.20970634

H(0 K) = -4864.466733

H(298 K) = -4864.364872

G(298 K) = -4864.618461

BS2 (def2-tzvp) = -4870.02204508

Low Freq. = 3.0866cm-1, 10.9531cm-1

[(BDI*)Ca]2(P4) (5)

SCF = -5821.12403653

H(0 K) = -5819.383673

H(298 K) = -5819.281489

G(298 K) = -5819.530733

SCF+D3 = -5821.44735114

BS2 (def2-tzvp) = -5824.99143585

Low Freq. = 5.2783cm-1, 9.9504cm-1

[(BDI*)Ca]2(N2)_singlet

SCF = -4565.81471209

H(0 K) = -4564.074251

H(298 K) = -4563.975623

G(298 K) = -4564.218582

SCF+D3 = -4566.10762459

BS2 (def2-tzvp) = -4569.33806798

Low Freq. = 8.7765cm-1, 12.5782cm-1

[(BDI*)Ca]2(N2)_triplet

SCF = -4565.79787940

H(0 K) = -4564.058471

H(298 K) = -4563.959483

G(298 K) = -4564.204305

SCF+D3 = -4566.09177285

BS2 (def2-tzvp) = -4569.32275564

Low Freq. = 6.7891cm-1, 13.0235cm-1

[(BDI*)Ca]2(C6H6)_singlet

SCF = -4688.30038758

H(0 K) = -4686.470810

H(298 K) = -4686.368014

G(298 K) = -4686.618302

SCF+D3 = -4688.62241410

BS2 (def2-tzvp) = -4691.94394418

Low Freq. = 6.4863cm-1, 15.9861cm-1

[(BDI*)Ca]2(C6H6)_triplet

SCF = -4688.30422996

H(0 K) = -4686.474168

H(298 K) = -4686.371623

G(298 K) = -4686.622210

SCF+D3 = -4688.62618724

BS2 (def2-tzvp) = -4691.94717155

Low Freq. = 6.4889cm-1, 16.2065cm-1

[(BDI*)Ca]2(p-Xyl)_singlet

SCF = -4766.77515489

H(0 K) = -4764.889219

H(298 K) = -4764.783943

G(298 K) = -4765.036270

SCF+D3 = -4767.11142095

BS2 (def2-tzvp) = -4770.50072746

Low Freq. = 7.4563cm-1, 13.3623cm-1

[(BDI*)Ca]2(p-Xyl)_triplet

SCF = -4766.77336553

H(0 K) = -4764.887390

H(298 K) = -4764.781907

G(298 K) = -4765.037036

SCF+D3 = -4767.11022714

BS2 (def2-tzvp) = -4770.49696313

Low Freq. = 3.2154cm-1, 12.6116cm-1

[(BDI*)Ca]2(DBA)_singlet (4)

SCF = -5047.10156331

H(0 K) = -5045.121866

H(298 K) = -5045.011328

G(298 K) = -5045.274688

SCF+D3 = -5047.47997381

BS2 (def2-tzvp) = -5051.11082005

Low Freq. = 9.0756cm-1, 13.6697cm-1

[(BDI*)Ca]2(DBA)_triplet (4)

SCF = -5047.04698145

H(0 K) = -5045.070626

H(298 K) = -5044.959533

G(298 K) = -5045.225569

SCF+D3 = -5047.42444782

BS2 (def2-tzvp) = -5051.05676215

Low Freq. = 8.4146cm-1, 13.4080cm-1

XYZ Coordinates

2

N2

N	0.000000000	0.000000000	0.548897000
N	0.000000000	0.000000000	-0.548897000

12

Benzene

C	0.000000000	0.000000000	1.395033000
H	0.000000000	0.000000000	2.488082000
C	0.000000000	1.208057000	0.697446000
C	0.000000000	1.208109000	-0.697480000
C	0.000000000	0.000000000	-1.394983000
C	0.000000000	-1.208109000	-0.697480000
C	0.000000000	-1.208057000	0.697446000
H	0.000000000	2.154685000	1.243947000
H	0.000000000	2.154717000	-1.243927000
H	0.000000000	0.000000000	-2.488011000
H	0.000000000	-2.154717000	-1.243927000
H	0.000000000	-2.154685000	1.243947000

18

Xylene

C	2.924643000	-0.005458000	0.000000000
H	3.335205000	1.014242000	-0.000001000
H	3.322094000	-0.527230000	0.885550000
C	1.422516000	0.005299000	0.000000000
H	3.322094000	-0.527231000	-0.885549000
C	0.695088000	-1.193385000	0.000000000
C	-0.695099000	-1.193383000	0.000000000
C	-1.422492000	0.005314000	0.000000000
C	-0.697726000	1.199320000	0.000000000
C	0.697722000	1.199308000	0.000000000
H	1.231837000	-2.147055000	0.000000000
H	-1.231854000	-2.147049000	0.000000000
C	-2.924625000	-0.005464000	0.000000000
H	-1.234387000	2.152632000	0.000000000
H	1.234390000	2.152614000	0.000000000
H	-3.335256000	1.014330000	0.000000000
H	-3.322138000	-0.527279000	-0.885642000
H	-3.322138000	-0.527279000	0.885642000

30

DBA

C	-0.366040	-0.483868	-2.953424
C	-0.895352	-1.370790	-2.002487
C	-1.529973	-2.559745	-2.456419
C	-1.602288	-2.800097	-3.837749
C	-1.061316	-1.910789	-4.765636
C	-0.439721	-0.746171	-4.320998
B	-0.787018	-1.056487	-0.464906
C	-0.152871	0.310523	0.002062
B	-2.137190	-3.586331	-1.430686
C	-2.908088	-4.853141	-1.969040
C	-1.936251	-3.322775	0.105549
C	-1.300222	-2.131232	0.560467
C	-1.135540	-1.942399	1.940714
C	-1.566680	-2.890688	2.869413
C	-2.187705	-4.054063	2.425251
C	-2.371313	-4.257459	1.056847
H	-0.194838	0.511291	1.081003
H	0.910575	0.342575	-0.297650
H	-0.628844	1.160295	-0.515993
H	-3.391396	-5.476995	-1.205232
H	-3.680013	-4.560470	-2.700802
H	-2.205799	-5.499843	-2.526149
H	0.121229	0.437089	-2.622322
H	-0.014135	-0.040897	-5.039920
H	-1.126668	-2.125307	-5.835727
H	-2.090557	-3.706894	-4.204540
H	-0.653675	-1.033095	2.308038
H	-2.864568	-5.174874	0.726697
H	-2.532001	-4.803378	3.143084
H	-1.419154	-2.718736	3.938977

30

DBA(1-)_doublet

C	-2.574119000	-1.383302000	0.000022000
C	-1.321951000	-0.719663000	-0.000015000
C	-1.321939000	0.719698000	-0.000020000
C	-2.574107000	1.383340000	-0.000022000
C	-3.781814000	0.703742000	0.000006000
C	-3.781822000	-0.703697000	0.000040000
B	0.008841000	-1.519311000	-0.000029000
C	-0.034956000	-3.123621000	-0.000048000
B	0.008872000	1.519320000	-0.000014000
C	-0.034820000	3.123638000	0.000007000
C	1.338602000	0.722611000	-0.000008000
C	1.338585000	-0.722638000	-0.000003000
C	2.593836000	-1.382239000	0.000019000
C	3.801179000	-0.704243000	0.000031000
C	3.801194000	0.704176000	0.000007000
C	2.593861000	1.382192000	-0.000012000
H	0.952721000	-3.611325000	-0.000314000
H	-0.581377000	-3.508998000	0.880872000
H	-0.581943000	-3.509094000	-0.880560000
H	0.952907000	3.611239000	0.000364000
H	-0.581193000	3.509177000	-0.880861000
H	-0.581764000	3.509044000	0.880580000
H	-2.590642000	-2.478606000	0.000041000
H	-4.728443000	-1.254910000	0.000081000
H	-4.728431000	1.254962000	0.000002000
H	-2.590640000	2.478643000	-0.000052000
H	2.612823000	-2.477026000	0.000030000

H 2.612853000 2.476978000 -0.000041000
H 4.747106000 1.256466000 -0.000003000
H 4.747080000 -1.256553000 0.000058000

4
P4_singlet
P 0.508345000 -1.107375000 0.575970000
P -0.334408000 0.890136000 0.955024000
P 0.922483000 0.544247000 -0.818284000
P -1.096419000 -0.327008000 -0.712710000

4
P4_triplet
P -0.000634000 1.124046000 0.558702000
P 0.000705000 -1.123986000 0.558761000
P -1.527458000 -0.000523000 -0.558706000
P 1.527387000 0.000463000 -0.558758000

4
P4(1-)_doublet
P 0.001871000 -1.082260000 -0.661253000
P 1.419065000 0.000199000 0.660741000
P -0.000813000 1.083908000 -0.659244000
P -1.420123000 -0.001847000 0.659756000

4
P4(2-)
P 0.000176000 -1.064018000 -0.537233000
P 1.684079000 -0.000683000 0.536337000
P -0.000327000 1.065703000 -0.535536000
P -1.683928000 -0.001001000 0.536432000

198
[(BDI*)Ca]2(P4) (5)
C 3.489760 -3.481219 -1.678788
C 3.815431 -2.946515 -0.406774
C 3.537021 -3.686622 0.771141
C 2.884154 -4.917513 0.651725
C 2.509079 -5.420723 -0.588500
C 2.821002 -4.707754 -1.739269
N 4.357722 -1.635931 -0.307543
C 5.676308 -1.469911 -0.301283
C 6.568242 -2.685311 -0.375400
C 3.952844 -3.168397 2.138136
C 2.815251 -2.418659 2.857644
C 1.583256 -3.226718 3.230563
C 3.824410 -2.743477 -2.966557
C 2.589648 -1.975897 -3.465913
C 2.825043 -1.043369 -4.644429
C 6.338196 -0.227867 -0.217862
C 5.836075 1.079734 -0.061599
C 6.870125 2.177294 -0.000690
N 4.549622 1.398451 0.044608
C 4.177407 2.750702 0.272871
C 4.056655 3.226973 1.605190
C 3.568156 4.520835 1.811138
C 3.216285 5.340492 0.745070
C 3.376197 4.880316 -0.556076
C 3.862852 3.596142 -0.821703
C 4.441648 2.349284 2.787752
C 3.250300 1.527070 3.315552
C 2.114425 2.304734 3.958047
C 4.047119 3.124918 -2.256859
C 2.776772 2.467134 -2.828315
C 1.540689 3.343686 -2.943601
C 5.133653 3.104477 3.931058
C 6.461199 3.748682 3.563766
C 4.574112 4.209684 -3.207079
C 5.946344 4.761514 -2.853639
C 4.593842 -4.263952 3.004912
C 5.265182 -3.746759 4.269034
C 4.388659 -3.655686 -4.066071
C 5.654227 -4.416314 -3.703387
Ca 2.679479 -0.017435 -0.042899
P 0.039063 1.621297 0.062888
P -0.002221 0.077930 -1.476553
Ca -2.644434 0.063519 0.043081
N -4.453356 1.548573 -0.179856
C -4.047009 2.906095 -0.308051
C -3.787877 3.455233 -1.589321
C -3.255806 4.746129 -1.672385
C -3.008762 5.503742 -0.535318
C -3.311060 4.977401 0.715098
C -3.833078 3.688390 0.857603
C -4.035270 2.672117 -2.869984
C -2.712958 2.062836 -3.365164
C -2.815730 1.159037 -4.584356
C -4.146156 3.139417 2.241598
C -4.735906 4.175737 3.209071
C -6.064539 4.775831 2.776584
C -5.750299 1.254882 -0.149042
C -6.755908 2.373309 -0.269929
C -6.293366 -0.038560 -0.008774
C -5.683253 -1.301078 0.141275
C -6.627920 -2.473180 0.242855
N -4.373512 -1.522263 0.197853
C -3.890886 -2.854128 0.331689
C -3.609924 -3.620970 -0.829963
C -3.002728 -4.870865 -0.677910
C -2.681784 -5.374410 0.577243
C -3.000550 -4.637415 1.710022
C -3.619548 -3.386566 1.617309
C -3.945184 -3.098927 -2.219481
C -2.778087 -2.313650 -2.846481
C -1.486550 -3.076770 -3.088277
C -3.950133 -2.625920 2.892923

C	-4.588653	-3.503664	3.980539
C	-5.874274	-4.213152	3.586460
C	-2.687815	-1.925524	3.421952
C	-2.887588	-1.026822	4.632860
C	-4.457359	-4.176774	-3.185945
C	-5.747932	-4.860113	-2.761812
C	-4.707405	3.500606	-3.975685
C	-6.048008	4.120266	-3.614889
C	-2.932453	2.432914	2.874573
C	-1.696006	3.281347	3.117826
H	7.424949	-0.291570	-0.262705
H	-7.382343	-0.068237	-0.019038
H	-6.605322	2.931825	-1.204154
H	-6.625462	3.101904	0.543392
H	-7.785314	1.996511	-0.243917
H	-2.775907	-5.467566	-1.563836
H	-2.194911	-6.347951	0.671642
H	6.811143	2.716491	0.955825
H	6.685947	2.927087	-0.783740
H	7.886116	1.782557	-0.120804
H	-6.463623	-3.023818	1.179481
H	-6.442718	-3.192361	-0.568111
H	-7.675894	-2.153622	0.198133
H	-4.677122	-1.837132	2.633346
H	-4.911849	2.356825	2.114700
H	6.322230	-3.303234	-1.249917
H	6.412445	-3.327573	0.505703
H	7.627984	-2.407137	-0.421062
H	-3.035984	5.170990	-2.654937
H	-3.138170	5.587014	1.604378
H	-4.710622	1.834226	-2.625294
H	-2.590879	6.509463	-0.622759
H	3.468271	4.901147	2.830023
H	-4.761939	-2.368089	-2.102619
H	-2.769018	-5.047286	2.696271
H	-2.240128	-1.330457	2.606509
H	-1.923741	-2.691449	3.644333
H	4.736409	-2.412538	1.967874
H	1.985577	-6.377205	-0.658213
H	3.124220	5.540687	-1.388453
H	4.598409	-1.994000	-2.728528
H	4.459448	3.869905	4.349465
H	5.294745	2.384414	4.752465
H	2.546652	-5.119755	-2.713608
H	-3.679146	-4.938373	-3.359359
H	-4.608738	-3.693641	-4.166978
H	-2.636488	1.577024	2.239673
H	-3.261857	1.977590	3.823893
H	2.822777	0.932896	2.487404
H	3.639703	0.782083	4.031280
H	3.852275	5.040006	-3.279733
H	4.609469	3.771374	-4.219680
H	3.607720	-4.366667	-4.389183
H	4.593226	-3.032820	-4.951724
H	2.830073	6.345848	0.928911
H	2.665270	-5.498198	1.549962
H	5.166911	1.608259	2.414995
H	-4.792946	-2.865040	4.854878
H	-3.849243	-4.244620	4.332745
H	5.338646	-4.794715	2.387714
H	3.846079	-5.026547	3.279410
H	2.482718	-1.580386	2.219454
H	3.229526	-1.936282	3.758929
H	-4.010645	4.985328	3.393951
H	-4.864317	3.677789	4.185931
H	-3.133872	-1.879759	-3.796458
H	-2.543006	-1.442846	-2.207541
H	-4.013183	4.290006	-4.314471
H	-4.846223	2.846442	-4.851325
H	3.027613	2.049002	-3.818275
H	2.512869	1.589221	-2.210856
H	2.168398	-1.390815	-2.629619
H	1.796589	-2.703713	-3.712903
H	-2.249990	1.493876	-2.539839
H	-1.998626	2.881593	-3.563756
H	4.807606	2.327055	-2.236109
H	-6.053811	-5.622212	-3.495031
H	-5.640486	-5.360953	-1.787124
H	-6.575015	-4.136881	-2.677881
H	-0.730897	-2.410656	-3.532198
H	-1.065973	-3.472616	-2.151031
H	-1.630065	-3.920085	-3.782126
H	-1.864194	0.632309	-4.754252
H	-3.598753	0.394665	-4.452493
H	-3.050440	1.718083	-5.502644
H	-6.449997	4.706580	-4.455534
H	-6.794197	3.346843	-3.372409
H	-5.967439	4.794484	-2.748204
H	0.699116	2.761606	-3.350253
H	1.225316	3.733480	-1.963723
H	1.704038	4.199525	-3.617342
H	6.261428	5.534010	-3.571899
H	5.954867	5.216733	-1.851099
H	6.712187	3.969333	-2.868582
H	1.309878	1.618366	4.262075
H	2.443966	2.848867	4.856750
H	1.677826	3.033953	3.258665
H	6.899127	4.275765	4.425341
H	7.194458	2.995459	3.233628
H	6.344268	4.482083	2.750695
H	-6.427459	5.509518	3.512729
H	-5.980716	5.291422	1.807290
H	-6.840952	4.000205	2.677832
H	-0.901399	2.673861	3.575484
H	-1.295327	3.692511	2.178659
H	-1.901701	4.121256	3.799821

H	-6.252384	-4.832162	4.414654
H	-6.667562	-3.492275	3.331879
H	-5.727658	-4.873647	2.717892
H	-1.978788	-0.437712	4.829677
H	-3.715961	-0.317182	4.474032
H	-3.112573	-1.597827	5.546187
H	1.930179	-0.432107	-4.837633
H	3.662268	-0.353550	-4.447942
H	3.056309	-1.588194	-5.572169
H	5.986331	-5.051577	-4.538980
H	6.481637	-3.727465	-3.469474
H	5.502364	-5.069236	-2.829915
H	0.851136	-2.586172	3.746122
H	1.086050	-3.642163	2.340717
H	1.825300	-4.060809	3.908640
H	5.783619	-4.559023	4.801123
H	6.013958	-2.971626	4.036985
H	4.543057	-3.306903	4.974098
P	0.038860	0.067237	1.581865
P	-0.003356	-1.483082	0.051108

228

[(BDI*Ca)(P4) + DBA TS

C	0.936724	4.886119	3.142245
C	0.352807	4.360636	1.991376
C	0.626586	4.892126	0.724163
C	1.506073	6.003231	0.617389
C	2.050014	6.544393	1.797211
C	1.783679	5.991433	3.047152
B	1.901463	6.567566	-0.791672
C	1.505731	5.738726	-2.067699
C	0.621693	4.630749	-1.962560
B	-0.092809	4.370818	-0.578985
C	2.017601	6.071851	-3.336580
C	1.705338	5.327656	-4.470679
C	0.852492	4.228351	-4.357193
C	0.310146	3.897998	-3.117142
C	-1.661124	4.207331	-0.559251
C	2.751319	7.890867	-0.942819
C	4.017412	2.696359	-3.160828
C	3.357316	1.329397	-3.248400
C	4.307109	0.161591	-3.555844
C	5.075834	0.400437	-4.866767
C	6.070856	-0.684401	-5.246829
C	3.576382	-1.171056	-3.560358
C	3.729445	-2.106700	-2.509781
C	3.202345	-3.421649	-2.639682
C	2.420466	-3.716690	-3.758990
C	2.175438	-2.764698	-4.745512
C	2.770470	-1.514040	-4.652032
N	4.323251	-1.716488	-1.282342
C	5.573541	-2.049871	-0.984577
C	6.476719	-2.649914	-2.038941
C	3.512116	-4.485667	-1.595730
C	3.693224	-5.893497	-2.178898
C	4.853203	-6.032438	-3.153263
C	6.183894	-1.856086	0.269224
C	5.671162	-1.303784	1.457858
C	6.628004	-1.304062	2.628440
N	4.438771	-0.835126	1.600978
C	4.038385	-0.124793	2.759831
C	3.266865	-0.755040	3.764741
C	2.668179	0.043383	4.745602
C	2.855465	1.419175	4.770700
C	3.704836	2.015659	3.842561
C	4.327951	1.265276	2.842552
C	3.114546	-2.266257	3.822674
C	3.447440	-2.828952	5.214699
C	4.829824	-2.485059	5.745856
C	5.331425	1.895045	1.884740
C	4.713239	2.313958	0.540867
C	3.697550	3.435964	0.601727
C	1.716057	-2.706676	3.365268
C	1.503703	-4.211365	3.282260
C	6.142987	3.041682	2.501735
C	7.024346	2.629723	3.671587
C	2.509313	-4.487316	-0.429413
C	1.094495	-4.911844	-0.769350
C	-3.014751	2.155225	-3.939648
C	-3.793568	1.145929	-3.112737
C	-5.182958	1.591696	-2.611855
C	-6.043876	2.104155	-3.773668
C	-7.515476	2.300572	-3.444712
C	-5.097753	2.540293	-1.423494
C	-5.058441	2.026253	-0.101818
C	-5.051703	2.899326	1.016810
C	-5.042359	4.277730	0.782461
C	-5.055891	4.791639	-0.509636
C	-5.089261	3.927547	-1.598315
N	-5.050056	0.617881	0.103138
C	-6.238613	0.028167	0.236878
C	-7.494214	0.864099	0.160755
C	-5.092583	2.336713	2.431717
C	-5.893656	3.183726	3.429486
C	-7.377726	3.312975	3.123685
C	-6.454416	-1.346863	0.432972
C	-5.557286	-2.428461	0.494936
C	-6.205563	-3.786467	0.630685
N	-4.230367	-2.340535	0.422988
C	-3.511603	-3.569696	0.441479
C	-3.054982	-4.103820	1.669695
C	-2.322884	-5.295562	1.646952
C	-2.073710	-5.968165	0.456800
C	-2.556548	-5.452608	-0.741531
C	-3.277678	-4.255931	-0.777615
C	-3.347326	-3.406000	2.990248

C	-3.648939	-4.366218	4.149106
C	-4.840305	-5.288601	3.942198
C	-3.836711	-3.715870	-2.084862
C	-4.387127	-4.818324	-2.999949
C	-5.168085	-4.293096	-4.195911
C	-2.842445	-2.779066	-2.800768
C	-1.652482	-3.444361	-3.471434
C	-2.195397	-2.452618	3.347099
C	-2.444992	-1.527222	4.528329
C	-3.687514	2.022928	2.984497
C	-2.856794	3.215376	3.427650
Ca	-2.903913	-0.405558	0.124574
Ca	2.678413	-0.634773	0.036582
P	-0.019085	0.291922	1.457572
P	-0.175598	-1.446820	0.112894
P	-0.024990	-0.121514	-1.626882
P	0.198380	1.546751	-0.285296
H	-7.502723	-1.628196	0.520640
H	7.221046	-2.190242	0.329392
H	6.657442	-2.312713	3.071439
H	6.326393	-0.604077	3.417797
H	7.649388	-1.063812	2.302333
H	1.998769	-4.718055	-3.869771
H	1.545574	-3.013091	-5.603146
H	-7.565338	1.371332	-0.811982
H	-7.488713	1.660372	0.917442
H	-8.391014	0.249533	0.302100
H	7.165641	-1.872283	-2.406418
H	5.921141	-3.037389	-2.901368
H	7.094424	-3.454347	-1.615987
H	5.050876	0.122423	-2.740976
H	6.062877	1.110725	1.634069
H	-5.799103	-4.337359	1.489853
H	-5.995467	-4.406698	-0.254385
H	-7.292477	-3.699137	0.744837
H	2.048352	-0.427325	5.513282
H	3.891004	3.089848	3.904129
H	3.839322	-2.693768	3.108435
H	2.365614	2.027672	5.534756
H	-5.125251	4.343969	-2.607403
H	4.479497	-4.218140	-1.143645
H	2.622833	-0.788119	-5.455803
H	2.826531	1.122768	-2.300359
H	2.550809	1.350683	-4.002209
H	-4.696385	-3.080468	-1.819552
H	-1.508926	-6.903386	0.462704
H	-5.040669	4.967916	1.628895
H	-4.245418	-2.782525	2.839158
H	-5.629243	3.044195	-4.173149
H	-5.952416	1.376640	-4.599457
H	-1.958592	-5.720260	2.585503
H	2.762858	-6.236582	-2.661040
H	3.847209	-6.583000	-1.330314
H	4.265579	1.417623	0.070168
H	5.537765	2.587713	-0.140008
H	-3.165200	0.862247	-2.246323
H	-3.918092	0.219503	-3.699275
H	-5.450449	4.188757	3.521707
H	-5.769741	2.724090	4.425887
H	-2.748505	-4.963039	4.378846
H	-3.827039	-3.759942	5.051682
H	-5.048928	5.872505	-0.668676
H	-2.377841	-6.002635	-1.667593
H	-5.668091	0.674452	-2.241816
H	5.612756	1.358192	-4.778552
H	4.353923	0.546815	-5.690030
H	-5.039207	-5.472842	-2.396633
H	-3.571210	-5.467614	-3.358363
H	-2.451193	-2.052583	-2.064675
H	-3.392508	-2.176851	-3.543523
H	5.479621	3.867794	2.806324
H	6.777002	3.462754	1.701961
H	2.914231	-5.139513	0.364012
H	2.483272	-3.475792	0.014023
H	2.680035	-2.492562	5.934693
H	3.345935	-3.925450	5.174049
H	-3.800620	1.327937	3.834079
H	-3.113576	1.461863	2.222627
H	-1.950033	-1.845202	2.457823
H	-1.284595	-3.049840	3.521863
H	1.509822	-2.263679	2.375648
H	0.963437	-2.253633	4.034726
H	-5.602609	1.362584	2.365223
H	4.944811	-7.066840	-3.518901
H	4.726498	-5.379497	-4.030692
H	5.809456	-5.764163	-2.676564
H	0.432712	-4.813354	0.101636
H	0.669603	-4.281375	-1.564113
H	1.048411	-5.959978	-1.104919
H	0.548740	-4.444010	2.786788
H	2.301656	-4.694631	2.695963
H	1.485211	-4.688447	4.273915
H	4.987140	-2.919636	6.745064
H	5.620762	-2.878735	5.088655
H	4.976877	-1.397169	5.827421
H	-1.854532	2.888021	3.741374
H	-2.728464	3.952567	2.620794
H	-3.313332	3.732413	4.285037
H	-7.885112	3.931002	3.880255
H	-7.551723	3.782883	2.143087
H	-7.873976	2.329401	3.117900
H	-2.012946	1.764059	-4.171885
H	-3.513601	2.366710	-4.897300
H	-2.886302	3.111013	-3.409764
H	-8.072462	2.664056	-4.321842
H	-7.984945	1.355997	-3.126475

H	-7.656311	3.034848	-2.636261
H	7.605515	3.483513	4.052703
H	6.430332	2.231272	4.508421
H	7.741477	1.847807	3.373714
H	3.259402	3.630821	-0.386936
H	2.867461	3.202269	1.283914
H	4.152614	4.375887	0.946987
H	6.577529	-0.439356	-6.193022
H	6.850285	-0.799759	-4.477578
H	5.581886	-1.662557	-5.372968
H	3.304265	3.451012	-2.800617
H	4.877372	2.683631	-2.471922
H	4.380922	3.043008	-4.139432
H	-1.638028	-0.783275	4.613586
H	-3.393265	-0.976844	4.412514
H	-2.494871	-2.070654	5.483901
H	-4.994179	-5.937871	4.817828
H	-5.768723	-4.713763	3.795165
H	-4.707171	-5.940908	3.065323
H	-0.945151	-2.689229	-3.846252
H	-1.101296	-4.085829	-2.768393
H	-1.959369	-4.067058	-4.326547
H	-5.612837	-5.118646	-4.772281
H	-5.989537	-3.630335	-3.877807
H	-4.531454	-3.719802	-4.887681
H	2.432425	8.496866	-1.805718
H	2.755067	8.538638	-0.054563
H	3.803825	7.613800	-1.142829
H	-2.070045	5.230961	-0.663121
H	-2.070445	3.626699	-1.397654
H	-2.067491	3.809749	0.381703
H	2.682654	6.933955	-3.439366
H	2.123719	5.601839	-5.442879
H	0.602416	3.633934	-5.240649
H	-0.368603	3.044227	-3.042410
H	-0.316354	3.500740	2.079200
H	0.723957	4.437945	4.116959
H	2.231181	6.421775	3.947307
H	2.711464	7.413149	1.736111

228

[(BDI*)Ca]2(P4) + DBA Adduct

C	-2.207583	-4.528823	-0.370274
C	-2.635539	-3.804652	0.772367
C	-2.072916	-4.065147	2.045131
C	-1.012632	-4.972990	2.129166
C	-0.546672	-5.646688	1.006565
C	-1.157211	-5.438706	-0.226083
N	-3.645907	-2.814402	0.623538
C	-4.908910	-3.232573	0.700140
C	-5.194005	-4.681780	1.019167
C	-2.610281	-3.393597	3.301891
C	-1.821847	-2.110789	3.601079
C	-2.396021	-1.211581	4.684542
C	-2.919998	-4.362064	-1.705608
C	-2.323055	-3.238905	-2.572436
C	-0.919504	-3.467886	-3.100951
C	-6.045072	-2.438730	0.476049
C	-6.170735	-1.093313	0.086296
C	-7.587661	-0.640134	-0.170495
N	-5.166344	-0.234514	-0.086155
C	-5.516642	1.092508	-0.471462
C	-5.537732	1.439820	-1.846531
C	-5.760439	2.776713	-2.193161
C	-5.991766	3.745346	-1.223451
C	-6.047144	3.377984	0.116817
C	-5.827331	2.057594	0.522406
C	-5.360621	0.377320	-2.924463
C	-6.297360	0.554412	-4.129508
C	-7.782247	0.445807	-3.822267
C	-5.952217	1.669689	1.991994
C	-4.632728	1.815397	2.776209
C	-4.145723	3.235695	3.009984
C	-3.902544	0.222502	-3.399998
C	-3.339304	1.352580	-4.244961
C	-7.089454	2.392550	2.731415
C	-8.493359	2.118970	2.215405
C	-2.647751	-4.316940	4.528030
C	-3.495937	-5.570927	4.382548
C	-3.061139	-5.688863	-2.466635
C	-3.991682	-5.618760	-3.668935
Ca	-2.806416	-0.658886	0.092864
P	0.193092	-1.566898	0.026229
P	0.114534	-0.193595	-1.654870
P	0.255547	1.405184	-0.210575
P	0.118005	0.061858	1.471314
B	-0.934499	3.123918	-0.328300
C	-2.423096	2.459235	-0.341964
C	-0.650730	3.987290	0.999918
C	-0.673400	3.397007	2.275021
C	-0.432361	4.126309	3.437403
C	-0.194672	5.498541	3.359978
C	-0.205011	6.112282	2.112417
C	-0.414847	5.388294	0.921284
B	-0.346510	6.113686	-0.461642
C	-0.250484	7.691099	-0.567495
C	-0.322831	5.248559	-1.765742
C	-0.564280	3.848634	-1.715760
C	-0.544707	3.134247	-2.926456
C	-0.280037	3.748552	-4.147750
C	-0.037057	5.122115	-4.196507
C	-0.068839	5.852948	-3.014842
Ca	2.945778	-0.146026	-0.040327
N	4.652521	0.063095	1.567273
C	4.061595	0.552054	2.760886
C	3.484541	-0.339617	3.695518

C	2.683107	0.187835	4.713980
C	2.489236	1.556730	4.844198
C	3.152100	2.430908	3.987004
C	3.961954	1.957688	2.951792
C	3.755249	-1.834456	3.642147
C	2.550643	-2.607155	3.085363
C	2.766857	-4.102260	2.903901
C	4.761454	2.910292	2.073202
C	5.298952	4.136005	2.823765
C	6.276766	3.811237	3.942974
C	5.965280	-0.056198	1.433931
C	6.875511	0.120352	2.627474
C	6.623273	-0.371154	0.227614
C	6.109708	-0.646819	-1.052111
C	7.136668	-0.932003	-2.120263
N	4.817290	-0.669808	-1.375548
C	4.435426	-1.108485	-2.673018
C	4.144096	-0.170625	-3.693426
C	3.600571	-0.636345	-4.896445
C	3.392839	-1.990064	-5.122074
C	3.754272	-2.911783	-4.143869
C	4.279527	-2.501550	-2.915774
C	4.410362	1.317170	-3.521114
C	5.094745	1.948853	-4.744270
C	6.419273	1.326601	-5.156529
C	4.669780	-3.522580	-1.856032
C	3.501246	-3.839330	-0.904736
C	2.367509	-4.660454	-1.491449
C	3.110919	2.062421	-3.174491
C	3.252944	3.553840	-2.915082
C	5.284363	-4.812073	-2.417050
C	6.598885	-4.629904	-3.159907
C	4.196246	-2.393341	5.005140
C	5.414287	-1.722843	5.620394
C	4.022922	3.323994	0.788977
C	2.832222	4.244628	0.968784
H	-6.991571	-2.966745	0.581243
H	7.710428	-0.409833	0.301976
H	7.215229	-0.869594	2.972554
H	6.374901	0.615303	3.468663
H	7.773669	0.691667	2.354258
H	3.630085	-3.977822	-4.346194
H	2.969289	-2.332048	-6.069499
H	-7.704759	-0.321530	-1.215656
H	-7.848629	0.232260	0.441629
H	-8.306350	-1.442630	0.033183
H	7.020144	-0.231474	-2.959085
H	7.001164	-1.937076	-2.543530
H	8.158155	-0.850121	-1.729827
H	5.093957	1.433131	-2.661910
H	5.647080	2.353595	1.730020
H	-4.683075	-4.995412	1.939370
H	-4.814086	-5.339092	0.221813
H	-6.270129	-4.859422	1.131367
H	2.210286	-0.491049	5.428488
H	3.041221	3.507250	4.133750
H	4.590034	-1.988673	2.936682
H	1.847507	1.949188	5.636730
H	-5.769622	3.065826	-3.246234
H	5.441356	-3.055158	-1.225033
H	3.349284	0.081082	-5.681537
H	2.649266	1.585550	-2.289704
H	2.373296	1.892532	-3.977987
H	-3.945598	-4.031468	-1.476903
H	0.278036	-6.358135	1.096359
H	-6.277510	4.138441	0.865803
H	-3.648656	-3.086283	3.088441
H	-6.100655	1.518583	-4.625595
H	-6.025298	-0.213221	-4.875386
H	-0.555632	-5.175797	3.100578
H	4.563049	-5.327593	-3.072181
H	5.440910	-5.499739	-1.567259
H	3.699821	2.407668	0.258531
H	4.759166	3.790999	0.111923
H	-3.241708	0.083176	-2.522117
H	-3.829233	-0.723808	-3.962950
H	-6.910944	3.479884	2.732320
H	-7.031453	2.093307	3.792675
H	-1.617030	-4.593553	4.812007
H	-3.030264	-3.731204	5.379216
H	-6.154694	4.785636	-1.514510
H	-0.813617	-6.007231	-1.092361
H	-5.620878	-0.587229	-2.459521
H	5.260122	3.015527	-4.525842
H	4.396417	1.936118	-5.599841
H	-3.434551	-6.449993	-1.760494
H	-2.074369	-6.054177	-2.796319
H	-2.319445	-2.306487	-1.978773
H	-3.011747	-3.036444	-3.409476
H	4.467945	4.741268	3.221219
H	5.797538	4.784366	2.081998
H	3.907512	-4.353950	-0.017498
H	3.083531	-2.888844	-0.524706
H	3.348242	-2.342386	5.711320
H	4.407217	-3.467869	4.882494
H	-4.765693	1.310298	3.749131
H	-3.843387	1.238917	2.258962
H	-1.735675	-1.527743	2.668348
H	-0.778221	-2.378639	3.843780
H	2.262557	-2.168086	2.114276
H	1.676041	-2.428332	3.735816
H	-6.189118	0.594286	2.019364
H	6.974963	-5.591130	-3.542780
H	6.488083	-3.951241	-4.020077
H	7.377795	-4.212938	-2.501790
H	1.530767	-4.722662	-0.781361

H	1.981410	-4.207659	-2.416826
H	2.685978	-5.688131	-1.725698
H	1.938992	-4.544778	2.330729
H	3.699647	-4.306941	2.353540
H	2.827379	-4.636128	3.864407
H	5.667809	-2.179200	6.589619
H	6.297039	-1.822082	4.969629
H	5.246576	-0.648303	5.790366
H	-3.202461	3.240430	3.573066
H	-3.954560	3.767806	2.066244
H	-4.874191	3.822704	3.589752
H	-9.240682	2.687738	2.789827
H	-8.602256	2.406775	1.158026
H	-8.756806	1.053132	2.304442
H	-2.298845	1.137445	-4.528768
H	-3.909599	1.482322	-5.177123
H	-3.340303	2.312808	-3.708169
H	-8.384899	0.596545	-4.731037
H	-8.039666	-0.547509	-3.421268
H	-8.100181	1.200382	-3.086052
H	6.644023	4.728081	4.429212
H	5.810277	3.186591	4.720520
H	7.154286	3.264982	3.561398
H	2.325892	4.422277	0.009490
H	2.083854	3.820771	1.653944
H	3.132916	5.225817	1.366045
H	6.824462	1.822376	-6.052119
H	7.174596	1.429178	-4.360927
H	6.316368	0.255040	-5.387263
H	2.309438	3.971066	-2.534264
H	4.041975	3.760970	-2.174566
H	3.501185	4.110233	-3.830877
H	-1.827130	-0.270956	4.744361
H	-3.447754	-0.953976	4.476639
H	-2.360569	-1.677505	5.680823
H	-3.462872	-6.178203	5.300167
H	-4.552428	-5.319160	4.197307
H	-3.151428	-6.206031	3.551881
H	-0.566647	-2.594648	-3.670326
H	-0.208381	-3.634452	-2.279030
H	-0.870733	-4.340523	-3.771501
H	-4.131714	-6.613970	-4.117669
H	-4.987158	-5.240303	-3.384326
H	-3.601683	-4.959237	-4.459578
H	-0.885054	8.086260	-1.377156
H	-0.504278	8.234604	0.353580
H	0.785210	7.973528	-0.834286
H	-3.205342	3.230008	-0.438146
H	-2.566238	1.777953	-1.200602
H	-2.636014	1.938254	0.611953
H	0.111363	6.930724	-3.059758
H	0.168825	5.615497	-5.150220
H	-0.266955	3.154547	-5.066551
H	-0.739277	2.057079	-2.920998
H	-0.873360	2.325151	2.375173
H	-0.434327	3.620471	4.407535
H	-0.011478	6.082960	4.265750
H	-0.030793	7.190273	2.057738

198
[(BDI*)Mg]2(P4) (2)

Mg	-2.761974	-0.066456	0.279638
Mg	2.606486	0.103747	-0.456402
P	0.367205	0.416011	-1.800172
P	-0.413452	1.104994	0.162273
P	-0.413440	-1.066187	-0.340815
P	0.787685	-0.319250	1.373408
N	4.004320	1.623169	-0.293993
N	-4.372527	0.218338	-0.902640
N	-3.763639	-0.470223	1.997277
N	4.003386	-1.337849	-0.965584
C	5.862227	0.223461	-0.988627
H	6.932226	0.269317	-1.192386
C	-1.303957	-1.260381	5.333995
H	-0.658627	-1.463325	6.192064
C	5.292504	1.443153	-0.568226
C	-2.969333	-0.743833	3.149544
C	-4.197512	0.544594	-2.276738
C	5.291436	-1.057570	-1.137219
C	-5.921630	-0.208169	0.921433
H	-6.988701	-0.256435	1.139963
C	-2.526132	-2.067051	3.392258
C	-5.610751	0.109342	-0.413688
C	-5.090004	-0.476969	2.032474
C	-1.704323	0.039926	5.053887
H	-1.351158	0.853791	5.689684
C	-1.700834	-2.296814	4.498672
H	-1.344508	-3.308582	4.700489
C	3.534061	-2.674946	-1.076349
C	3.534899	2.878460	0.179825
C	6.264352	-2.162466	-1.478216
H	5.798472	-2.953758	-2.080122
H	6.631843	-2.637785	-0.554085
H	7.135749	-1.763894	-2.013670
C	6.266578	2.585322	-0.394999
H	5.803247	3.559734	-0.598354
H	7.140820	2.455516	-1.045924
H	6.629000	2.614402	0.645565
C	-2.529364	0.330083	3.961744
C	-5.828747	-0.786033	3.313309
H	-6.474670	-1.664853	3.167881
H	-6.490440	0.053047	3.575102
H	-5.152411	-0.979760	4.154204
C	-4.083283	-0.493573	-3.231788
C	-6.786929	0.336419	-1.333142
H	-6.751045	1.341567	-1.776260

H	-7.737283	0.218827	-0.799076
H	-6.766250	-0.365369	-2.178748
C	-1.666152	-4.062156	2.096625
H	-0.872053	-3.375680	1.758325
H	-1.258852	-4.559774	2.992286
C	-4.065934	1.900209	-2.661463
C	-1.678023	2.699771	3.713861
H	-0.875553	2.242793	3.111143
H	-1.281515	2.743880	4.741791
C	2.496171	5.307633	1.110562
H	2.096099	6.258113	1.472193
C	3.694957	3.234734	1.543265
C	3.700544	-3.583482	0.000002
C	-2.893543	-3.212751	2.463503
H	-3.245892	-2.755079	1.522890
C	1.363625	-2.299796	-4.180403
H	1.333060	-3.287878	-4.670049
H	0.548044	-2.310156	-3.437655
C	-2.900158	1.770119	3.646876
H	-3.243030	1.784644	2.597813
C	2.495245	-5.268535	-1.276306
H	2.095292	-6.282344	-1.356935
C	2.846990	3.737990	-0.715235
C	3.176014	4.460152	1.976974
H	3.309482	4.759329	3.018445
C	1.377521	3.872483	-2.791579
H	0.561355	3.554765	-2.120899
H	1.340244	4.974942	-2.804603
C	2.839647	-3.065565	-2.250353
C	3.918103	-2.142996	-4.358493
H	4.805958	-1.864937	-3.770485
H	3.804746	-1.337807	-5.103473
C	3.933289	3.817297	-3.011593
H	3.825364	3.409760	-4.030633
H	4.821733	3.317315	-2.596747
C	-4.008955	-0.142298	-4.584275
H	-3.938555	-0.927177	-5.339818
C	4.186079	5.315485	-3.089819
H	4.332891	5.756864	-2.091479
H	3.351007	5.851634	-3.568203
H	5.088939	5.528318	-3.682895
C	3.181443	-4.876384	-0.133268
H	3.319563	-5.594716	0.677214
C	-5.293588	-2.711011	-2.732536
H	-5.897163	-2.284099	-1.916197
H	-5.079219	-3.745270	-2.414749
C	-3.998854	1.188425	-4.985172
H	-3.951487	1.441090	-6.047176
C	4.176591	-3.461024	-5.073019
H	4.327732	-4.287744	-4.361154
H	5.078652	-3.394184	-5.700712
H	3.342329	-3.743646	-5.734658
C	-3.991666	2.195427	-4.027321

H	-3.907756	3.235562	-4.348074
C	4.400927	2.319547	2.530789
H	4.973990	1.587154	1.941893
C	-5.249424	3.672276	-1.216057
H	-5.023530	4.448260	-0.465434
H	-5.864010	2.931691	-0.680458
C	2.332572	4.941726	-0.220253
H	1.798827	5.614230	-0.893962
C	4.412456	-3.182114	1.281915
H	4.989579	-2.271196	1.061360
C	2.532882	-3.848469	2.973188
H	3.113054	-4.656566	3.447547
H	1.886546	-4.302858	2.206898
H	1.871415	-3.422477	3.743188
C	2.325220	-4.365347	-2.318945
H	1.786449	-4.682673	-3.213375
C	-3.920116	3.007401	-1.627371
H	-3.539434	2.524509	-0.709546
C	-3.954592	-1.949722	-2.808025
H	-3.567842	-1.936733	-1.773239
C	2.688304	-2.113402	-3.426928
H	2.666102	-1.092913	-3.006380
C	2.702227	3.385157	-2.187655
H	2.684022	2.283053	-2.247500
C	-3.882493	2.431554	5.967760
H	-3.068063	3.136845	6.197419
H	-3.636927	1.470907	6.445629
H	-4.791550	2.817544	6.454380
C	-6.116902	-2.738651	-4.011768
H	-5.568143	-3.201196	-4.847394
H	-6.408665	-1.726349	-4.332800
H	-7.039633	-3.321826	-3.868894
C	5.421127	3.074393	3.397708
H	4.907115	3.782038	4.069460
H	6.040255	3.702125	2.734233
C	2.523841	2.209087	4.348188
H	3.109504	2.734108	5.120150
H	1.861097	1.498157	4.865206
H	1.879117	2.950436	3.852073
C	-2.850250	4.029829	-2.049728
H	-2.008347	3.477630	-2.498993
H	-3.240833	4.682569	-2.848960
C	-2.899847	-2.687311	-3.651718
H	-3.301105	-2.908802	-4.655369
H	-2.050910	-2.002765	-3.813284
C	-3.852813	-4.862395	4.238691
H	-3.033468	-5.588745	4.118070
H	-4.756039	-5.434322	4.501875
H	-3.607513	-4.222011	5.099592
C	-6.064071	4.286490	-2.344749
H	-5.503858	5.068100	-2.881969
H	-6.979708	4.756141	-1.953618
H	-6.367575	3.532335	-3.087751

C	-4.102321	2.279228	4.469952
H	-4.417150	3.249030	4.048956
H	-4.950751	1.598995	4.299503
C	-1.924245	-5.108089	1.022529
H	-0.983981	-5.588074	0.710597
H	-2.375893	-4.658777	0.123307
H	-2.600557	-5.905609	1.367786
C	5.428223	-4.240108	1.740811
H	6.041893	-4.529629	0.870643
H	4.909988	-5.163123	2.050213
C	-1.938600	4.115767	3.222528
H	-1.001050	4.690315	3.170956
H	-2.625562	4.668429	3.882259
H	-2.378725	4.116872	2.212225
C	1.098557	3.356216	-4.195490
H	1.133327	2.255464	-4.230861
H	1.819252	3.740809	-4.933618
H	0.094540	3.658509	-4.530425
C	1.075721	-1.228058	-5.221584
H	1.099871	-0.220733	-4.775625
H	0.073620	-1.364843	-5.656003
H	1.797576	-1.247179	-6.052676
C	-4.086384	-4.047941	2.974930
H	-4.937981	-3.369739	3.138049
H	-4.402690	-4.723039	2.161852
C	-2.323587	4.890819	-0.911561
H	-1.862094	4.271165	-0.126027
H	-3.112711	5.496568	-0.439283
H	-1.550954	5.587323	-1.271478
C	-2.383491	-3.972678	-3.022972
H	-1.912002	-3.775144	-2.046913
H	-1.621611	-4.443970	-3.662545
H	-3.180296	-4.716212	-2.864983
C	3.407273	1.472639	3.353363
H	3.978638	0.691471	3.883410
H	2.762644	0.926538	2.645208
C	6.324273	2.170537	4.224482
H	5.761490	1.600414	4.979623
H	7.087106	2.755994	4.760356
H	6.852206	1.440786	3.588546
C	6.338968	-3.779196	2.869644
H	5.781242	-3.581285	3.797912
H	6.871149	-2.851321	2.602275
H	7.098344	-4.541459	3.102297
C	3.423310	-2.762778	2.389537
H	2.783875	-1.961602	1.984095
H	3.998676	-2.287921	3.202655

198

[(BDI*)Ca]2(P4) (5)

C	3.489760	-3.481219	-1.678788
C	3.815431	-2.946515	-0.406774
C	3.537021	-3.686622	0.771141
C	2.884154	-4.917513	0.651725
C	2.509079	-5.420723	-0.588500
C	2.821002	-4.707754	-1.739269
N	4.357722	-1.635931	-0.307543
C	5.676308	-1.469911	-0.301283
C	6.568242	-2.685311	-0.375400
C	3.952844	-3.168397	2.138136
C	2.815251	-2.418659	2.857644
C	1.583256	-3.226718	3.230563
C	3.824410	-2.743477	-2.966557
C	2.589648	-1.975897	-3.465913
C	2.825043	-1.043369	-4.644429
C	6.338196	-0.227867	-0.217862
C	5.836075	1.079734	-0.061599
C	6.870125	2.177294	-0.000690
N	4.549622	1.398451	0.044608
C	4.177407	2.750702	0.272871
C	4.056655	3.226973	1.605190
C	3.568156	4.520835	1.811138
C	3.216285	5.340492	0.745070
C	3.376197	4.880316	-0.556076
C	3.862852	3.596142	-0.821703
C	4.441648	2.349284	2.787752
C	3.250300	1.527070	3.315552
C	2.114425	2.304734	3.958047
C	4.047119	3.124918	-2.256859
C	2.776772	2.467134	-2.828315
C	1.540689	3.343686	-2.943601
C	5.133653	3.104477	3.931058
C	6.461199	3.748682	3.563766
C	4.574112	4.209684	-3.207079
C	5.946344	4.761514	-2.853639
C	4.593842	-4.263952	3.004912
C	5.265182	-3.746759	4.269034
C	4.388659	-3.655686	-4.066071
C	5.654227	-4.416314	-3.703387
Ca	2.679479	-0.017435	-0.042899
P	0.039063	1.621297	0.062888
P	-0.002221	0.077930	-1.476553
Ca	-2.644434	0.063519	0.043081
N	-4.453356	1.548573	-0.179856
C	-4.047009	2.906095	-0.308051
C	-3.787877	3.455233	-1.589321
C	-3.255806	4.746129	-1.672385
C	-3.008762	5.503742	-0.535318
C	-3.311060	4.977401	0.715098
C	-3.833078	3.688390	0.857603
C	-4.035270	2.672117	-2.869984
C	-2.712958	2.062836	-3.365164
C	-2.815730	1.159037	-4.584356
C	-4.146156	3.139417	2.241598
C	-4.735906	4.175737	3.209071
C	-6.064539	4.775831	2.776584

C	-5.750299	1.254882	-0.149042
C	-6.755908	2.373309	-0.269929
C	-6.293366	-0.038560	-0.008774
C	-5.683253	-1.301078	0.141275
C	-6.627920	-2.473180	0.242855
N	-4.373512	-1.522263	0.197853
C	-3.890886	-2.854128	0.331689
C	-3.609924	-3.620970	-0.829963
C	-3.002728	-4.870865	-0.677910
C	-2.681784	-5.374410	0.577243
C	-3.000550	-4.637415	1.710022
C	-3.619548	-3.386566	1.617309
C	-3.945184	-3.098927	-2.219481
C	-2.778087	-2.313650	-2.846481
C	-1.486550	-3.076770	-3.088277
C	-3.950133	-2.625920	2.892923
C	-4.588653	-3.503664	3.980539
C	-5.874274	-4.213152	3.586460
C	-2.687815	-1.925524	3.421952
C	-2.887588	-1.026822	4.632860
C	-4.457359	-4.176774	-3.185945
C	-5.747932	-4.860113	-2.761812
C	-4.707405	3.500606	-3.975685
C	-6.048008	4.120266	-3.614889
C	-2.932453	2.432914	2.874573
C	-1.696006	3.281347	3.117826
H	7.424949	-0.291570	-0.262705
H	-7.382343	-0.068237	-0.019038
H	-6.605322	2.931825	-1.204154
H	-6.625462	3.101904	0.543392
H	-7.785314	1.996511	-0.243917
H	-2.775907	-5.467566	-1.563836
H	-2.194911	-6.347951	0.671642
H	6.811143	2.716491	0.955825
H	6.685947	2.927087	-0.783740
H	7.886116	1.782557	-0.120804
H	-6.463623	-3.023818	1.179481
H	-6.442718	-3.192361	-0.568111
H	-7.675894	-2.153622	0.198133
H	-4.677122	-1.837132	2.633346
H	-4.911849	2.356825	2.114700
H	6.322230	-3.303234	-1.249917
H	6.412445	-3.327573	0.505703
H	7.627984	-2.407137	-0.421062
H	-3.035984	5.170990	-2.654937
H	-3.138170	5.587014	1.604378
H	-4.710622	1.834226	-2.625294
H	-2.590879	6.509463	-0.622759
H	3.468271	4.901147	2.830023
H	-4.761939	-2.368089	-2.102619
H	-2.769018	-5.047286	2.696271
H	-2.240128	-1.330457	2.606509
H	-1.923741	-2.691449	3.644333
H	4.736409	-2.412538	1.967874
H	1.985577	-6.377205	-0.658213
H	3.124220	5.540687	-1.388453
H	4.598409	-1.994000	-2.728528
H	4.459448	3.869905	4.349465
H	5.294745	2.384414	4.752465
H	2.546652	-5.119755	-2.713608
H	-3.679146	-4.938373	-3.359359
H	-4.608738	-3.693641	-4.166978
H	-2.636488	1.577024	2.239673
H	-3.261857	1.977590	3.823893
H	2.822777	0.932896	2.487404
H	3.639703	0.782083	4.031280
H	3.852275	5.040006	-3.279733
H	4.609469	3.771374	-4.219680
H	3.607720	-4.366667	-4.389183
H	4.593226	-3.032820	-4.951724
H	2.830073	6.345848	0.928911
H	2.665270	-5.498198	1.549962
H	5.166911	1.608259	2.414995
H	-4.792946	-2.865040	4.854878
H	-3.849243	-4.244620	4.332745
H	5.338646	-4.794715	2.387714
H	3.846079	-5.026547	3.279410
H	2.482718	-1.580386	2.219454
H	3.229526	-1.936282	3.758929
H	-4.010645	4.985328	3.393951
H	-4.864317	3.677789	4.185931
H	-3.133872	-1.879759	-3.796458
H	-2.543006	-1.442846	-2.207541
H	-4.013183	4.290006	-4.314471
H	-4.846223	2.846442	-4.851325
H	3.027613	2.049002	-3.818275
H	2.512869	1.589221	-2.210856
H	2.168398	-1.390815	-2.629619
H	1.796589	-2.703713	-3.712903
H	-2.249990	1.493876	-2.539839
H	-1.998626	2.881593	-3.563756
H	4.807606	2.327055	-2.236109
H	-6.053811	-5.622212	-3.495031
H	-5.640486	-5.360953	-1.787124
H	-6.575015	-4.136881	-2.677881
H	-0.730897	-2.410656	-3.532198
H	-1.065973	-3.472616	-2.151031
H	-1.630065	-3.920085	-3.782126
H	-1.864194	0.632309	-4.754252
H	-3.598753	0.394665	-4.452493
H	-3.050440	1.718083	-5.502644
H	-6.449997	4.706580	-4.455534
H	-6.794197	3.346843	-3.372409
H	-5.967439	4.794484	-2.748204
H	0.699116	2.761606	-3.350253
H	1.225316	3.733480	-1.963723

H	1.704038	4.199525	-3.617342
H	6.261428	5.534010	-3.571899
H	5.954867	5.216733	-1.851099
H	6.712187	3.969333	-2.868582
H	1.309878	1.618366	4.262075
H	2.443966	2.848867	4.856750
H	1.677826	3.033953	3.258665
H	6.899127	4.275765	4.425341
H	7.194458	2.995459	3.233628
H	6.344268	4.482083	2.750695
H	-6.427459	5.509518	3.512729
H	-5.980716	5.291422	1.807290
H	-6.840952	4.000205	2.677832
H	-0.901399	2.673861	3.575484
H	-1.295327	3.692511	2.178659
H	-1.901701	4.121256	3.799821
H	-6.252384	-4.832162	4.414654
H	-6.667562	-3.492275	3.331879
H	-5.727658	-4.873647	2.717892
H	-1.978788	-0.437712	4.829677
H	-3.715961	-0.317182	4.474032
H	-3.112573	-1.597827	5.546187
H	1.930179	-0.432107	-4.837633
H	3.662268	-0.353550	-4.447942
H	3.056309	-1.588194	-5.572169
H	5.986331	-5.051577	-4.538980
H	6.481637	-3.727465	-3.469474
H	5.502364	-5.069236	-2.829915
H	0.851136	-2.586172	3.746122
H	1.086050	-3.642163	2.340717
H	1.825300	-4.060809	3.908640
H	5.783619	-4.559023	4.801123
H	6.013958	-2.971626	4.036985
H	4.543057	-3.306903	4.974098
P	0.038860	0.067237	1.581865
P	-0.003356	-1.483082	0.051108

196
[(BDI*)Ca]2(N2)_singlet

C	-2.801241	3.870142	0.380551
C	-3.374233	2.803116	1.116082
C	-3.361464	2.831039	2.537914
C	-2.775774	3.925039	3.182293
C	-2.189213	4.963143	2.464453
C	-2.202578	4.926581	1.075762
N	-3.891794	1.673152	0.437090
C	-5.203355	1.549739	0.268855
C	-6.106466	2.688001	0.684813
C	-3.965598	1.694062	3.345351
C	-2.892033	0.706638	3.856248
C	-2.043081	1.174844	5.028666
C	-2.806432	3.874120	-1.139298
C	-1.439850	3.407249	-1.672690
C	-1.363920	3.193834	-3.176955
C	-5.861871	0.417473	-0.256840
C	-5.386583	-0.836507	-0.700379
C	-6.455189	-1.832098	-1.087327
N	-4.110875	-1.195184	-0.789300
C	-3.783579	-2.473131	-1.306688
C	-3.528275	-3.550923	-0.421085
C	-3.154051	-4.788255	-0.957535
C	-3.025603	-4.974442	-2.328420
C	-3.269890	-3.910552	-3.190600
C	-3.646736	-2.653129	-2.709677
C	-3.631126	-3.362464	1.084076
C	-2.268695	-2.990578	1.708339
C	-1.168634	-4.036405	1.613809
C	-3.864177	-1.490525	-3.666861
C	-2.556490	-0.704202	-3.898346
C	-1.468294	-1.428642	-4.674635
C	-4.271796	-4.544583	1.823459
C	-5.708595	-4.839483	1.422684
C	-4.518240	-1.874611	-5.000369
C	-5.925055	-2.440689	-4.886903
C	-4.905511	2.194469	4.452428
C	-5.699621	1.090672	5.135653
C	-3.211491	5.226539	-1.743140
C	-4.582504	5.733156	-1.324396
Ca	-2.214382	0.111732	-0.193606
N	-0.022234	-0.037882	0.508141
N	0.024808	-0.047611	-0.742376
Ca	2.219185	-0.105429	-0.017706
N	3.968046	-1.546686	0.713466
C	3.520493	-2.800994	1.198528
C	3.224473	-3.850130	0.293647
C	2.704785	-5.049989	0.792949
C	2.492459	-5.236664	2.152012
C	2.787677	-4.204475	3.037469
C	3.291853	-2.979838	2.591287
C	3.414866	-3.689788	-1.205841
C	2.057055	-3.380225	-1.865030
C	2.092959	-3.139408	-3.366909
C	3.536571	-1.843890	3.573297
C	4.052361	-2.286422	4.948583
C	5.408301	-2.974973	4.936754
C	5.264645	-1.258719	0.741196
C	6.239959	-2.323816	1.185582
C	5.845478	-0.020635	0.386972
C	5.302423	1.184351	-0.110791
C	6.296607	2.300465	-0.329767
N	4.023233	1.400635	-0.394101
C	3.629149	2.618510	-1.002140
C	3.702651	2.755945	-2.416201
C	3.211050	3.924357	-3.004584
C	2.649736	4.942968	-2.240307
C	2.588448	4.805039	-0.859889

C	3.080328	3.662923	-0.217492
C	4.265111	1.635411	-3.278222
C	3.166504	0.633832	-3.689063
C	2.118269	1.145147	-4.663926
C	2.975236	3.554050	1.294932
C	3.413370	4.828519	2.030972
C	4.836179	5.284511	1.749505
C	1.542467	3.146836	1.684334
C	1.308633	2.891209	3.165039
C	5.061376	2.110233	-4.500693
C	6.316138	2.907179	-4.179661
C	4.097173	-4.895762	-1.867936
C	5.462975	-5.254813	-1.304845
C	2.285830	-0.951308	3.712644
C	1.070355	-1.589891	4.365534
H	-6.946244	0.517150	-0.297098
H	6.926710	0.008701	0.518752
H	6.133131	-3.222576	0.560871
H	6.033467	-2.643760	2.216840
H	7.277399	-1.973502	1.127503
H	3.269800	4.044663	-4.088576
H	2.267644	5.845978	-2.722641
H	-6.371485	-2.738383	-0.468899
H	-6.332003	-2.161843	-2.128564
H	-7.461765	-1.414491	-0.965653
H	5.998476	3.192609	0.239915
H	6.319858	2.607471	-1.385141
H	7.309089	2.008391	-0.026406
H	3.650055	2.741008	1.614638
H	4.313441	-1.195946	3.137263
H	-5.750235	3.640474	0.267160
H	-6.100199	2.810482	1.778929
H	-7.141343	2.520243	0.362883
H	2.470373	-5.860893	0.098525
H	2.619615	-4.357147	4.105766
H	4.069284	-2.815058	-1.364399
H	2.095369	-6.184247	2.523830
H	-2.968580	-5.629327	-0.285909
H	4.963400	1.060848	-2.649554
H	2.156719	5.610351	-0.259976
H	1.257979	2.237989	1.123941
H	0.839472	3.919467	1.326656
H	-4.587623	1.107562	2.652704
H	-1.730551	5.804365	2.989868
H	-3.172545	-4.065937	-4.267366
H	-3.557993	3.133467	-1.463301
H	-3.662217	-5.454799	1.697375
H	-4.233686	-4.319938	2.903779
H	-1.752340	5.749114	0.513852
H	4.417738	2.698982	-5.175039
H	5.340096	1.213614	-5.081856
H	1.985398	-0.589387	2.713345
H	2.574326	-0.050160	4.281361

H	-1.887148	-2.060685	1.247611
H	-2.440546	-2.731422	2.767964
H	-3.881726	-2.587191	-5.550657
H	-4.542201	-0.967447	-5.629476
H	-2.443927	5.983404	-1.502078
H	-3.188731	5.132112	-2.840708
H	-2.738068	-5.950402	-2.726886
H	-2.782315	3.971103	4.273408
H	-4.287007	-2.492443	1.251492
H	3.306649	4.650602	3.113003
H	2.707489	5.646911	1.802614
H	-5.603938	2.923712	4.007629
H	-4.341558	2.761710	5.211761
H	-2.225759	0.448865	3.013209
H	-3.394533	-0.236266	4.132961
H	3.315097	-2.938953	5.445086
H	4.109419	-1.385186	5.583867
H	3.662544	-0.253759	-4.118908
H	2.650410	0.277158	-2.779769
H	3.429264	-5.774040	-1.814433
H	4.204285	-4.680119	-2.943033
H	-2.811448	0.236155	-4.417829
H	-2.134944	-0.402870	-2.922392
H	-1.151885	2.466816	-1.167838
H	-0.664681	4.127950	-1.357731
H	1.608019	-2.496577	-1.373517
H	1.356091	-4.202414	-1.636369
H	-4.548908	-0.785509	-3.169382
H	6.835140	3.219078	-5.099070
H	6.081911	3.817544	-3.605976
H	7.028647	2.311629	-3.586933
H	1.357193	0.373293	-4.851875
H	1.598476	2.030947	-4.269004
H	2.556125	1.416128	-5.636754
H	1.117133	-2.780621	-3.725116
H	2.843898	-2.379550	-3.635496
H	2.331370	-4.054808	-3.928532
H	5.893199	-6.120063	-1.832265
H	6.171802	-4.418502	-1.415676
H	5.409258	-5.509815	-0.235203
H	-0.562214	-0.807942	-4.736614
H	-1.187925	-2.374392	-4.186459
H	-1.778610	-1.659462	-5.705289
H	-6.334080	-2.690475	-5.878009
H	-5.944532	-3.359279	-4.279727
H	-6.612316	-1.716508	-4.421149
H	-0.240137	-3.670842	2.076278
H	-1.442839	-4.970442	2.128608
H	-0.932134	-4.285684	0.568492
H	-6.116850	-5.686164	1.995719
H	-6.361375	-3.971228	1.608046
H	-5.785644	-5.093834	0.354018
H	5.717304	-3.262246	5.953626

H	5.393626	-3.890047	4.324195
H	6.190170	-2.313160	4.531206
H	0.221235	-0.891050	4.355595
H	0.752761	-2.496941	3.829716
H	1.259242	-1.866269	5.414489
H	5.076489	6.200846	2.310424
H	5.567481	4.516949	2.049889
H	4.993179	5.497634	0.680792
H	0.304735	2.472095	3.325557
H	2.039534	2.173324	3.571024
H	1.377280	3.811945	3.763567
H	-0.398005	2.748212	-3.455570
H	-2.157132	2.513512	-3.528042
H	-1.462981	4.135502	-3.737140
H	-4.811214	6.698707	-1.801241
H	-5.375074	5.026402	-1.618499
H	-4.648365	5.876149	-0.234725
H	-1.312537	0.402022	5.311181
H	-1.475943	2.086583	4.789394
H	-2.652258	1.383969	5.921973
H	-6.416074	1.507243	5.860176
H	-6.273339	0.500019	4.402810
H	-5.050097	0.391501	5.685033

196
[(BDI*)Ca]2(N2)_triplet

C	2.773960	3.855986	-0.406610
C	3.364206	2.793342	-1.134097
C	3.375566	2.822440	-2.555747
C	2.806569	3.920140	-3.208514
C	2.211540	4.959198	-2.499215
C	2.194010	4.916938	-1.111015
N	3.883304	1.667286	-0.446521
C	5.195966	1.558956	-0.267341
C	6.091763	2.700788	-0.687498
C	3.983911	1.682449	-3.355664
C	2.909615	0.701583	-3.878356
C	2.069579	1.179797	-5.053297
C	2.734343	3.853155	1.112756
C	1.341428	3.413000	1.600005
C	1.206239	3.209997	3.101487
C	5.859317	0.439126	0.276352
C	5.385835	-0.808473	0.737829
C	6.453800	-1.793959	1.149495
N	4.109331	-1.167257	0.824131
C	3.784554	-2.438846	1.363575
C	3.536952	-3.533271	0.497123
C	3.177411	-4.764594	1.057022
C	3.052129	-4.926610	2.431185
C	3.284533	-3.844595	3.273718
C	3.649703	-2.593122	2.769166
C	3.628722	-3.370127	-1.011439
C	2.254505	-3.041291	-1.634771
C	1.188885	-4.122587	-1.545679
C	3.854989	-1.409681	3.703067
C	2.543427	-0.620749	3.899412
C	1.441860	-1.333799	4.666390
C	4.294028	-4.550144	-1.732112
C	5.739769	-4.803481	-1.335285
C	4.492009	-1.762627	5.053146
C	5.901670	-2.327037	4.969351
C	4.938056	2.176687	-4.453363
C	5.732568	1.067976	-5.128091
C	3.147238	5.195226	1.734512
C	4.536647	5.681489	1.354530
Ca	2.227935	0.113118	0.194088
N	0.028988	-0.108208	-0.530692
N	-0.029974	-0.124140	0.740604
Ca	-2.237826	-0.103332	-0.007109
N	-3.954743	-1.530561	-0.775987
C	-3.496498	-2.781854	-1.264531
C	-3.218944	-3.839548	-0.364662
C	-2.713825	-5.043152	-0.870252
C	-2.491786	-5.221336	-2.228636
C	-2.755437	-4.174788	-3.107164
C	-3.245909	-2.947123	-2.654699
C	-3.403209	-3.687816	1.136455
C	-2.031388	-3.439804	1.794173
C	-2.052283	-3.215178	3.298912
C	-3.443760	-1.791877	-3.624447
C	-3.915943	-2.202054	-5.024934
C	-5.280151	-2.872571	-5.072696
C	-5.252121	-1.246919	-0.833799
C	-6.212183	-2.305132	-1.323891
C	-5.844599	-0.017112	-0.471381
C	-5.311905	1.173617	0.067263
C	-6.306375	2.287448	0.292253
N	-4.037073	1.376806	0.382211
C	-3.651008	2.577577	1.030422
C	-3.761539	2.679493	2.444738
C	-3.278833	3.829503	3.074964
C	-2.694476	4.864233	2.350596
C	-2.598883	4.761174	0.969153
C	-3.077175	3.637242	0.285702
C	-4.355866	1.541964	3.261980
C	-3.281553	0.510544	3.661762
C	-2.231643	0.979235	4.655480
C	-2.930247	3.565011	-1.225308
C	-3.363273	4.852430	-1.942094
C	-4.798041	5.287484	-1.690482
C	-1.482792	3.185142	-1.587370
C	-1.214769	2.945690	-3.064965
C	-5.167715	1.991620	4.483751
C	-6.405373	2.815272	4.163381
C	-4.130454	-4.874233	1.786147
C	-5.507262	-5.178926	1.218331

C	-2.174915	-0.917153	-3.702662
C	-0.941723	-1.569902	-4.305682
H	6.942806	0.545325	0.321602
H	-6.922076	0.015432	-0.630031
H	-6.117914	-3.216279	-0.715653
H	-5.979826	-2.602632	-2.356390
H	-7.251526	-1.958025	-1.283631
H	-3.363067	3.922281	4.159932
H	-2.320377	5.752668	2.865155
H	6.373988	-2.712793	0.549650
H	6.326924	-2.102255	2.196904
H	7.459893	-1.376598	1.023230
H	-5.995604	3.190345	-0.253026
H	-6.348200	2.572243	1.353271
H	-7.313730	2.004431	-0.035747
H	-3.587684	2.752940	-1.582115
H	-4.227014	-1.140393	-3.205064
H	5.725973	3.653306	-0.278622
H	6.090768	2.815367	-1.782345
H	7.125866	2.543040	-0.358241
H	-2.496244	-5.862568	-0.180373
H	-2.571491	-4.317978	-4.174082
H	-4.024758	-2.791046	1.305372
H	-2.106609	-6.172009	-2.605079
H	3.000108	-5.619672	0.401157
H	-5.051562	0.997226	2.604491
H	-2.148805	5.579348	0.400977
H	-1.184389	2.277872	-1.030169
H	-0.802253	3.969168	-1.211591
H	4.595466	1.092537	-2.656514
H	1.767315	5.804040	-3.031151
H	3.186864	-3.980571	4.353014
H	3.462263	3.097637	1.456496
H	3.707310	-5.471984	-1.584482
H	4.243572	-4.347390	-2.816276
H	1.732768	5.737841	-0.555680
H	-4.528859	2.550654	5.187282
H	-5.469702	1.082166	5.032318
H	-1.909376	-0.570468	-2.688614
H	-2.424407	-0.005910	-4.273677
H	1.833165	-2.130606	-1.170133
H	2.418787	-2.774190	-2.693859
H	3.849981	-2.464252	5.610956
H	4.506056	-0.841705	5.662198
H	2.398836	5.966042	1.477776
H	3.093948	5.095082	2.830487
H	2.774730	-5.897955	2.847716
H	2.832566	3.967934	-4.299190
H	4.264121	-2.488573	-1.197377
H	-3.223303	4.702662	-3.024584
H	-2.673151	5.671883	-1.673108
H	5.636237	2.902143	-4.002058
H	4.385286	2.746710	-5.218730
H	2.236725	0.441674	-3.041315
H	3.410296	-0.241733	-4.156747
H	-3.169522	-2.854465	-5.507709
H	-3.939610	-1.288471	-5.644412
H	-3.799837	-0.375728	4.067448
H	-2.767462	0.160819	2.748670
H	-3.494420	-5.775342	1.724293
H	-4.230434	-4.665155	2.863273
H	2.788434	0.328943	4.406460
H	2.139067	-0.337629	2.911136
H	1.047272	2.475451	1.091855
H	0.592543	4.148128	1.256408
H	-1.542258	-2.572225	1.312190
H	-1.367530	-4.289626	1.556016
H	4.545988	-0.714789	3.199919
H	-6.937668	3.106699	5.081887
H	-6.148440	3.738985	3.621698
H	-7.114297	2.247460	3.539889
H	-1.502239	0.179226	4.850203
H	-1.674661	1.848529	4.274917
H	-2.675991	1.258523	5.623011
H	-1.059781	-2.902273	3.654618
H	-2.767391	-2.425569	3.579618
H	-2.328043	-4.124585	3.853403
H	-5.971474	-6.029586	1.740697
H	-6.184345	-4.316815	1.330270
H	-5.459586	-5.432098	0.147880
H	0.534984	-0.712716	4.700955
H	1.171332	-2.286372	4.186306
H	1.734277	-1.548144	5.705813
H	6.299531	-2.553358	5.970573
H	5.931196	-3.258792	4.383024
H	6.592421	-1.611242	4.495753
H	0.248457	-3.778142	-2.000160
H	1.488295	-5.044077	-2.069206
H	0.962512	-4.384372	-0.501201
H	6.165346	-5.649393	-1.896786
H	6.370534	-3.922964	-1.538380
H	5.828711	-5.038993	-0.263140
H	-5.558419	-3.135429	-6.104919
H	-5.297947	-3.799655	-4.478583
H	-6.066661	-2.208414	-4.680041
H	-0.082117	-0.887283	-4.243147
H	-0.666959	-2.488510	-3.766250
H	-1.085441	-1.828713	-5.366320
H	-5.031498	6.215541	-2.234795
H	-5.512862	4.521214	-2.031146
H	-4.987384	5.472273	-0.621673
H	-0.198928	2.548846	-3.205834
H	-1.921229	2.213962	-3.489678
H	-1.291369	3.867953	-3.660322
H	0.221580	2.783692	3.342456
H	1.971497	2.515575	3.485736

H	1.301691	4.152085	3.661746
H	4.770067	6.638515	1.846063
H	5.309527	4.958367	1.661027
H	4.631568	5.833838	0.268222
H	1.343723	0.408129	-5.350154
H	1.497708	2.087317	-4.809228
H	2.686428	1.399491	-5.938757
H	6.459477	1.480259	-5.844583
H	6.294580	0.473457	-4.389328
H	5.084670	0.373171	-5.684831

206

[(BDI*)Ca]2(C6H6)_singlet

C	3.364719	-2.749039	-2.543968
C	3.510047	-2.609624	-1.139049
C	3.002789	-3.608518	-0.264793
C	2.365431	-4.723465	-0.819769
C	2.214389	-4.863172	-2.195240
C	2.709678	-3.878908	-3.042800
N	4.054539	-1.425073	-0.591772
C	5.361853	-1.235463	-0.508070
C	6.329877	-2.287046	-1.009538
C	3.166226	-3.480252	1.242140
C	2.021719	-4.130008	2.032383
C	2.000074	-3.768978	3.510570
C	3.853974	-1.670720	-3.499592
C	4.434638	-2.220687	-4.809790
C	5.608014	-3.175739	-4.652575
C	5.984804	-0.081572	0.019247
C	5.446981	1.097418	0.568389
C	6.453556	2.103667	1.077779
N	4.150902	1.375615	0.672439
C	3.740631	2.581098	1.292690
C	3.522186	3.746568	0.512731
C	2.999006	4.884383	1.138701
C	2.710264	4.896453	2.498448
C	2.977532	3.767603	3.266052
C	3.498753	2.603431	2.692163
C	3.900173	3.783380	-0.959046
C	2.698036	3.576490	-1.901046
C	1.641081	4.669486	-1.918840
C	3.840005	1.400535	3.556624
C	4.583406	1.800388	4.840728
C	5.159128	0.625310	5.617909
C	4.731051	5.030657	-1.303173
C	5.350690	5.004453	-2.692899
C	2.626457	0.487612	3.818186
C	1.522271	1.056819	4.695132
C	4.557525	-3.953082	1.713711
C	4.865042	-5.426114	1.489023
C	2.723180	-0.661220	-3.768025
C	3.126527	0.579121	-4.551637
Ca	2.288622	0.073275	-0.017688
C	0.009293	1.457838	-0.040122
C	0.090799	0.763164	-1.272451
C	-0.025874	-0.744183	-1.235669
C	0.048330	-1.391158	-0.004648
C	0.148424	-0.696007	1.227650
C	0.001056	0.812007	1.195930
H	-0.025533	1.301823	-2.212532
H	0.107630	-1.240086	2.170787
Ca	-2.216955	-0.002691	0.026505
N	-4.057435	-1.468675	0.398082
C	-5.349091	-1.220908	0.229858
C	-5.889018	-0.005642	-0.237660
C	-5.286792	1.209578	-0.627172
N	-3.983478	1.457712	-0.633035
C	-3.467076	2.663095	-1.165277
C	-3.363739	2.833884	-2.572045
C	-2.734045	3.979726	-3.066755
C	-2.226330	4.954037	-2.216063
C	-2.343323	4.788987	-0.840861
C	-2.953953	3.657418	-0.289057
C	-3.872539	1.778482	-3.543615
C	-2.744881	0.782127	-3.867764
C	-3.164800	-0.430551	-4.684900
C	-3.095780	3.518787	1.219515
C	-1.976340	4.221064	2.000332
C	-1.950816	3.906882	3.488969
C	-3.599330	-2.660433	1.003598
C	-3.697043	-2.837525	2.409317
C	-3.084211	-3.950514	2.994994
C	-2.401779	-4.888108	2.231425
C	-2.340609	-4.730146	0.850174
C	-2.930351	-3.635259	0.210565
C	-4.464177	-1.878468	3.307312
C	-3.535520	-1.108201	4.255869
C	-2.611648	-0.138532	3.541765
C	-2.903481	-3.518104	-1.307401
C	-1.725596	-4.256678	-1.956021
C	-1.540718	-3.970374	-3.438524
C	-6.385237	-2.277470	0.556876
C	-4.260053	-3.913193	-1.928664
C	-4.672587	-5.364285	-1.731839
C	-5.575237	-2.615334	4.078929
C	-6.638823	-1.702931	4.673307
C	-6.280164	2.270099	-1.059565
C	-4.486515	2.361708	-4.824784
C	-5.649493	3.320185	-4.618113
C	-4.504460	3.926175	1.700050
C	-4.865627	5.391197	1.505629
H	7.143808	-2.436502	-0.285881
H	6.795778	-1.945762	-1.947473
H	5.843332	-3.250384	-1.206754
H	7.074861	-0.120864	0.011702
H	6.321563	3.075915	0.578663

H	7.483723	1.762720	0.918999
H	6.306630	2.290686	2.152926
H	1.976264	-5.502125	-0.161156
H	1.712781	-5.742629	-2.606544
H	2.588304	-3.994860	-4.122500
H	3.132784	-2.399369	1.469706
H	1.064360	-3.827497	1.577742
H	2.060343	-5.228033	1.933847
H	1.107454	-4.185714	4.001767
H	1.976856	-2.676227	3.651896
H	2.879509	-4.154214	4.049441
H	5.319900	-3.340846	1.209984
H	4.659181	-3.715353	2.786018
H	4.173927	-6.084079	2.039459
H	5.882939	-5.668251	1.831561
H	4.800378	-5.696476	0.423297
H	4.659248	-1.113924	-2.993110
H	3.633233	-2.705969	-5.394965
H	4.757220	-1.364758	-5.423954
H	5.339079	-4.062268	-4.058231
H	6.456750	-2.686397	-4.149736
H	5.965676	-3.526073	-5.633040
H	1.896533	-1.183325	-4.282317
H	2.295179	-0.342518	-2.801404
H	2.293550	1.296879	-4.600402
H	3.416483	0.345655	-5.587131
H	3.977426	1.092147	-4.074248
H	2.833349	5.790384	0.551965
H	2.303299	5.796234	2.966439
H	2.793024	3.800291	4.342037
H	4.555391	2.915152	-1.134131
H	3.085034	3.431441	-2.923812
H	2.212344	2.618120	-1.644406
H	0.833339	4.415870	-2.622046
H	2.054536	5.641879	-2.232240
H	1.174955	4.807955	-0.931453
H	4.120420	5.942968	-1.196288
H	5.531306	5.127634	-0.549714
H	4.589700	5.024637	-3.488264
H	5.959867	4.097846	-2.842530
H	6.007277	5.874088	-2.849486
H	4.540897	0.783491	2.972774
H	5.399819	2.488186	4.561954
H	3.925038	2.385117	5.504913
H	4.373291	-0.031898	6.021488
H	5.761329	0.972661	6.471410
H	5.811680	0.005657	4.981233
H	2.191471	0.191536	2.847292
H	2.992183	-0.453456	4.262676
H	1.099127	1.981699	4.273850
H	1.879872	1.286983	5.711465
H	0.698833	0.333096	4.798536
H	-0.037827	2.551523	-0.063723

H	0.008813	1.387489	2.121653
H	0.038123	-2.485185	0.013368
H	-5.803627	3.229841	-1.292819
H	-7.025863	2.428049	-0.266005
H	-6.832407	1.933328	-1.949960
H	-6.978783	-0.002238	-0.309454
H	-5.941279	-3.271348	0.696709
H	-7.137358	-2.331762	-0.243199
H	-6.921379	-2.013187	1.482002
H	-2.643061	4.115973	-4.146943
H	-1.742461	5.844801	-2.623725
H	-1.949070	5.562901	-0.180342
H	-4.663498	1.202418	-3.037218
H	-1.931859	1.324249	-4.383222
H	-2.300869	0.437427	-2.917808
H	-3.511111	-0.157542	-5.692870
H	-3.980078	-0.982727	-4.189185
H	-2.323213	-1.127576	-4.812499
H	-4.831622	1.521140	-5.447591
H	-3.698474	2.854085	-5.422097
H	-6.029802	3.690894	-5.582459
H	-5.359896	4.194126	-4.014973
H	-6.487938	2.826652	-4.102751
H	-3.010725	2.440365	1.446816
H	-1.007775	3.936491	1.557210
H	-2.048567	5.313836	1.871598
H	-2.844086	4.282419	4.011701
H	-1.074085	4.368281	3.968750
H	-1.893209	2.821889	3.669340
H	-5.240262	3.296594	1.178548
H	-4.599419	3.661782	2.766888
H	-5.897395	5.585599	1.836757
H	-4.794451	5.688690	0.447614
H	-4.209096	6.061444	2.082822
H	-1.822274	-5.483983	0.255598
H	-1.928610	-5.750193	2.707832
H	-3.148114	-4.085792	4.078219
H	-2.774007	-2.445514	-1.543861
H	-0.798817	-3.986241	-1.424466
H	-1.836262	-5.345420	-1.817597
H	-2.381503	-4.338153	-4.046633
H	-0.625942	-4.451611	-3.815992
H	-1.445287	-2.889188	-3.626323
H	-5.035229	-3.252564	-1.514225
H	-4.229301	-3.681488	-3.006611
H	-5.666773	-5.549940	-2.166801
H	-4.721433	-5.630305	-0.664011
H	-3.972746	-6.064887	-2.214309
H	-4.953065	-1.127759	2.666690
H	-1.952212	0.393579	4.244060
H	-3.190528	0.625015	2.996190
H	-1.956853	-0.668292	2.829394
H	-2.941290	-1.827259	4.846781

H	-4.148835	-0.551651	4.983734
H	-6.057344	-3.336957	3.399538
H	-5.121484	-3.223525	4.881120
H	-6.221558	-0.994428	5.405334
H	-7.415489	-2.285526	5.192131
H	-7.137843	-1.107828	3.890861
H	-0.018505	-1.320633	-2.161100

206
[(BDI*)Ca]2(C6H6)_triplet

C	3.454246	-2.726005	-2.565835
C	3.548746	-2.573521	-1.158197
C	3.021138	-3.570431	-0.293483
C	2.413133	-4.695570	-0.859569
C	2.308029	-4.846357	-2.238333
C	2.823465	-3.865002	-3.076662
N	4.068267	-1.382703	-0.597773
C	5.372734	-1.189416	-0.477595
C	6.358835	-2.236492	-0.952903
C	3.136157	-3.429778	1.217046
C	1.989119	-4.106263	1.980458
C	1.920575	-3.742843	3.456880
C	3.975908	-1.658580	-3.517235
C	4.614795	-2.224935	-4.793332
C	5.783653	-3.173571	-4.576018
C	5.978640	-0.035025	0.067934
C	5.422563	1.133765	0.618925
C	6.412227	2.151853	1.137736
N	4.121599	1.391775	0.718178
C	3.692861	2.593705	1.333248
C	3.463843	3.751024	0.543981
C	2.921013	4.885440	1.157756
C	2.622826	4.901599	2.515870
C	2.902374	3.782643	3.292974
C	3.445133	2.621733	2.731070
C	3.849187	3.776238	-0.926483
C	2.660628	3.493248	-1.865749
C	1.572004	4.552415	-1.928116
C	3.811642	1.436054	3.609942
C	4.568314	1.869374	4.875686
C	5.174704	0.717644	5.664357
C	4.622792	5.051219	-1.297999
C	5.241143	5.021777	-2.688274
C	2.616262	0.509589	3.905970
C	1.509999	1.078616	4.780670
C	4.525290	-3.862136	1.732816
C	4.876515	-5.327699	1.524499
C	2.851075	-0.662464	-3.852539
C	3.282697	0.574756	-4.626252
Ca	2.276098	0.091732	-0.018654
C	0.002489	1.474643	-0.069806
C	0.036695	0.732865	-1.301371
C	0.031087	-0.706969	-1.273088
C	0.076779	-1.397158	-0.013126
C	0.081765	-0.655965	1.219266
C	0.000664	0.783200	1.191788
H	-0.001489	1.263986	-2.254083
H	0.091840	-1.191326	2.170315
Ca	-2.199544	-0.021355	-0.026794
N	-4.038742	-1.481900	0.404834
C	-5.331288	-1.248733	0.222122
C	-5.881444	-0.037658	-0.243214
C	-5.289881	1.182865	-0.631929
N	-3.987701	1.433208	-0.663282
C	-3.489535	2.652484	-1.180751
C	-3.414786	2.856656	-2.584445
C	-2.806516	4.021282	-3.063361
C	-2.294694	4.981231	-2.199168
C	-2.381740	4.781652	-0.825988
C	-2.966330	3.629119	-0.290864
C	-3.929671	1.818098	-3.571232
C	-2.796026	0.845086	-3.942798
C	-3.221059	-0.366822	-4.758835
C	-3.066690	3.443575	1.216109
C	-1.950095	4.156223	1.991247
C	-1.880096	3.800594	3.469214
C	-3.573467	-2.677068	0.998177
C	-3.686992	-2.885735	2.398409
C	-3.067886	-4.004947	2.966626
C	-2.365344	-4.917875	2.191809
C	-2.287308	-4.728560	0.815164
C	-2.880381	-3.625738	0.193556
C	-4.489572	-1.967124	3.308443
C	-3.610448	-1.253099	4.344918
C	-2.649224	-0.246639	3.740575
C	-2.833512	-3.471447	-1.320677
C	-1.648783	-4.192655	-1.975670
C	-1.455720	-3.875142	-3.451121
C	-6.359547	-2.320321	0.524573
C	-4.181795	-3.849328	-1.970294
C	-4.592490	-5.306489	-1.820054
C	-5.634368	-2.741885	3.990554
C	-6.729739	-1.861990	4.576107
C	-6.294674	2.244115	-1.035670
C	-4.575947	2.422643	-4.826349
C	-5.749611	3.357486	-4.576799
C	-4.473833	3.793563	1.744043
C	-4.882144	5.251976	1.599761
H	7.152905	-2.382316	-0.206781
H	6.849283	-1.892514	-1.877117
H	5.882867	-3.202086	-1.163969
H	7.068946	-0.065829	0.077291
H	6.267463	3.124966	0.643677
H	7.447749	1.827097	0.979800
H	6.259844	2.330863	2.213394
H	2.009133	-5.472822	-0.208405

H	1.827084	-5.732875	-2.659044
H	2.739880	-3.990969	-4.158825
H	3.069825	-2.348405	1.438656
H	1.035927	-3.832430	1.500332
H	2.061004	-5.202962	1.887286
H	1.020685	-4.172313	3.923063
H	1.877184	-2.650438	3.597972
H	2.790147	-4.113471	4.021354
H	5.286100	-3.232397	1.249228
H	4.588260	-3.617182	2.806435
H	4.189063	-6.000880	2.060887
H	5.891352	-5.541250	1.893976
H	4.845586	-5.604521	0.458958
H	4.754175	-1.087045	-2.985695
H	3.840006	-2.721500	-5.404283
H	4.959170	-1.376152	-5.405696
H	5.494273	-4.050654	-3.977289
H	6.612488	-2.673922	-4.050871
H	6.177881	-3.539507	-5.536559
H	2.057001	-1.197371	-4.403999
H	2.373813	-0.344253	-2.909555
H	2.446337	1.283239	-4.728494
H	3.630187	0.335200	-5.642431
H	4.101920	1.101660	-4.109987
H	2.745316	5.784972	0.563995
H	2.199127	5.798125	2.975157
H	2.711919	3.820922	4.367747
H	4.544465	2.935751	-1.080522
H	3.054636	3.315044	-2.880407
H	2.199342	2.536433	-1.563783
H	0.760870	4.240313	-2.603341
H	1.955153	5.515971	-2.300962
H	1.117604	4.732128	-0.942086
H	3.972597	5.937736	-1.210253
H	5.418473	5.198969	-0.548006
H	4.478760	4.991022	-3.482072
H	5.889479	4.139846	-2.819672
H	5.858497	5.915788	-2.865512
H	4.512298	0.818719	3.026085
H	5.369296	2.565186	4.572964
H	3.910615	2.455518	5.539295
H	4.406057	0.054996	6.091469
H	5.783761	1.089889	6.502357
H	5.827975	0.097550	5.028812
H	2.177612	0.192721	2.943933
H	3.002430	-0.415879	4.365907
H	1.058342	1.979271	4.337255
H	1.874435	1.348674	5.784670
H	0.705494	0.339177	4.917199
H	-0.038318	2.563801	-0.096768
H	0.004126	1.352865	2.122434
H	0.108165	-2.485852	0.007945
H	-5.828100	3.213996	-1.246082

H	-7.041297	2.374343	-0.238150
H	-6.843872	1.924451	-1.934287
H	-6.971484	-0.042335	-0.311126
H	-5.904925	-3.308140	0.672553
H	-7.091243	-2.383484	-0.293671
H	-6.921603	-2.064900	1.436361
H	-2.737636	4.184332	-4.141504
H	-1.829513	5.887432	-2.594542
H	-1.981649	5.543359	-0.154908
H	-4.701966	1.219271	-3.062413
H	-2.009704	1.407165	-4.478358
H	-2.316984	0.498178	-3.010997
H	-3.603013	-0.091348	-5.753235
H	-4.011965	-0.936163	-4.243314
H	-2.372908	-1.049676	-4.918101
H	-4.918014	1.592107	-5.464296
H	-3.806030	2.942305	-5.424138
H	-6.148634	3.749872	-5.524889
H	-5.463696	4.217988	-3.952896
H	-6.573815	2.837564	-4.064484
H	-2.945037	2.361503	1.411306
H	-0.984817	3.914465	1.517342
H	-2.059167	5.249447	1.896522
H	-2.768754	4.138639	4.024423
H	-1.002936	4.269932	3.940452
H	-1.792002	2.712596	3.618032
H	-5.204323	3.156398	1.224640
H	-4.532604	3.497542	2.805164
H	-5.911373	5.406045	1.958816
H	-4.843483	5.580751	0.549306
H	-4.232583	5.925314	2.181206
H	-1.751637	-5.462434	0.211085
H	-1.889544	-5.785548	2.655380
H	-3.145774	-4.166244	4.045410
H	-2.701762	-2.393007	-1.532526
H	-0.727632	-3.930417	-1.431341
H	-1.758388	-5.284184	-1.860938
H	-2.283455	-4.247682	-4.074089
H	-0.528126	-4.331736	-3.828002
H	-1.379671	-2.788882	-3.619334
H	-4.963502	-3.202790	-1.546729
H	-4.137988	-3.586833	-3.040661
H	-5.582051	-5.481152	-2.269824
H	-4.650840	-5.603597	-0.760946
H	-3.886407	-5.991129	-2.316135
H	-4.946517	-1.182655	2.684357
H	-2.027147	0.238489	4.508495
H	-3.195593	0.550600	3.209966
H	-1.965676	-0.728570	3.024180
H	-3.049685	-2.006200	4.925985
H	-4.262690	-0.738649	5.069954
H	-6.080408	-3.433875	3.258331
H	-5.213958	-3.384725	4.783961

H	-6.351107	-1.186022	5.358159
H	-7.525945	-2.470909	5.031276
H	-7.195833	-1.233345	3.799571
H	0.068348	-1.276605	-2.203005

212
[(BDI*)Ca]2(p-Xyl)_singlet

C	3.689086	-2.773609	-2.595315
C	3.593831	-2.599110	-1.188786
C	2.903593	-3.561874	-0.396987
C	2.292743	-4.644994	-1.035423
C	2.351062	-4.800451	-2.417719
C	3.051296	-3.875245	-3.180105
N	4.078593	-1.428273	-0.567178
C	5.378062	-1.205397	-0.425291
C	6.391821	-2.273091	-0.787601
C	2.895817	-3.448914	1.121962
C	4.272981	-3.811327	1.719235
C	4.714225	-5.251820	1.507260
C	4.502719	-1.854183	-3.496626
C	5.594599	-2.656912	-4.233381
C	6.722433	-1.805554	-4.798431
C	5.949293	-0.010975	0.055458
C	5.371727	1.186630	0.523757
C	6.389809	2.253594	0.878569
N	4.071395	1.414172	0.656830
C	3.599383	2.594154	1.275146
C	3.667760	2.737544	2.687904
C	3.073158	3.858099	3.277466
C	2.439717	4.830498	2.512726
C	2.394314	4.694231	1.129299
C	2.961316	3.589280	0.485411
C	4.326245	1.679499	3.563809
C	5.043040	2.253004	4.794492
C	6.148422	3.256385	4.501657
C	2.935697	3.477659	-1.033018
C	4.307929	3.816096	-1.652886
C	4.778698	5.248671	-1.450900
C	3.301235	0.604513	3.968345
C	3.892616	-0.636909	4.620364
C	1.793175	4.264060	-1.689316
C	1.605996	3.983194	-3.173598
C	1.755585	-4.218935	1.799436
C	1.610042	-3.937471	3.287954
C	3.636533	-1.059763	-4.490406
C	2.994176	0.181813	-3.897007
Ca	2.241323	-0.019740	0.055425
C	-0.050159	1.428402	0.067014
C	0.001695	0.726619	1.305168
C	-0.002224	-0.671081	1.323438
C	-0.097412	-1.430539	0.029870
C	0.003052	-0.738077	-1.204483
C	0.043917	0.662176	-1.226924
Ca	-2.268816	0.052272	-0.010087
N	-4.136128	1.293981	-0.884867
C	-3.773117	2.416127	-1.675607
C	-3.582160	3.685566	-1.068460
C	-3.131203	4.748744	-1.858855
C	-2.891384	4.591504	-3.219291
C	-3.141062	3.362701	-3.819369
C	-3.591220	2.265857	-3.075599
C	-3.920871	3.911454	0.396251
C	-4.780606	5.168277	0.603567
C	-5.347292	5.307926	2.009060
C	-3.941961	0.960916	-3.771665
C	-2.706971	0.091697	-4.074486
C	-1.748578	0.609345	-5.136526
C	-5.432930	1.051905	-0.696936
C	-6.450778	1.978260	-1.325289
C	-5.974301	-0.013492	0.045331
C	-5.374383	-1.078744	0.752920
C	-6.375714	-2.000724	1.420500
N	-4.071605	-1.291283	0.862421
C	-3.566255	-2.389099	1.600191
C	-3.097571	-3.537312	0.904522
C	-2.472000	-4.553171	1.634901
C	-2.310562	-4.463363	3.013399
C	-2.800869	-3.353024	3.689719
C	-3.444160	-2.312049	3.012596
C	-3.332572	-3.691704	-0.591033
C	-4.754124	-4.214822	-0.890474
C	-5.045844	-5.632031	-0.420372
C	-3.980401	-1.120741	3.794196
C	-2.884591	-0.056098	3.966776
C	-3.358751	1.279944	4.519688
C	-2.249365	-4.512524	-1.302427
C	-2.394696	-4.561158	-2.817413
C	-4.601612	-1.500872	5.146520
C	-5.750096	-2.496482	5.087256
C	-2.681269	3.863798	1.309187
C	-1.687259	5.007363	1.180517
C	-4.841528	1.186635	-4.997477
C	-5.412099	-0.091420	-5.595037
H	-7.148147	-2.309757	0.701179
H	-6.894676	-1.461884	2.228542
H	-5.910776	-2.895709	1.851306
H	-7.064613	-0.027153	0.074943
H	-6.308426	3.013338	-0.979515
H	-7.476222	1.668190	-1.091293
H	-6.332560	2.005011	-2.419285
H	-2.105708	-5.440796	1.116246
H	-1.819273	-5.270281	3.562735
H	-2.691668	-3.299040	4.775503
H	-3.299212	-2.674446	-1.021853
H	-1.266424	-4.088291	-1.041010
H	-2.235902	-5.546889	-0.921093

H	-1.508326	-5.019952	-3.281745
H	-2.507542	-3.552369	-3.248486
H	-3.270325	-5.148476	-3.133639
H	-5.479731	-3.525250	-0.437555
H	-4.928291	-4.144236	-1.977287
H	-4.400446	-6.375491	-0.914864
H	-6.087743	-5.909644	-0.642910
H	-4.899055	-5.737192	0.666034
H	-4.774434	-0.654909	3.188884
H	-3.813066	-1.881010	5.820104
H	-4.962589	-0.576283	5.624877
H	-5.442945	-3.450935	4.633271
H	-6.590450	-2.103824	4.494279
H	-6.133806	-2.714574	6.095844
H	-2.083869	-0.470371	4.605598
H	-2.406934	0.106204	2.986129
H	-2.537708	2.012848	4.535096
H	-3.735338	1.197637	5.550277
H	-4.169158	1.700970	3.902164
H	-2.987364	5.731167	-1.404228
H	-2.542078	5.436248	-3.818251
H	-3.005950	3.260823	-4.898288
H	-4.540353	3.055892	0.708497
H	-3.021801	3.793229	2.356428
H	-2.151877	2.919003	1.097311
H	-0.837361	4.860383	1.864862
H	-2.139831	5.981747	1.425469
H	-1.274963	5.075433	0.162230
H	-4.205594	6.075540	0.353303
H	-5.610527	5.141683	-0.123139
H	-4.557666	5.451384	2.762904
H	-5.921451	4.412769	2.299710
H	-6.024582	6.172940	2.079066
H	-4.536883	0.374583	-3.053981
H	-5.670316	1.849458	-4.695927
H	-4.297790	1.742229	-5.779845
H	-4.628401	-0.738327	-6.018983
H	-6.121272	0.133344	-6.406428
H	-5.951853	-0.681458	-4.836260
H	-2.148763	-0.040007	-3.132375
H	-3.053001	-0.917031	-4.358897
H	-1.292537	1.567555	-4.844297
H	-2.248009	0.757923	-6.107086
H	-0.930212	-0.107633	-5.305104
H	0.061213	2.511598	0.059077
H	0.050097	1.201976	-2.175369
C	0.061038	-1.533309	-2.477275
H	-0.052788	-2.517678	0.043911
H	5.927826	3.190986	1.210689
H	7.024631	2.463934	0.004462
H	7.058642	1.895081	1.675385
H	7.041476	-0.010544	0.062887
H	5.926552	-3.250408	-0.967920

H	7.132012	-2.375721	0.019132
H	6.946834	-1.987530	-1.694486
H	3.109743	3.975151	4.362922
H	1.985623	5.700256	2.993788
H	1.903699	5.468903	0.538305
H	5.087704	1.164849	2.957138
H	2.548083	1.062697	4.633743
H	2.743689	0.296586	3.066519
H	4.362400	-0.419459	5.591365
H	4.658236	-1.096493	3.974041
H	3.114418	-1.394104	4.800436
H	5.474474	1.409822	5.357149
H	4.301745	2.701887	5.479282
H	6.609820	3.617322	5.433795
H	5.772986	4.133990	3.953642
H	6.947430	2.804836	3.893421
H	2.762887	2.411286	-1.277168
H	0.855255	4.030907	-1.160850
H	1.949327	5.346942	-1.550112
H	2.451676	4.344452	-3.778897
H	0.697303	4.476524	-3.550706
H	1.499306	2.903699	-3.367132
H	5.054469	3.123185	-1.240001
H	4.267420	3.589882	-2.731645
H	5.781103	5.393916	-1.882256
H	4.835350	5.509194	-0.382185
H	4.110115	5.978828	-1.933720
H	1.760557	-5.389841	-0.441837
H	1.861233	-5.652175	-2.895990
H	3.111824	-4.011580	-4.263326
H	2.743638	-2.380489	1.375225
H	0.807925	-3.970402	1.296451
H	1.893952	-5.303584	1.654278
H	2.469860	-4.301510	3.871141
H	0.709226	-4.424587	3.689256
H	1.510651	-2.857055	3.481220
H	5.025141	-3.131295	1.295936
H	4.254412	-3.585740	2.798298
H	5.722733	-5.414210	1.917754
H	4.743768	-5.513444	0.437659
H	4.043559	-5.970672	2.003926
H	5.013579	-1.114772	-2.860860
H	2.358738	0.700629	-4.631556
H	3.758315	0.897009	-3.552505
H	2.355427	-0.060075	-3.033150
H	2.863212	-1.724506	-4.912654
H	4.264588	-0.754052	-5.343741
H	6.017199	-3.404033	-3.542353
H	5.129287	-3.239044	-5.048263
H	6.360664	-1.061210	-5.524758
H	7.468959	-2.427949	-5.315307
H	7.245948	-1.253168	-4.000916
C	0.053481	1.510655	2.585163

H	-0.048264	-1.213165	2.269461
H	0.107565	0.851129	3.461145
H	0.922462	2.187375	2.595771
H	-0.837089	2.150218	2.684284
H	-0.838931	-2.157787	-2.584772
H	0.140728	-0.885305	-3.358612
H	0.918064	-2.225147	-2.467434

212
[(BDI*)Ca]2(p-Xyl)_triplet

C	-3.622184	-2.781796	2.607252
C	-3.549114	-2.624907	1.197233
C	-2.879465	-3.602049	0.406904
C	-2.258544	-4.676797	1.049990
C	-2.291448	-4.813010	2.435042
C	-2.976812	-3.875916	3.196568
N	-4.044738	-1.458759	0.574416
C	-5.347608	-1.241652	0.457277
C	-6.349781	-2.314774	0.837205
C	-2.904921	-3.515546	-1.113063
C	-4.284074	-3.924220	-1.674783
C	-4.692156	-5.367368	-1.418983
C	-4.418579	-1.845543	3.506654
C	-5.473037	-2.633902	4.308922
C	-6.590553	-1.775940	4.884634
C	-5.934663	-0.048606	-0.007876
C	-5.375896	1.158846	-0.473849
C	-6.413302	2.208710	-0.825767
N	-4.079552	1.410292	-0.606686
C	-3.636393	2.593528	-1.240977
C	-3.716279	2.714650	-2.655660
C	-3.155167	3.841000	-3.265514
C	-2.542502	4.841067	-2.519727
C	-2.487467	4.727143	-1.134932
C	-3.024214	3.618964	-0.470572
C	-4.356263	1.627431	-3.509508
C	-5.076873	2.161077	-4.755518
C	-6.203412	3.147782	-4.487173
C	-2.995390	3.538747	1.049409
C	-4.368583	3.880233	1.665035
C	-4.855335	5.302578	1.430937
C	-3.316759	0.555696	-3.883568
C	-3.892643	-0.710890	-4.499890
C	-1.855160	4.345204	1.685125
C	-1.644670	4.077179	3.168466
C	-1.756275	-4.267097	-1.797117
C	-1.624237	-3.984440	-3.286354
C	-3.528101	-1.002345	4.434232
C	-2.880976	0.186473	3.746247
Ca	-2.220362	-0.007261	-0.017394
C	0.001072	1.458355	-0.020922
C	0.021079	0.787408	-1.297793
C	0.004778	-0.657166	-1.298920
C	0.063363	-1.392157	-0.065737
C	0.064744	-0.724195	1.210861
C	-0.006110	0.720355	1.215483
Ca	2.270505	0.084680	-0.019590
N	4.153775	1.330293	0.817310
C	3.808918	2.466906	1.595429
C	3.614971	3.725674	0.968805
C	3.185435	4.806741	1.746432
C	2.969297	4.675800	3.113604
C	3.219654	3.455850	3.731210
C	3.648734	2.342212	3.000167
C	3.917765	3.914135	-0.508331
C	4.732477	5.187990	-0.780090
C	5.265366	5.292061	-2.201825
C	3.991700	1.044140	3.711919
C	2.748154	0.191057	4.025197
C	1.791355	0.737816	5.073880
C	5.446535	1.072580	0.625058
C	6.476992	2.004977	1.223184
C	5.973302	-0.014602	-0.095935
C	5.359308	-1.096634	-0.764816
C	6.347635	-2.037137	-1.425952
N	4.054903	-1.311519	-0.844153
C	3.536766	-2.419811	-1.557126
C	3.069285	-3.553310	-0.836967
C	2.436571	-4.580512	-1.544654
C	2.263652	-4.514248	-2.923127
C	2.747438	-3.415577	-3.622202
C	3.399299	-2.364733	-2.969031
C	3.305156	-3.675451	0.662205
C	4.742256	-4.145951	0.973255
C	5.082642	-5.556134	0.515124
C	3.922473	-1.184722	-3.775989
C	2.822376	-0.123843	-3.947649
C	3.282252	1.196237	-4.549032
C	2.249771	-4.524076	1.383800
C	2.407584	-4.569841	2.897639
C	4.523036	-1.584735	-5.132031
C	5.670243	-2.582088	-5.076592
C	2.657330	3.798365	-1.385071
C	1.625971	4.907038	-1.247223
C	4.894051	1.276784	4.934232
C	5.457220	0.001043	5.543624
H	7.133695	-2.325676	-0.712971
H	6.850677	-1.521215	-2.258511
H	5.874905	-2.943887	-1.822213
H	7.062973	-0.033977	-0.140105
H	6.331671	3.035456	0.864979
H	7.497697	1.688002	0.978144
H	6.375718	2.047528	2.318414
H	2.072575	-5.458452	-1.008677
H	1.766541	-5.329839	-3.453961
H	2.626166	-3.378747	-4.707397

H	3.237398	-2.653322	1.078917
H	1.251358	-4.131077	1.130805
H	2.264065	-5.558836	1.003707
H	1.534144	-5.047809	3.366882
H	2.500383	-3.559665	3.329381
H	3.297454	-5.139508	3.206251
H	5.446264	-3.435371	0.519240
H	4.908072	-4.060929	2.060194
H	4.462908	-6.317184	1.015616
H	6.133342	-5.796178	0.739773
H	4.939266	-5.675184	-0.570391
H	4.725647	-0.708700	-3.190956
H	3.723930	-1.971592	-5.789168
H	4.879184	-0.667577	-5.627732
H	5.368690	-3.529761	-4.605018
H	6.520527	-2.183200	-4.502212
H	6.037978	-2.814564	-6.087905
H	2.005051	-0.553842	-4.554014
H	2.369453	0.070498	-2.960420
H	2.461818	1.929611	-4.555952
H	3.626209	1.086859	-5.588431
H	4.112498	1.630387	-3.967899
H	3.038256	5.781165	1.275974
H	2.636180	5.533554	3.703145
H	3.100434	3.373746	4.813650
H	4.556055	3.067673	-0.807477
H	2.969413	3.718570	-2.440187
H	2.171744	2.835994	-1.148282
H	0.755851	4.712672	-1.892789
H	2.034725	5.888642	-1.535862
H	1.253542	4.990552	-0.214764
H	4.135274	6.086523	-0.551522
H	5.577557	5.212567	-0.071048
H	4.456525	5.384234	-2.943195
H	5.860523	4.404354	-2.472267
H	5.914233	6.173688	-2.317921
H	4.580842	0.443439	3.001345
H	5.726709	1.931365	4.625207
H	4.354832	1.843000	5.712073
H	4.669776	-0.636787	5.974228
H	6.168376	0.229186	6.352321
H	5.992639	-0.599662	4.790164
H	2.193570	0.041528	3.083625
H	3.084136	-0.814926	4.330304
H	1.349116	1.697241	4.764658
H	2.288064	0.895482	6.044458
H	0.963150	0.034033	5.249238
H	-0.038722	2.548142	0.006200
H	0.011671	1.262136	2.163852
C	0.035196	-1.530485	2.480136
H	0.091041	-2.481808	-0.095728
H	-5.968903	3.153212	-1.161620
H	-7.046927	2.409908	0.051417

H	-7.080486	1.838061	-1.618378
H	-7.026822	-0.059562	-0.004275
H	-5.877282	-3.289863	1.010369
H	-7.103522	-2.421282	0.043586
H	-6.890426	-2.031413	1.753602
H	-3.201109	3.940646	-4.352279
H	-2.112851	5.714205	-3.016767
H	-2.016468	5.524265	-0.557722
H	-5.113932	1.117402	-2.894292
H	-2.568721	1.004590	-4.560430
H	-2.755276	0.282352	-2.973354
H	-4.369771	-0.524548	-5.473735
H	-4.648726	-1.164514	-3.838366
H	-3.104840	-1.461196	-4.665072
H	-5.489348	1.298274	-5.302373
H	-4.341350	2.610102	-5.446330
H	-6.669386	3.479533	-5.427847
H	-5.847848	4.044060	-3.956481
H	-6.994706	2.692584	-3.871466
H	-2.814146	2.479059	1.310212
H	-0.921224	4.119649	1.145988
H	-2.025747	5.425111	1.539534
H	-2.495081	4.415037	3.780621
H	-0.748077	4.599560	3.535432
H	-1.503149	3.002171	3.364374
H	-5.109417	3.170346	1.270506
H	-4.323223	3.680077	2.748884
H	-5.855916	5.448843	1.866224
H	-4.922486	5.535665	0.356546
H	-4.190540	6.051204	1.890197
H	-1.741058	-5.432327	0.456561
H	-1.794163	-5.658633	2.916406
H	-3.019104	-3.997108	4.282427
H	-2.786632	-2.447982	-1.380389
H	-0.809420	-4.001198	-1.300928
H	-1.873300	-5.354024	-1.649522
H	-2.494547	-4.337422	-3.860706
H	-0.733555	-4.480136	-3.699647
H	-1.515033	-2.904686	-3.477507
H	-5.042767	-3.249107	-1.254256
H	-4.290422	-3.725787	-2.759436
H	-5.701409	-5.562496	-1.813187
H	-4.704049	-5.600359	-0.342500
H	-4.011624	-6.085065	-1.903862
H	-4.962759	-1.135497	2.865466
H	-2.234303	0.752954	4.433932
H	-3.641791	0.880030	3.353749
H	-2.247003	-0.132433	2.904548
H	-2.755421	-1.648368	4.885947
H	-4.138630	-0.631543	5.274405
H	-5.910795	-3.407294	3.657056
H	-4.974059	-3.184070	5.126007
H	-6.213245	-1.006128	5.575607

H	-7.313128	-2.389123	5.444737
H	-7.145728	-1.253859	4.088121
C	-0.080894	1.569220	-2.578682
H	0.049545	-1.198414	-2.246764
H	-0.123742	0.894010	-3.445300
H	-0.981678	2.205740	-2.608063
H	0.775900	2.244975	-2.734007
H	0.945326	-2.137250	2.617040
H	-0.054492	-0.880190	3.360553
H	-0.810750	-2.238876	2.501432

224

[(BDI*)Ca]2(DBA)_singlet (4)

Ca	-2.393527	-0.000189	-0.000527
N	-4.214559	1.585815	0.102315
N	-4.214039	-1.586557	-0.102544
C	-6.550954	2.385163	0.217016
H	-6.786192	2.559237	1.277836
H	-7.483696	2.092061	-0.281992
H	-6.209328	3.343014	-0.192632
C	-5.512073	1.288102	0.091612
C	-6.071455	-0.000712	0.000369
H	-7.161572	-0.000794	0.000614
C	-5.511745	-1.289262	-0.091327
C	-6.549643	-2.387166	-0.216630
H	-6.775765	-2.570408	-1.277842
H	-7.486479	-2.090655	0.272458
H	-6.210928	-3.341634	0.203305
C	-3.869056	2.956182	0.271231
C	-3.541181	3.749018	-0.860985
C	-3.196862	5.089389	-0.653213
H	-2.952984	5.721188	-1.508061
C	-3.150701	5.641433	0.620804
H	-2.869429	6.688849	0.755261
C	-3.473407	4.857174	1.720492
H	-3.446344	5.302850	2.717068
C	-3.856082	3.520759	1.574493
C	-3.581193	3.177281	-2.276328
H	-3.148322	2.160185	-2.214749
C	-2.714528	3.968852	-3.267357
H	-1.727408	4.133419	-2.810451
H	-3.143144	4.973274	-3.420556
C	-2.534476	3.307783	-4.626321
H	-2.138736	2.284266	-4.529650
H	-1.824257	3.877100	-5.245555
H	-3.476683	3.243910	-5.191653
C	-5.014816	2.989811	-2.817151
H	-5.557966	2.295054	-2.162657
H	-4.949527	2.469765	-3.787428
C	-5.818282	4.270905	-2.987128
H	-6.847082	4.047325	-3.308853
H	-5.380469	4.939217	-3.745256
H	-5.879936	4.841167	-2.046468
C	-4.254786	2.707841	2.801974
H	-4.968754	1.934832	2.473559
C	-4.949249	3.535957	3.894320
H	-5.161545	2.866139	4.742347
H	-4.239923	4.281545	4.294440
C	-6.245217	4.223324	3.491576
H	-7.022975	3.489235	3.227491
H	-6.108677	4.890392	2.626939
H	-6.642187	4.828516	4.321204
C	-3.046348	1.954876	3.383257
H	-2.496577	1.474277	2.555973
H	-2.334603	2.690102	3.798032
C	-3.389377	0.905219	4.430575
H	-4.097486	0.160142	4.031203
H	-3.845273	1.344360	5.330358
H	-2.487350	0.367600	4.756776
C	-3.868818	-2.956963	-0.271630
C	-3.856050	-3.521383	-1.574966
C	-3.474103	-4.857997	-1.721117
H	-3.447208	-5.303576	-2.717734
C	-3.151849	-5.642552	-0.621512
H	-2.871143	-6.690106	-0.756081
C	-3.197480	-5.090557	0.652555
H	-2.953671	-5.722539	1.507286
C	-3.541014	-3.750015	0.860509
C	-4.253858	-2.707979	-2.802454
H	-4.967717	-1.934780	-2.474185
C	-4.947852	-3.535539	-3.895517
H	-4.238572	-4.281435	-4.295104
H	-5.159018	-2.865483	-4.743623
C	-6.244557	-4.222313	-3.494128
H	-6.640756	-4.827600	-4.324050
H	-7.022406	-3.487862	-3.231240
H	-6.109248	-4.889266	-2.629194
C	-3.044866	-1.955249	-3.382894
H	-2.333121	-2.690582	-3.797479
H	-2.495366	-1.475080	-2.555172
C	-3.386943	-0.905173	-4.430112
H	-3.842605	-1.343861	-5.330234
H	-2.484517	-0.367892	-4.755731
H	-4.094917	-0.159869	-4.030911
C	-3.579436	-3.178202	2.275933
H	-3.145310	-2.161668	2.213957
C	-5.012299	-2.988887	2.818158
H	-5.555421	-2.293865	2.163926
H	-4.945377	-2.468384	3.788075
C	-5.816926	-4.269040	2.989827
H	-5.880044	-4.840015	2.049674
H	-6.845195	-4.044179	3.312358
H	-5.379084	-4.937247	3.748017
C	-2.712960	-3.970833	3.266301
H	-1.726342	-4.136519	2.808739
H	-3.142626	-4.974774	3.419706

C	-2.531179	-3.310109	4.625198
H	-1.821330	-3.880421	5.243946
H	-3.472939	-3.245026	5.191129
H	-2.134117	-2.287118	4.528354
C	0.000136	1.340377	-0.651827
C	0.000382	2.645961	-1.249245
H	0.000656	2.708548	-2.339990
C	0.000319	3.799568	-0.511954
H	0.000516	4.774260	-1.005571
C	-0.000027	3.734110	0.905594
H	-0.000073	4.655741	1.491167
C	-0.000313	2.516251	1.529819
H	-0.000630	2.485569	2.621299
C	-0.000214	1.269954	0.812652
C	0.000878	0.225716	-3.141655
H	0.000059	-0.733690	-3.679567
H	0.876959	0.790007	-3.504698
H	-0.873743	0.791910	-3.505232
B	0.000349	0.084661	-1.545531
C	-0.000098	-1.340168	0.651481
C	-0.000325	-2.645761	1.248887
H	-0.000634	-2.708363	2.339630
C	-0.000198	-3.799360	0.511579
H	-0.000372	-4.774061	1.005178
C	0.000173	-3.733896	-0.905970
H	0.000269	-4.655525	-1.491548
C	0.000413	-2.516032	-1.530182
H	0.000735	-2.485339	-2.621662
C	0.000272	-1.269740	-0.813005
C	-0.000835	-0.225474	3.141309
H	-0.000809	0.733940	3.679207
H	-0.876437	-0.790512	3.504345
H	0.874271	-0.790901	3.504916
B	-0.000329	-0.084447	1.545184
Ca	2.393490	0.000239	0.000267
N	4.214520	-1.585821	-0.102263
N	4.214209	1.586471	0.102616
C	6.550783	-2.385461	-0.216416
H	6.784742	-2.560983	-1.277266
H	7.484118	-2.091872	0.281187
H	6.209493	-3.342766	0.194788
C	5.512078	-1.288232	-0.091206
C	6.071545	0.000510	0.000281
H	7.161664	0.000542	0.000346
C	5.511885	1.289116	0.091690
C	6.549954	2.386871	0.217105
H	6.777814	2.568294	1.278268
H	7.486022	2.091037	-0.273886
H	6.210678	3.342014	-0.200828
C	3.869049	-2.956204	-0.271187
C	3.541020	-3.749004	0.861024
C	3.196806	-5.089401	0.653266
H	2.952809	-5.721167	1.508101

C	3.150805	-5.641502	-0.620737
H	2.869573	-6.688929	-0.755189
C	3.473574	-4.857260	-1.720417
H	3.446581	-5.302959	-2.716985
C	3.856198	-3.520829	-1.574422
C	3.580590	-3.177191	2.276363
H	3.147610	-2.160159	2.214609
C	2.713732	-3.968801	3.267193
H	1.726778	-4.133595	2.810012
H	3.142483	-4.973129	3.420627
C	2.533137	-3.307617	4.626028
H	2.137063	-2.284252	4.529128
H	1.822963	-3.877096	5.245165
H	3.475189	-3.243351	5.191577
C	5.014016	-2.989504	2.817658
H	5.557337	-2.294813	2.163243
H	4.948327	-2.469290	3.787823
C	5.817541	-4.270488	2.988173
H	6.846141	-4.046751	3.310419
H	5.379411	-4.938779	3.746137
H	5.879758	-4.840855	2.047608
C	4.254821	-2.707916	-2.801941
H	4.968760	-1.934856	-2.473568
C	4.949222	-3.536048	-3.894317
H	5.161432	-2.866245	-4.742371
H	4.239865	-4.281643	-4.294355
C	6.245244	-4.223404	-3.491719
H	7.023069	-3.489318	-3.227817
H	6.108822	-4.890425	-2.627024
H	6.642064	-4.828662	-4.321367
C	3.046303	-1.955044	-3.383185
H	2.496532	-1.474475	-2.555876
H	2.334595	-2.690332	-3.797920
C	3.389186	-0.905370	-4.430526
H	4.096923	-0.159984	-4.031078
H	3.845457	-1.344424	-5.330161
H	2.487037	-0.368132	-4.756997
C	3.868906	2.956875	0.271594
C	3.855984	3.521365	1.574910
C	3.473903	4.857942	1.720958
H	3.446875	5.303571	2.717551
C	3.151714	5.642431	0.621280
H	2.870918	6.689970	0.755779
C	3.197556	5.090396	-0.652757
H	2.953842	5.722326	-1.507551
C	3.541207	3.749864	-0.860607
C	4.253814	2.708051	2.802440
H	4.967726	1.934887	2.474217
C	4.947801	3.535733	3.895429
H	4.238502	4.281618	4.295012
H	5.159061	2.865754	4.743574
C	6.244450	4.222527	3.493878
H	6.640780	4.827803	4.323742

H	7.022244	3.488075	3.230843
H	6.109011	4.889478	2.628965
C	3.044885	1.955277	3.382944
H	2.333284	2.690568	3.797869
H	2.495162	1.475336	2.555241
C	3.387161	0.904932	4.429823
H	3.843514	1.343334	5.329735
H	2.484734	0.367936	4.755912
H	4.094634	0.159431	4.030112
C	3.580017	3.178047	-2.276009
H	3.146133	2.161399	-2.214137
C	5.013063	2.989071	-2.817868
H	5.556135	2.294112	-2.163534
H	4.946517	2.468639	-3.787850
C	5.817499	4.269390	-2.989171
H	5.880324	4.840210	-2.048906
H	6.845874	4.044755	-3.311527
H	5.379701	4.937639	-3.747351
C	2.713559	3.970464	-3.266565
H	1.726813	4.135931	-2.809195
H	3.143029	4.974499	-3.419903
C	2.532202	3.309682	-4.625489
H	1.822384	3.879850	-5.244403
H	3.474101	3.244766	-5.191211
H	2.135306	2.286618	-4.528710

224

[(BDI*)Ca]2(DBA)_triplet (4)

Ca	-2.431458	0.000034	-0.000212
N	-4.238370	1.583581	0.102639
N	-4.238558	-1.583158	-0.103022
C	-6.569826	2.390414	0.216179
H	-6.752611	2.617017	1.277197
H	-7.524994	2.078510	-0.224989
H	-6.245936	3.327587	-0.252190
C	-5.536899	1.288605	0.091280
C	-6.097750	0.000324	-0.000340
H	-7.187569	0.000444	-0.000402
C	-5.537096	-1.288013	-0.091790
C	-6.569912	-2.389912	-0.216558
H	-6.748990	-2.620417	-1.277346
H	-7.526512	-2.076440	0.220329
H	-6.247701	-3.325512	0.256132
C	-3.892629	2.954347	0.270957
C	-3.565093	3.746115	-0.862013
C	-3.231430	5.089554	-0.656163
H	-2.989189	5.721197	-1.511552
C	-3.194265	5.645018	0.616571
H	-2.921927	6.694955	0.749401
C	-3.508195	4.859278	1.717493
H	-3.483227	5.306256	2.713538
C	-3.880320	3.519317	1.573652
C	-3.593589	3.169879	-2.275898
H	-3.160273	2.153254	-2.207782
C	-2.718196	3.959297	-3.260847
H	-1.734046	4.121592	-2.796650
H	-3.143079	4.964808	-3.416930
C	-2.530427	3.298512	-4.618876
H	-2.141542	2.272606	-4.520063
H	-1.811622	3.864310	-5.231391
H	-3.468219	3.241026	-5.192204
C	-5.021959	2.978977	-2.829080
H	-5.570772	2.286790	-2.176397
H	-4.947244	2.454469	-3.796263
C	-5.824460	4.258780	-3.012864
H	-6.849911	4.033135	-3.343680
H	-5.379404	4.923721	-3.769733
H	-5.895675	4.833756	-2.075689
C	-4.265868	2.702523	2.802823
H	-4.977640	1.925492	2.478603
C	-4.956760	3.524506	3.902006
H	-5.156807	2.852259	4.751026
H	-4.249365	4.274746	4.296757
C	-6.261324	4.202787	3.511706
H	-7.036014	3.463121	3.253883
H	-6.137402	4.872335	2.647051
H	-6.655466	4.803892	4.345641
C	-3.048425	1.956068	3.374032
H	-2.501705	1.478787	2.542386
H	-2.337438	2.694996	3.783361
C	-3.377224	0.904063	4.423457
H	-4.084997	0.155694	4.029624
H	-3.827587	1.340136	5.327514
H	-2.469733	0.370758	4.741424
C	-3.893247	-2.954042	-0.271243
C	-3.881105	-3.519039	-1.573945
C	-3.509635	-4.859183	-1.717739
H	-3.484791	-5.306199	-2.713767
C	-3.196173	-5.645057	-0.616775
H	-2.924374	-6.695140	-0.749564
C	-3.233047	-5.089528	0.655935
H	-2.991083	-5.721267	1.511331
C	-3.566019	-3.745905	0.861751
C	-4.266090	-2.701992	-2.803144
H	-4.977838	-1.924884	-2.479050
C	-4.956750	-3.523646	-3.902722
H	-4.249470	-4.274165	-4.297140
H	-5.156067	-2.851279	-4.751817
C	-6.261819	-4.201375	-3.513143
H	-6.655880	-4.802140	-4.347362
H	-7.036271	-3.461373	-3.255549
H	-6.138608	-4.871156	-2.648566
C	-3.048386	-1.955598	-3.373872
H	-2.337582	-2.694500	-3.783562
H	-2.501558	-1.478925	-2.541953
C	-3.376859	-0.902927	-4.422732

H	-3.827575	-1.338344	-5.326930
H	-2.469176	-0.369882	-4.740579
H	-4.084236	-0.154436	-4.028419
C	-3.593774	-3.169535	2.275618
H	-3.159546	-2.153310	2.207309
C	-5.021809	-2.977297	2.829186
H	-5.570215	-2.284740	2.176551
H	-4.946357	-2.452686	3.796255
C	-5.825332	-4.256390	3.013472
H	-5.897089	-4.831654	2.076508
H	-6.850573	-4.029815	3.344302
H	-5.380741	-4.921434	3.770523
C	-2.718858	-3.959662	3.260430
H	-1.734971	-4.122933	2.796018
H	-3.144638	-4.964764	3.416707
C	-2.530118	-3.298935	4.618350
H	-1.811853	-3.865481	5.230807
H	-3.467745	-3.240322	5.191833
H	-2.140076	-2.273486	4.519355
C	0.000304	1.380817	-0.641979
C	0.000679	2.655435	-1.238373
H	0.000909	2.719489	-2.328982
C	0.000757	3.844202	-0.488980
H	0.001027	4.813007	-0.993392
C	0.000472	3.776939	0.894061
H	0.000508	4.691066	1.491472
C	0.000133	2.527777	1.527643
H	-0.000081	2.499617	2.619187
C	0.000068	1.307593	0.812830
C	0.000297	0.232751	-3.123677
H	-0.000276	-0.721876	-3.669908
H	0.874753	0.804554	-3.481736
H	-0.873385	0.805700	-3.481778
B	0.000170	0.081756	-1.533497
C	-0.000296	-1.380639	0.641895
C	-0.000665	-2.655259	1.238290
H	-0.000875	-2.719309	2.328898
C	-0.000761	-3.844028	0.488900
H	-0.001019	-4.812832	0.993313
C	-0.000521	-3.776767	-0.894140
H	-0.000592	-4.690896	-1.491549
C	-0.000201	-2.527606	-1.527723
H	-0.000021	-2.499447	-2.619267
C	-0.000088	-1.307416	-0.812914
C	-0.000485	-0.232584	3.123592
H	0.000098	0.722047	3.669817
H	-0.875044	-0.804307	3.481521
H	0.873090	-0.805614	3.481830
B	-0.000134	-0.081584	1.533412
Ca	2.431558	0.000007	0.000142
N	4.238276	-1.583671	-0.102711
N	4.238768	1.583018	0.102884
C	6.569560	-2.390837	-0.216212

H	6.750901	-2.619015	-1.277135
H	7.525325	-2.078460	0.223311
H	6.246158	-3.327332	0.253862
C	5.536846	-1.288825	-0.091422
C	6.097827	-0.000601	0.000084
H	7.187645	-0.000803	0.000098
C	5.537274	1.287784	0.091617
C	6.570194	2.389567	0.216491
H	6.749814	2.619393	1.277338
H	7.526565	2.076272	-0.221032
H	6.247821	3.325460	-0.255500
C	3.892490	-2.954430	-0.270940
C	3.564931	-3.746142	0.862062
C	3.231373	-5.089616	0.656268
H	2.989119	-5.721231	1.511676
C	3.194323	-5.645151	-0.616439
H	2.922084	-6.695121	-0.749220
C	3.508207	-4.859443	-1.717398
H	3.483277	-5.306474	-2.713419
C	3.880215	-3.519443	-1.573615
C	3.593166	-3.169787	2.275906
H	3.159619	-2.153265	2.207651
C	2.717815	-3.959325	3.260790
H	1.733732	-4.121784	2.796512
H	3.142842	-4.964768	3.416926
C	2.529856	-3.298540	4.618797
H	2.141176	-2.272559	4.519929
H	1.810817	-3.864225	5.231141
H	3.467535	-3.241234	5.192329
C	5.021396	-2.978541	2.829308
H	5.570229	-2.286391	2.176605
H	4.946408	-2.453868	3.796381
C	5.824031	-4.258206	3.013498
H	6.849479	-4.032365	3.344187
H	5.379064	-4.922927	3.770613
H	5.895252	-4.833498	2.076514
C	4.265650	-2.702586	-2.802781
H	4.977430	-1.925556	-2.478570
C	4.956488	-3.524478	-3.902065
H	5.156282	-2.852215	-4.751133
H	4.249151	-4.274839	-4.296683
C	6.261228	-4.202526	-3.511955
H	7.035817	-3.462719	-3.254220
H	6.137539	-4.872120	-2.647301
H	6.655374	-4.803544	-4.345951
C	3.048164	-1.956100	-3.373869
H	2.501282	-1.479168	-2.542133
H	2.337315	-2.694977	-3.783528
C	3.376960	-0.903696	-4.422896
H	4.084382	-0.155226	-4.028629
H	3.827746	-1.339368	-5.326936
H	2.469402	-0.370582	-4.740995
C	3.893507	2.953906	0.271182

C	3.881384	3.518836	1.573901
C	3.509961	4.858988	1.717767
H	3.485141	5.305946	2.713824
C	3.196531	5.644927	0.616847
H	2.924778	6.695015	0.749688
C	3.233390	5.089461	-0.655897
H	2.991455	5.721254	-1.511259
C	3.566321	3.745841	-0.861783
C	4.266283	2.701728	2.803077
H	4.977853	1.924463	2.478961
C	4.957140	3.523259	3.902624
H	4.249914	4.273720	4.297256
H	5.156621	2.850788	4.751597
C	6.262121	4.201070	3.512906
H	6.656266	4.801791	4.347116
H	7.036573	3.461124	3.255156
H	6.138764	4.870907	2.648392
C	3.048390	1.955646	3.373811
H	2.337527	2.694775	3.782985
H	2.501759	1.478662	2.541936
C	3.376446	0.903389	4.423219
H	3.826461	1.339203	5.327574
H	2.468669	0.370247	4.740637
H	4.084240	0.154924	4.029608
C	3.594122	3.169541	-2.275681
H	3.159952	2.153289	-2.207427
C	5.022172	2.977404	-2.829249
H	5.570621	2.284858	-2.176640
H	4.946743	2.452824	-3.796337
C	5.825619	4.256553	-3.013498
H	5.897214	4.831859	-2.076548
H	6.850919	4.030040	-3.344185
H	5.381073	4.921521	-3.770640
C	2.719182	3.959642	-3.260494
H	1.735281	4.122865	-2.796099
H	3.144932	4.964759	-3.416756
C	2.530510	3.298904	-4.618421
H	1.812119	3.865324	-5.230846
H	3.468130	3.240470	-5.191934
H	2.140660	2.273381	-4.519425

7. References

S1 S. N. Kessler, M. Neuburger and H. A. Wegner, *Eur. J. Org. Chem.*, 2011, 3238–3245.

S2 J. Hicks, M. Juckel, A. Paparo, D. Dange and C. Jones, *Organometallics*, 2018, **37**, 4810–4813.

S3 W. L. F. Armareg and C. Cha, *Purification of Laboratory Chemicals*, Elsevier, 6th edn., 2009.

S4 B. Rösch, T. X. Gentner, J. Langer, C. Färber, J. Eyselein, L. Zhao, C. Ding, G. Frenking and S. Harder, *Science*, 2021, **371**, 1125–1128.

S5 J. Mai, B. Rösch, N. Patel, J. Langer and S. Harder, *Chem. Sci.*, 2023, **14**, 4724–4734.

S6 T. X. Gentner, B. Rösch, G. Ballmann, J. Langer, H. Elsen and S. Harder, *Angew. Chem. Int. Ed.*, 2019, **58**, 607–611.

S7 Rigaku Oxford Diffraction, *CrysAlisPro Softw. Syst. version 1.171.43.106a*, 2024, Rigaku Corporation, Oxford, UK (all other compound.

S8 O. V. Dolomanov, L. J. Bourhis, R. J. Gildea, J. A. K. Howard and H. Puschmann, *J. Appl. Cryst.*, 2009, **42**, 339–341.

S9 G. M. Sheldrick, *Acta Crystallogr. Sect. A Found. Adv.*, 2015, **71**, 3–8.

S10 G. M. Sheldrick, *Acta Crystallogr. Sect. C Struct. Chem.*, 2015, **71**, 3–8.

S11 A. Thorn, B. Dittrich and G. M. Sheldrick, *Acta Crystallogr. Sect. A Found. Crystallogr.*, 2012, **68**, 448–451.

S12 P. van der Sluis and A. L. Spek, *Acta Crystallogr. Sect. A Found. Crystallogr.*, 1990, **46**, 194–201.

S13 D. J. Frisch, M. J.; Trucks, G. W.; Schlegel, H. B.; Scuseria, G. E.; Robb, M. A.; Cheeseman, J. R.; Scalmani, G.; Barone, V.; Petersson, G. A.; Nakatsuji, H.; Li, X.; Caricato, M.; Marenich, A. V.; Bloino, J.; Janesko, B. G.; Gomperts, R.; Mennucci, B.; Hratch, *Gaussian 16 Rev, Inc., Wallingford CT*, 2016.

S14 C. Adamo and V. Barone, *J. Chem. Phys.*, 1999, **110**, 6158–6170.

S15 M. Ernzerhof and G. E. Scuseria, *J. Chem. Phys.*, 1999, **110**, 5029–5036.

S16 F. Weigend and R. Ahlrichs, *Phys. Chem. Chem. Phys.*, 2005, **7**, 3297–3305.

S17 S. Grimme, J. Antony, S. Ehrlich and H. Krieg, *J. Chem. Phys.*, 2010, **132**, 154104.

S18 S. Grimme, S. Ehrlich and L. Goerigk, *J. Comput. Chem.*, 2011, **32**, 1456–1465.

S19 F. Glendening, E. D.; Badenhoop, J. K.; Reed, A. E.; Carpenter, J. E.; Bohmann, J. A.; Morales, C. M.; Karafiloglou, P.; Landis, C. R.; Weinhold, *Theoretical Chemistry Institute, University of Wisconsin, Madison, WI,* 2018.

S20 R. F. W. Bader, *Chem. Rev.*, 1991, **91**, 893–928.

S21 Keith, T. A. AIMAll (Version 17.01.25), TK Gristmill Software, Overland Park KS USA, 2017.
